# Clonal haematopoiesis is associated with protection against angina pectoris in the UK population

**DOI:** 10.1016/j.jare.2025.08.002

**Published:** 2025-08-05

**Authors:** Yidan Zheng, Zihao Zhou, Xingyu Qian, Jingyu Xu, Shiyan Hu, Chen Jiang, Yuqi Liu, Fuqiang Tong, Ming Chen, Pengning Fan, Zhe Chen, Nianguo Dong, Li Xu, Fei Li

**Affiliations:** a Department of Cardiovascular Surgery, Union Hospital, Tongji Medical College, Huazhong University of Science and Technology, Wuhan, China; b Department of Structural Heart Disease, Fuwai Yunnan Cardiovascular Hospital, Kunming, China

**Keywords:** Clonal haematopoiesis, Angina pectoris, Unstable angina pectoris, Interleukin-1β, Atherosclerotic plaque stability

## Abstract

**Introduction:**

Clonal haematopoiesis of indeterminate potential (CHIP) has been reported to increase the risk of various diseases, including cardiovascular diseases (CVDs). In contrast, a newly published study suggests that CHIP itself may not directly contribute to the risks of CVDs or other age-related conditions. CHIP could also serve as a protective factor against certain diseases, such as Alzheimer’s disease. However, protective effects of CHIP against CVDs have rarely been investigated.

**Objectives:**

To demonstrate the associations with CHIP and lower angina pectoris (AP) risk especially unstable angina pectoris (UAP), and explore potential mechanisms.

**Methods:**

This study was carried out based on UK Biobank cohort. General linear models adjusted for covariates were used to detect associations between CHIP and AP. Propensity score matching models were used for validation. Mendelian randomization was utilized for causality inference. Mediation analysis was conducted to explore underlying mechanisms of cytokines level.

**Results:**

In covariate models, large overall CHIP was associated with reduced AP and UAP risk. Large *TET2* CHIP reduced AP and UAP risk. Propensity score matching models confirmed that large *TET2* CHIP protected against AP and UAP. Mendelian randomization supported the effects of large *TET2* CHIP against UAP. Large *TET2* CHIP was linked to increased IL-1β levels, with IL-1β mediating the protection against UAP.

**Conclusions:**

CHIP protected against AP, with large *TET2* CHIP specifically protecting against UAP. Elevated IL-1β levels might mediate this effect via plaque stabilization.

## Highlights

• First population-based study to explore protection of CHIP in cardiovascular health.
• CHIP could decrease the risk of AP, especially UAP.
• Large *TET2* CHIP was the main subtype account for the protection.
• IL-1β may mediate the protection of CHIP through its atheroprotective effects.
• This study stressed the need for tailored therapies in atherosclerotic disease.

## Graphical abstract

**Figure:**
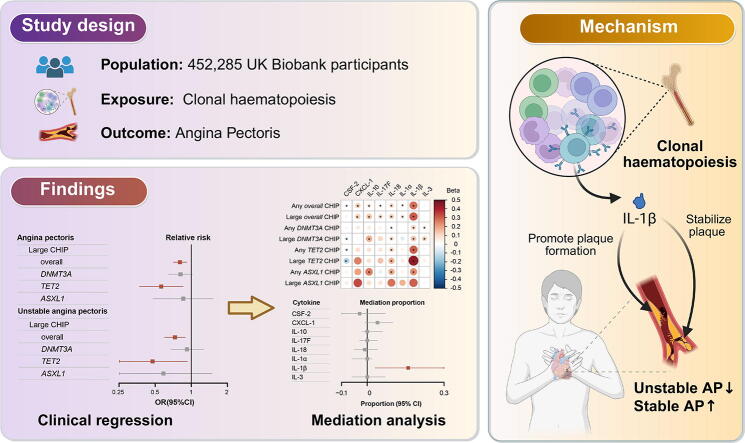

## Introduction

Angina pectoris (AP) is a common manifestation of coronary artery disease, presenting as chest pain due to myocardial ischemia [1]. It is classified into stable angina pectoris (SAP), which occurs with exertion, and unstable angina pectoris (UAP), marked by sudden, unpredictable chest pain and a higher risk of acute myocardial infarction requiring urgent management [2]. The pathophysiology involves genetic, environmental, and inflammatory factors leading to atherosclerotic plaques in the coronary arteries [3], with SAP caused by atherosclerotic plaque buildup and UAP by plaque rupture and thrombosis [4].

Clonal haematopoiesis of indeterminate potential (CHIP) is characterized by somatic mutations in haematopoietic cells, which confer a growth advantage without progressing to overt haematological malignancies [5]. Mutations in CHIP genes, such as *DNMT3A*, *TET2*, and *ASXL1* [6], may mediate aging and cardiovascular diseases through pro-inflammatory pathways. CHIP may contribute to the development of inflammatory disorders through mechanisms such as the nucleotide oligomerization domain-, leucine-rich repeat-, and pyrin domain-containing protein 3 (NLRP3) inflammasome and subsequent interleukin (IL)-1β secretion, worsening atherosclerosis by affecting macrophage behaviour and thickening plaques [7,8]. However, emerging evidence has also illuminated the dichotomous role of IL-1β, conventionally regarded as a pro-inflammatory factor, in modulating plaque stabilization by promoting outward remodelling and preserving a fibrous cap rich in smooth muscle cells (SMCs) and collagen [9,10]. Considering the strong pathophysiological association between plaque stability and AP and UAP, CHIP, a significant IL-1β-promoting source closely linked to aging and heightened cardiovascular risk, has rarely been investigated for its potential protective role in UAP.

On the basis of the UK Biobank (UKB) cohort, our study is the first to reveal the protective effect of CHIP against the development of AP. In brief, the associations between different CHIP statuses and the risk of distinctive AP types were discovered via covariate models and validated via propensity score matching (PSM) models. Mendelian randomization (MR) analyses were carried out to rule out confounding effects from horizontal pleiotropy and reverse causality. To explore the underlying mechanisms, mediation effects of cytokines and metabolites on the CHIP-AP associations were elucidated.

## Methods

### Study population

In this research, individuals between the ages of 40 and 70 years were enrolled at 22 different assessment centres across the UK from 2006 to 2010 [11]. The study was approved by the North West Multicentre Research Ethics Committee under UKB application number 105945, and all participants provided written informed consent.

Among the 469,686 UKB participants whose exome sequencing data were available, those with haematologic malignancies, congenital heart disease, or discrepancies between self-reported and genetic sex at baseline were excluded. Participants with incomplete covariate data were also excluded from the statistical analysis (Supplementary Fig. S1 **and** Supplementary Table S1). The ancestry categories were White British and other, according to both self-reported data and genetic ancestry analysis (Supplementary Methods).

### CHIP variant calling

To identify CHIP variants, the GATK Mutect2 somatic caller was used to detect somatic mutations from the whole exome sequencing of UKB participants [12], with gnomAD v4 as the reference for germline allele frequencies. A cohort-specific panel of normals was created by selecting 150 UKB samples from individuals aged 40 or younger without haematological malignancies. Initially, CHIP variants were limited to genes associated with haematological malignancies, based on the Catalogue Of Somatic Mutations In Cancer (COSMIC) database (http://cancer.sanger.ac.uk/cancergenome/projects/cosmic/). Quality control procedures and additional details are provided in the Supplementary Methods [13].

### Exposure

Participants with a variant allele fraction (VAF) less than 2 % for CHIP variants were classified as no CHIP status, whereas those with a VAF of 2 % or greater were identified as having any overall CHIP. A VAF of 10 % or greater was considered to indicate large overall CHIP. The three most common driver genes, *DNMT3A*, *TET2*, and *ASXL1*, were prominently represented in the gene-specific analysis (Supplementary Methods).

### Outcome

The focus of this study was on AP and its subtypes, UAP and SAP, identified through hospitalization records via the International Classification of Diseases, Ninth Revision (ICD-9) and Tenth Revision (ICD-10) codes. AP corresponded to the ICD-9 code 413 and the ICD-10 code I20. UAP was labelled I20.0, whereas SAP was labelled I20.8 in the ICD-10 (Supplementary Table S1).

### Pro-inflammatory and anti-inflammatory cytokines

Plasmic proteomic data from blood samples were obtained via the Olink Explore 1536 platform, which can be used to measure 1463 unique proteins (Supplementary Methods) [14]. To prevent batch effects, data from Instance 0 of the Olink platform, which involved 53,014 participants, were used. Common proinflammatory and anti-inflammatory cytokines were chosen for association and mediation analyses (Supplementary Table S2).

### Mendelian randomization analysis

A genome-wide association study (GWAS) of each CHIP status and the risk of developing AP was carried out with unrelated White British participants and adjusted for age, sex and 5 genetic principal components via Plink2. Mendelian randomization (MR) analysis was conducted with different CHIP statuses as exposures and each AP type as an outcome via the TwoSampleMR R package version 0.5.10. Details of MR and sensitivity analysis were in Supplementary Methods.

### Statistical analysis

Baseline characteristics were summarised by CHIP status and categorised by the CHIP gene and VAF thresholds. Categorical variables were reported as counts and percentages, whereas continuous variables were presented as the means with standard deviations (SD). Multivariable general linear models were constructed for assessing CHIP-AP associations, adjusting for sociodemographic characteristics, lifestyle factors, metabolic parameters, medication history, prevalent diseases, and family history of disease (Supplementary Methods). For validation, propensity score matching (PSM) was applied to match individuals with and without CHIP to balance confounding factors, with a 1:1, 1:2, 1:3, 1:4, or 1:5 ratio and using the MatchIt R package version 4.5.5 [15]. Restricted cubic spline (RCS) analysis was performed to examine the relationships between VAF and AP, UAP, and SAP, with identification of threshold values. To evaluate generalisability, associations were compared before and after adjustment for population stratification and replicated across White, Asian, and Black subgroups. Potential pathogenic mechanisms were investigated through analysis of CHIP associations with atherosclerotic cardiovascular disease (ASCVD), coronary artery disease (CAD), and myocardial infarction (MI).

To investigate potential mediators of the impacts of CHIP against the development of AP, we examined the associations between CHIP and cytokine levels. In light of 200 comparisons (8 exposures and 25 markers), a 2-sided P value threshold of < 0.00025 (i.e., P < 0.05/200) was applied to indicate statistical significance under multiple comparisons, while a P value of < 0.05 was used to denote nominal significance. Mediation analysis was also tested on UKB metabolomic data (details in Supplementary Methods). The mediating effects were analysed via the mediation R package version 4.5.0. All the statistical analyses were conducted in 2024 via R version 4.3.1 (Supplementary Methods).

## Results

### Baseline characteristics and CHIP frequency

A total of 452,285 participants from the UKB cohort were included. The mean age was 56.5 (SD = 8.1) years, with 246,608 (54.5 %) females and 205,677 (45.5 %) males. In terms of genetic ethnicity, 380,320 (84.1 %) were White British, and 71,965 (15.9 %) were from other genetic ancestries.

We identified 34,163 variants across 32,484 (7.2 %) participants. For gene-specific CHIP, we detected 12,854 variants in *DNMT3A*, 5225 in *TET2*, and 1093 in *ASXL1*. Variants were carried by 12,596 (2.8 %), 5150 (1.1 %), and 1089 (0.2 %) participants for these genes, respectively (Table 1 **and** Supplementary Tables S3-S5).

**Table 1:** Baseline characteristics of the study cohorts. Baseline characteristics were compared between participants of no CHIP and different CHIP statuses using *t*-test. Chi-squared or exact Fisher test was used for categorical variables. Abbreviations: SD, standard deviation; BMI, body mass index.

Characteristics	No CHIP (n = 419,801)	Any CHIP (n = 32,484)	P value	Large CHIP (n = 9899)	P value
Age, mean (SD), y	56.3 (8.10)	58.4 (7.72)	<0.001	58.8 (7.73)	<0.001
Sex			<0.001		0.027
Female	228,475 (54.4)	18,133 (55.8)		5499 (55.6)	
Male	191,326 (45.6)	14,351 (44.2)		4400 (44.4)	
Ancestry			0.028		<0.001
White	353,145 (84.1)	27,175 (83.7)		8116 (82.0)	
Other	66,656 (15.9)	5309 (16.3)		1783 (18.0)	
BMI, mean (SD) a	27.4 (4.77)	27.4 (4.73)	0.172	27.4 (4.69)	0.513
Alcohol intake frequency			<0.001		<0.001
Never	32,903 (7.84)	2661 (8.19)		843 (8.52)	
Special occasions only	47,869 (11.4)	3839 (11.8)		1194 (12.1)	
One to three times a month	46,951 (11.2)	3527 (10.9)		1068 (10.8)	
Once or twice a week	108,822 (25.9)	8144 (25.1)		2478 (25.0)	
Three or four times a week	97,848 (23.3)	7363 (22.7)		2182 (22.0)	
Daily or almost daily	85,408 (20.3)	6950 (21.4)		2134 (21.6)	
Smoking			<0.001		<0.001
Ever	250,541 (59.7)	19,766 (60.8)		6115 (61.8)	
Never	169,260 (40.3)	12,718 (39.2)		3784 (38.2)	
Townsend deprivation index, mean (SD)	−1.33 (3.07)	−1.36 (3.08)	0.093	−1.31 (3.11)	0.484
Diabetes	2974 (0.71)	248 (0.76)	0.271	78 (0.79)	0.384
Atherosclerotic heart disease	21,590 (5.14)	1860 (5.73)	<0.001	584 (5.90)	0.001
Atrial fibrillation and flutter	13,896 (3.31)	1157 (3.56)	0.016	364 (3.68)	0.047
Heart failure	4480 (1.07)	418 (1.29)	<0.001	147 (1.48)	<0.001
Chronic renal failure	1314 (0.31)	103 (0.32)	0.940	35 (0.35)	0.534
Colchicine use			0.855		0.348
Yes	203 (0.05)	17 (0.05)		7 (0.07)	
No	419,598 (99.95)	32,467 (99.95)		9892 (99.93)	

a Calculated as weight in kilograms divided by height in meters squared.

### Association of CHIP with AP

**Fig. 1:**
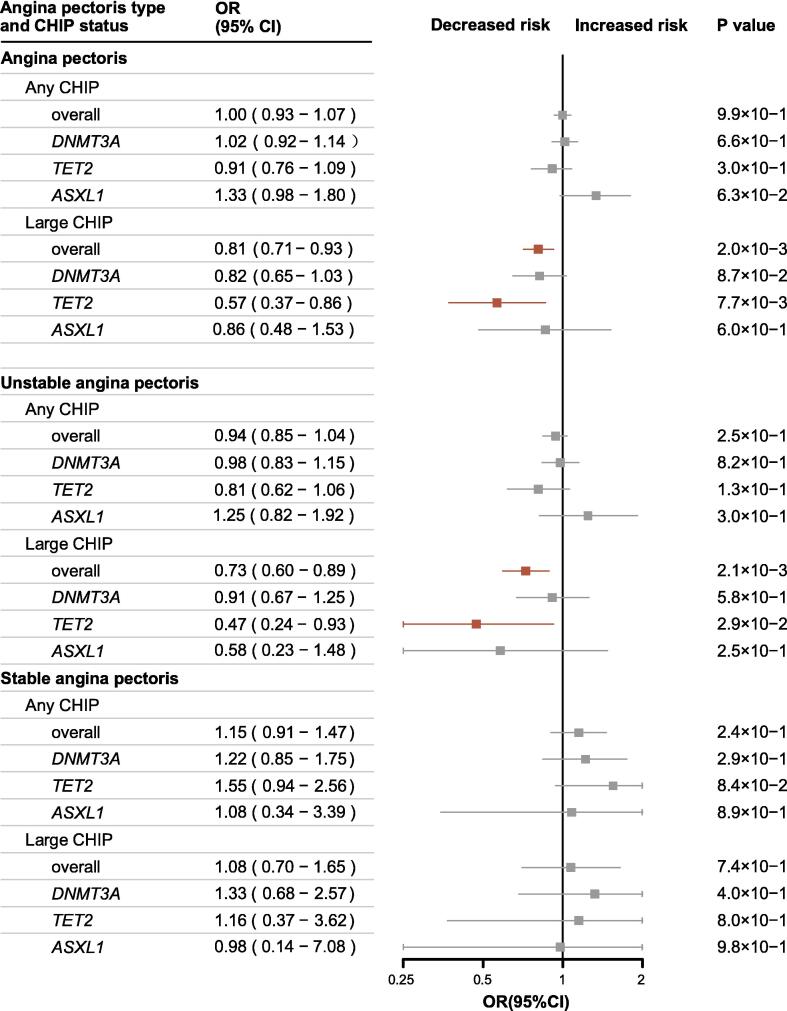
Association of Different CHIP Status and AP in Covariate Model 2. Effects of CHIP status on each AP type were presented as odds ratio (OR), 95% confidence interval (CI), and P value. Point estimate boxes and error bars in red indicated significant effects, while grey ones meant non-significant. The model 2 was adjusted for age, sex, ancestry, BMI, smoking status, alcohol intake, Townsend deprivation index, diabetes, atherosclerotic heart disease, hypertensive diseases, atrial fibrillation and flutter, stroke, heart failure, peripheral artery and capillary disease, chronic renal failure.

According to the adjusted models, large overall CHIP was associated with a significant reduction in AP risk (OR, 0.81; 95 % CI, 0.71–0.93; P = 0.002) and UAP risk (OR, 0.73; 95 % CI, 0.60–0.89; P = 0.002) compared with no CHIP status. Although an increased risk of developing SAP was observed, it was not statistically significant. Gene-specific analysis revealed that large *TET2* CHIP was protective against the development of AP (OR, 0.57; 95 % CI, 0.37–0.86; P = 0.008) and UAP (OR, 0.47; 95 % CI, 0.24–0.93; P = 0.03). No significant associations were found for other CHIP statuses (Fig. 1). The results demonstrated consistent patterns across multivariable models (Supplementary Fig. S2-S8). Large *TET2* CHIP exhibited consistent protective effects against UAP across White, Asian, and Black populations in covariate models (Supplementary Fig. S9-S11). Associations between CHIP and mortality of AP demonstrated similar trends (Supplementary Table S6). CHIP was significantly associated with elevated ASCVD and CAD risks, but non-significant associations were detected between CHIP and MI (Supplementary Fig. S12-S14).

**Fig. 2:**
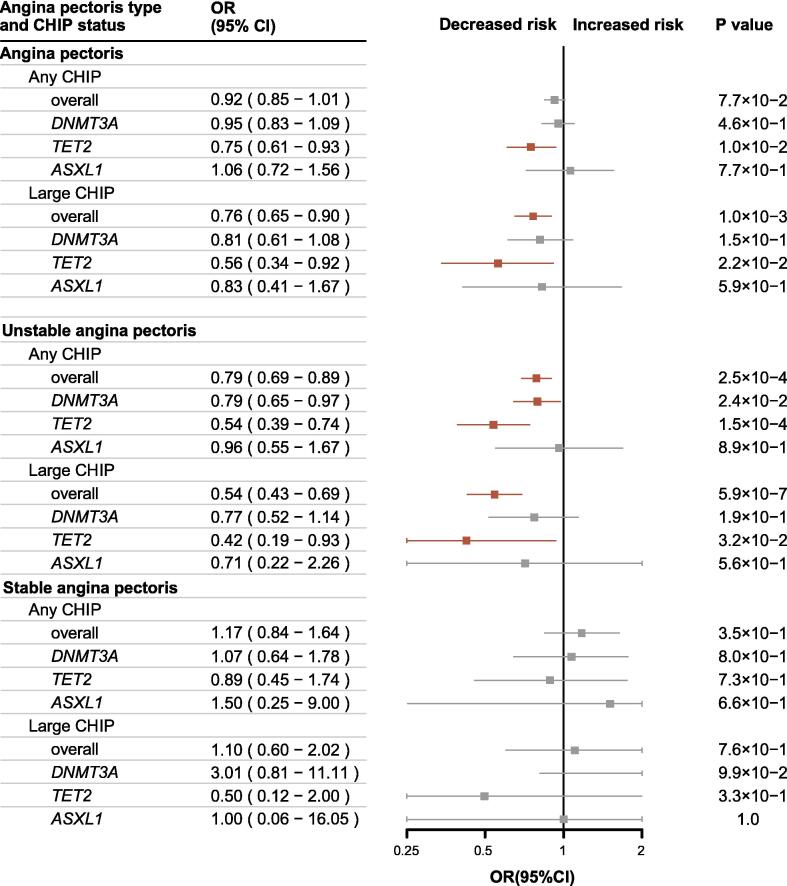
Association of Different CHIP Status and AP in PSM Models of 1:1 Ratio. Effects of CHIP status on each AP type were presented as OR, 95% CI, and P value. Point estimate boxes and error bars in red indicated significant effects, while grey ones meant non-significant. The model was adjusted by PSM, balancing age, sex, genetic ancestry, BMI, smoking status, alcohol intake status, Townsend deprivation index, diabetes, atherosclerotic heart disease, hypertensive diseases, atrial fibrillation and flutter, stroke, heart failure, peripheral artery and capillary disease, chronic renal failure.

According to the PSM models balancing all covariates (Supplementary Tables S7-S10), large overall CHIP was shown to have a significant protective effect against the development of AP (OR, 0.76; 95 % CI, 0.65–0.90; P = 0.001) and a stronger effect against the development of UAP (OR, 0.54; 95 % CI, 0.43–0.69; P < 0.0001). Any overall CHIP also significantly protected against the development of UAP (OR, 0.79; 95 % CI, 0.69–0.89; P = 0.00025). Gene-specific analysis confirmed the protective effects of large *TET2* CHIP for AP (OR, 0.56; 95 % CI, 0.34–0.92; P = 0.02) and UAP (OR, 0.42; 95 % CI, 0.19–0.93; P = 0.03). Protective effects were also detected for any *TET2* CHIP against the development of AP (OR, 0.75; 95 % CI, 0.61–0.93; P = 0.01) and for any *DNMT3A* CHIP (OR, 0.79; 95 % CI, 0.65–0.97; P = 0.02) and any *TET2* CHIP (OR, 0.54; 95 % CI, 0.39–0.74; P = 0.00015) against the development of UAP. Consistent with the results of the covariate models, CHIP had a detrimental but non-significant effect on the development of SAP (Fig. 2). Similar patterns were demonstrated using varying matching ratios (1:2, 1:3, 1:4, and 1:5) (Supplementary Fig. S15-S18). Large *TET2* CHIP exhibited consistent protective effects against UAP across White, Asian, and Black populations in PSM models (Supplementary Fig. S19-S21).

Analysis with a continuous VAF variable as the exposure showed that a higher overall VAF (OR, 0.43; 95 % CI, 0.23–0.84; P = 0.01) and *TET2* VAF (OR, 0.08; 95 % CI, 0.01–0.64; P = 0.02) were linked to a reduced AP risk. An increased overall VAF also lowered the risk of developing UAP (OR, 0.24; 95 % CI, 0.09–0.66; P = 0.005) (Supplementary Fig. S22). Subgroup analysis revealed a protective effect of CHIP, mainly in White British individuals, those with a history of smoking or high alcohol consumption, and those without various CVDs. Interestingly, CHIP was associated with a greater risk of developing SAP in some subgroups, suggesting distinctive effects for different angina types (Supplementary Tables S11-S16). The RCS curve showed that when the overall VAF exceeded 0.0923 and 0.0920, it exhibited significant protective effects against AP and UAP, respectively, while a *TET2* VAF greater than 0.130 and 0.122 conferred significant protection against AP and UAP, respectively. *DNMT3A* VAF exhibited significant protection against UAP when exceeding 0.0340, while turned non-significant above 0.0626. The VAF thresholds were consistent with the protective effects of large overall CHIP and large *TET2* CHIP against both AP and UAP (Supplementary Fig. S23).

### MR analysis interfering causal relationship between CHIP and AP

**Fig. 3:**
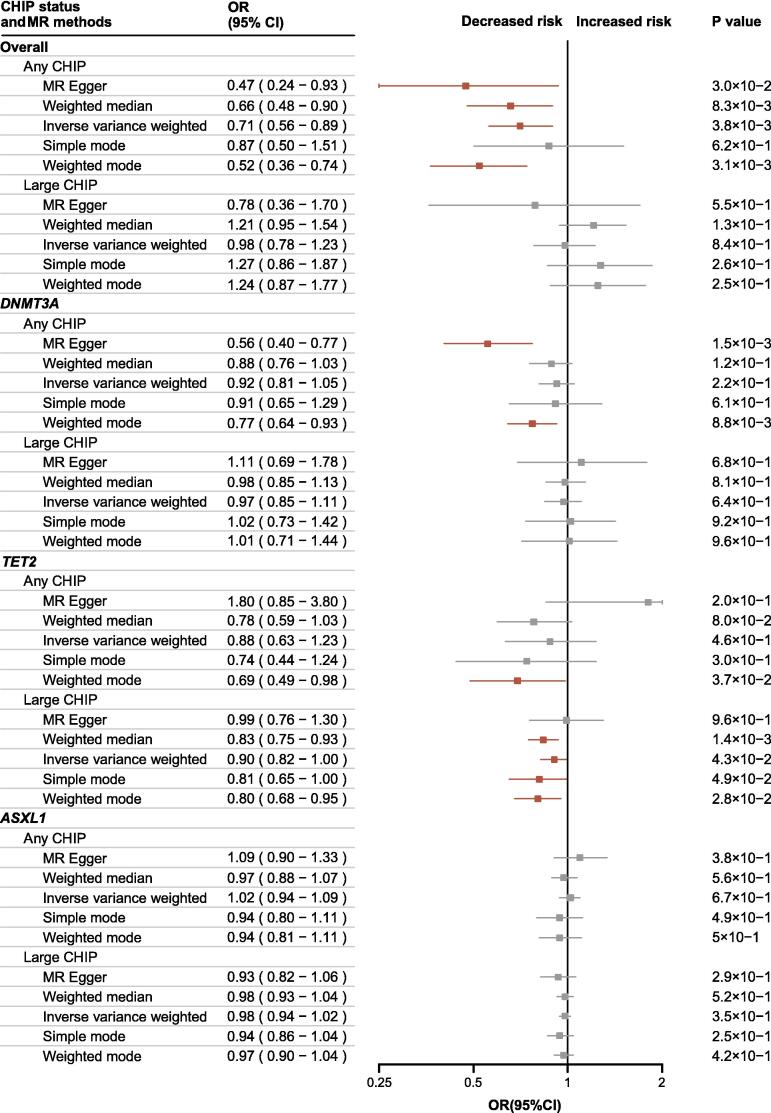
Mendelian Randomization Analysis for Causal Association of CHIP Status on UAP. Causal associations of different CHIP status with UAP were presented as OR, 95% CI, and P value. Point estimate boxes and error bars in red indicated significant effects, while grey ones meant non-significant.

MR analysis revealed that any overall CHIP had a protective effect against the development of AP (Weighted median: OR, 0.80; 95 % CI, 0.66–0.98; P = 0.03), with a more robust association with the risk of developing UAP (MR-Egger: OR, 0.47; 95 % CI, 0.24–0.93; P = 0.03. Weighted median: OR, 0.66; 95 % CI, 0.48–0.90; P = 0.008. IVW: OR, 0.71; 95 % CI, 0.56–0.89; P = 0.004. Weighted mode: OR, 0.52; 95 % CI, 0.36–0.74; P = 0.003) (Fig. 3**,** Supplementary Figs. S24, S30 and S31). Gene-specific analysis further confirmed that large *TET2* CHIP provided protection against the development of UAP (IVW: OR, 0.90; 95 % CI, 0.82–1.00; P = 0.04. Weighted median: OR, 0.83; 95 % CI, 0.75–0.93; P = 0.001. Weighted mode: OR, 0.80; 95 % CI, 0.68–0.95; P = 0.03), aligning with observational findings (Fig. 3 **and** Supplementary Figs. S25 and S31). Additionally, any *DNMT3A* CHIP was associated with the risk of developing AP (MR-Egger: OR, 0.76; 95 % CI, 0.61–0.95; P = 0.02) and UAP (MR-Egger: OR, 0.56; 95 % CI, 0.40–0.77; P = 0.002. Weighted mode: OR, 0.77; 95 % CI, 0.64–0.93; P = 0.009) (Fig. 3 **and** Supplementary Figs. S24, S30 and S31). Consistently, large *TET2* CHIP demonstrated a significant protective effect against the development of UAP across covariate, PSM, and MR models, highlighting its primary role in protection and the need to investigate possible mechanisms.

Sensitivity analysis confirmed the robustness of the instrumental variables, with a mean F statistic of 30.37 indicating strong instruments (Supplementary Tables S17 and S18). Horizontal pleiotropy was observed for the effects of any *DNMT3A* CHIP on the risk of developing AP (P = 0.039) and UAP (P = 0.003) but was adjusted via MR-Egger (Supplementary Table S19). Heterogeneity was detected (Supplementary Table S20). MR analysis excluding variants linked to known AP risk factors yielded consistent results (Supplementary Table S21 **and** Supplementary Figs. S27-S29). No significant reverse causality was found in the Steiger filtering test or reversal MR analysis (Supplementary Tables S22-S28).

### Association of CHIP with cytokine levels and mediation analysis

**Fig. 4:**
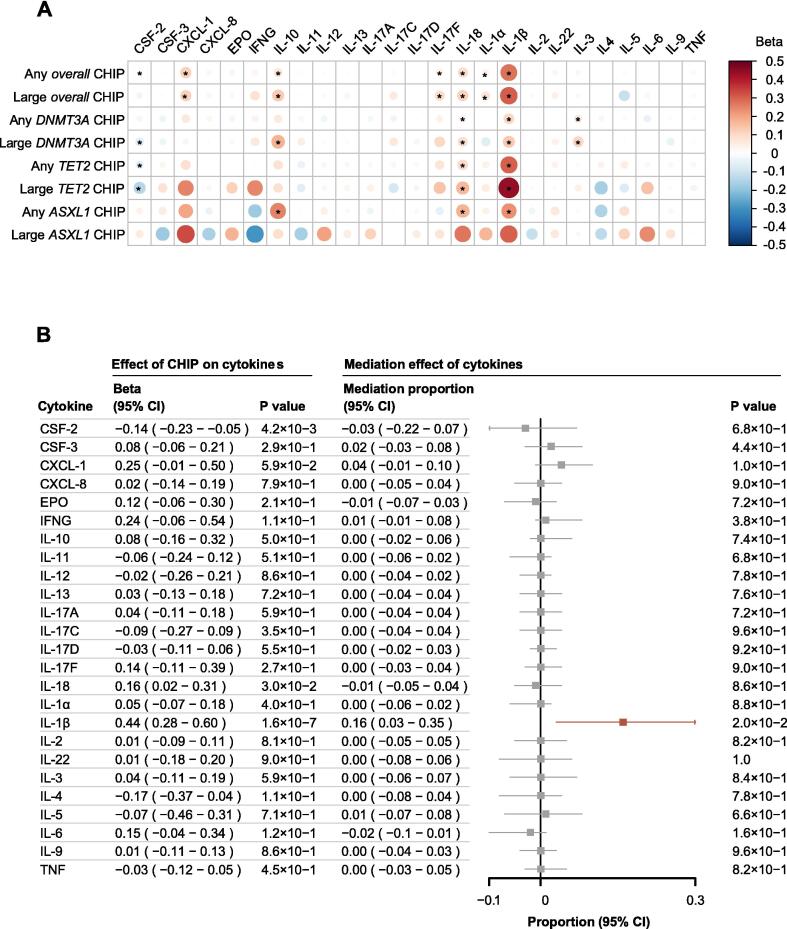
Association of CHIP with Cytokine Levels and Mediation Analyses on Cytokines in Model 1. (A) Associations of CHIP status with serum cytokine levels were detected in PSM models and shown in heat map. Each row presented one CHIP status, with CHIP gene and VAF threshold. Each column presented one cytokine. The size and color of each circle meant the association size and direction, respectively. The asterisk showed the nominal significant association (P < 0.05). (B) Mediation effects of cytokines in the association between large *TET2* CHIP and UAP were presented as mediation proportion, 95 % CI, and P value. Point estimate boxes and error bars in red indicated significant effects, while grey ones meant non-significant. Effects of large *TET2* CHIP on each cytokine level and corresponding significances were also shown along with the forest plot.

According to the covariate models, the serum protein levels of IL-1β, IL-10, IL-17D, IL-17F and IL-22 were increased in the CHIP population, whereas those of IL-1α, IL-5, IL-6, IL-11, IL-12, CXCL-8, CSF-2 and CSF-3 were decreased at the nominal significance level (P < 0.05). CSF-2 remained decreased at significance level adjusted for multiple comparisons (P < 0.00025) (Supplementary Table S29 **and** Supplementary Fig. S32). In the PSM models, IL-1β, IL-18, IL-1α, IL-3, IL-10, IL-17F and CXCL-1 were upregulated, whereas CSF-2 was downregulated at the nominal significance level. IL-1β, IL-18 and CXCL-1 remained increased at significance level adjusted for multiple comparisons (Fig. 4**A and** Supplementary Table S30). The direction of the associations remained consistent across both sets of models for IL-1β, IL-10, IL-17F, and CSF2. In particular, IL-1β showed consistent significant associations with all CHIP statuses except large *ASXL1* CHIP and exhibited the greatest increase in large *TET2* CHIP (beta, 0.44; 95 % CI, 0.28–0.60; P < 0.0001), highlighting its substantial impact (Fig. 4**A**).

To elucidate the underlying mechanisms of large *TET2* CHIP-related protection against the development of CVDs, the mediating effects of cytokine levels were assessed. IL-1β levels exhibited a significant mediating effect on the protection conferred by large *TET2* CHIP against both AP (proportion mediated, 0.16; 95 % CI, 0.03–0.33; P = 0.02) and UAP (proportion mediated, 0.16; 95 % CI, 0.03–0.35; P = 0.02) (Fig. 4**B and** Supplementary Fig. S35). IL-1β levels also had notable mediating effects on the protection provided by other CHIP statuses, including large overall CHIP (proportion mediated, 0.17; 95 % CI, 0.09–0.24; P < 0.0001) and any *TET2* CHIP (proportion mediated, 0.18; 95 % CI, 0.10–0.32; P < 0.0001) against the development of AP (Supplementary Figs. S33 and S34), and any overall CHIP (proportion mediated, 0.08; 95 % CI, 0.02–0.16; P < 0.0001), large overall CHIP (proportion mediated, 0.14; 95 % CI, 0.09–0.19; P < 0.0001), and any *TET2* CHIP (proportion mediated, 0.15; 95 % CI, 0.07–0.22; P < 0.0001) against the development of UAP (Supplementary Figs. S36-S39). No significant mediating effects were observed for the other cytokines. In sensitivity analysis, IL-1β demonstrated a consistently significant mediation effect on the association between large *TET2* CHIP and UAP across all multivariable models (Supplementary Figs. S40-S45).

### Association of CHIP with metabolites and mediation analysis

**Fig. 5:**
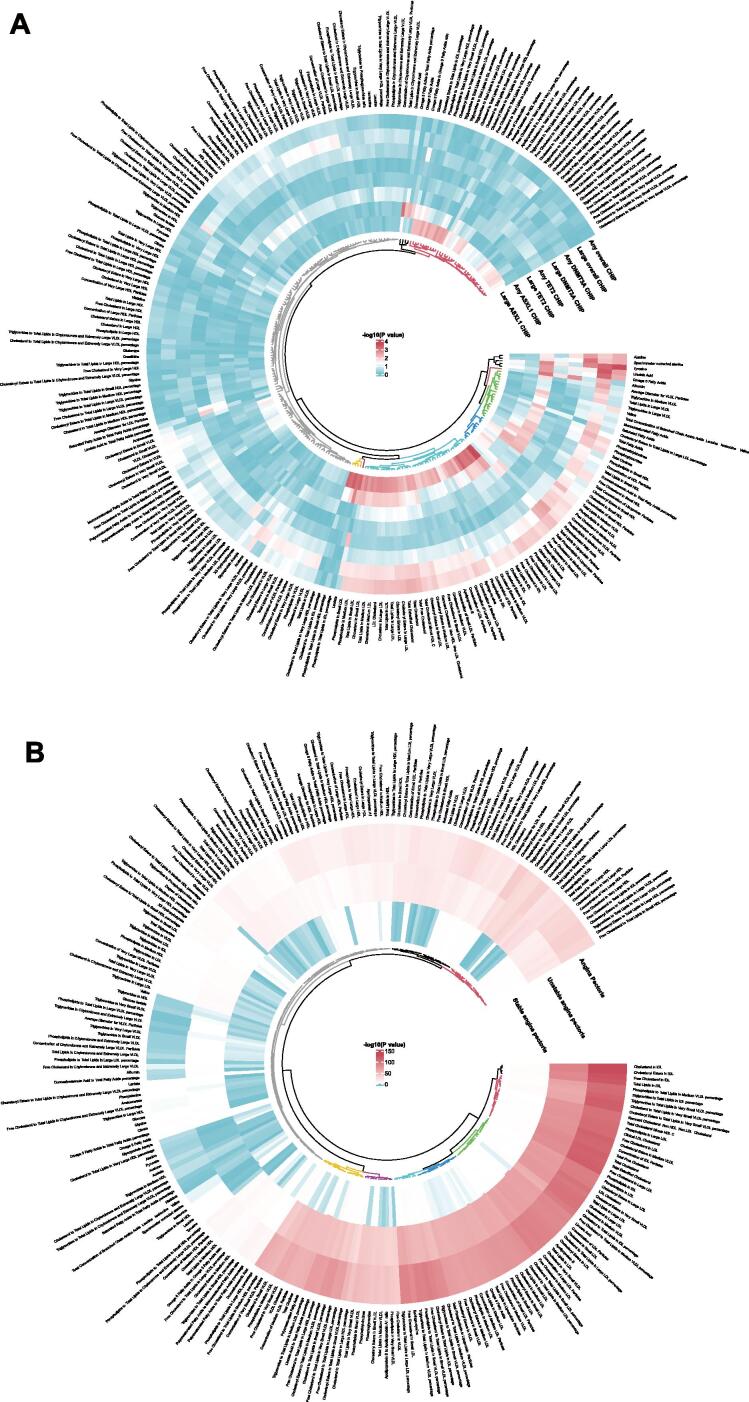
Heatmap of associations between CHIP, metabolites and AP. (A) Associations between CHIP status and metabolites. (B) Associations between metabolites and AP and its subtypes. Significant (red) and non-significant (blue) results are shown with colour intensity scaled to p-values, presented in clustered dendrograms.

Metabolomic data was analysed for assessing the roles of metabolites in CHIP-AP relationship. Totally 146 metabolites associated with both CHIP and AP or their respective subtypes, among which five metabolites exhibited significant mediation effects on the protective association between CHIP and AP or UAP (Fig. 5**,** Supplementary Tables S31-S32). However, interrogation of the metabolomic data revealed no consistent statistically significant mediation effects for these metabolites in multivariable models (Supplementary Fig. S46).

### Single-cell RNA sequencing analysis revealing effects of CHIP on atherosclerotic plaques

**Fig. 6:**
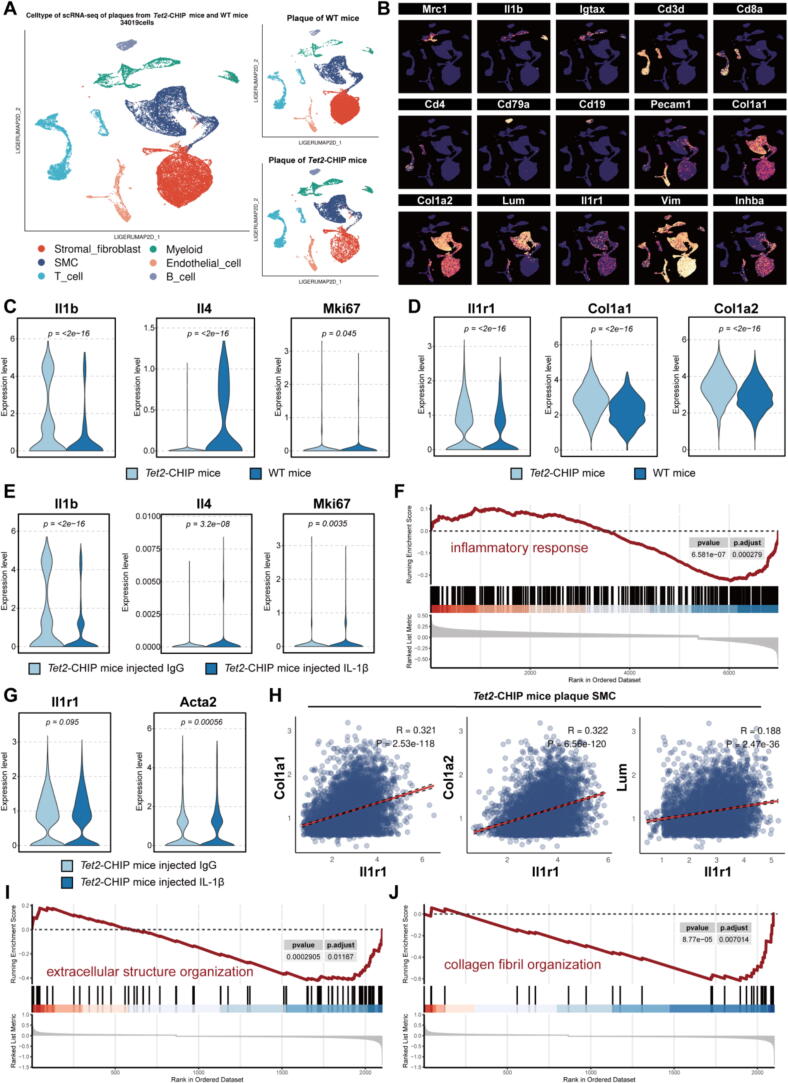
(A) ScRNA atlas of atherosclerotic plaques in *Tet2*-drived clonal haematopoiesis mice and control. (B) Major markers for cell subclusters in atherosclerotic plaques. (C) Violin plot of Il1b, Il4, and Mki67 levels in myeloid cells of atherosclerotic plaques from *Tet2*-drived clonal haematopoiesis mice and control. (D) Violin plot of Il1r1, Col1a1, and Col1a2 levels in SMC of atherosclerotic plaques from *Tet2*-drived clonal haematopoiesis mice and control. (E) Violin plot of Il1b, Il4, and Mki67 levels in myeloid cells of atherosclerotic plaques from *Tet2*-drived clonal haematopoiesis mice treated with Il1b antibodies and control. (F) GSEA analysis reveals that inflammatory response pathway was down-regulated in myeloid cells of atherosclerotic plaques from *Tet2*-drived clonal haematopoiesis mice after Il1b antibodies treatment. (G) Violin plot of Il1r1 and Acta2 levels in SMC of atherosclerotic plaques from *Tet2*-drived clonal haematopoiesis mice treated with Il1b antibodies and control. (H) Regression between Col1a1, Col1a2, Lum levels and expression of Il1r1 in SMC of atherosclerotic plaques from *Tet2*-drived clonal haematopoiesis mice treated with Il1b antibodies and control. (I) GSEA analysis reveals that extracellular structure organization pathway was down-regulated in SMC of atherosclerotic plaques from *Tet2*-drived clonal haematopoiesis mice after Il1b antibodies treatment. (J) GSEA analysis reveals that collagen fibril organization pathway was down-regulated in SMC of atherosclerotic plaques from *Tet2*-drived clonal haematopoiesis mice after Il1b antibodies treatment.

To further explore the effects of CHIP on atherosclerotic plaques, we analysed the single-cell RNA sequencing (scRNA-seq) dataset from atherosclerotic plaques of *Tet2* CHIP mice. Dimension reduction and cell-type annotation and clustering demonstrated a consistent increase in the proportion of SMCs and myeloid cells within atherosclerotic plaques of *Tet2* CHIP mice (Fig. 6**A and 6B**). Consistent with previous findings that myeloid cells in *Tet2* CHIP mice demonstrate increased proliferation and a pro-inflammatory phenotype characterized by elevated IL-1β expression (Fig. 6**C**), we additionally found that SMCs in *Tet2* CHIP mice showed upregulated expression of collagen-associated genes (Fig. 6**D**). Notably, IL-1β antibody neutralization in *Tet2* CHIP mice led to downregulation of αSMA expression in plaque SMCs (Fig. 6**G**), along with significant positive correlations between IL1 receptor levels and the expression of collagen, matrix, and fibre-related proteins (Fig. 6**H**). Furthermore, pathway enrichment analysis revealed that IL-1β neutralization attenuated extracellular matrix (ECM) deposition and collagen fibre formation in *Tet2* CHIP SMCs (Fig. 6**I and J**).

## Discussion

In our study, the presence of CHIP was independently associated with a reduced risk of AP, especially the UAP subtype. Gene-specific analysis indicated that this protective effect was primarily observed in large *TET2* CHIP and was mainly mediated by elevated serum IL-1β levels.

CHIP has been reported to elevate risk of age-related CVDs, including atherosclerotic heart disease and chronic ischemic heart failure [16,17]. However, exceptions emerge with the further and comprehensive understanding of its nature. Newly published study suggested that the increase risks of CHIP to CVDs or other age-related conditions were affected by covariates, instead of itself only [18,19]. Additionally, CHIP was also demonstrated to serve as a protective factor against certain conditions including Alzheimer’s disease, attributed to the enhanced phagocytic activity of microglia harbouring the mutation [20]. Observed diversity highlights the importance of analysing CHIP effects according to specific conditions and distinct pathophysiological mechanisms.

Increasing inflammation level, mainly contributed by IL-1β, was recognized as the key intermediary between CHIP and CVDs risk [5,7,8,16,17,21,22]. From a mechanistic perspective, CHIP, especially *TET2* CHIP may contribute to inflammatory disorders through the NLRP3 inflammasome and subsequent IL-1β secretion, which could increase risks of CVDs. Anti-inflammatory treatment, especially targeting IL-1β, is gaining increasing attention since the inflammation hypothesis was revived and gained widespread acceptance in the early 1990 s, when evidence linking chronic inflammation to atherosclerosis progression became irrefutable [23]. However, its clinical effects have been proved to be more complex than idealised expectations from latest trials. Applying anakinra, a recombinant IL-1 receptor antagonist (IL-1ra), increased major adverse cardiovascular events, including myocardial infarction, at one year by greater than fourfold in MRC-ILA Heart Study [24]. Moreover, low-dose colchicine did not demonstrate a significant effect on reducing cardiovascular events in the prevention of recurrent strokes in acute cerebrovascular disease in the CHANCE-3 trial [25]. These findings suggested that IL-1 signal pathway plays dialectical roles in different stages or types of CVDs. Indiscriminate pharmacological blockade without precise stratification of disease subtypes, optimal therapeutic timing, or dose titration may yield suboptimal clinical outcomes.

In the development of atherosclerotic diseases, IL-1β is recognized contributing to the acceleration of inflammation and plaque thickening [7,8]. As a direct clinical manifestation of plaque thickening, SAP demonstrated positive association with CHIP in our study, although not significant. CHIP was further observed elevating risks of ASCVD and CAD, supporting its involvement in atherosclerotic progression [26,27]. Nevertheless, the association between CHIP and MI was not statistically significant, consistent with previous studies [28,29]. Though MI represents severe end-stage manifestation of coronary atherosclerosis, CHIP appears to exert inconsistent effects in these conditions. This intriguing discrepancy may be attributed to fundamental differences between CAD versus MI or UAP, particularly regarding plaque stability versus rupture.

Except for the effects in driving plaque formation, IL-1β has also been shown to stabilize advanced atherosclerotic lesions [9,10], thus might help protecting against UAP caused by plaque rupture and thrombosis [4,30,31]. Through multifaceted approaches combining SMC lineage tracing, scRNA-seq, high-resolution tissue imaging, and cell-specific gene knockout, two studies by Gomez et al. and Karnewar et al. consistently demonstrated that IL-1β stabilize plaque by promoting extracellular matrix synthesis via IL-1R1 signalling in SMCs, thereby facilitating the formation and maintenance of an SMC- and collagen-rich fibrous cap [9,10]. Building upon these findings, SMC lineage tracing in anti-IL-1β monoclonal antibody-treated *Apoe*−/− mice revealed a reduction in contractile SMCs accompanied by osteochondrocyte-like cell expansion [10]. Our scRNA-seq analysis of *Tet2* CHIP mice revealed concurrent expansion of SMCs and myeloid cells in atherosclerotic plaques. IL-1β neutralization downregulated αSMA in plaque SMCs and attenuated ECM/collagen production, which correlated strongly with IL1 receptor levels. These observations implicate SMCs and immune cell subsets dynamics as potential mediators of IL-1β-mediated plaque stabilization. The metabolome-based mediation analysis did not identify metabolites with stable mediating effects, highlighting the critical need for stratified, condition-specific metabolomic profiling to elucidate context-dependent metabolic regulatory networks.

MI patients were observed to exhibit higher plaque burden than UAP [32], which could be exacerbated by *TET2* CHIP through IL-1β secretion [26]. Although CHIP may reduce UAP risk by stabilizing plaques, its concurrent effect on promoting plaque growth may lead to inconclusive net effects on MI, without demonstrating definitive protective or detrimental tendencies. The CANTOS trial demonstrated that canakinumab, an IL-1β monoclonal antibody, reduced cardiovascular events and mortality post-MI. However, clinical benefit was only observed at intermediate doses, and patients failing to achieve significant IL-6 reduction showed outcomes comparable to placebo group [33]. These findings collectively indicate that anti-inflammatory therapeutic efficacy depends on multiple determinants, including treatment dosage, cytokine specificity, atherosclerotic stage, CHIP status, systemic inflammatory status, and comprehensive cytokine signatures. While we fundamentally support the inflammation hypothesis of atherosclerosis and acknowledge the therapeutic value of anti-inflammatory strategies [23], future clinical studies should incorporate multifactorial patient assessments and advanced analytical approaches to optimize personalized anti-inflammatory interventions for cardiovascular risk management.

Our study highlighted protective role of CHIP against AP using covariate, PSM, and MR analyses, which addressed confounding and reverse causality. Integrated mediation and scRNA-seq analyses explored potential mechanisms. However, several limitations should be considered when interpreting our findings. First, the UKB cohort is predominantly composed of White British individuals. Although we conducted subgroup analyses on Asian and Black populations within the UKB, further investigations should be extended to cohorts and regions with distinct major population groups, including Arab and Latino populations, to enhance the robustness and generalisability of the findings. Second, AP indicates acute coronary changes but poses challenges in imaging plaque rupture and stability, warranting future research to enhance plaque rupture detection. Third, given that CHIP is characterized by the proliferation of haematopoietic cells and cytokine dysregulation, while other potential mediating factors, including metabolome and immune cell subsets, warrant more systematic and comprehensive examination. Fourth, CHIP drivers harbouring fewer mutation sites require comprehensive investigation through large-scale clinical cohorts coupled with mechanistic studies.

## Conclusions

The results from our study showed that CHIP, especially large *TET2* CHIP, offered protection against the development of AP, with a notable protective effect against the development of UAP. Elevated IL-1β levels may mediate this protection by stabilizing atherosclerotic plaques. Further research is needed to explore the detailed mechanisms underlying this protective effect to better understand the roles of CHIP in CVDs and yield actionable insights for tailored anti-inflammatory strategies in cardiovascular risk management.

## Data sharing statement

Data generated and analysed in this study are available from corresponding author upon request except for UKB data, which only available directly on application.

## Compliance with ethics requirements

The study was approved by the North West Multicentre Research Ethics Committee under UKB application number 105945, and all participants provided written informed consent.

## Declaration of competing interest

The authors declare that they have no known competing financial interests or personal relationships that could have appeared to influence the work reported in this paper.

## References

[1] Joshi P.H., de Lemos J.A. (2021). Diagnosis and management of stable angina: a review. J Am Med Assoc.

[2] Collet J.P., Thiele H., Barbato E., Barthélémy O., Bauersachs J., Bhatt D.L. (2021). 2020 ESC guidelines for the management of acute coronary syndromes in patients presenting without persistent ST-segment elevation. Eur Heart J.

[3] Libby P., Pasterkamp G., Crea F., Jang I.K. (2019). Reassessing the mechanisms of acute coronary syndromes. Circ Res.

[4] Sechtem U., Brown D., Godo S., Lanza G.A., Shimokawa H., Sidik N. (2020). Coronary microvascular dysfunction in stable ischaemic heart disease (non-obstructive coronary artery disease and obstructive coronary artery disease). Cardiovasc Res.

[5] Evans M.A., Walsh K. (2023). Clonal hematopoiesis, somatic mosaicism, and age-associated disease. Physiol Rev.

[6] Vlasschaert C., Mack T., Heimlich J.B., Niroula A., Uddin M.M., Weinstock J. (2023). A practical approach to curate clonal hematopoiesis of indeterminate potential in human genetic data sets. Blood.

[7] Paramel Varghese G., Folkersen L., Strawbridge R.J., Halvorsen B., Yndestad A., Ranheim T. (2016). NLRP3 inflammasome expression and activation in human atherosclerosis. J Am Heart Assoc.

[8] Sharma B.R., Kanneganti T.D. (2021). NLRP3 inflammasome in cancer and metabolic diseases. Nat Immunol.

[9] Gomez D., Baylis R.A., Durgin B.G., Newman A.A.C., Alencar G.F., Mahan S. (2018). Interleukin-1β has atheroprotective effects in advanced atherosclerotic lesions of mice. Nat Med.

[10] Karnewar S., Karnewar V., Deaton R.A., Shankman L.S., Benavente E.D., Williams C.M. (2024). IL-1β inhibition partially negates the beneficial effects of diet-induced atherosclerosis regression in mice. Arterioscler Thromb Vasc Biol.

[11] Backman J.D., Li A.H., Marcketta A., Sun D., Mbatchou J., Kessler M.D. (2021). Exome sequencing and analysis of 454,787 UK Biobank participants. Nature.

[12] Benjamin D, Sato T, Cibulskis K, Getz G, Stewart C, Lichtenstein L. Calling Somatic SNVs and Indels with Mutect2. bioRxiv. 2019:861054.

[13] Wong W.J., Emdin C., Bick A.G., Zekavat S.M., Niroula A., Pirruccello J.P. (2023). Clonal haematopoiesis and risk of chronic liver disease. Nature.

[14] Sun B.B., Chiou J., Traylor M., Benner C., Hsu Y.H., Richardson T.G. (2023). Plasma proteomic associations with genetics and health in the UK Biobank. Nature.

[15] Kurz C.F., Krzywinski M., Altman N. (2024). Propensity score matching. Nat Methods.

[16] Gumuser E.D., Schuermans A., Cho S.M.J., Sporn Z.A., Uddin M.M., Paruchuri K. (2023). Clonal hematopoiesis of indeterminate potential predicts adverse outcomes in patients with atherosclerotic cardiovascular disease. J Am Coll Cardiol.

[17] Dorsheimer L., Assmus B., Rasper T., Ortmann C.A., Ecke A., Abou-El-Ardat K. (2019). Association of mutations contributing to clonal hematopoiesis with prognosis in chronic ischemic heart failure. JAMA Cardiol.

[18] Stacey S.N., Zink F., Halldorsson G.H., Stefansdottir L., Gudjonsson S.A., Einarsson G. (2023). Genetics and epidemiology of mutational barcode-defined clonal hematopoiesis. Nat Genet.

[19] Is clonal hematopoiesis 'mostly harmless'? Nat Genet 2023;55(12):2029-30. 10.1038/s41588-023-01556-y 37996619

[20] Bouzid H., Belk J.A., Jan M., Qi Y., Sarnowski C., Wirth S. (2023). Clonal hematopoiesis is associated with protection from Alzheimer's disease. Nat Med.

[21] Steensma D.P., Bejar R., Jaiswal S., Lindsley R.C., Sekeres M.A., Hasserjian R.P. (2015). Clonal hematopoiesis of indeterminate potential and its distinction from myelodysplastic syndromes. Blood.

[22] Boettcher S., Ebert B.L. (2019). Clonal hematopoiesis of indeterminate potential. J Clin Oncol.

[23] Grebe A., Hoss F., Latz E. (2018). NLRP3 inflammasome and the IL-1 pathway in atherosclerosis. Circ Res.

[24] Morton A.C., Rothman A.M., Greenwood J.P., Gunn J., Chase A., Clarke B. (2015). The effect of interleukin-1 receptor antagonist therapy on markers of inflammation in non-ST elevation acute coronary syndromes: the MRC-ILA Heart Study. Eur Heart J.

[25] Li J., Meng X., Shi F.D., Jing J., Gu H.Q., Jin A. (2024). Colchicine in patients with acute ischaemic stroke or transient ischaemic attack (CHANCE-3): multicentre, double blind, randomised, placebo controlled trial. BMJ (Clin Res Ed).

[26] Fuster J.J., MacLauchlan S., Zuriaga M.A., Polackal M.N., Ostriker A.C., Chakraborty R. (2017). Clonal hematopoiesis associated with TET2 deficiency accelerates atherosclerosis development in mice. Science (New York, NY).

[27] Zekavat S.M., Viana-Huete V., Matesanz N., Jorshery S.D., Zuriaga M.A., Uddin M.M. (2023). TP53-mediated clonal hematopoiesis confers increased risk for incident atherosclerotic disease. Nat Cardiovasc Res.

[28] Fawaz S., Marti S., Dufossee M., Pucheu Y., Gaufroy A., Broitman J. (2024). Evaluation of clonal hematopoiesis and mosaic loss of Y chromosome in cardiovascular risk: an analysis in prospective studies. Elife.

[29] Jaiswal S., Natarajan P., Silver A.J., Gibson C.J., Bick A.G., Shvartz E. (2017). Clonal hematopoiesis and risk of atherosclerotic cardiovascular disease. N Engl J Med.

[30] Levy B.I., Heusch G., Camici P.G. (2019). The many faces of myocardial ischaemia and angina. Cardiovasc Res.

[31] Bentzon J.F., Otsuka F., Virmani R., Falk E. (2014). Mechanisms of plaque formation and rupture. Circ Res.

[32] Jang I.K., Tearney G.J., MacNeill B., Takano M., Moselewski F., Iftima N. (2005). In vivo characterization of coronary atherosclerotic plaque by use of optical coherence tomography. Circulation.

[33] Ridker P.M., Everett B.M., Thuren T., MacFadyen J.G., Chang W.H., Ballantyne C. (2017). Antiinflammatory therapy with canakinumab for atherosclerotic disease. N Engl J Med.

