# Supplementary material for: Clonal haematopoiesis is associated with protection against angina pectoris in the UK population
Source: J Adv Res. 2025 Aug 5;83:647–58. doi: 10.1016/j.jare.2025.08.002 (PMC13131456; doi:10.1016/j.jare.2025.08.002)
Supplement: Supplementary Data 1 [file mmc1.pdf]

Supplementary Materials

Supplementary Methods 4
CHIP variant calling..4
Covariates..4
Analysis of continuous association between VAF and AP..5
Subgroup analysis..5
Genome-wide association study..5
Mendelian randomization analysis..6
Proteomic measurement, processing and quality control..6
Metabolomic processing and mediation analysis..6
Single cell sequencing data acquisition and processing..6
References..8
Supplementary Figures..9
Supplementary Figure S1: Study design..9
Supplementary Figure S2: Association of different CHIP status and AP in covariate model 1 10
Supplementary Figure S3: Association of different CHIP status and AP in covariate model 3 11
Supplementary Figure S4: Association of different CHIP status and AP in covariate model 4 12
Supplementary Figure S5: Association of different CHIP status and AP in covariate model 5 13
Supplementary Figure S6: Association of different CHIP status and AP in covariate model 6 14
Supplementary Figure S7: Association of different CHIP status and AP in covariate model 7 15
Supplementary Figure S8: Association of different CHIP status and AP in covariate model 7 not adjusting for population confounders..16
Supplementary Figure S9: Association of different CHIP status and AP in covariate model 7 in White population..17
Supplementary Figure S10: Association of different CHIP status and AP in covariate model 7 in Asian population..18
Supplementary Figure S11: Association of different CHIP status and AP in covariate model 7 in Black population..19
Supplementary Figure S12: Association of different CHIP status and ASCVD and CAD in covariate model 7..20
Supplementary Figure S13: Association of different CHIP status and MI in covariate model 7 21
Supplementary Figure S14: Association of JAK2 CHIP and ASCVD and MI in covariate model 7..22
Supplementary Figure S15: Association of different CHIP status and AP in PSM models of 1:2 ratio..23
Supplementary Figure S16: Association of different CHIP status and AP in PSM models of 1:3 ratio..24
Supplementary Figure S17: Association of different CHIP status and AP in PSM models of 1:4 ratio..25
Supplementary Figure S18: Association of different CHIP status and AP in PSM models of 1:5 ratio..26
Supplementary Figure S19: Association of different CHIP status and AP in PSM models of 1:1 ratio in White population..27
Supplementary Figure S20: Association of different CHIP status and AP in PSM models of 1:1 ratio in Asian population..28
Supplementary Figure S21: Association of different CHIP status and AP in PSM models of 1:1 ratio in Black population..29
Supplementary Figure S22: Association between VAF and AP based on the model fully adjusted for covariates..30
Supplementary Figure S23: RCS curve for associations between VAF and AP..31

Supplementary Figure S24: Forest plot of Mendelian randomization analysis for causal association of CHIP on AP..32
Supplementary Figure S25: Forest plot of Mendelian randomization analysis of more methods for causal association of large *TET2* CHIP on UAP 33
Supplementary Figure S26: Forest plot of Mendelian randomization analysis for causal association of CHIP on SAP 34
Supplementary Figure S27: Forest plot of Mendelian randomization analysis for causal association of CHIP on AP after excluding IVs relevant to AP risk factors............................35
Supplementary Figure S28: Forest plot of Mendelian randomization analysis for causal association of CHIP on UAP after excluding IVs relevant to AP risk factors.........................36
Supplementary Figure S29: Forest plot of Mendelian randomization analysis for causal association of CHIP on SAP after excluding IVs relevant to AP risk factors 37
Supplementary Figure S30: Scatter plots of Mendelian randomization analysis for causal association of CHIP on AP..38
Supplementary Figure S31: Scatter plots of Mendelian randomization analysis for causal association of CHIP on UAP..39
Supplementary Figure S32: Heatmap of association between CHIP and cytokines based on the model fully adjusted for covariate...40
Supplementary Figure S33: Forest plot of mediation analysis for mediation effect of cytokines between large overall CHIP and AP in model 1 41
Supplementary Figure S34: Forest plot of mediation analysis for mediation effect of cytokines between any *TET2* CHIP and AP in model 1 42
Supplementary Figure S35: Forest plot of mediation analysis for mediation effect of cytokines between large *TET2* CHIP and AP in model 1 43
Supplementary Figure S36: Forest plot of mediation analysis for mediation effect of cytokines between any overall CHIP and UAP in model 1 44
Supplementary Figure S37: Forest plot of mediation analysis for mediation effect of cytokines between large overall CHIP and UAP in model 1 45
Supplementary Figure S38: Forest plot of mediation analysis for mediation effect of cytokines between any *DNMT3A* CHIP and UAP in model 1..46
Supplementary Figure S39: Forest plot of mediation analysis for mediation effect of cytokines between any *TET2* CHIP and UAP in model 1 47
Supplementary Figure S40: Forest plot of mediation analysis for mediation effect of cytokines between large *TET2* CHIP and UAP in model 2 48
Supplementary Figure S41: Forest plot of mediation analysis for mediation effect of cytokines between large *TET2* CHIP and UAP in model 3 49
Supplementary Figure S42: Forest plot of mediation analysis for mediation effect of cytokines between large *TET2* CHIP and UAP in model 4 50
Supplementary Figure S43: Forest plot of mediation analysis for mediation effect of cytokines between large *TET2* CHIP and UAP in model 5 51
Supplementary Figure S44: Forest plot of mediation analysis for mediation effect of cytokines between large *TET2* CHIP and UAP in model 6 52
Supplementary Figure S45: Forest plot of mediation analysis for mediation effect of cytokines between large *TET2* CHIP and UAP in model 7 53
Supplementary Figure S46: Forest plot of mediation analysis for mediation effect of candidate metabolites 54
Supplementary Tables 55
Supplementary Table S1: Phenotyping identification in UKB cohort...................................55
Supplementary Table S2: Olink0 proteins used in association and mediation analysis 56
Supplementary Table S3: Baseline characteristics of the *DNMT3A* CHIP cohorts in association analysis of covariate models 57
Supplementary Table S4: Baseline characteristics of the *TET2* CHIP cohorts in association analysis of covariate models 59
Supplementary Table S5: Baseline characteristics of the *ASXL1* CHIP cohorts in association analysis of covariate models 61
Supplementary Table S6: Association between CHIP and mortality of AP based on the model partially adjusted for covariates 63

Supplementary Table S7: Baseline characteristics of the overall CHIP cohorts in association analysis of PSM models 64
Supplementary Table S8: Baseline characteristics of the *DNMT3A* CHIP cohorts in association analysis of PSM models 66
Supplementary Table S9: Baseline characteristics of the *TET2* CHIP cohorts in association analysis of PSM models 68
Supplementary Table S10: Baseline characteristics of the *ASXL1* CHIP cohorts in association analysis of PSM models 70
Supplementary Table S11: Stratified odds ratios of any overall CHIP associated with different types of AP 72
Supplementary Table S12: Stratified odds ratios of large overall CHIP associated with different types of AP 76
Supplementary Table S13: Stratified odds ratios of any *DNMT3A* CHIP associated with different types of AP 80
Supplementary Table S14: Stratified odds ratios of large *DNMT3A* CHIP associated with different types of AP 84
Supplementary Table S15: Stratified odds ratios of any *TET2* CHIP associated with different types of AP 88
Supplementary Table S16: Stratified odds ratios of large *TET2* CHIP associated with different types of AP 92
Supplementary Table S17: Instrumental variables used in Mendelian randomization analysis for causal association of any CHIP on each AP type 96
Supplementary Table S18: Instrumental variables used in Mendelian randomization analysis for causal association of large CHIP on each AP type 99
Supplementary Table S19: Pleiotropy analysis for causal association of CHIP on each AP type 102
Supplementary Table S20: Heterogeneity analysis for causal association of CHIP on each AP type 104
Supplementary Table S21: Instrumental variables associated with other phenotypes 107
Supplementary Table S22: Steiger directional test for causal association of CHIP on each AP type 109
Supplementary Table S23: Reverse Mendelian randomization analysis for causal association of AP on CHIP 111
Supplementary Table S24: Reverse Mendelian randomization analysis for causal association of UAP on CHIP 113
Supplementary Table S25: Reverse Mendelian randomization analysis for causal association of SAP on CHIP 115
Supplementary Table S26: Instrumental variables used in MR analysis for causal association of each AP type on CHIP 117
Supplementary Table S27: Pleiotropy analysis for causal association of each AP type on CHIP 120
Supplementary Table S28: Heterogeneity analysis for causal association of each AP type on CHIP 122
Supplementary Table S29: Association analysis between CHIP and cytokines using covariate models 125
Supplementary Table S30: Association analysis between CHIP and cytokines using PSM models 134
Supplementary Table S31: Metabolites significantly associated with CHIP status 143
Supplementary Table S32: Metabolites significantly associated with AP and its subtypes 156

Supplementary Methods

CHIP variant calling

The sequencing protocols involve capturing exomes with the IDT xGen Exome Research Panel v1.0, targeting 39 Mbp of the human genome (19,396 genes). Samples were multiplexed and sequenced with dual-indexed 75x75 bp paired-end reads on the Illumina NovaSeq 6000 platform, using S2 flow cells for the initial 50k release and S4 flow cells for later samples. Different IDT v1.0 oligo lots were used for the initial and subsequent samples; this should be included as a covariate in downstream analysis. Coverage exceeds 20X at 95.2% of targeted bases on average. The updated Functional Equivalence (OQFE) protocol was used for analysis, retaining original quality scores in aligned sequencing (CRAM) files. This protocol aligns and duplicates all raw sequencing data (FASTQ) to the GRCh38 reference build, producing CRAM files for CHIP calling. (1)

Somatic mutation detection for CHIP identification was performed using GATK Mutect2 on UK Biobank whole exome sequencing data. Germline variant filtering employed gnomAD v4 allele frequencies as reference. To improve specificity, we generated a panel of normals comprising 150 UKB samples from healthy donors aged $\leq$40 years without haematologic malignancies. Variant calling was initially restricted to haematologic malignancy-associated genes curated from the COSMIC database (http://cancer.sanger.ac.uk/cancergenome/projects/cosmic/). (2-4) For quality control, only variants with a total depth $\geq$ 10, alternate allele depth (AD) $\geq$ 2, and F1R2 and F2R1 read pair depth $\geq$ 1 were kept. Multi-allelic variants, variants flagged as part of the panel of normals (unless previously reported), and indels flagged by the position filter were excluded. Variants in homo-polymer repeats were filtered out unless AD $\geq$ 10 or VAF $\geq$ 0.08. Missense mutations in CBL or *TET2* inconsistent with somatic origin (P value $>$ 0.001 in a binomial test of VAF = 0.5) were removed. For population-level QC, novel variants with a median VAF $>$ 0.35 were removed due to their likelihood of being germline variants or sequencing errors.(5) Additionally, novel variants with a maximum AD $<$ 6 across all samples and fewer than 2 supportive reads from F1R2 (C>A) or F2R1 (G>T) mate pairs were excluded to account for oxidation artifacts. CHIP variants were further restricted to genes accounting for 90% of mutations. Variant allele fraction (VAF) was calculated as AD/(reference allele depth (RD) + AD).

Covariates

Codes to define variables for covariate and PSM adjustment were as follows: age at recruitment (Field ID: 21022), sex (Field ID: 31), genetic ancestry (Field ID: 22006), Townsend deprivation index (Field ID: 22189), assessment centre (Field ID: 54), education level (Field ID: 6138), sleep duration (Field ID: 1160), physical activity (Field ID:), diet (Category ID: 100052), ever smoked (Field ID: 20160), alcohol intake frequency (Field ID: 1558), body-mass index (BMI, Field ID: 21001), waist circumference (Field ID: 48), systolic blood pressure (Field ID: 4080), diastolic blood pressure (Field ID: 4079), total cholesterol (Field ID: 30690), low-density lipoprotein cholesterol (Field ID: 30780), high-density lipoprotein cholesterol (Field ID: 30760), triglycerides (Field ID: 30870), glucose (Field ID: 30740), glycated haemoglobin (HbA1c, Field ID: 30750), estimated glomerular filtration rate (eGFR, calculated based on creatinine, Field ID: 30700), lipid lowering medication (Field ID: 6153), anti-hypertensive medication (Field ID: 6153), antidiabetic medication (Field ID: 6153), diabetes (main ICD9: 250, 3572, 6480; main ICD10: E10, E11, E13, E14, N08.3, O24), atherosclerotic heart disease (main ICD9: 4140; main ICD10: I25.0, I25.1), hypertensive diseases (main ICD9: 401, 403; main ICD10: I10, I11, I12, I13, I15), atrial fibrillation and flutter (main ICD9: 4273; main ICD10: I48.0, I48.1, I48.2, I48.3, I48.4, I48.9), stroke (main ICD10: I63, I64), heart failure (main ICD9: 428; main ICD10: I50), peripheral artery and capillary disease (main ICD9: 440, 441, 442, 443, 444, 445, 446, 447, 448; main ICD10: I70, I71, I72, I73, I74, I75, I76, I77, I78, I79), chronic renal failure (main ICD9: 585; main ICD10: N18), liver disease (main ICD10: K70-K77), cancer (main ICD10: C00-C97), family history of heart disease, stroke, high blood pressure, diabetes, chronic bronchitis emphysema, dementia, Parkinson, depression, and cancer (Field ID: 20107, 20110, 20111).

The variables were described as follows: Genetic ancestry was classified as White British based on self-report and principal component analysis of genotypes. BMI was calculated from height and weight measured during the initial Assessment Centre visit using the formula weight (kg) / height^2 (m^2). Education level was classified into college degree or not. Smoking status was determined from "Current tobacco smoking" (Field ID: 1239) and "Past tobacco smoking" (Field ID: 1249). Ever smokers were those who reported smoking “most days” (1) or “occasionally” (2) in either current or

past smoking records, or who had tried smoking “once or twice” (3). Never smokers were those who reported “no” (0) for current smoking and “never” (4) for past smoking. Alcohol intake frequency was derived from the ACE touchscreen question "About how often do you drink alcohol?". The Townsend deprivation index was calculated based on national census output areas before participants joined UK Biobank, with scores assigned according to postcode location. Diet was assessed by diet score derived from five nutritional components, with one point awarded for each optimal consumption level: (1) vegetables ($\geq$4 tbsp/day), (2) fruits ($\geq$3 servings/day), (3) fish ($\geq$2 servings/week), while limiting (4) unprocessed red meat and (5) processed meats (both $\leq$2 servings/week). This composite score ranged theoretically from 0 (poorest) to 5 (optimal).(6) Physical activity levels were categorized into four groups: (1) inactive (no reported activity), (2) low (only light domestic activities), (3) moderate (vigorous gardening or recreational walking), and (4) high (intensive sports participation), based on self-reported activities during the preceding 4-week period.(7) Diseases were classified using ICD9 and ICD10 codes from hospital inpatient records.

For analysing the association between CHIP and AP, model 1 included age, sex, genetic ancestry, BMI, smoking status, alcohol intake frequency, Townsend deprivation index, diabetes, and atherosclerotic heart disease. Model 2 additionally accounted for hypertensive diseases, atrial fibrillation and flutter, heart failure, peripheral artery and capillary disease, stroke, and chronic renal failure based on model 1. Model 3 additionally adjusted for education level, sleep duration, physical activity, diet score based on model 2. Model 4 additionally adjusted for waist circumference, systolic blood pressure, diastolic blood pressure, total cholesterol, low-density lipoprotein cholesterol, high-density lipoprotein cholesterol, triglycerides, glucose glycated haemoglobin, estimated glomerular filtration rate based on model 3. Model 5 additionally adjusted for lipid lowering medication, anti-hypertensive medication, antidiabetic medication based on model 4. Model 6 additionally adjusted for prevalent cancer and liver disease based on model 5. Model 7 additionally adjusted for based on family history of diseases based on model 6. This comprehensive set of covariates was used in analysis of the continuous relationship between VAF and AP, as well as in subgroup analysis.

Analysis of continuous association between VAF and AP

To study the dose-response relationship between CHIP and AP, VAF was analysed as a continuous variable, which has been previously mentioned. (8) To address potential confounding from the skewed distribution of VAF, the VAF for participants without CHIP was set to 1%, which is half of the threshold for any CHIP. VAF was also capped at 25% due to the small number of individuals above that level.

Subgroup analysis

To explore the interactions between CHIP and various covariates, subgroup analysis was conducted on CHIP status, which has been associated with AP in covariate and PSM models. The study population was stratified by sex, ancestry, BMI, smoking history, alcohol intake frequency, Townsend deprivation index, diabetes, hypertensive diseases, atrial fibrillation and flutter, stroke, heart failure, peripheral artery and capillary disease, and chronic renal failure. Participants with a BMI ≥ 28 were classified as the high BMI group, while those with a BMI < 28 were classified as the low BMI group. The Townsend deprivation index was divided into high ($\geq$ median level) and low ($<$ median level) groups. Alcohol intake frequency was categorized as high for those who drank "daily or almost daily," "three or four times a week," or "once or twice a week," and as low for those who drank "one to three times a month," "on special occasions only," or "never."

Genome-wide association study

In the UKB cohort, genotyping was performed using Affymetrix arrays: about 50,000 participants were genotyped on the UK BiLEVE Axiom array, and around 450,000 on the UK Biobank Axiom array. The combined results included 805,426 markers mapped to GRCh37 coordinates. Genotyping was not possible for approximately 3% of participants due to insufficient blood sample DNA. GWAS was conducted among unrelated White British participants, with adjustments for age, sex, and 10 genetic principal components to control for population structure effects.

Mendelian randomization analysis

GWAS summaries from the UKB cohort were obtained using the outlined procedures. Instrumental variants (IVs) were selected based on a genome-wide significance threshold (P value $\leq 1\times10^{-5}$) and an effect allele frequency (EAF) greater than 0.01 in genotype-exposure data, to exclude variants directly associated with the outcome but not through the exposure. IVs were then clumped at an r^2 of 0.5 to minimize false positives, and palindromic variants were removed. Variants with an F statistic $\leqslant$ 10 were excluded to avoid weak IVs.(9)

Various MR methods were used to analyse causal effects, including inverse variance weighted (IVW), MR-Egger, weighted-median, simple mode, and weighted-mode approaches. The IVW method with a random-effects model was primarily used.(10) The MR-Egger method provided robust estimates even in the presence of horizontal pleiotropy.(11) The weighted median method gave the median MR estimate to account for heterogeneity and outliers.(12) To validate robustness, additional adjusted methods were adopted including maximum likelihood, simple median, penalized weighted median, IVW radial, IVW (multiplicative random effects), and IVW (fixed effects).

For sensitivity analysis, pleiotropy was assessed using the intercept P value from MR-Egger, and heterogeneity was measured with Cochran's Q statistic. To further address potential pleiotropy, IVs associated with risk factors for AP were identified using the GWAS Catalogue project, which includes variant-trait associations and metadata from over 45,000 published GWAS studies.(13) Reverse MR analysis and Steiger-filtering test were conducted to investigate reverse causal relationships. MR analysis was performed using the TwoSampleMR R package version 0.5.10.

Proteomic measurement, processing and quality control

UK Biobank Pharma Proteomics Project consortium collected blood samples in 9 mL EDTA vacutainers, which were then divided into 850 μL aliquots of plasma, buffy coat, and red cells. The plasma was stored at −80°C and later shipped on dry ice to Olink Analysis Service in Sweden. Between April 2021 and January 2022, 1463 unique proteins were measured using Proximity Extension Assay combined with Next-Generation Sequencing, following stringent quality control procedures (see biobank.ndph.ox.ac.uk/ukb/ukb/docs/PPP_Phase_1_QC_dataset_companion_doc.pdf). The proteins were analysed across four panels: cardiometabolic, inflammation, neurology, and oncology. Detailed information on sample selection, processing, and quality control was available in prior publications.(14, 15)

Metabolomic processing and mediation analysis

Metabolic biomarker data from the UK Biobank, collected between March 2006 and October 2010 using NMR spectroscopy on the Nightingale platform, were utilized in this study. Plasma samples from approximately 280,000 participants were analysed, resulting in measurements of 251 metabolic biomarkers, including lipoprotein lipids, fatty acids, amino acids, ketone bodies, and glycolytic metabolic biomarkers. The data were reported in molar concentrations from EDTA plasma samples, with quality control measures applied to minimize variability. A natural logarithm transformation was applied to each metabolic biomarker. Data from Phase 1 were last updated in July 2023 and may be subject to further revisions following recalibration and normalization.(16, 17)

For mediation analysis, we first identifying metabolites associated with both CHIP and AP or its subtypes, then conducting formal mediation analysis with these candidate mediators. The mediating effects were further analysed via the mediation R package version 4.5.0.

Single cell sequencing data acquisition and processing

Previously published single cell sequencing (scRNA-seq) data from Gene Expression Omnibus database was included for reanalysis. (GSE248395) The 20% Cx3cr1-cre Tet2 fl/fl bone marrow mixed with 80% WT bone marrow was transplanted into $Ldlr^{-/-}$ mice. Mice were pulsed with tamoxifen, then put on a western diet for the last 6 weeks of diet mice were given IgG or IL-1b antibodies.

Cells were removed under the condition of expressing fewer than 3200 genes or greater than 50% mitochondrial genes. R package Seurat (version 4.2.0) were used to perform dimensional reduction of scRNA-seq data. The "NormalizeData" and "ScaleData" function from Seurat was used for

normalization, then followed by “FindVariableFeatures” to calculate highly variable genes. Cells were projected in 2D space using Uniform Manifold Approximation and Projection (UMAP). To identify differentially expressed genes (DEGs) among each cluster, the “FindAllMarkers” function from Seurat was used and non-parametric Wilcoxon rank sum tests were set to evaluate the significance of each individual DEG. “DimPlot” and “VlnPlot” were used to visualize the expression of individual genes, cells were grouped by their cell type as determined by analysis with Seurat. Pathway enrichment was performed by ‘fgsea’ R package (version 1.27.1). Gene set was extracted from ‘MH’ geneset in ‘msigbr’ R package (version 7.5.1).

References

1. Van Hout CV, Tachmazidou I, Backman JD, Hoffman JD, Liu D, Pandey AK, et al. Exome sequencing and characterization of 49,960 individuals in the UK Biobank. Nature. 2020;586(7831):749-56.
2. Tate JG, Bamford S, Jubb HC, Sondka Z, Beare DM, Bindal N, et al. COSMIC: the Catalogue Of Somatic Mutations In Cancer. Nucleic acids research. 2019;47(D1):D941-d7.
3. Jaiswal S, Natarajan P, Silver AJ, Gibson CJ, Bick AG, Shvartz E, et al. Clonal Haematopoiesis and Risk of Atherosclerotic Cardiovascular Disease. The New England journal of medicine. 2017;377(2):111-21.
4. Wong WJ, Emdin C, Bick AG, Zekavat SM, Niroula A, Pirruccello JP, et al. Clonal haematopoiesis and risk of chronic liver disease. Nature. 2023;616(7958):747-54.
5. Bick AG, Weinstock JS, Nandakumar SK, Fulco CP, Bao EL, Zekavat SM, et al. Inherited causes of clonal haematopoiesis in 97,691 whole genomes. Nature. 2020;586(7831):763-8.
6. Wang M, Zhou T, Song Y, Li X, Ma H, Hu Y, et al. Joint exposure to various ambient air pollutants and incident heart failure: a prospective analysis in UK Biobank. European heart journal. 2021;42(16):1582-91.
7. Hanlon P, Nicholl BI, Jani BD, Lee D, McQueenie R, Mair FS. Frailty and pre-frailty in middle-aged and older adults and its association with multimorbidity and mortality: a prospective analysis of 493 737 UK Biobank participants. The Lancet Public health. 2018;3(7):e323-e32.
8. Saadatagah S, Naderian M, Uddin M, Dikilitas O, Niroula A, Schuermans A, et al. Atrial Fibrillation and Clonal Haematopoiesis in TET2 and ASXL1. JAMA cardiology. 2024;9(6):497-506.
9. Pierce BL, Ahsan H, Vanderweele TJ. Power and instrument strength requirements for Mendelian randomization studies using multiple genetic variants. International journal of epidemiology. 2011;40(3):740-52.
10. Burgess S, Scott RA, Timpson NJ, Davey Smith G, Thompson SG. Using published data in Mendelian randomization: a blueprint for efficient identification of causal risk factors. European journal of epidemiology. 2015;30(7):543-52.
11. Bowden J, Davey Smith G, Burgess S. Mendelian randomization with invalid instruments: effect estimation and bias detection through Egger regression. International journal of epidemiology. 2015;44(2):512-25.
12. Bowden J, Davey Smith G, Haycock PC, Burgess S. Consistent Estimation in Mendelian Randomization with Some Invalid Instruments Using a Weighted Median Estimator. Genetic epidemiology. 2016;40(4):304-14.
13. Sollis E, Mosaku A, Abid A, Buniello A, Cerezo M, Gil L, et al. The NHGRI-EBI GWAS Catalog: knowledgebase and deposition resource. Nucleic acids research. 2023;51(D1):D977-d85.
14. Elliott P, Peakman TC. The UK Biobank sample handling and storage protocol for the collection, processing and archiving of human blood and urine. International journal of epidemiology. 2008;37(2):234-44.
15. Sun BB, Chiou J, Traylor M, Benner C, Hsu YH, Richardson TG, et al. Plasma proteomic associations with genetics and health in the UK Biobank. Nature. 2023;622(7982):329-38.
16. Julkunen H, Cichońska A, Slagboom PE, Würtz P. Metabolic biomarker profiling for identification of susceptibility to severe pneumonia and COVID-19 in the general population. eLife. 2021;10.
17. Ritchie SC, Surendran P, Karthikeyan S, Lambert SA, Bolton T, Pennells L, et al. Quality control and removal of technical variation of NMR metabolic biomarker data in ~120,000 UK Biobank participants. Scientific data. 2023;10(1):64.

Supplementary Figures

Supplementary Figure S1: Study design

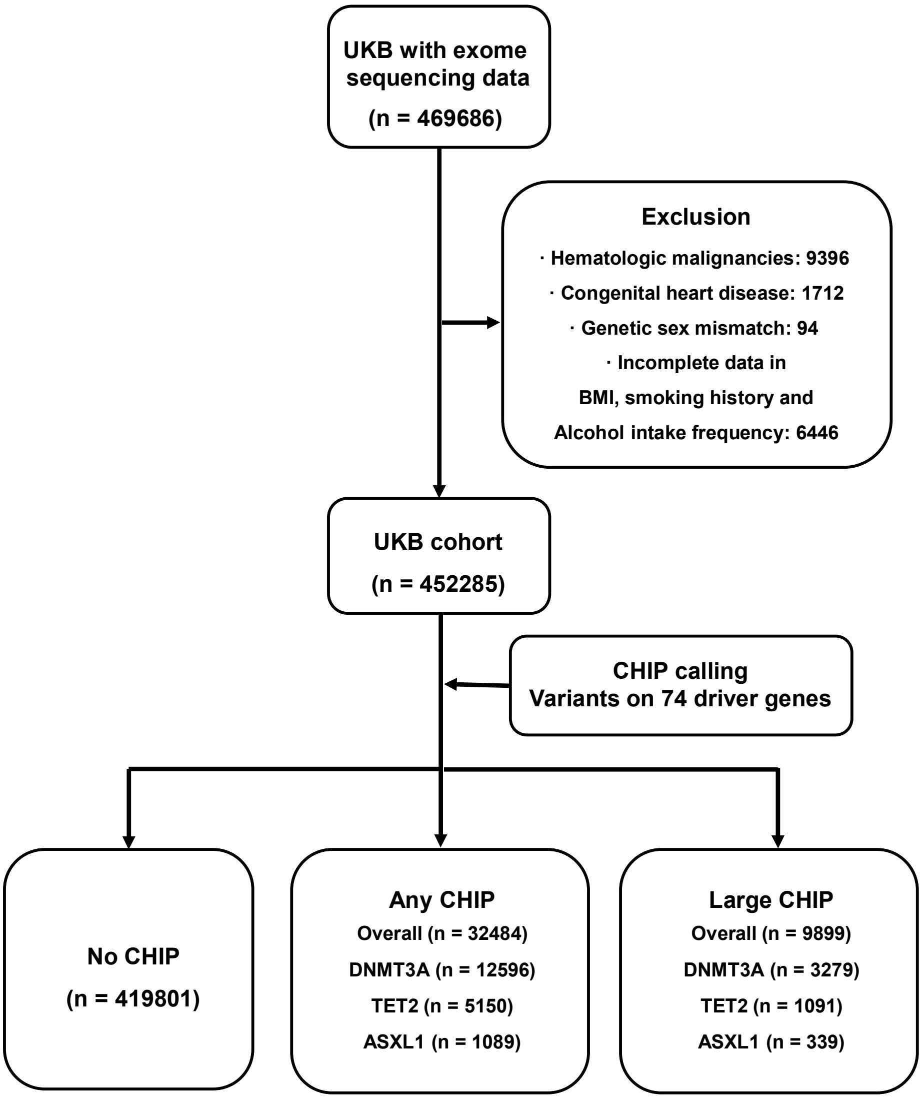

Abbreviations: UKB, UK Biobank; CHIP, clonal haematopoiesis of indeterminate potential; BMI, body mass index.

Supplementary Figure S2: Association of different CHIP status and AP in covariate model 1

Angina pectoris type and CHIP status	OR (95% CI)	P value
**Angina pectoris**		
Any CHIP		
overall	1.00 (0.93 − 1.08)	9.5×10−1
DNMT3A	1.03 (0.92 − 1.15)	6.1×10−1
TET2	0.90 (0.76 − 1.08)	2.6×10−1
ASXL1	1.35 (1.00 − 1.81)	5.1×10−2
Large CHIP		
overall	0.82 (0.72 − 0.93)	3.1×10−3
DNMT3A	0.84 (0.67 − 1.05)	1.2×10−1
TET2	0.56 (0.37 − 0.85)	6.7×10−3
ASXL*1*	0.91 (0.52 − 1.61)	7.5×10−1
**Unstable angina pectoris**		
Any CHIP		
overall	0.94 (0.85 − 1.05)	2.7×10−1
DNMT3A	0.99 (0.84 − 1.16)	9.1×10−1
TET2	0.80 (0.61 − 1.05)	1.1×10−1
ASXL1	1.28 (0.84 − 1.95)	2.6×10−1
Large CHIP		
overall	0.74 (0.61 − 0.91)	3.4×10−3
DNMT3A	0.94 (0.69 − 1.29)	7.2×10−1
TET2	0.47 (0.24 − 0.92)	2.7×10−2
ASXL1	0.63 (0.25 − 1.59)	3.3×10−1
**Stable angina pectoris**		
Any CHIP		
overall	1.16 (0.91 − 1.47)	2.3×10−1
DNMT3A	1.23 (0.85 − 1.76)	2.7×10−1
TET2	1.54 (0.94 − 2.54)	8.9×10−2
ASXL1	1.09 (0.35 − 3.41)	8.9×10−1
Large CHIP		
overall	1.08 (0.71 − 1.66)	7.1×10−1
DNMT3A	1.35 (0.70 − 2.62)	3.7×10−1
TET2	1.16 (0.37 − 3.63)	8.0×10−1
ASXL1	1.00 (0.14 − 7.24)	10.0×10−1

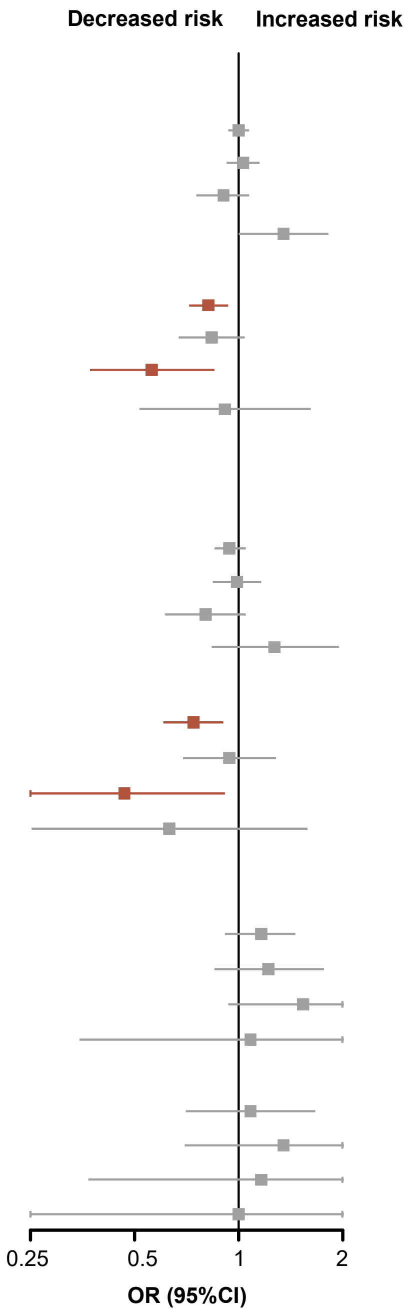

Model 1 adjusted for age at recruitment, sex, genetic ancestry, BMI, ever smoked, alcohol intake frequency, Townsend deprivation index, diabetes, atherosclerotic heart disease. Abbreviations: BMI, body mass index.

Supplementary Figure S3: Association of different CHIP status and AP in covariate model 3

Angina pectoris type and CHIP status	OR (95% CI)	P value
**Angina pectoris**		
**Any CHIP**		
overall	1.00 (0.93 − 1.07)	9.8×10−1
DNMT3A	1.03 (0.92 − 1.15)	6.0×10−1
TET2	0.91 (0.76 − 1.09)	3.0×10−1
ASXL1	1.33 (0.99 − 1.80)	5.9×10−2
**Large CHIP**		
overall	0.81 (0.71 − 0.92)	1.6×10−3
DNMT3A	0.83 (0.66 − 1.04)	1.1×10−1
TET2	0.55 (0.36 − 0.84)	5.5×10−3
ASXL1	0.90 (0.51 − 1.60)	7.2×10−1
**Unstable angina pectoris**		
**Any CHIP**		
overall	0.94 (0.84 − 1.04)	2.4×10−1
DNMT3A	0.99 (0.84 − 1.16)	9.1×10−1
TET2	0.81 (0.62 − 1.06)	1.2×10−1
ASXL1	1.26 (0.82 − 1.93)	2.9×10−1
**Large CHIP**		
overall	0.73 (0.59 − 0.89)	2.0×10−3
DNMT3A	0.93 (0.68 − 1.28)	6.7×10−1
TET2	0.46 (0.23 − 0.90)	2.3×10−2
ASXL1	0.61 (0.24 − 1.55)	3×10−1
**Stable angina pectoris**		
**Any CHIP**		
overall	1.14 (0.90 − 1.45)	2.7×10−1
DNMT3A	1.21 (0.84 − 1.74)	2.9×10−1
TET2	1.52 (0.92 − 2.51)	9.9×10−2
ASXL1	1.07 (0.34 − 3.34)	9.1×10−1
**Large CHIP**		
overall	1.07 (0.70 − 1.64)	7.6×10−1
DNMT3A	1.34 (0.69 − 2.61)	3.8×10−1
TET2	1.12 (0.36 − 3.52)	8.5×10−1
ASXL1	0.99 (0.14 − 7.14)	9.9×10−1

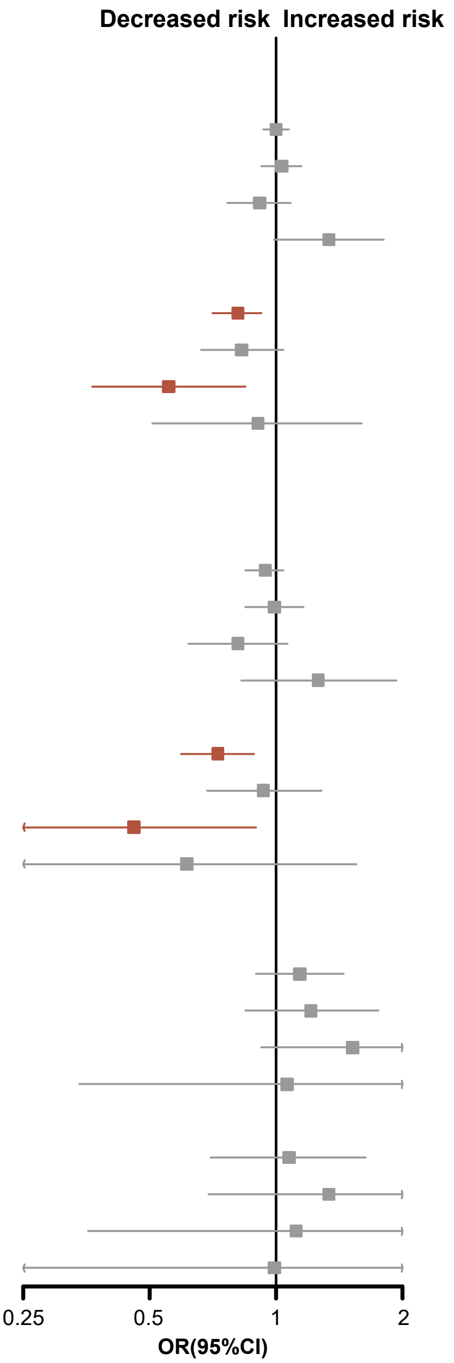

Model 3 adjusted for age, sex, genetic ancestry, BMI, smoking status, alcohol intake status, Townsend deprivation index, diabetes, atherosclerotic heart disease, hypertensive diseases, atrial fibrillation and flutter, stroke, heart failure, peripheral artery and capillary disease, chronic renal failure, education level, sleep duration, physical activity, diet score.

Supplementary Figure S4: Association of different CHIP status and AP in covariate model 4

Angina pectoris type and CHIP status	OR (95% CI)	P value
**Angina pectoris**		
**Any CHIP**		
overall	1.00 (0.93 − 1.07)	9.2×10−1
DNMT3A	1.03 (0.92 − 1.15)	6.1×10−1
TET2	0.90 (0.76 − 1.08)	2.6×10−1
ASXL1	1.32 (0.98 − 1.79)	6.8×10−2
**Large CHIP**		
overall	0.81 (0.71 − 0.92)	1.7×10−3
DNMT3A	0.83 (0.66 − 1.04)	1.0×10−1
TET2	0.55 (0.36 − 0.84)	6.0×10−3
ASXL1	0.88 (0.50 − 1.57)	6.8×10−1
**Unstable angina pectoris**		
**Any CHIP**		
overall	0.94 (0.84 − 1.04)	2.2×10−1
DNMT3A	0.99 (0.84 − 1.16)	8.9×10−1
TET2	0.80 (0.61 − 1.04)	1.0×10−1
ASXL1	1.26 (0.82 − 1.92)	2.9×10−1
**Large CHIP**		
overall	0.73 (0.59 − 0.89)	2.0×10−3
DNMT3A	0.94 (0.68 − 1.28)	6.8×10−1
TET2	0.45 (0.23 − 0.89)	2.1×10−2
ASXL1	0.62 (0.25 − 1.55)	3.1×10−1
**Stable angina pectoris**		
**Any CHIP**		
overall	1.14 (0.90 − 1.45)	2.8×10−1
DNMT3A	1.21 (0.84 − 1.74)	3.0×10−1
TET2	1.52 (0.92 − 2.50)	1.0×10−1
ASXL1	1.07 (0.34 − 3.36)	9.1×10−1
**Large CHIP**		
overall	1.07 (0.70 − 1.64)	7.5×10−1
DNMT3A	1.35 (0.69 − 2.62)	3.8×10−1
TET2	1.14 (0.36 − 3.58)	8.2×10−1
ASXL1	0.99 (0.14 − 7.20)	10×10−1

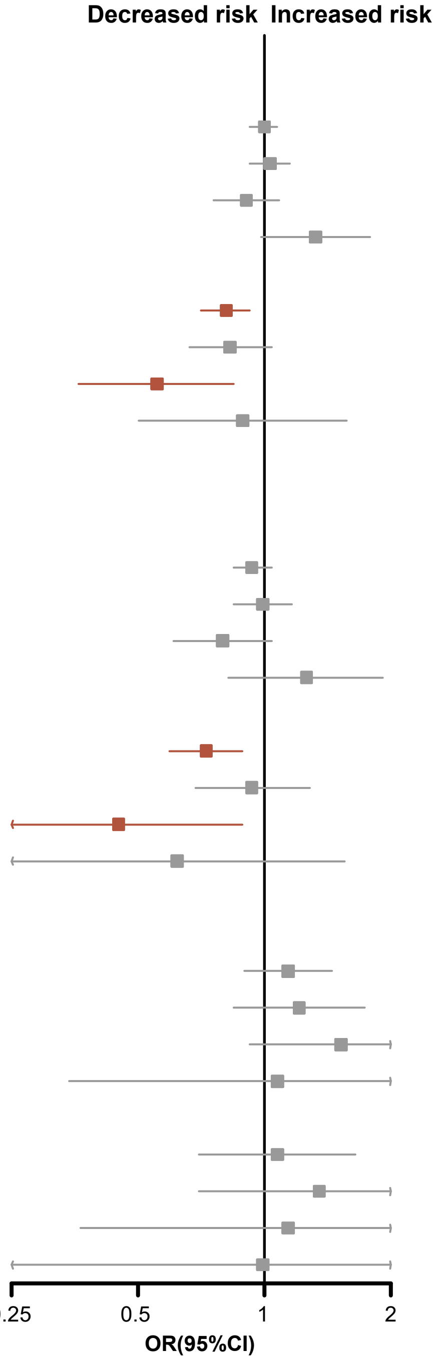

Model 4 adjusted for age, sex, genetic ancestry, BMI, smoking status, alcohol intake status, Townsend deprivation index, diabetes, atherosclerotic heart disease, hypertensive diseases, atrial fibrillation and flutter, stroke, heart failure, peripheral artery and capillary disease, chronic renal failure, education level, sleep duration, physical activity, diet score, waist circumference, systolic blood pressure, diastolic blood pressure, total cholesterol, low-density lipoprotein cholesterol (LDL-C), high-density lipoprotein cholesterol (HDL-C), triglycerides, glucose glycated haemoglobin (HbA1c), estimated glomerular filtration rate (eGFR).

Supplementary Figure S5: Association of different CHIP status and AP in covariate model 5

Angina pectoris type and CHIP status	OR (95% CI)	P value
**Angina pectoris**		
**Any CHIP**		
overall	1.00 (0.93 − 1.07)	9.4×10−1
DNMT3A	1.03 (0.93 − 1.16)	5.5×10−1
TET2	0.89 (0.75 − 1.07)	2.1×10−1
ASXL1	1.32 (0.97 − 1.78)	7.5×10−2
**Large CHIP**		
overall	0.81 (0.71 − 0.93)	2.1×10−3
DNMT3A	0.83 (0.66 − 1.04)	1.1×10−1
TET2	0.55 (0.36 − 0.84)	5.9×10−3
ASXL1	0.88 (0.49 − 1.56)	6.6×10−1
**Unstable angina pectoris**		
**Any CHIP**		
overall	0.94 (0.84 − 1.04)	2.2×10−1
DNMT3A	0.99 (0.84 − 1.17)	9.4×10−1
TET2	0.78 (0.60 − 1.03)	8.0×10−2
ASXL1	1.25 (0.82 − 1.92)	3.0×10−1
**Large CHIP**		
overall	0.73 (0.60 − 0.89)	2.3×10−3
DNMT3A	0.94 (0.68 − 1.29)	6.9×10−1
TET2	0.45 (0.23 − 0.89)	2.2×10−2
ASXL1	0.61 (0.24 − 1.54)	2.9×10−1
**Stable angina pectoris**		
**Any CHIP**		
overall	1.14 (0.90 − 1.45)	2.8×10−1
DNMT3A	1.21 (0.85 − 1.75)	2.9×10−1
TET2	1.51 (0.91 − 2.48)	1.1×10−1
ASXL1	1.07 (0.34 − 3.37)	9.0×10−1
**Large CHIP**		
overall	1.08 (0.70 − 1.65)	7.4×10−1
DNMT3A	1.35 (0.69 − 2.62)	3.8×10−1
TET2	1.15 (0.36 − 3.60)	8.2×10−1
ASXL1	1.00 (0.14 − 7.24)	1

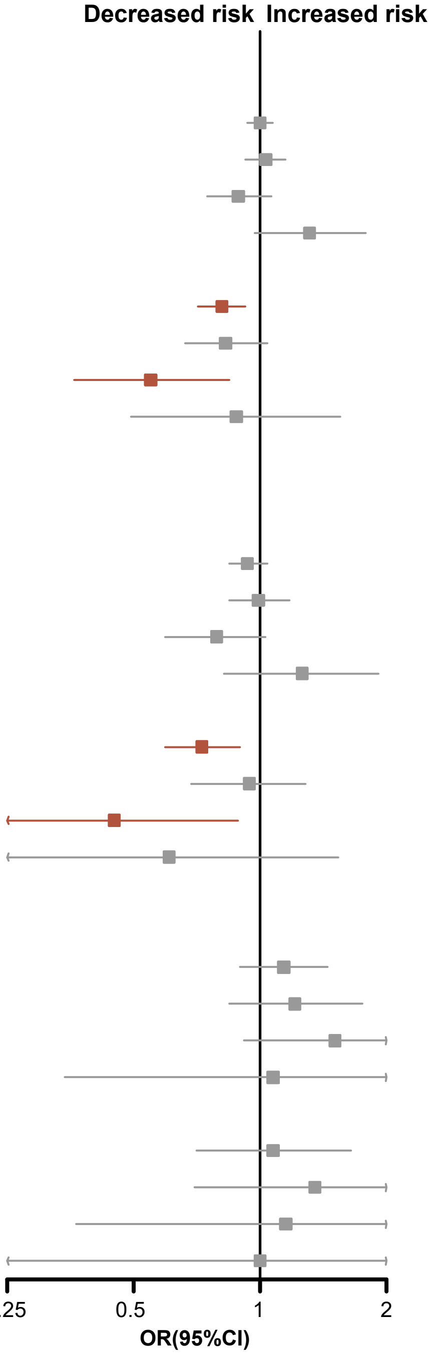

Model 5 adjusted for age, sex, genetic ancestry, BMI, smoking status, alcohol intake status, Townsend deprivation index, diabetes, atherosclerotic heart disease, hypertensive diseases, atrial fibrillation and flutter, stroke, heart failure, peripheral artery and capillary disease, chronic renal failure, education level, sleep duration, physical activity, diet score, waist circumference, systolic blood pressure, diastolic blood pressure, total cholesterol, low-density lipoprotein cholesterol (LDL-C), high-density lipoprotein cholesterol (HDL-C), triglycerides, glucose glycated haemoglobin (HbA1c), estimated glomerular filtration rate (eGFR), lipid-lowering medication, anti-hypertensive medication, antidiabetic medication.

Supplementary Figure S6: Association of different CHIP status and AP in covariate model 6

Angina pectoris type and CHIP status	OR (95% CI)	P value
**Angina pectoris**		
**Any CHIP**		
overall	0.99 (0.92 − 1.07)	8.7×10−1
DNMT3A	1.03 (0.92 − 1.15)	5.8×10−1
TET2	0.89 (0.75 − 1.06)	2.0×10−1
ASXL1	1.31 (0.97 − 1.77)	8.1×10−2
**Large CHIP**		
overall	0.81 (0.70 − 0.92)	1.6×10−3
DNMT3A	0.82 (0.66 − 1.04)	9.7×10−2
TET2	0.55 (0.36 − 0.84)	5.9×10−3
ASXL1	0.86 (0.48 − 1.53)	6.0×10−1
**Unstable angina pectoris**		
**Any CHIP**		
overall	0.93 (0.84 − 1.04)	1.9×10−1
DNMT3A	0.99 (0.84 − 1.16)	8.8×10−1
TET2	0.78 (0.60 − 1.03)	8.0×10−2
ASXL1	1.25 (0.81 − 1.91)	3.1×10−1
**Large CHIP**		
overall	0.72 (0.59 − 0.89)	1.8×10−3
DNMT3A	0.93 (0.68 − 1.28)	6.5×10−1
TET2	0.45 (0.23 − 0.90)	2.3×10−2
ASXL1	0.59 (0.24 − 1.50)	2.7×10−1
**Stable angina pectoris**		
**Any CHIP**		
overall	1.14 (0.90 − 1.45)	2.9×10−1
DNMT3A	1.21 (0.84 − 1.74)	3.0×10−1
TET2	1.51 (0.91 − 2.49)	1.1×10−1
ASXL1	1.07 (0.34 − 3.36)	9.1×10−1
**Large CHIP**		
overall	1.07 (0.70 − 1.64)	7.5×10−1
DNMT3A	1.34 (0.69 − 2.60)	3.9×10−1
TET2	1.15 (0.37 − 3.62)	8.1×10−1
ASXL1	0.99 (0.14 − 7.15)	9.9×10−1

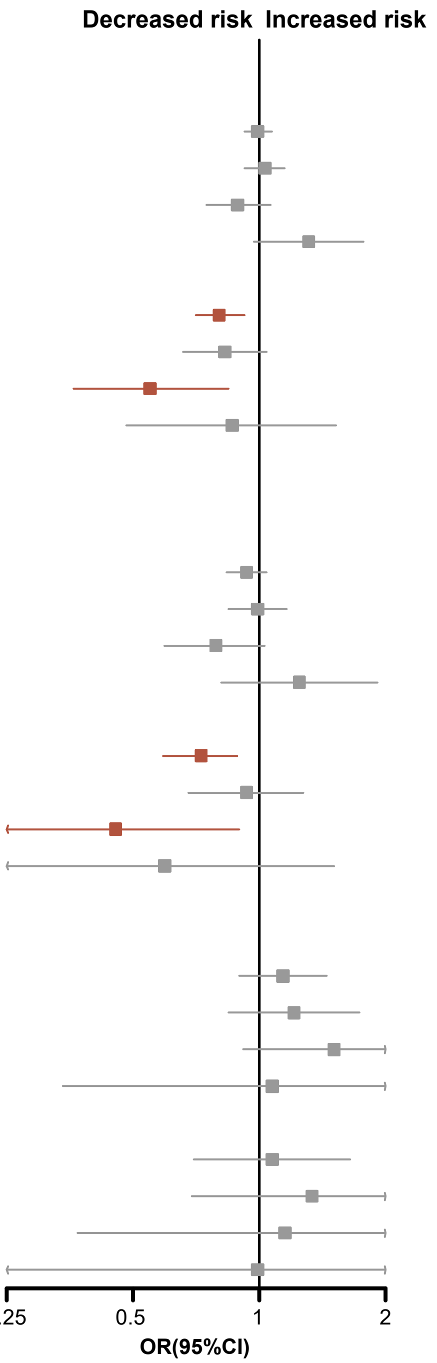

Model 6 adjusted for age, sex, genetic ancestry, BMI, smoking status, alcohol intake status, Townsend deprivation index, diabetes, atherosclerotic heart disease, hypertensive diseases, atrial fibrillation and flutter, stroke, heart failure, peripheral artery and capillary disease, chronic renal failure, education level, sleep duration, physical activity, diet score, waist circumference, systolic blood pressure, diastolic blood pressure, total cholesterol, low-density lipoprotein cholesterol (LDL-C), high-density lipoprotein cholesterol (HDL-C), triglycerides, glucose glycated haemoglobin (HbA1c), estimated glomerular filtration rate (eGFR), lipid-lowering medication, anti-hypertensive medication, antidiabetic medication, cancer, liver disease.

Supplementary Figure S7: Association of different CHIP status and AP in covariate model 7

Angina pectoris type and CHIP status	OR (95% CI)	P value
**Angina pectoris**		
**Any CHIP**		
overall	1.00 (0.93 − 1.07)	9.8×10−1
DNMT3A	1.04 (0.93 − 1.16)	4.8×10−1
TET2	0.89 (0.74 − 1.06)	1.8×10−1
ASXL1	1.32 (0.97 − 1.78)	7.3×10−2
**Large CHIP**		
overall	0.81 (0.71 − 0.93)	2.7×10−3
DNMT3A	0.84 (0.67 − 1.05)	1.3×10−1
TET2	0.55 (0.36 − 0.84)	5.5×10−3
ASXL1	0.86 (0.48 − 1.54)	6.1×10−1
**Unstable angina pectoris**		
**Any CHIP**		
overall	0.94 (0.84 − 1.04)	2.3×10−1
DNMT3A	1.00 (0.85 − 1.17)	9.6×10−1
TET2	0.78 (0.59 − 1.03)	7.6×10−2
ASXL1	1.27 (0.83 − 1.94)	2.8×10−1
**Large CHIP**		
overall	0.73 (0.60 − 0.90)	2.6×10−3
DNMT3A	0.95 (0.69 − 1.31)	7.6×10−1
TET2	0.45 (0.23 − 0.89)	2.2×10−2
ASXL1	0.60 (0.24 − 1.53)	2.9×10−1
**Stable angina pectoris**		
**Any CHIP**		
overall	1.14 (0.90 − 1.45)	2.7×10−1
DNMT3A	1.23 (0.85 − 1.76)	2.7×10−1
TET2	1.50 (0.91 − 2.48)	1.1×10−1
ASXL1	1.07 (0.34 − 3.34)	9.1×10−1
**Large CHIP**		
overall	1.09 (0.71 − 1.67)	7.0×10−1
DNMT3A	1.37 (0.70 − 2.65)	3.6×10−1
TET2	1.15 (0.37 − 3.60)	8.1×10−1
ASXL1	0.98 (0.13 − 7.07)	9.8×10−1

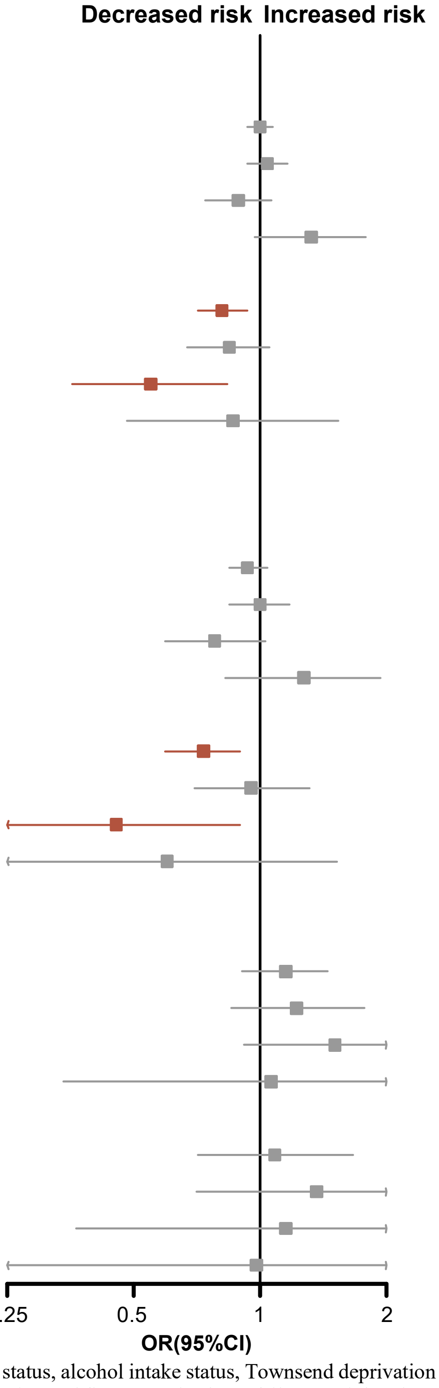

Model 7 adjusted for age, sex, genetic ancestry, BMI, smoking status, alcohol intake status, Townsend deprivation index, diabetes, atherosclerotic heart disease, hypertensive diseases, atrial fibrillation and flutter, stroke, heart failure, peripheral artery and capillary disease, chronic renal failure, education level, sleep duration, physical activity, diet score, waist circumference, systolic blood pressure, diastolic blood pressure, total cholesterol, low-density lipoprotein cholesterol (LDL-C), high-density lipoprotein cholesterol (HDL-C), triglycerides, glucose glycated haemoglobin (HbA1c), estimated glomerular filtration rate (eGFR), lipid-lowering medication, anti-hypertensive medication, antidiabetic medication, cancer, liver disease, family history of diseases.

Supplementary Figure S8: Association of different CHIP status and AP in covariate model 7 not adjusting for population confounders

Angina pectoris type and CHIP status	OR (95% CI)	P value
**Angina pectoris**		
**Any CHIP**		
overall	1.00 (0.93 − 1.08)	9.9×10−1
DNMT3A	1.04 (0.93 − 1.17)	4.6×10−1
TET2	0.89 (0.74 − 1.06)	1.9×10−1
ASXL1	1.32 (0.97 − 1.78)	7.4×10−2
**Large CHIP**		
overall	0.82 (0.71 − 0.93)	3.0×10−3
DNMT3A	0.84 (0.67 − 1.06)	1.4×10−1
TET2	0.55 (0.36 − 0.84)	5.4×10−3
ASXL1	0.86 (0.48 − 1.53)	6.0×10−1
**Unstable angina pectoris**		
**Any CHIP**		
overall	0.94 (0.84 − 1.04)	2.4×10−1
DNMT3A	1.00 (0.85 − 1.17)	9.8×10−1
TET2	0.78 (0.60 − 1.03)	7.7×10−2
ASXL1	1.26 (0.82 − 1.94)	2.8×10−1
**Large CHIP**		
overall	0.73 (0.60 − 0.90)	2.8×10−3
DNMT3A	0.95 (0.70 − 1.31)	7.7×10−1
TET2	0.45 (0.23 − 0.89)	2.2×10−2
ASXL1	0.60 (0.24 − 1.51)	2.8×10−1
**Stable angina pectoris**		
**Any CHIP**		
overall	1.15 (0.90 − 1.46)	2.6×10−1
DNMT3A	1.23 (0.86 − 1.77)	2.6×10−1
TET2	1.50 (0.91 − 2.48)	1.1×10−1
ASXL1	1.06 (0.34 − 3.34)	9.2×10−1
**Large CHIP**		
overall	1.09 (0.71 − 1.67)	6.9×10−1
DNMT3A	1.37 (0.70 − 2.66)	3.5×10−1
TET2	1.15 (0.37 − 3.60)	8.1×10−1
ASXL1	0.97 (0.13 − 7.02)	9.8×10−1

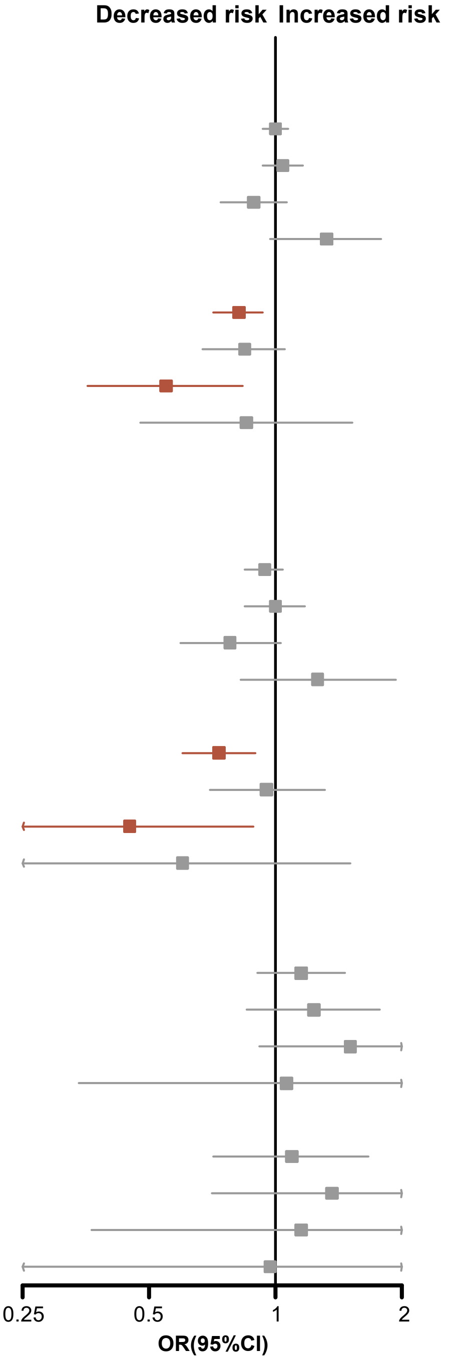

Model adjusted for age, sex, BMI, smoking status, alcohol intake status, Townsend deprivation index, diabetes, atherosclerotic heart disease, hypertensive diseases, atrial fibrillation and flutter, stroke, heart failure, peripheral artery and capillary disease, chronic renal failure, education level, sleep duration, physical activity, diet score, waist circumference, systolic blood pressure, diastolic blood pressure, total cholesterol, low-density lipoprotein cholesterol (LDL-C), high-density lipoprotein cholesterol (HDL-C), triglycerides, glucose glycated haemoglobin (HbA1c), estimated glomerular filtration rate (eGFR), lipid-lowering medication, anti-hypertensive medication, antidiabetic medication, cancer, liver disease, family history of diseases.

Supplementary Figure S9: Association of different CHIP status and AP in covariate model 7 in White population

Angina pectoris type and CHIP status	OR (95% CI)	P value
**Angina pectoris**		
**Any CHIP**		
overall	0.98 (0.89 − 1.08)	6.7×10−1
DNMT3A	1.02 (0.88 − 1.20)	7.6×10−1
TET2	0.92 (0.71 − 1.18)	5×10−1
ASXL1	1.46 (0.91 − 2.35)	1.1×10−1
**Large CHIP**		
overall	0.78 (0.65 − 0.93)	6.2×10−3
DNMT3A	0.85 (0.62 − 1.17)	3.2×10−1
TET2	0.46 (0.27 − 0.79)	4.9×10−3
ASXL1	1.11 (0.47 − 2.62)	8.1×10−1
**Unstable angina pectoris**		
**Any CHIP**		
overall	0.87 (0.76 − 1.01)	6.3×10−2
DNMT3A	0.83 (0.67 − 1.04)	1.1×10−1
TET2	0.79 (0.55 − 1.16)	2.3×10−1
ASXL1	1.32 (0.68 − 2.55)	4.1×10−1
**Large CHIP**		
overall	0.62 (0.48 − 0.82)	6.1×10−4
DNMT3A	1.05 (0.66 − 1.67)	8.3×10−1
TET2	0.32 (0.14 − 0.70)	4.5×10−3
ASXL1	0.79 (0.22 − 2.82)	7.2×10−1
**Stable angina pectoris**		
**Any CHIP**		
overall	1.18 (0.84 − 1.67)	3.3×10−1
DNMT3A	1.33 (0.76 − 2.30)	3.2×10−1
TET2	1.34 (0.22 − 8.13)	7.5×10−1
ASXL1	1.34 (0.43 − 4.12)	6.1×10−1
**Large CHIP**		
overall	1.03 (0.56 − 1.89)	9.3×10−1
DNMT3A	1.26 (0.48 − 3.30)	6.4×10−1
TET2	1.31 (0.23 − 7.39)	7.6×10−1
ASXL1	1.64 (0.12 − 21.96)	7.1×10−1

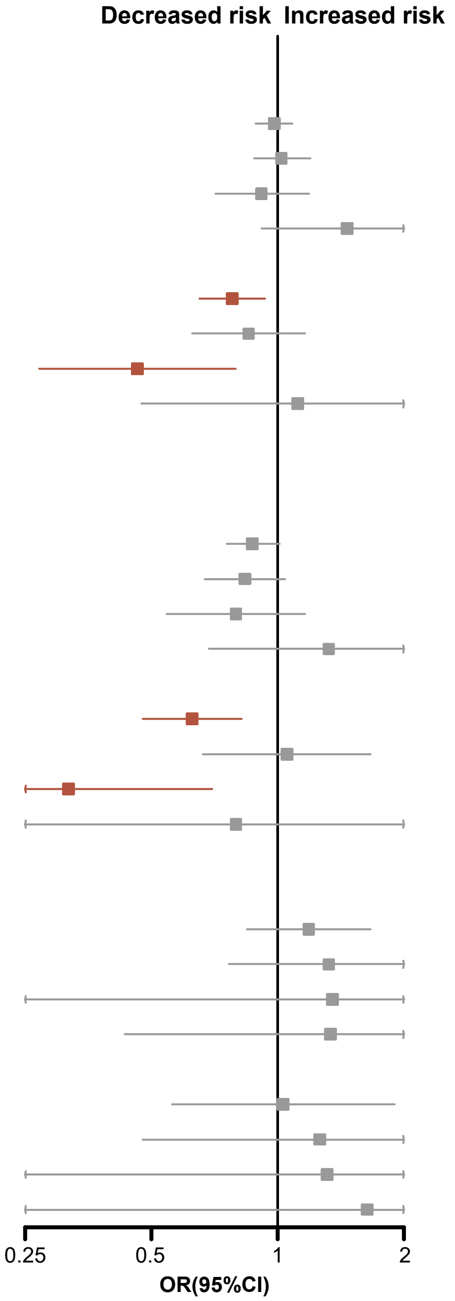

Model adjusted for age, sex, BMI, smoking status, alcohol intake status, Townsend deprivation index, diabetes, atherosclerotic heart disease, hypertensive diseases, atrial fibrillation and flutter, stroke, heart failure, peripheral artery and capillary disease, chronic renal failure, education level, sleep duration, physical activity, diet score, waist circumference, systolic blood pressure, diastolic blood pressure, total cholesterol, low-density lipoprotein cholesterol (LDL-C), high-density lipoprotein cholesterol (HDL-C), triglycerides, glucose glycated haemoglobin (HbA1c), estimated glomerular filtration rate (eGFR), lipid-lowering medication, anti-hypertensive medication, antidiabetic medication, cancer, liver disease, family history of diseases.

Supplementary Figure S10: Association of different CHIP status and AP in covariate model 7 in Asian population

Angina pectoris type and CHIP status	OR (95% CI)	P value
**Angina pectoris**		
**Any CHIP**		
overall	0.66 (0.41 − 1.05)	7.6×10−2
DNMT3A	0.53 (0.26 − 1.08)	8.1×10−2
TET2	0.01 (0.00 − 1.58)	7.1×10−2
ASXL1	1.27 (0.04 − 44.18)	9.0×10−1
**Large CHIP**		
overall	0.31 (0.14 − 0.66)	2.5×10−3
DNMT3A	0.63 (0.12 − 3.44)	5.9×10−1
TET2	0.32 (0.14 − 0.72)	6.6×10−3
ASXL1	0.57 (0.15 − 2.22)	4.2×10−1
**Unstable angina pectoris**		
**Any CHIP**		
overall	0.47 (0.26 − 0.85)	1.3×10−2
DNMT3A	0.57 (0.37 − 0.89)	1.3×10−2
TET2	0.61 (0.04 − 8.60)	7.2×10−1
ASXL1	0.68 (0.06 − 7.50)	7.5×10−1
**Large CHIP**		
overall	0.42 (0.18 − 0.99)	4.8×10−2
DNMT3A	0.51 (0.05 − 4.83)	5.6×10−1
TET2	0.29 (0.16 − 0.52)	2.3×10−5
ASXL1	0.37 (0.04 − 3.93)	4.1×10−1
**Stable angina pectoris**		
**Any CHIP**		
overall	1.06 (0.64 − 1.75)	8.2×10−1
DNMT3A	1.49 (0.07 − 30.54)	8.0×10−1
TET2	1.05 (0.25 − 4.48)	9.5×10−1
ASXL1	1.52 (0.07 − 30.96)	7.9×10−1
**Large CHIP**		
overall	1.02 (0.23 − 4.61)	9.8×10−1
DNMT3A	1.28 (0.19 − 8.69)	8.0×10−1
TET2	1.19 (0.08 − 16.64)	9.0×10−1
ASXL1	1.63 (0.34 − 7.94)	5.4×10−1

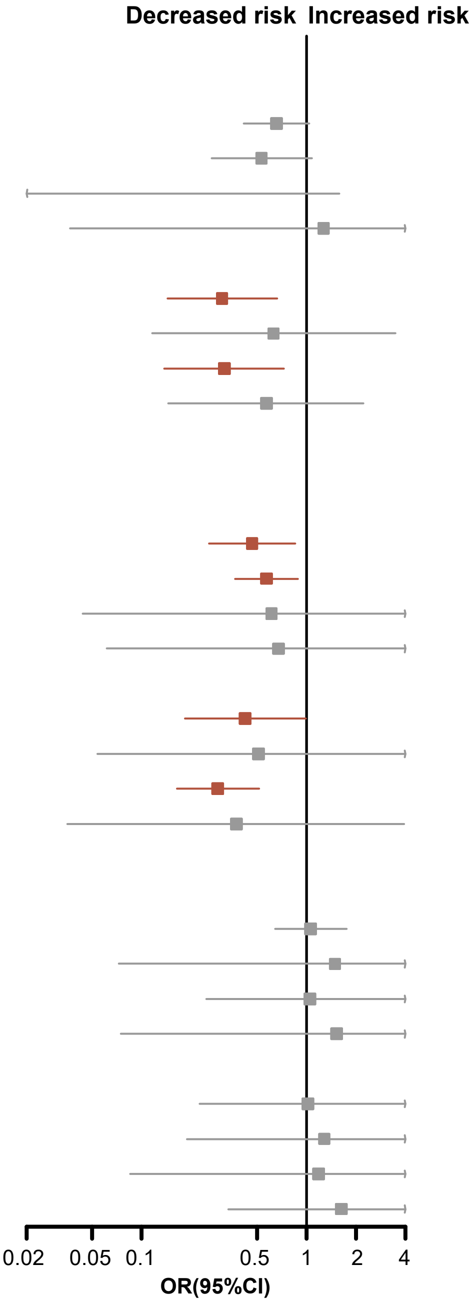

Model adjusted for age, sex, BMI, smoking status, alcohol intake status, Townsend deprivation index, diabetes, atherosclerotic heart disease, hypertensive diseases, atrial fibrillation and flutter, stroke, heart failure, peripheral artery and capillary disease, chronic renal failure, education level, sleep duration, physical activity, diet score, waist circumference, systolic blood pressure, diastolic blood pressure, total cholesterol, low-density lipoprotein cholesterol (LDL-C), high-density lipoprotein cholesterol (HDL-C), triglycerides, glucose glycated haemoglobin (HbA1c), estimated glomerular filtration rate (eGFR), lipid-lowering medication, anti-hypertensive medication, antidiabetic medication, cancer, liver disease, family history of diseases.

Supplementary Figure S11: Association of different CHIP status and AP in covariate model 7 in Black population

Angina pectoris type and CHIP status	OR (95% CI)	P value
**Angina pectoris**		
**Any CHIP**		
overall	0.59 (0.31 − 1.13)	1.1×10−1
DNMT3A	0.48 (0.18 − 1.30)	1.5×10−1
TET2	0.19 (0.02 − 1.84)	1.5×10−1
ASXL1	0.28 (0.00 − 23.27)	5.7×10−1
**Large CHIP**		
overall	0.66 (0.14 − 3.17)	6.1×10−1
DNMT3A	0.54 (0.11 − 2.67)	4.5×10−1
TET2	0.34 (0.16 − 0.72)	4.6×10−3
ASXL1	0.18 (0.02 − 1.50)	1.1×10−1
**Unstable angina pectoris**		
**Any CHIP**		
overall	0.62 (0.22 − 1.76)	3.7×10−1
DNMT3A	0.30 (0.06 − 1.37)	1.2×10−1
TET2	0.34 (0.09 − 1.24)	1.0×10−1
ASXL1	0.54 (0.02 − 14.89)	7.2×10−1
**Large CHIP**		
overall	0.34 (0.19 − 0.62)	4.3×10−4
DNMT3A	0.45 (0.11 − 1.95)	2.9×10−1
TET2	0.32 (0.12 − 0.86)	2.4×10−2
ASXL1	0.04 (0.00 − 263.99)	4.7×10−1
**Stable angina pectoris**		
**Any CHIP**		
overall	1.05 (0.21 − 5.34)	9.5×10−1
DNMT3A	7.06 (0.61 − 81.07)	1.2×10−1
TET2	5.53 (0.47 − 65.54)	1.7×10−1
ASXL1	0.27 (0.00 − 83.25)	6.6×10−1
**Large CHIP**		
overall	14.63 (0.42 − 513.11)	1.4×10−1
DNMT3A	3.30 (0.33 − 33.27)	3.1×10−1
TET2	1.50 (0.07 − 30.73)	7.9×10−1
ASXL1	1.62 (0.03 − 84.50)	8.1×10−1

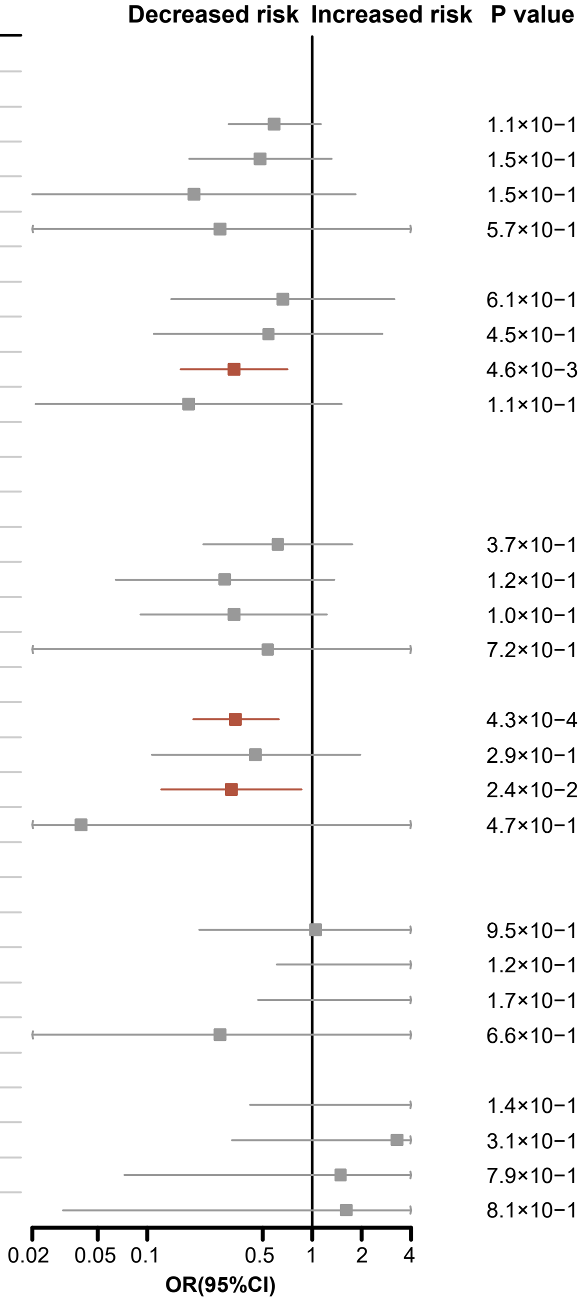

Model adjusted for age, sex, BMI, smoking status, alcohol intake status, Townsend deprivation index, diabetes, atherosclerotic heart disease, hypertensive diseases, atrial fibrillation and flutter, stroke, heart failure, peripheral artery and capillary disease, chronic renal failure, education level, sleep duration, physical activity, diet score, waist circumference, systolic blood pressure, diastolic blood pressure, total cholesterol, low-density lipoprotein cholesterol (LDL-C), high-density lipoprotein cholesterol (HDL-C), triglycerides, glucose glycated haemoglobin (HbA1c), estimated glomerular filtration rate (eGFR), lipid-lowering medication, anti-hypertensive medication, antidiabetic medication, cancer, liver disease, family history of diseases.

Supplementary Figure S12: Association of different CHIP status and ASCVD and CAD in covariate model 7

CHIP status	OR (95% CI)	P value
**ASCVD**		
**Any CHIP**		
overall	1.02 (0.99 − 1.06)	3.9×10−1
DNMT3A	1.05 (0.98 − 1.13)	1.4×10−1
TET2	1.18 (1.01 − 1.37)	3.9×10−2
ASXL1	1.14 (0.94 − 1.39)	1.9×10−1
**Large CHIP**		
overall	1.08 (1.02 − 1.15)	1.2×10−2
DNMT3A	1.14 (0.98 − 1.33)	8.4×10−2
TET2	1.14 (0.94 − 1.38)	1.7×10−1
ASXL1	1.40 (1.00 − 1.95)	4.8×10−2
**CAD**		
**Any CHIP**		
overall	1.00 (0.96 − 1.04)	9.5×10−1
DNMT3A	1.01 (0.93 − 1.09)	8.6×10−1
TET2	1.07 (0.94 − 1.22)	3.0×10−1
ASXL1	1.23 (0.99 − 1.51)	5.7×10−2
**Large CHIP**		
overall	1.08 (1.01 − 1.16)	2.2×10−2
DNMT3A	1.08 (0.91 − 1.28)	3.7×10−1
TET2	1.12 (0.91 − 1.36)	2.9×10−1
ASXL1	1.46 (1.02 − 2.11)	4.1×10−2

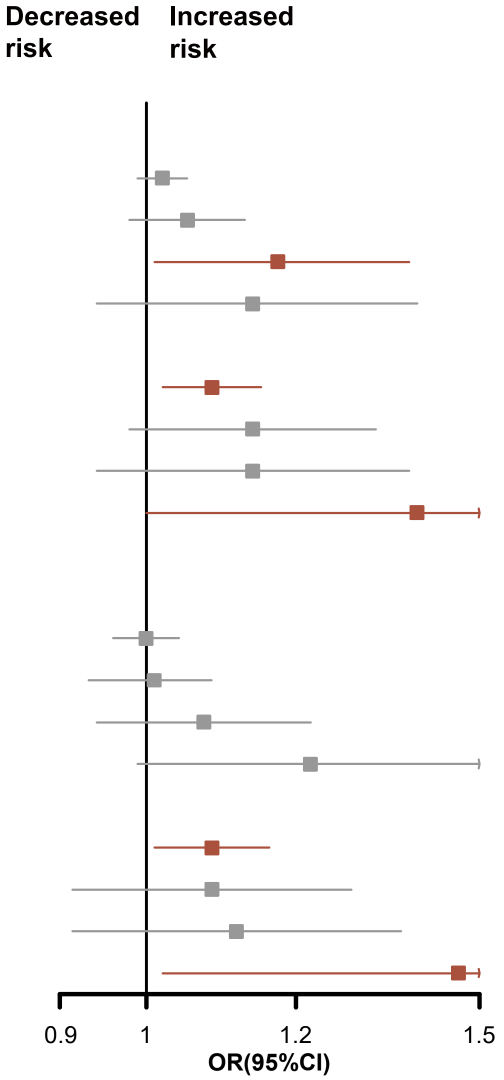

Model adjusted for age, sex, genetic ancestry, BMI, smoking status, alcohol intake status, Townsend deprivation index, diabetes, atherosclerotic heart disease, hypertensive diseases, atrial fibrillation and flutter, stroke, heart failure, peripheral artery and capillary disease, chronic renal failure, education level, sleep duration, physical activity, diet score, waist circumference, systolic blood pressure, diastolic blood pressure, total cholesterol, low-density lipoprotein cholesterol (LDL-C), high-density lipoprotein cholesterol (HDL-C), triglycerides, glucose glycated haemoglobin (HbA1c), estimated glomerular filtration rate (eGFR), lipid-lowering medication, anti-hypertensive medication, antidiabetic medication, cancer, liver disease, family history of diseases.

Supplementary Figure S13: Association of different CHIP status and MI in covariate model 7

CHIP status	OR (95% CI)	P value
**Any CHIP**		
overall	1.04 (0.91 − 1.18)	5.5×10−1
DNMT3A	1.03 (0.84 − 1.25)	8.0×10−1
TET2	1.13 (0.82 − 1.56)	4.6×10−1
ASXL1	1.08 (0.65 − 1.81)	7.6×10−1
**Large CHIP**		
overall	1.05 (0.85 − 1.28)	6.7×10−1
DNMT3A	1.16 (0.82 − 1.65)	3.9×10−1
TET2	1.43 (0.84 − 2.45)	1.9×10−1
ASXL1	1.31 (0.51 − 3.35)	5.8×10−1

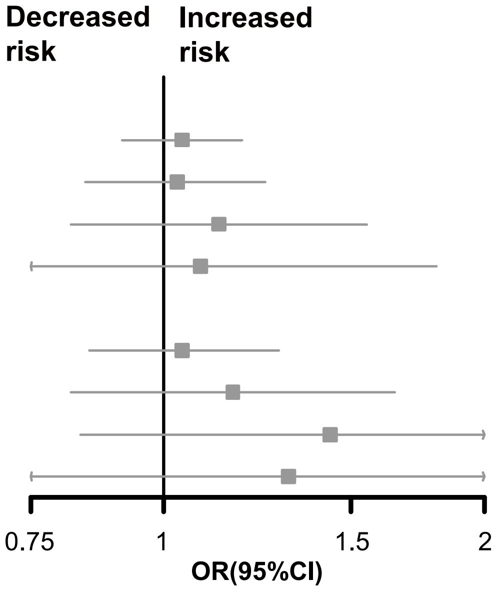

Model 7 adjusted for age, sex, genetic ancestry, BMI, smoking status, alcohol intake status, Townsend deprivation index, diabetes, atherosclerotic heart disease, hypertensive diseases, atrial fibrillation and flutter, stroke, heart failure, peripheral artery and capillary disease, chronic renal failure, education level, sleep duration, physical activity, diet score, waist circumference, systolic blood pressure, diastolic blood pressure, total cholesterol, low-density lipoprotein cholesterol (LDL-C), high-density lipoprotein cholesterol (HDL-C), triglycerides, glucose glycated haemoglobin (HbA1c), estimated glomerular filtration rate (eGFR), lipid-lowering medication, anti-hypertensive medication, antidiabetic medication, cancer, liver disease, family history of diseases.

Supplementary Figure S14: Association of JAK2 CHIP and ASCVD and MI in covariate model 7

CHIP status	OR (95% CI)	P value
**ASCVD**		
Any JAK2 CHIP	2.25 (1.42 − 3.55)	5.4×10−4
Large JAK2 CHIP	2.58 (1.45 − 4.59)	1.3×10−3
**MI**		
Any JAK2 CHIP	1.30 (0.66 − 2.58)	4.5×10−1
Large JAK2 CHIP	1.46 (0.60 − 3.52)	4.0×10−1

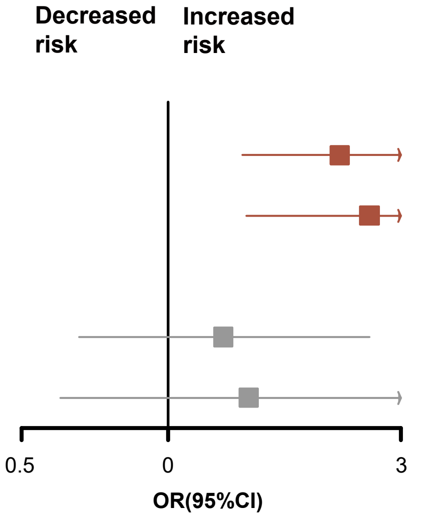

Model adjusted for age, sex, genetic ancestry, BMI, smoking status, alcohol intake status, Townsend deprivation index, diabetes, atherosclerotic heart disease, hypertensive diseases, atrial fibrillation and flutter, stroke, heart failure, peripheral artery and capillary disease, chronic renal failure, education level, sleep duration, physical activity, diet score, waist circumference, systolic blood pressure, diastolic blood pressure, total cholesterol, low-density lipoprotein cholesterol (LDL-C), high-density lipoprotein cholesterol (HDL-C), triglycerides, glucose glycated haemoglobin (HbA1c), estimated glomerular filtration rate (eGFR), lipid-lowering medication, anti-hypertensive medication, antidiabetic medication, cancer, liver disease, family history of diseases.

Supplementary Figure S15: Association of different CHIP status and AP in PSM models of 1:2 ratio

Angina pectoris type and CHIP status	OR (95% CI)	P value
**Angina pectoris**		
**Any CHIP**		
overall	0.94 (0.87 − 1.03)	1.9×10−1
DNMT3A	0.93 (0.82 − 1.06)	2.9×10−1
TET2	0.71 (0.58 − 0.88)	1.9×10−3
ASXL1	1.21 (0.82 − 1.77)	3.3×10−1
**Large CHIP**		
overall	0.71 (0.60 − 0.83)	2.3×10−5
DNMT3A	0.67 (0.51 − 0.88)	4.3×10−3
TET2	0.42 (0.26 − 0.68)	4.1×10−4
ASXL1	0.80 (0.41 − 1.57)	5.2×10−1
**Unstable angina pectoris**		
**Any CHIP**		
overall	0.83 (0.74 − 0.94)	4.0×10−3
DNMT3A	0.77 (0.64 − 0.94)	9.1×10−3
TET2	0.53 (0.39 − 0.73)	9.5×10−5
ASXL1	0.82 (0.49 − 1.38)	4.5×10−1
**Large CHIP**		
overall	0.58 (0.45 − 0.73)	7.5×10−6
DNMT3A	0.60 (0.41 − 0.87)	6.6×10−3
TET2	0.33 (0.16 − 0.69)	3.2×10−3
ASXL1	0.43 (0.15 − 1.27)	1.3×10−1
**Stable angina pectoris**		
**Any CHIP**		
overall	1.05 (0.79 − 1.40)	7.4×10−1
DNMT3A	1.51 (0.94 − 2.45)	9.1×10−2
TET2	1.06 (0.55 − 2.02)	8.7×10−1
ASXL1	2.74 (0.45 − 16.82)	2.8×10−1
**Large CHIP**		
overall	1.17 (0.68 − 2.01)	5.7×10−1
DNMT3A	1.44 (0.61 − 3.40)	4.0×10−1
TET2	0.66 (0.13 − 3.36)	6.1×10−1
ASXL1	0.55 (0.06 − 5.15)	6.0×10−1

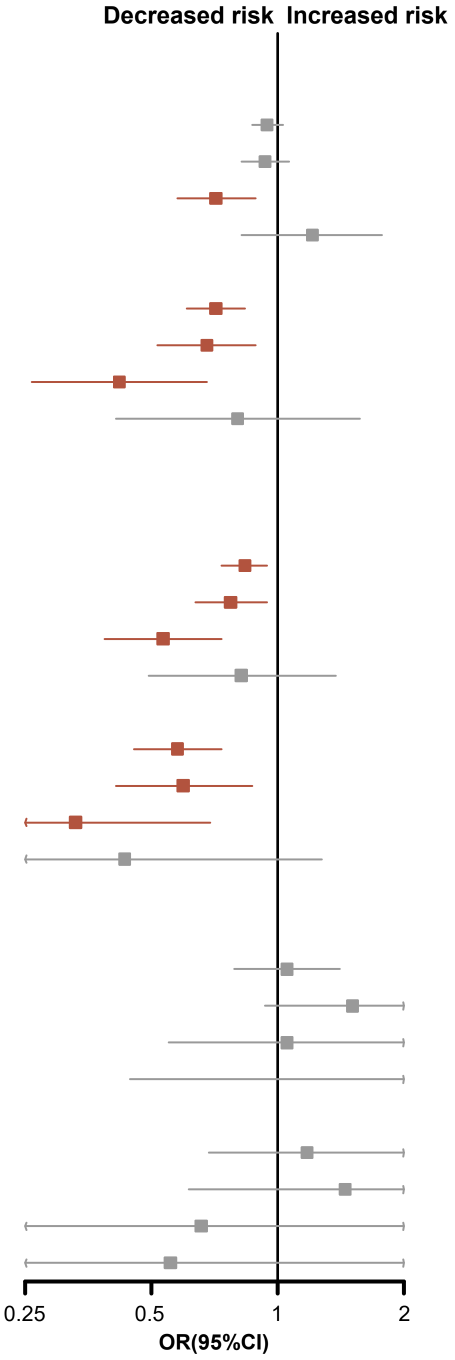

The model was adjusted by PSM, balancing age, sex, genetic ancestry, BMI, smoking status, alcohol intake status, Townsend deprivation index, diabetes, atherosclerotic heart disease, hypertensive diseases, atrial fibrillation and flutter, stroke, heart failure, peripheral artery and capillary disease, chronic renal failure.

Supplementary Figure S16: Association of different CHIP status and AP in PSM models of 1:3 ratio

Angina pectoris type and CHIP status	OR (95% CI)	P value
**Angina pectoris**		
**Any CHIP**		
overall	0.96 (0.89 − 1.04)	3.5×10−1
DNMT3A	0.96 (0.85 − 1.09)	5.4×10−1
TET2	0.79 (0.64 − 0.97)	2.3×10−2
ASXL1	1.28 (0.89 − 1.83)	1.8×10−1
**Large CHIP**		
overall	0.72 (0.62 − 0.84)	3.0×10−5
DNMT3A	0.70 (0.54 − 0.91)	6.9×10−3
TET2	0.44 (0.28 − 0.71)	5.8×10−4
ASXL1	0.84 (0.44 − 1.61)	6.0×10−1
**Unstable angina pectoris**		
**Any CHIP**		
overall	0.87 (0.78 − 0.99)	2.7×10−2
DNMT3A	0.84 (0.70 − 1.01)	6.1×10−2
TET2	0.63 (0.46 − 0.85)	3.1×10−3
ASXL1	0.92 (0.56 − 1.51)	7.5×10−1
**Large CHIP**		
overall	0.60 (0.48 − 0.76)	1.8×10−5
DNMT3A	0.66 (0.46 − 0.95)	2.7×10−2
TET2	0.36 (0.18 − 0.74)	5.3×10−3
ASXL1	0.43 (0.15 − 1.23)	1.2×10−1
**Stable angina pectoris**		
**Any CHIP**		
overall	1.09 (0.83 − 1.43)	5.3×10−1
DNMT3A	1.38 (0.89 − 2.15)	1.5×10−1
TET2	1.15 (0.62 − 2.14)	6.5×10−1
ASXL1	2.54 (0.49 − 13.16)	2.7×10−1
**Large CHIP**		
overall	1.12 (0.68 − 1.86)	6.5×10−1
DNMT3A	1.46 (0.65 − 3.23)	3.6×10−1
TET2	0.54 (0.12 − 2.45)	4.2×10−1
ASXL1	0.71 (0.08 − 6.33)	7.6×10−1

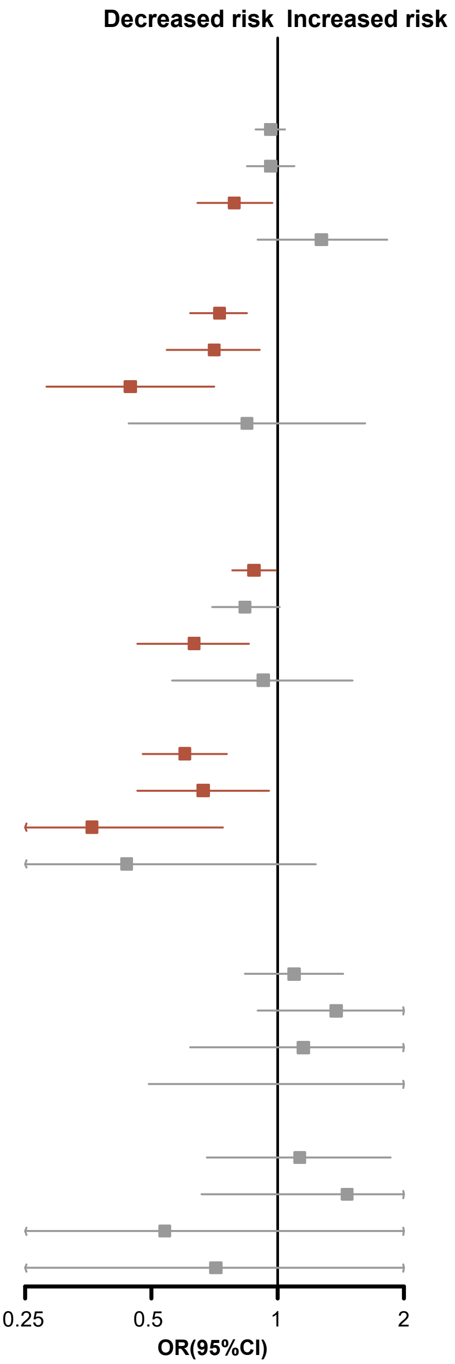

The model was adjusted by PSM, balancing age, sex, genetic ancestry, BMI, smoking status, alcohol intake status, Townsend deprivation index, diabetes, atherosclerotic heart disease, hypertensive diseases, atrial fibrillation and flutter, stroke, heart failure, peripheral artery and capillary disease, chronic renal failure.

Supplementary Figure S17: Association of different CHIP status and AP in PSM models of 1:4 ratio

Angina pectoris type and CHIP status	OR (95% CI)	P value
**Angina pectoris**		
**Any CHIP**		
overall	0.99 (0.91 − 1.07)	7.8×10−1
DNMT3A	0.93 (0.83 − 1.06)	2.8×10−1
TET2	0.80 (0.66 − 0.98)	3.1×10−2
ASXL1	1.25 (0.89 − 1.76)	2.0×10−1
**Large CHIP**		
overall	0.74 (0.64 − 0.86)	7.0×10−5
DNMT3A	0.72 (0.56 − 0.92)	8.8×10−3
TET2	0.46 (0.29 − 0.72)	7.7×10−4
ASXL1	0.84 (0.45 − 1.57)	5.8×10−1
**Unstable angina pectoris**		
**Any CHIP**		
overall	0.91 (0.81 − 1.02)	9.9×10−2
DNMT3A	0.84 (0.71 − 1.01)	6.3×10−2
TET2	0.66 (0.49 − 0.89)	7.0×10−3
ASXL1	0.91 (0.57 − 1.47)	7.1×10−1
**Large CHIP**		
overall	0.61 (0.49 − 0.77)	2.3×10−5
DNMT3A	0.72 (0.51 − 1.03)	7.2×10−2
TET2	0.36 (0.18 − 0.72)	4.3×10−3
ASXL1	0.46 (0.17 − 1.29)	1.4×10−1
**Stable angina pectoris**		
**Any CHIP**		
overall	1.18 (0.90 − 1.53)	2.3×10−1
DNMT3A	1.25 (0.83 − 1.90)	2.9×10−1
TET2	1.33 (0.73 − 2.44)	3.6×10−1
ASXL1	1.06 (0.29 − 3.93)	9.3×10−1
**Large CHIP**		
overall	1.13 (0.69 − 1.83)	6.3×10−1
DNMT3A	1.35 (0.63 − 2.87)	4.4×10−1
TET2	0.61 (0.14 − 2.72)	5.2×10−1
ASXL1	0.81 (0.09 − 7.10)	8.5×10−1

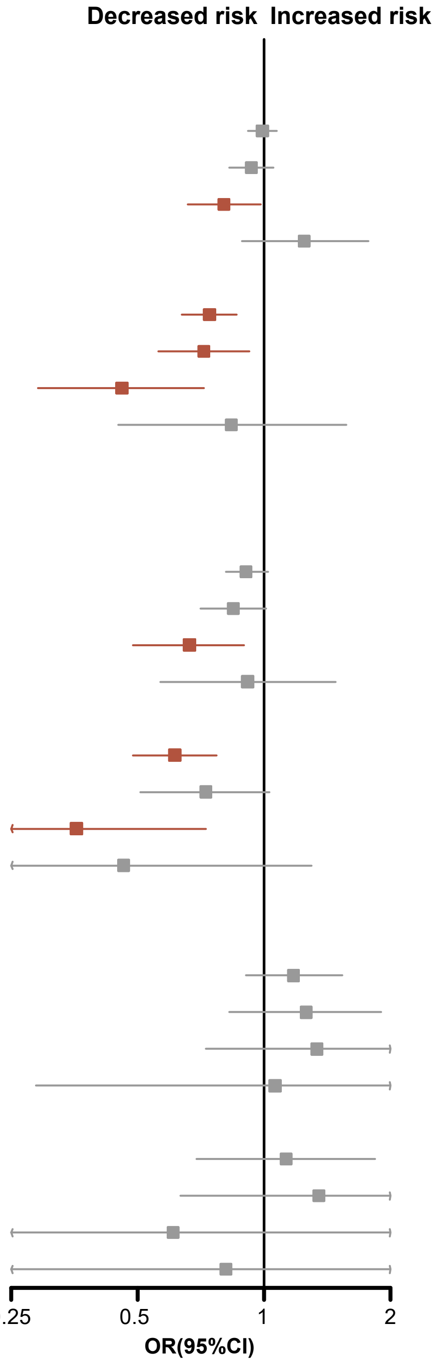

The model was adjusted by PSM, balancing age, sex, genetic ancestry, BMI, smoking status, alcohol intake status, Townsend deprivation index, diabetes, atherosclerotic heart disease, hypertensive diseases, atrial fibrillation and flutter, stroke, heart failure, peripheral artery and capillary disease, chronic renal failure.

Supplementary Figure S18: Association of different CHIP status and AP in PSM models of 1:5 ratio

Angina pectoris type and CHIP status	OR (95% CI)	P value
**Angina pectoris**		
**Any CHIP**		
overall	1.00 (0.93 − 1.08)	1
DNMT3A	0.95 (0.85 − 1.08)	4.4×10−1
TET2	0.79 (0.65 − 0.96)	1.6×10−2
ASXL1	1.16 (0.83 − 1.62)	3.8×10−1
**Large CHIP**		
overall	0.76 (0.66 − 0.88)	2.7×10−4
DNMT3A	0.77 (0.60 − 0.98)	3.5×10−2
TET2	0.49 (0.31 − 0.76)	1.7×10−3
ASXL1	0.79 (0.43 − 1.45)	4.5×10−1
**Unstable angina pectoris**		
**Any CHIP**		
overall	0.92 (0.82 − 1.03)	1.7×10−1
DNMT3A	0.86 (0.72 − 1.02)	8.4×10−2
TET2	0.66 (0.49 − 0.89)	6.7×10−3
ASXL1	0.92 (0.58 − 1.47)	7.4×10−1
**Large CHIP**		
overall	0.63 (0.51 − 0.79)	6.3×10−5
DNMT3A	0.78 (0.55 − 1.10)	1.6×10−1
TET2	0.39 (0.20 − 0.80)	9.4×10−3
ASXL1	0.50 (0.18 − 1.35)	1.7×10−1
**Stable angina pectoris**		
**Any CHIP**		
overall	1.24 (0.95 − 1.61)	1.1×10−1
DNMT3A	1.36 (0.90 − 2.05)	1.5×10−1
TET2	1.29 (0.71 − 2.32)	4.0×10−1
ASXL1	0.83 (0.24 − 2.88)	7.6×10−1
**Large CHIP**		
overall	1.18 (0.73 − 1.89)	5.1×10−1
DNMT3A	1.37 (0.66 − 2.88)	4.0×10−1
TET2	0.69 (0.15 − 3.05)	6.2×10−1
ASXL1	0.58 (0.07 − 4.77)	6.1×10−1

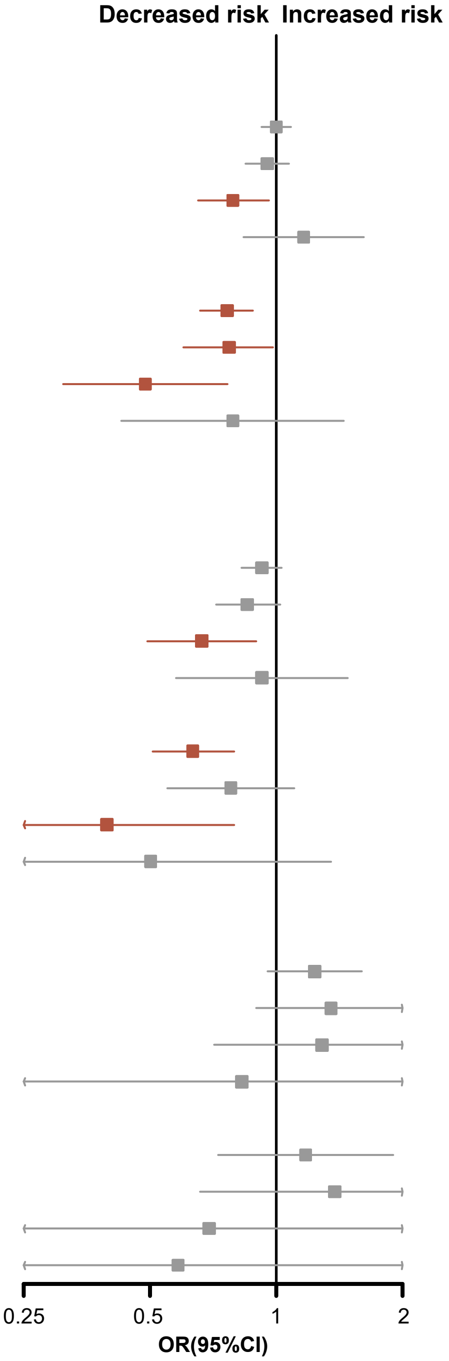

The model was adjusted by PSM, balancing age, sex, genetic ancestry, BMI, smoking status, alcohol intake status, Townsend deprivation index, diabetes, atherosclerotic heart disease, hypertensive diseases, atrial fibrillation and flutter, stroke, heart failure, peripheral artery and capillary disease, chronic renal failure.

Supplementary Figure S19: Association of different CHIP status and AP in PSM models of 1:1 ratio in White population

Angina pectoris type and CHIP status	OR (95% CI)	P value
**Angina pectoris**		
**Any CHIP**		
overall	0.91 (0.83 − 1.00)	6.0×10−2
DNMT3A	0.89 (0.76 − 1.04)	1.4×10−1
TET2	0.67 (0.53 − 0.85)	1.0×10−3
ASXL1	1.34 (0.84 − 2.13)	2.2×10−1
**Large CHIP**		
overall	0.76 (0.63 − 0.91)	2.6×10−3
DNMT3A	0.64 (0.47 − 0.87)	3.8×10−3
TET2	0.47 (0.27 − 0.83)	8.7×10−3
ASXL1	0.74 (0.35 − 1.60)	4.5×10−1
**Unstable angina pectoris**		
**Any CHIP**		
overall	0.85 (0.73 − 0.98)	2.2×10−2
DNMT3A	0.82 (0.66 − 1.03)	8.7×10−2
TET2	0.61 (0.43 − 0.87)	6.7×10−3
ASXL1	1.03 (0.55 − 1.93)	9.3×10−1
**Large CHIP**		
overall	0.59 (0.45 − 0.77)	1.2×10−4
DNMT3A	0.63 (0.41 − 0.96)	3.0×10−2
TET2	0.34 (0.15 − 0.78)	1.0×10−2
ASXL1	0.34 (0.11 − 1.07)	6.6×10−2
**Stable angina pectoris**		
**Any CHIP**		
overall	1.30 (0.91 − 1.84)	1.5×10−1
DNMT3A	1.00 (0.60 − 1.67)	1
TET2	1.39 (0.63 − 3.09)	4.1×10−1
ASXL1	1.23 (0.88 − 1.71)	2.3×10−1
**Large CHIP**		
overall	3.90 (0.39 − 39.54)	2.5×10−1
DNMT3A	4.64 (0.26 − 84.26)	3.0×10−1
TET2	1.55 (1.08 − 2.22)	1.9×10−2
ASXL1	1.26 (0.91 − 1.75)	1.7×10−1

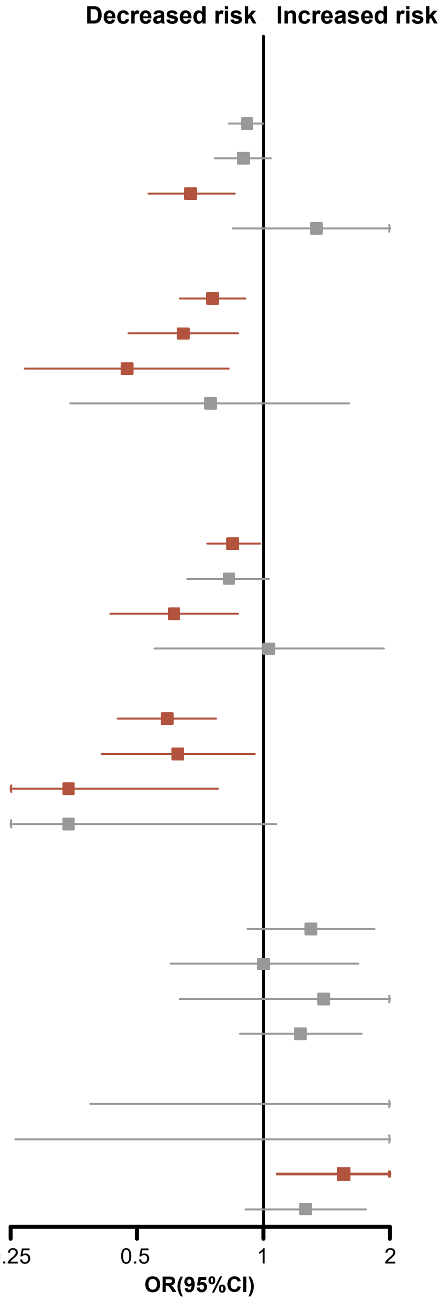

The model was adjusted by PSM, balancing age, sex, BMI, smoking status, alcohol intake status, Townsend deprivation index, diabetes, atherosclerotic heart disease, hypertensive diseases, atrial fibrillation and flutter, stroke, heart failure, peripheral artery and capillary disease, chronic renal failure.

Supplementary Figure S20: Association of different CHIP status and AP in PSM models of 1:1 ratio in Asian population

Angina pectoris type and CHIP status	OR (95% CI)	P value
**Angina pectoris**		
**Any CHIP**		
overall	0.53 (0.34 − 0.81)	3.6×10−3
DNMT3A	0.62 (0.25 − 1.53)	3.0×10−1
TET2	0.41 (0.25 − 0.67)	4.1×10−4
ASXL1	0.02 (0.00 − 1.66)	8.1×10−2
**Large CHIP**		
overall	0.42 (0.21 − 0.83)	1.3×10−2
DNMT3A	0.18 (0.02 − 2.16)	1.8×10−1
TET2	0.26 (0.14 − 0.47)	7.0×10−6
ASXL1	0.51 (0.02 − 14.52)	6.9×10−1
**Unstable angina pectoris**		
**Any CHIP**		
overall	0.34 (0.20 − 0.60)	1.5×10−4
DNMT3A	0.50 (0.31 − 0.79)	3.3×10−3
TET2	0.38 (0.20 − 0.70)	1.9×10−3
ASXL1	0.66 (0.01 − 36.99)	8.4×10−1
**Large CHIP**		
overall	0.25 (0.11 − 0.57)	8.5×10−4
DNMT3A	0.26 (0.05 − 1.20)	8.5×10−2
TET2	0.25 (0.12 − 0.54)	4.3×10−4
ASXL1	0 (0 − Inf)	1
**Stable angina pectoris**		
**Any CHIP**		
overall	1.22 (0.22 − 6.82)	8.2×10−1
DNMT3A	1.26 (0.15 − 10.42)	8.3×10−1
TET2	1.07 (0.04 − 29.38)	9.7×10−1
ASXL1	1.49 (0.07 − 30.54)	8.0×10−1
**Large CHIP**		
overall	2.55 (0.16 − 41.41)	5.1×10−1
DNMT3A	2.82 (0.23 − 34.38)	4.2×10−1
TET2	1.26 (0.60 − 2.65)	5.3×10−1
ASXL1	Inf (0 − Inf)	1

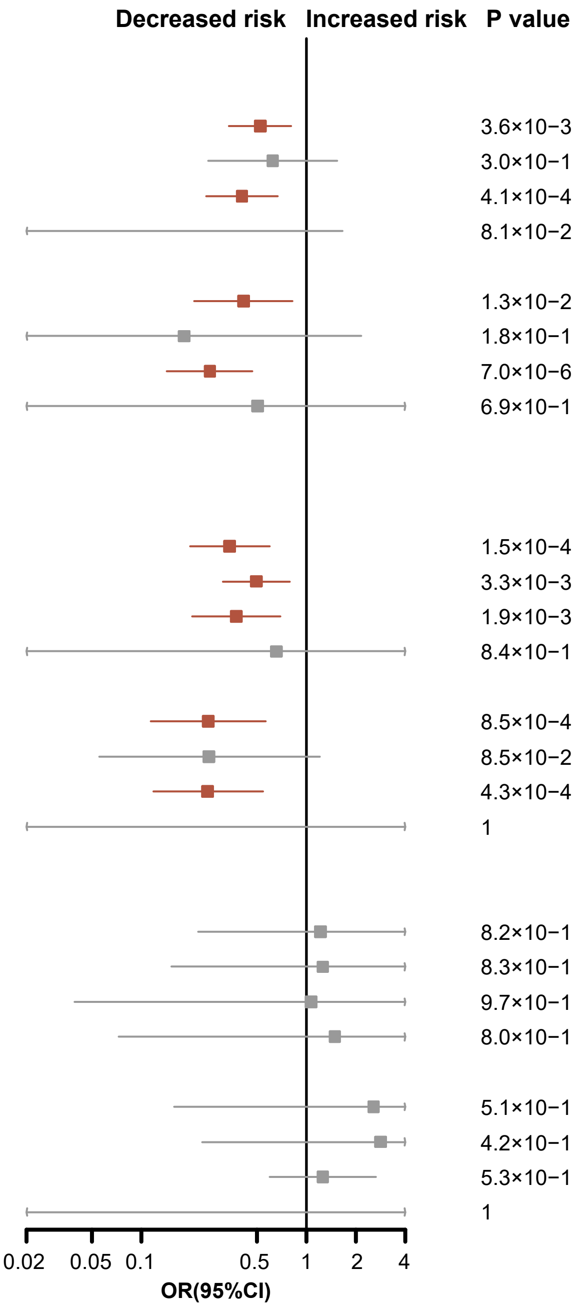

The model was adjusted by PSM, balancing age, sex, BMI, smoking status, alcohol intake status, Townsend deprivation index, diabetes, atherosclerotic heart disease, hypertensive diseases, atrial fibrillation and flutter, stroke, heart failure, peripheral artery and capillary disease, chronic renal failure.

Supplementary Figure S21: Association of different CHIP status and AP in PSM models of 1:1 ratio in Black population

Angina pectoris type and CHIP status	OR (95% CI)	P value
**Angina pectoris**		
**Any CHIP**		
overall	0.76 (0.39 − 1.49)	4.3×10−1
DNMT3A	0.38 (0.11 − 1.38)	1.4×10−1
TET2	0.31 (0.09 − 1.11)	7.1×10−2
ASXL1	2.04 (0.34 − 12.17)	4.3×10−1
**Large CHIP**		
overall	0.24 (0.06 − 0.96)	4.3×10−2
DNMT3A	0.36 (0.06 − 2.03)	2.5×10−1
TET2	0.26 (0.14 − 0.47)	7.5×10−6
ASXL1	0.56 (0.25 − 1.23)	1.5×10−1
**Unstable angina pectoris**		
**Any CHIP**		
overall	0.28 (0.06 − 1.27)	9.9×10−2
DNMT3A	0.57 (0.34 − 0.96)	3.3×10−2
TET2	0.54 (0.29 − 1.01)	5.5×10−2
ASXL1	0.78 (0.04 − 16.49)	8.8×10−1
**Large CHIP**		
overall	0.27 (0.15 − 0.48)	9.7×10−6
DNMT3A	0.47 (0.08 − 2.80)	4.0×10−1
TET2	0.22 (0.10 − 0.52)	5.0×10−4
ASXL1	0 (0 − Inf)	1
**Stable angina pectoris**		
**Any CHIP**		
overall	1.04 (0.06 − 16.81)	9.8×10−1
DNMT3A	1.31 (0.23 − 7.55)	7.6×10−1
TET2	1.18 (0.65 − 2.13)	5.8×10−1
ASXL1	2.30 (0.13 − 39.93)	5.7×10−1
**Large CHIP**		
overall	1.26 (0.6 − 2.64)	5.4×10−1
DNMT3A	1.89 (0.14 − 26.09)	6.3×10−1
TET2	1.51 (0.32 − 7.10)	6.0×10−1
ASXL1	Inf (0 − Inf)	1

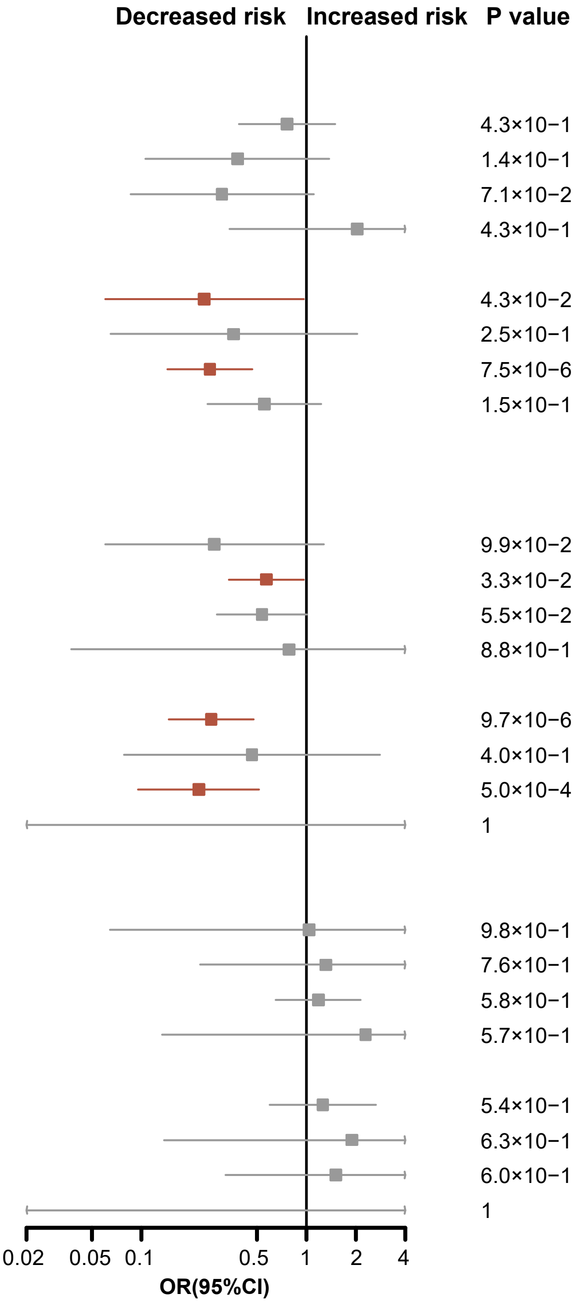

The model was adjusted by PSM, balancing age, sex, BMI, smoking status, alcohol intake status, Townsend deprivation index, diabetes, atherosclerotic heart disease, hypertensive diseases, atrial fibrillation and flutter, stroke, heart failure, peripheral artery and capillary disease, chronic renal failure.

Supplementary Figure S22: Association between VAF and AP based on the model fully adjusted for covariates

Angina pectoris type and CHIP status	OR (95% CI)	P value
**Angina pectoris**		
overall	0.43 (0.23 − 0.84)	1.2×10−2
DNMT3A	0.56 (0.18 − 1.79)	3.3×10−1
TET2	0.08 (0.01 − 0.64)	1.7×10−2
ASXL1	1.75 (0.12 − 25.53)	6.8×10−1
**Unstable angina pectoris**		
overall	0.24 (0.09 − 0.66)	5.3×10−3
DNMT3A	0.46 (0.09 − 2.52)	3.7×10−1
TET2	0.06 (0.00 − 1.40)	8.1×10−2
ASXL1	0.69 (0.01 − 33.99)	8.5×10−1
**Stable angina pectoris**		
overall	1.67 (0.20 − 13.69)	6.3×10−1
DNMT3A	7.09 (0.25 − 203.86)	2.5×10−1
TET2	7.59 (0.04 − 1403.25)	4.5×10−1
ASXL1	2.61 (0.00 − 18813.12)	8.3×10−1

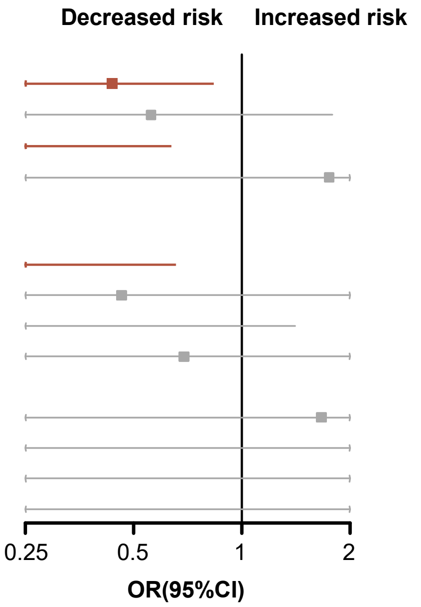

Model adjusted for age at recruitment, sex, genetic ancestry, BMI, ever smoked, alcohol intake frequency, Townsend deprivation index, diabetes, atherosclerotic heart disease, hypertensive diseases, atrial fibrillation and flutter, stroke, heart failure, peripheral artery and capillary disease, chronic renal failure. Abbreviations: BMI, body mass index.

Supplementary Figure S23: RCS curve for associations between VAF and AP

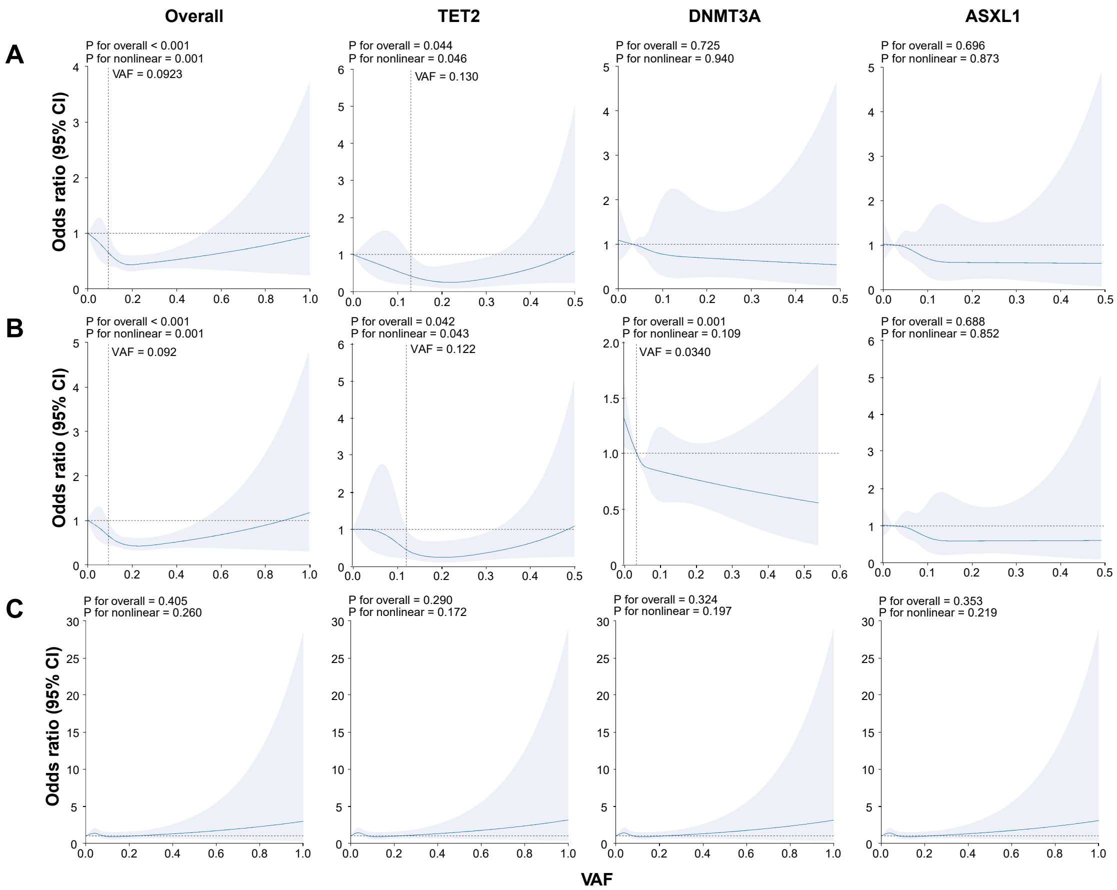

(A)Angina pectoris; (B)Unstable angina pectoris; (C)Stable angina pectoris.

Supplementary Figure S24: Forest plot of Mendelian randomization analysis for causal association of CHIP on AP

CHIP status and MR methods	OR (95% CI)	P value
**Overall**		
Any CHIP		
MR Egger	0.63 (0.38 − 1.03)	9.2×10−2
Weighted median	0.80 (0.66 − 0.98)	3.0×10−2
Inverse variance weighted	0.86 (0.72 − 1.03)	9.2×10−2
Simple mode	0.93 (0.70 − 1.24)	6.3×10−1
Weighted mode	0.80 (0.65 − 0.99)	3.9×10−2
Large CHIP		
MR Egger	1.28 (0.79 − 2.10)	3.4×10−1
Weighted median	0.94 (0.79 − 1.11)	4.6×10−1
Inverse variance weighted	1.06 (0.91 − 1.23)	4.6×10−1
Simple mode	0.85 (0.57 − 1.27)	4.4×10−1
Weighted mode	0.84 (0.58 − 1.23)	3.9×10−1
DNMT3A		
Any CHIP		
MR Egger	0.76 (0.61 − 0.95)	2.4×10−2
Weighted median	0.92 (0.83 − 1.01)	7.5×10−2
Inverse variance weighted	0.96 (0.89 − 1.04)	3.2×10−1
Simple mode	0.96 (0.80 − 1.17)	7.2×10−1
Weighted mode	0.91 (0.81 − 1.02)	1.1×10−1
Large CHIP		
MR Egger	1.03 (0.79 − 1.33)	8.5×10−1
Weighted median	0.94 (0.86 − 1.02)	1.6×10−1
Inverse variance weighted	0.98 (0.92 − 1.05)	6.2×10−1
Simple mode	0.91 (0.76 − 1.09)	3.5×10−1
Weighted mode	0.90 (0.79 − 1.02)	1.4×10−1
TET2		
Any CHIP		
MR Egger	1.30 (0.84 − 2.00)	3.0×10−1
Weighted median	0.90 (0.77 − 1.06)	2.1×10−1
Inverse variance weighted	0.96 (0.81 − 1.13)	6.3×10−1
Simple mode	0.87 (0.69 − 1.10)	2.9×10−1
Weighted mode	0.87 (0.73 − 1.05)	2.0×10−1
Large CHIP		
MR Egger	0.95 (0.82 − 1.10)	5.3×10−1
Weighted median	0.97 (0.91 − 1.03)	3.5×10−1
Inverse variance weighted	0.96 (0.91 − 1.01)	1.4×10−1
Simple mode	1.01 (0.91 − 1.13)	8.3×10−1
Weighted mode	0.96 (0.87 − 1.05)	3.8×10−1
ASXL1		
Any CHIP		
MR Egger	0.99 (0.87 − 1.14)	9.4×10−1
Weighted median	0.98 (0.92 − 1.05)	5.1×10−1
Inverse variance weighted	1.00 (0.95 − 1.05)	8.9×10−1
Simple mode	0.98 (0.87 − 1.11)	7.8×10−1
Weighted mode	0.98 (0.88 − 1.09)	7.6×10−1
Large CHIP		
MR Egger	0.99 (0.91 − 1.08)	8.7×10−1
Weighted median	1.01 (0.97 − 1.04)	7.5×10−1
Inverse variance weighted	1.00 (0.97 − 1.03)	9.8×10−1
Simple mode	1.01 (0.96 − 1.07)	6.5×10−1
Weighted mode	1.01 (0.96 − 1.06)	6.7×10−1

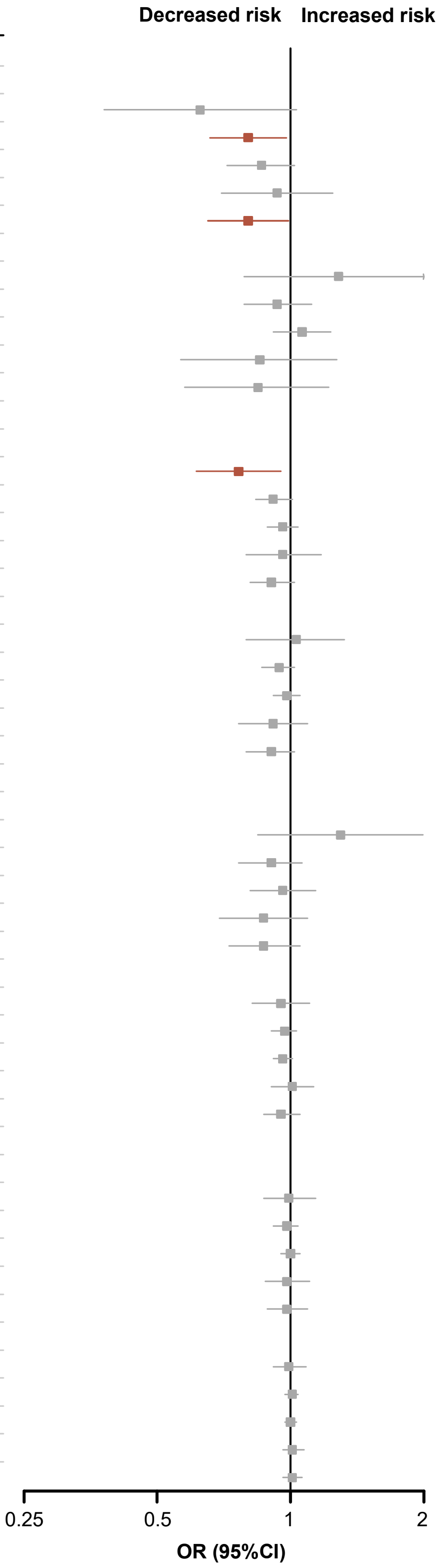

Point estimate boxes and error bars in red indicated significant effects, while grey ones meant non-significant.

Supplementary Figure S25: Forest plot of Mendelian randomization analysis of more methods for causal association of large *TET2* CHIP on UAP

MR methods	OR (95% CI)	P value
Maximum likelihood	0.90 (0.83 − 0.96)	2.7×10−3
MR Egger	0.99 (0.76 − 1.30)	9.6×10−1
Simple median	0.89 (0.78 − 1.01)	6.5×10−2
Weighted median	0.83 (0.75 − 0.93)	1.4×10−3
Penalised weighted median	0.82 (0.74 − 0.92)	3.9×10−4
Inverse variance weighted	0.90 (0.82 − 1.00)	4.3×10−2
IVW radial	0.90 (0.82 − 1.00)	4.3×10−2
Inverse variance weighted (multiplicative random effects)	0.90 (0.82 − 1.00)	4.3×10−2
Inverse variance weighted (fixed effects)	0.90 (0.84 − 0.97)	3.7×10−3
Simple mode	0.81 (0.65 − 1.00)	4.9×10−2
Weighted mode	0.80 (0.68 − 0.95)	2.8×10−2

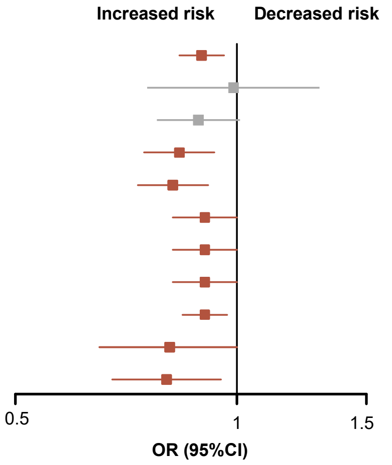

Point estimate boxes and error bars in red indicated significant effects, while grey ones meant non-significant. Abbreviation: IVW, inverse variance weighted.

Supplementary Figure S26: Forest plot of Mendelian randomization analysis for causal association of CHIP on SAP

CHIP status and MR methods	OR (95% CI)	P value
**Overall**		
Any CHIP		
MR Egger	1.90 (0.31 − 11.56)	5.0×10^{-1}
Weighted median	1.73 (0.83 − 3.62)	1.4×10^{-1}
Inverse variance weighted	1.40 (0.77 − 2.55)	2.7×10^{-1}
Simple mode	2.54 (0.73 − 8.84)	1.7×10^{-1}
Weighted mode	2.30 (0.97 − 5.43)	8.1×10^{-2}
Large CHIP		
MR Egger	0.56 (0.15 − 2.10)	4.1×10^{-1}
Weighted median	0.80 (0.48 − 1.34)	4.0×10^{-1}
Inverse variance weighted	0.91 (0.62 − 1.33)	6.2×10^{-1}
Simple mode	0.69 (0.28 − 1.71)	4.4×10^{-1}
Weighted mode	0.72 (0.32 − 1.60)	4.3×10^{-1}
DNMT3A		
Any CHIP		
MR Egger	1.29 (0.58 − 2.85)	5.4×10^{-1}
Weighted median	1.25 (0.88 − 1.77)	2.2×10^{-1}
Inverse variance weighted	1.17 (0.90 − 1.52)	2.4×10^{-1}
Simple mode	1.67 (0.83 − 3.35)	1.6×10^{-1}
Weighted mode	1.47 (0.99 − 2.20)	6.7×10^{-2}
Large CHIP		
MR Egger	1.46 (0.68 − 3.14)	3.5×10^{-1}
Weighted median	1.19 (0.89 − 1.60)	2.4×10^{-1}
Inverse variance weighted	1.15 (0.93 − 1.43)	1.9×10^{-1}
Simple mode	1.15 (0.75 − 1.76)	5.4×10^{-1}
Weighted mode	1.16 (0.77 − 1.76)	4.9×10^{-1}
TET2		
Any CHIP		
MR Egger	1.59 (0.40 − 6.32)	5.4×10^{-1}
Weighted median	1.37 (0.79 − 2.39)	2.7×10^{-1}
Inverse variance weighted	1.41 (0.91 − 2.19)	1.2×10^{-1}
Simple mode	1.37 (0.64 − 2.91)	4.5×10^{-1}
Weighted mode	1.37 (0.69 − 2.72)	4.1×10^{-1}
Large CHIP		
MR Egger	1.49 (0.93 − 2.39)	1.3×10^{-1}
Weighted median	1.16 (0.92 − 1.47)	2.1×10^{-1}
Inverse variance weighted	1.10 (0.90 − 1.35)	3.4×10^{-1}
Simple mode	1.18 (0.81 − 1.74)	4.0×10^{-1}
Weighted mode	1.18 (0.86 − 1.61)	3.3×10^{-1}
ASXL1		
Any CHIP		
MR Egger	0.87 (0.54 − 1.42)	6.0×10^{-1}
Weighted median	0.99 (0.77 − 1.27)	9.3×10^{-1}
Inverse variance weighted	0.97 (0.80 − 1.16)	7.2×10^{-1}
Simple mode	1.08 (0.71 − 1.65)	7.3×10^{-1}
Weighted mode	1.13 (0.77 − 1.66)	5.3×10^{-1}
Large CHIP		
MR Egger	1.28 (0.96 − 1.69)	1.1×10^{-1}
Weighted median	1.04 (0.90 − 1.19)	6.0×10^{-1}
Inverse variance weighted	1.02 (0.92 − 1.14)	6.7×10^{-1}
Simple mode	1.07 (0.86 − 1.32)	5.6×10^{-1}
Weighted mode	1.06 (0.88 − 1.28)	5.5×10^{-1}

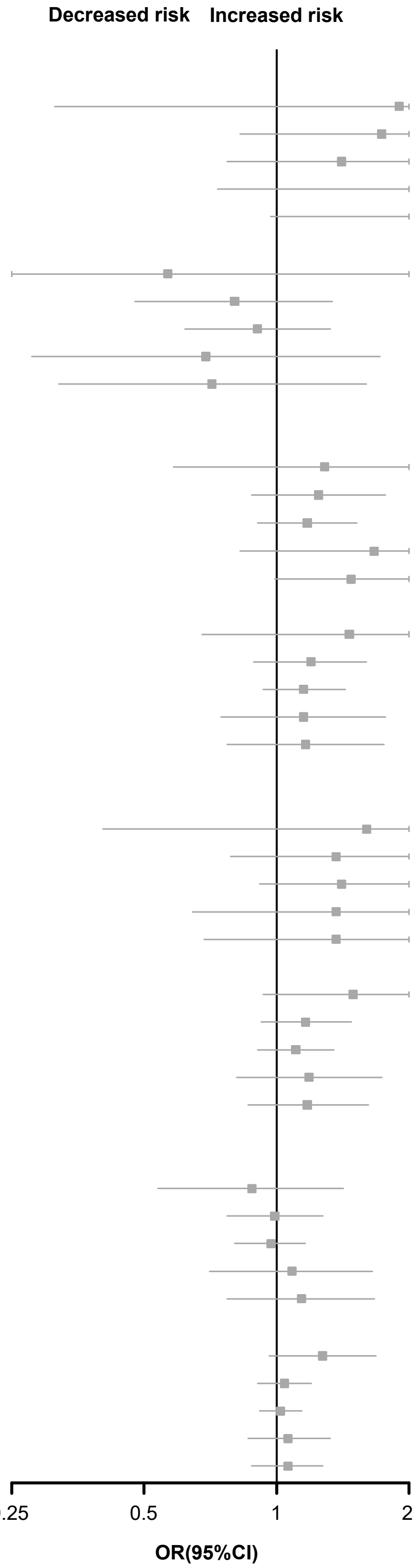

Point estimate boxes and error bars in red indicated significant effects, while grey ones meant non-significant.

Supplementary Figure S27: Forest plot of Mendelian randomization analysis for causal association of CHIP on AP after excluding IVs relevant to AP risk factors

CHIP status and MR methods	OR (95% CI)	P value
**Overall**		
Any CHIP		
MR Egger	0.63 (0.36 − 1.10)	1.4×10−1
Weighted median	0.79 (0.64 − 0.97)	2.3×10−2
Inverse variance weighted	0.85 (0.69 − 1.04)	1.1×10−1
Simple mode	0.87 (0.66 − 1.15)	3.5×10−1
Weighted mode	0.80 (0.64 − 0.99)	4.4×10−2
Large CHIP		
MR Egger	1.28 (0.74 − 2.19)	4.0×10−1
Weighted median	0.99 (0.84 − 1.17)	9.4×10−1
Inverse variance weighted	1.07 (0.91 − 1.25)	4.4×10−1
Simple mode	0.83 (0.54 − 1.29)	4.3×10−1
Weighted mode	0.84 (0.59 − 1.18)	3.4×10−1
DNMT3A		
Any CHIP		
MR Egger	0.78 (0.62 − 0.99)	4.2×10−2
Weighted median	0.88 (0.79 − 0.98)	2.5×10−2
Inverse variance weighted	0.95 (0.87 − 1.03)	2.3×10−1
Simple mode	0.97 (0.80 − 1.17)	7.3×10−1
Weighted mode	0.90 (0.80 − 1.01)	9.0×10−2

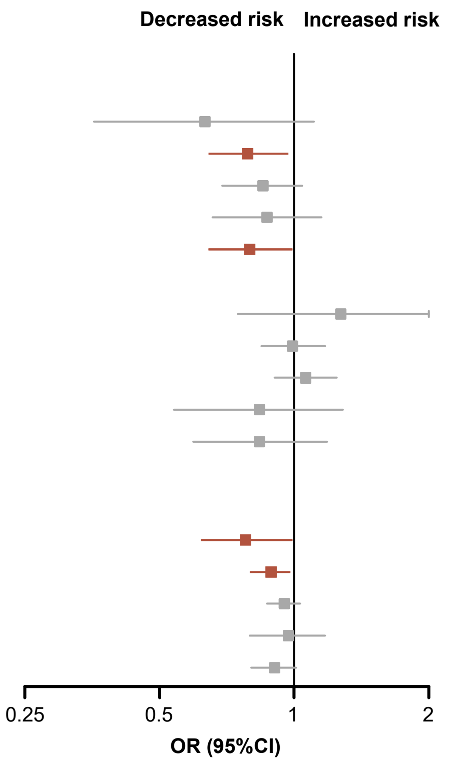

Point estimate boxes and error bars in red indicated significant effects, while grey ones meant non-significant. Mendelian randomization analysis between other CHIP status and AP were not changed since no variants were excluded.

Supplementary Figure S28: Forest plot of Mendelian randomization analysis for causal association of CHIP on UAP after excluding IVs relevant to AP risk factors

CHIP status and MR methods	OR (95% CI)	P value
**Overall**		
Any CHIP		
MR Egger	0.46 (0.22 − 0.97)	4.2×10−2
Weighted median	0.68 (0.49 − 0.94)	1.8×10−2
Inverse variance weighted	0.70 (0.53 − 0.91)	8.7×10−3
Simple mode	0.88 (0.48 − 1.62)	6.9×10−1
Weighted mode	0.53 (0.37 − 0.77)	6.5×10−3
Large CHIP		
MR Egger	0.75 (0.32 − 1.73)	5.1×10−1
Weighted median	1.21 (0.95 − 1.53)	1.2×10−1
Inverse variance weighted	0.98 (0.77 − 1.25)	8.8×10−1
Simple mode	1.26 (0.87 − 1.81)	2.5×10−1
Weighted mode	1.24 (0.85 − 1.80)	2.9×10−1
DNMT3A		
Any CHIP		
MR Egger	0.53 (0.38 − 0.75)	1.6×10−3
Weighted median	0.89 (0.76 − 1.05)	1.7×10−1
Inverse variance weighted	0.93 (0.81 − 1.07)	3.2×10−1
Simple mode	1.00 (0.69 − 1.46)	9.9×10−1
Weighted mode	0.76 (0.64 − 0.90)	4.7×10−3

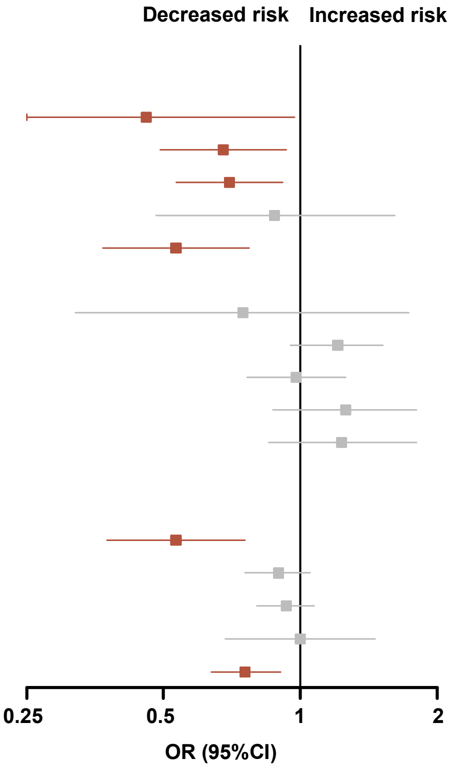

Point estimate boxes and error bars in red indicated significant effects, while grey ones meant non-significant. Mendelian randomization analysis between other CHIP status and AP were not changed since no variants were excluded.

Supplementary Figure S29: Forest plot of Mendelian randomization analysis for causal association of CHIP on SAP after excluding IVs relevant to AP risk factors

CHIP status and MR methods	OR (95% CI)	P value
**Overall**		
Any CHIP		
MR Egger	2.76 (0.49 − 15.43)	2.7×10−1
Weighted median	1.57 (0.73 − 3.37)	2.4×10−1
Inverse variance weighted	1.19 (0.64 − 2.18)	5.9×10−1
Simple mode	2.24 (0.56 − 8.86)	2.8×10−1
Weighted mode	2.17 (0.89 − 5.31)	1.2×10−1
Large CHIP		
MR Egger	0.75 (0.19 − 2.98)	6.9×10−1
Weighted median	0.78 (0.47 − 1.31)	3.5×10−1
Inverse variance weighted	0.84 (0.57 − 1.25)	4.0×10−1
Simple mode	0.70 (0.30 − 1.64)	4.3×10−1
Weighted mode	0.68 (0.29 − 1.59)	3.9×10−1
DNMT3A		
Any CHIP		
MR Egger	1.18 (0.54 − 2.59)	6.9×10−1
Weighted median	1.25 (0.85 − 1.82)	2.6×10−1
Inverse variance weighted	1.15 (0.88 − 1.51)	3.1×10−1
Simple mode	1.40 (0.73 − 2.67)	3.2×10−1
Weighted mode	1.42 (0.93 − 2.17)	1.2×10−1

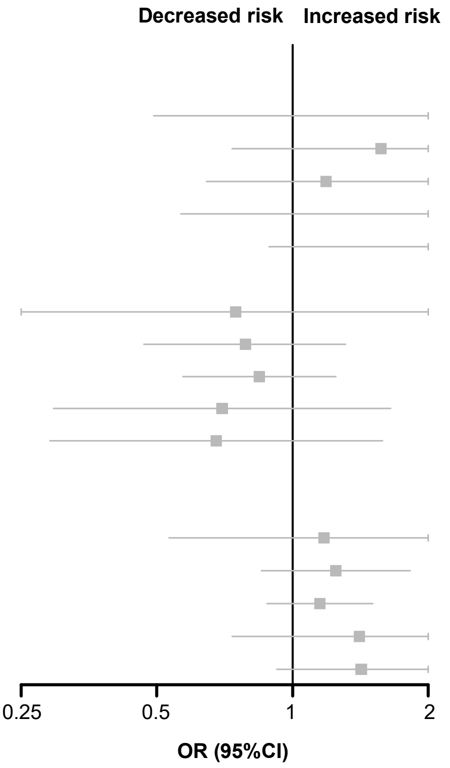

Point estimate boxes and error bars in red indicated significant effects, while grey ones meant non-significant. Mendelian randomization analysis between other CHIP status and AP were not changed since no variants were excluded.

Supplementary Figure S30: Scatter plots of Mendelian randomization analysis for causal association of CHIP on AP

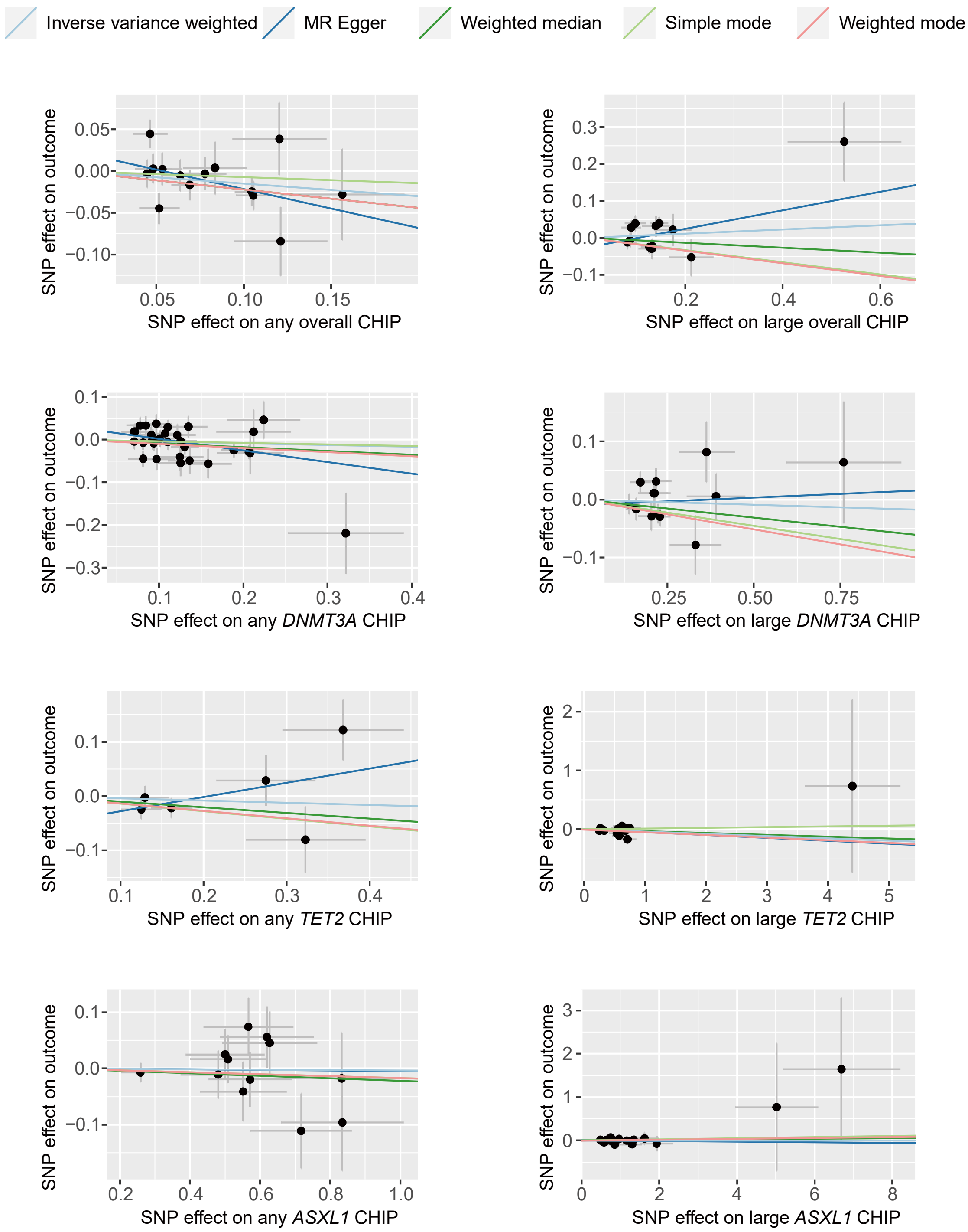

Supplementary Figure S31: Scatter plots of Mendelian randomization analysis for causal association of CHIP on UAP

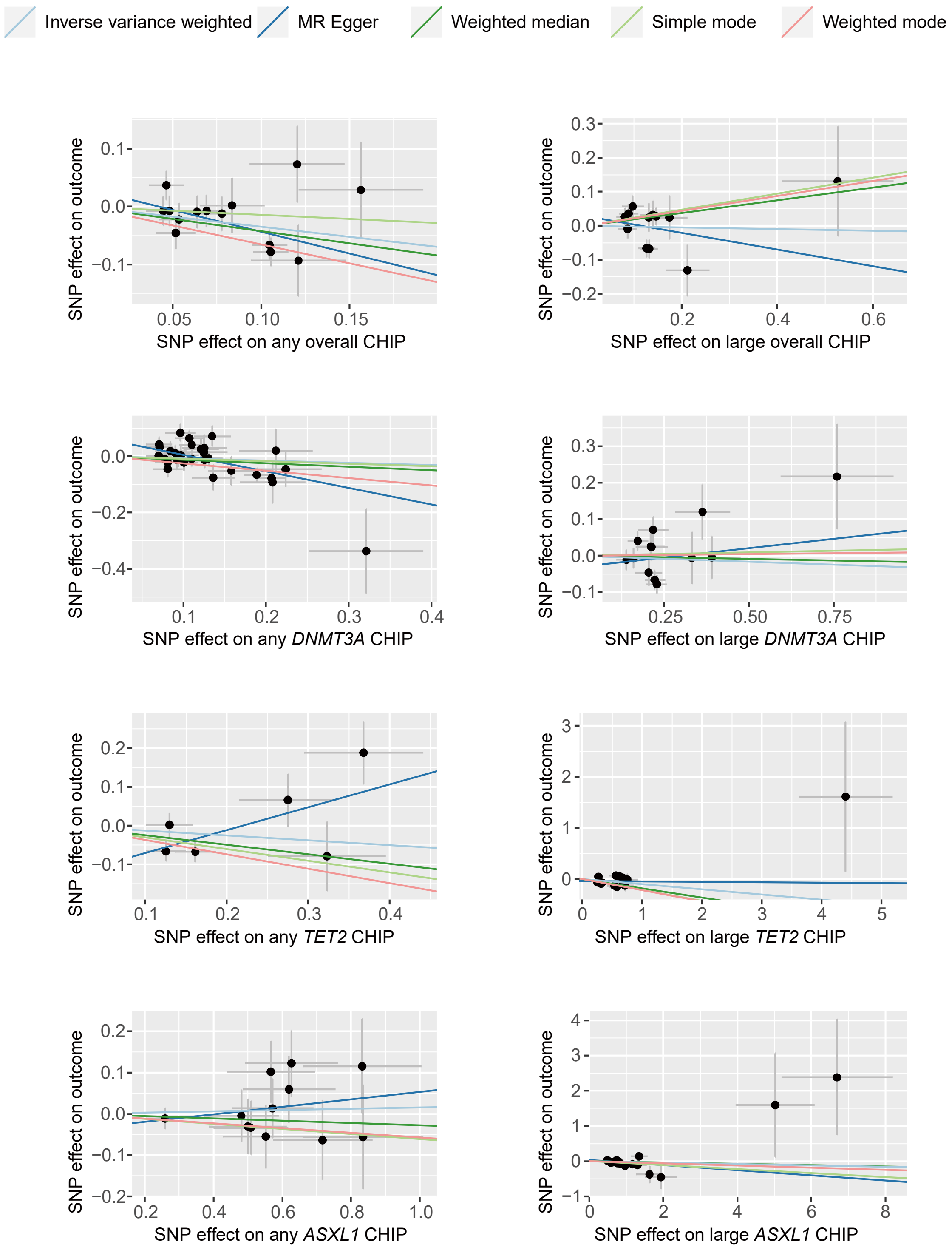

Supplementary Figure S32: Heatmap of association between CHIP and cytokines based on the model fully adjusted for covariate

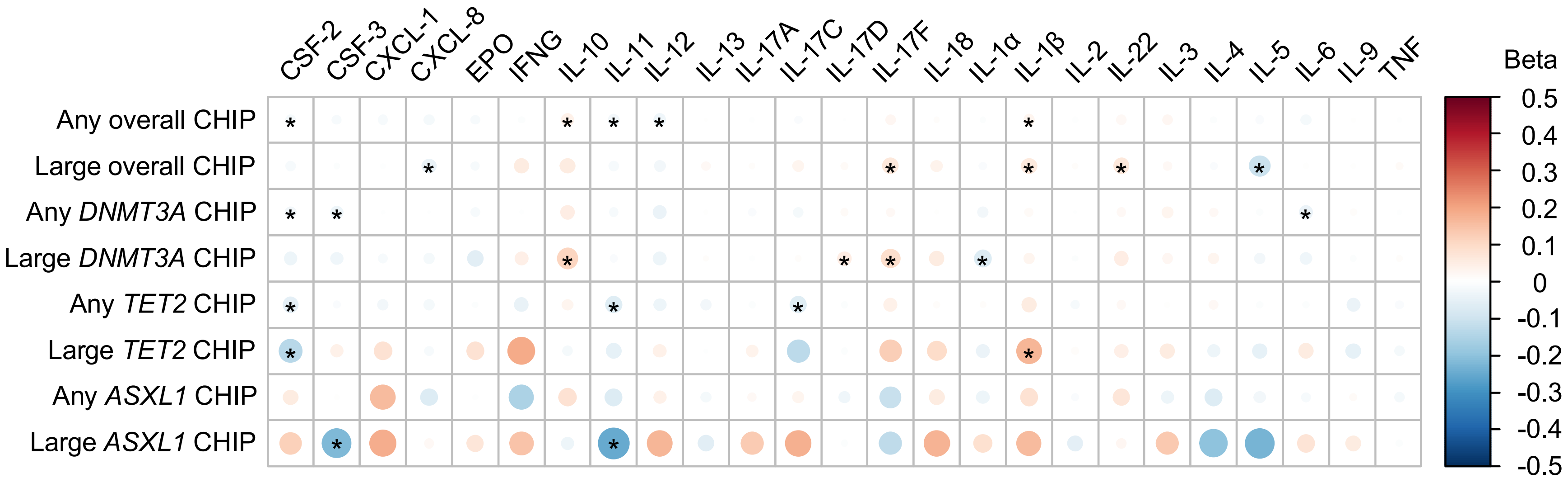

Associations of CHIP status with serum cytokine levels were detected in fully adjusted covariate models and shown in heat map. Each row presented one CHIP status, with CHIP gene and VAF threshold. Each column presented one cytokine. The size and color of each circle meant the association size and direction, respectively. The asterisk showed the nominal significant association (P < .05).

Supplementary Figure S33: Forest plot of mediation analysis for mediation effect of cytokines between large overall CHIP and AP in model 1

	Effect of CHIP on cytokines		Mediation effect of cytokines	
Cytokine	Beta (95% CI)	P value	Mediation proportion (95% CI)	P value
CSF-2	−0.03 (−0.06 − 0.00)	6.0×10−2	0.01 (−0.01 − 0.04)	3.6×10−1
CSF-3	0.01 (−0.04 − 0.06)	7.4×10−1	0.00 (−0.01 − 0.01)	8×10−1
CXCL-1	0.12 (0.03 − 0.21)	1.2×10−2	0.02 (0.00 − 0.05)	8×10−2
CXCL-8	−0.02 (−0.09 − 0.04)	4.8×10−1	0.00 (−0.01 − 0.02)	5.2×10−1
EPO	−0.02 (−0.09 − 0.06)	6.4×10−1	0.00 (−0.01 − 0.02)	6.8×10−1
IFNG	0.08 (−0.04 − 0.19)	1.9×10−1	0.00 (−0.03 − 0.01)	4.2×10−1
IL-10	0.13 (0.05 − 0.21)	8.0×10−4	0.00 (−0.02 − 0.03)	8.6×10−1
IL-11	−0.01 (−0.07 − 0.05)	7.5×10−1	0.00 (−0.01 − 0.01)	8.6×10−1
IL-12	−0.01 (−0.09 − 0.07)	7.6×10−1	0.00 (−0.03 − 0.02)	7.8×10−1
IL-13	0.03 (−0.02 − 0.08)	2.6×10−1	0.00 (−0.01 − 0.01)	9.4×10−1
IL-17A	0 (−0.04 − 0.05)	8.3×10−1	0.00 (−0.01 − 0.01)	9.4×10−1
IL-17C	0.06 (−0.02 − 0.13)	1.3×10−1	−0.01 (−0.03 − 0.00)	1.2×10−1
IL-17D	−0.01 (−0.04 − 0.03)	7.4×10−1	0.00 (−0.01 − 0.01)	7.8×10−1
IL-17F	0.09 (0.01 − 0.17)	2.1×10−2	−0.01 (−0.02 − 0.01)	2.8×10−1
IL-18	0.11 (0.07 − 0.16)	2.8×10−6	0.00 (−0.03 − 0.03)	9.2×10−1
IL-1α	0.07 (0.02 − 0.11)	5.7×10−3	0.00 (−0.01 − 0.03)	6.2×10−1
IL-1β	0.3 (0.25 − 0.36)	2.4×10−30	0.17 (0.09 − 0.24)	< 2×10-16
IL-2	0.01 (−0.02 − 0.04)	4.2×10−1	0.00 (−0.01 − 0.02)	6×10−1
IL-22	0.04 (−0.03 − 0.11)	2.6×10−1	−0.01 (−0.03 − 0.00)	2.4×10−1
IL-3	0.02 (−0.04 − 0.07)	5.4×10−1	0.00 (−0.02 − 0.01)	7.6×10−1
IL-4	0.00 (−0.09 − 0.09)	9.8×10−1	0.00 (−0.01 − 0.01)	9.8×10−1
IL-5	−0.11 (−0.24 − 0.02)	10.0×10−2	0.00 (−0.01 − 0.02)	9.8×10−1
IL-6	0.02 (−0.05 − 0.10)	5.4×10−1	0.00 (−0.03 − 0.01)	5.4×10−1
IL-9	0.00 (−0.06 − 0.05)	9.2×10−1	0.00 (−0.01 − 0.01)	8.6×10−1
TNF	0.01 (−0.02 − 0.05)	4.4×10−1	−0.01 (−0.02 − 0.01)	3.2×10−1

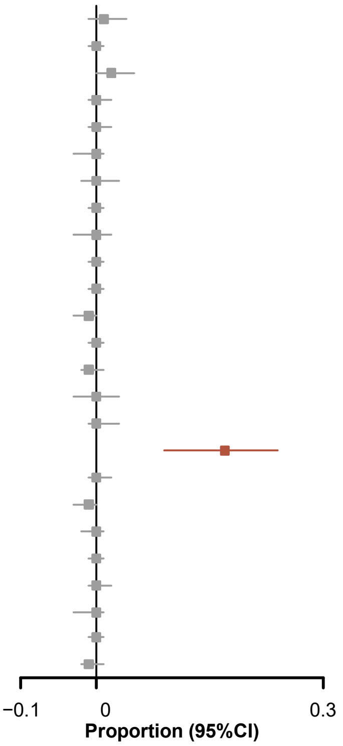

Mediation effects of cytokines in the association between large overall CHIP and AP were presented as mediation proportion, 95% CI and P value. Point estimate boxes and error bars in red indicated significant effects, while grey ones meant non-significant. Effects of large overall CHIP on each cytokine level and corresponding significances were also shown along with the forest plot.

Supplementary Figure S34: Forest plot of mediation analysis for mediation effect of cytokines between any *TET2* CHIP and AP in model 1

	Effect of CHIP on cytokines		Mediation effect of cytokines	
Cytokine	Beta (95% CI)	P value	Mediation proportion (95% CI)	P value
CSF-2	−0.06 (−0.10 − −0.01)	2.1×10−2	0.01 (−0.02 − 0.06)	4.8×10−1
CSF-3	0.03 (−0.04 − 0.10)	3.8×10−1	0.00 (−0.01 − 0.03)	5.6×10−1
CXCL-1	0.08 (−0.05 − 0.22)	2.2×10−1	0.01 (−0.01 − 0.06)	3.8×10−1
CXCL-8	0.00 (−0.09 − 0.09)	10.0×10−1	0.00 (−0.04 − 0.04)	9.0×10−1
EPO	0.00 (−0.09 − 0.09)	9.4×10−1	0.00 (−0.01 − 0.01)	9.2×10−1
IFNG	0.01 (−0.14 − 0.16)	9.2×10−1	0.00 (−0.03 − 0.02)	8.4×10−1
IL-10	0.08 (−0.03 − 0.19)	1.8×10−1	0.00 (−0.02 − 0.02)	9.6×10−1
IL-11	−0.04 (−0.12 − 0.03)	2.5×10−1	0.00 (−0.01 − 0.03)	4.0×10−1
IL-12	−0.02 (−0.13 − 0.09)	6.7×10−1	0.00 (−0.02 − 0.03)	8.4×10−1
IL-13	−0.02 (−0.09 − 0.04)	5.4×10−1	0.00 (−0.01 − 0.01)	9.4×10−1
IL-17A	−0.02 (−0.07 − 0.04)	6.2×10−1	0.00 (−0.01 − 0.02)	8.8×10−1
IL-17C	−0.02 (−0.11 − 0.07)	6.2×10−1	0.00 (−0.01 − 0.03)	7.2×10−1
IL-17D	−0.02 (−0.06 − 0.03)	5.0×10−1	0.01 (−0.03 − 0.05)	5.6×10−1
IL-17F	0.07 (−0.04 − 0.18)	2.0×10−1	0.00 (−0.01 − 0.04)	6.2×10−1
IL-18	0.10 (0.04 − 0.16)	2.1×10−3	0.02 (−0.01 − 0.06)	3.2×10−1
IL-1α	0.04 (−0.03 − 0.11)	3.0×10−1	0.00 (−0.02 − 0.01)	5.4×10−1
IL-1β	0.30 (0.23 − 0.37)	3.4×10−17	0.18 (0.10 − 0.32)	<2×10-16
IL-2	0.00 (−0.04 − 0.04)	9.6×10−1	0.00 (−0.01 − 0.02)	8.8×10−1
IL-22	0.03 (−0.05 − 0.12)	4.4×10−1	0.00 (−0.03 − 0.02)	7.2×10−1
IL-3	0.01 (−0.06 − 0.08)	7.5×10−1	0.00 (−0.02 − 0.03)	7.2×10−1
IL-4	0.03 (−0.08 − 0.14)	5.7×10−1	0.00 (−0.02 − 0.01)	9.0×10−1
IL-5	0.05 (−0.14 − 0.24)	6.0×10−1	0.00 (−0.02 − 0.02)	9.2×10−1
IL-6	0.02 (−0.09 − 0.13)	7.0×10−1	−0.01 (−0.04 − 0.02)	6.0×10−1
IL-9	−0.02 (−0.09 − 0.04)	4.7×10−1	0.00 (−0.01 − 0.01)	9.4×10−1
TNF	0.02 (−0.02 − 0.07)	3.4×10−1	0.00 (−0.04 − 0.01)	3.8×10−1

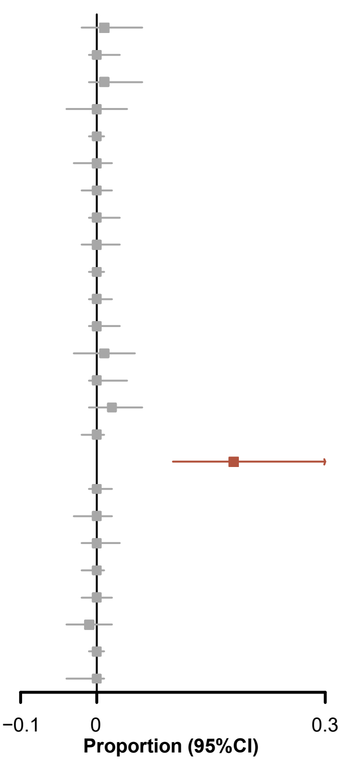

Mediation effects of cytokines in the association between any *TET2* CHIP and AP were presented as mediation proportion, 95% CI and P value. Point estimate boxes and error bars in red indicated significant effects, while grey ones meant non-significant. Effects of any *TET2* CHIP on each cytokine level and corresponding significances were also shown along with the forest plot.

Supplementary Figure S35: Forest plot of mediation analysis for mediation effect of cytokines between large *TET2* CHIP and AP in model 1

	Effect of CHIP on cytokines		Mediation effect of cytokines	
Cytokine	Beta (95% CI)	P value	Mediation proportion (95% CI)	P value
CSF-2	−0.14 (−0.23 − −0.05)	4.2×10−3	−0.03 (−0.25 − 0.09)	5.2×10−1
CSF-3	0.08 (−0.06 − 0.21)	2.9×10−1	0.02 (−0.01 − 0.09)	2.2×10−1
CXCL-1	0.25 (−0.01 − 0.50)	5.9×10−2	0.04 (0.00 − 0.11)	8.0×10−2
CXCL-8	0.02 (−0.14 − 0.19)	7.9×10−1	0.00 (−0.08 − 0.02)	6.2×10−1
EPO	0.12 (−0.06 − 0.30)	2.1×10−1	0.00 (−0.07 − 0.04)	8.6×10−1
IFNG	0.24 (−0.06 − 0.54)	1.1×10−1	0.01 (−0.02 − 0.08)	4.2×10−1
IL-10	0.08 (−0.16 − 0.32)	5.0×10−1	0.00 (−0.04 − 0.05)	9.2×10−1
IL-11	−0.06 (−0.24 − 0.12)	5.1×10−1	−0.01 (−0.05 − 0.02)	4.8×10−1
IL-12	−0.02 (−0.26 − 0.21)	8.6×10−1	0.00 (−0.04 − 0.02)	9.2×10−1
IL-13	0.03 (−0.13 − 0.18)	7.2×10−1	0.00 (−0.03 − 0.02)	8.4×10−1
IL-17A	0.04 (−0.11 − 0.18)	5.9×10−1	0.00 (−0.03 − 0.06)	9.6×10−1
IL-17C	−0.09 (−0.27 − 0.09)	3.5×10−1	0.00 (−0.03 − 0.04)	9.2×10−1
IL-17D	−0.03 (−0.11 − 0.06)	5.5×10−1	0.00 (−0.05 − 0.02)	8.2×10−1
IL-17F	0.14 (−0.11 − 0.39)	2.7×10−1	0.00 (−0.05 − 0.05)	9.6×10−1
IL-18	0.16 (0.02 − 0.31)	3.0×10−2	−0.01 (−0.05 − 0.03)	5.4×10−1
IL-1α	0.05 (−0.07 − 0.18)	4.0×10−1	0.00 (−0.05 − 0.02)	9.4×10−1
IL-1β	0.44 (0.28 − 0.60)	1.6×10−7	0.16 (0.03 − 0.33)	2.0×10−2
IL-2	0.01 (−0.09 − 0.11)	8.1×10−1	0.00 (−0.06 − 0.06)	8.6×10−1
IL-22	0.01 (−0.18 − 0.20)	9.0×10−1	0.00 (−0.15 − 0.07)	7.8×10−1
IL-3	0.04 (−0.11 − 0.19)	5.9×10−1	0.00 (−0.05 − 0.04)	7.4×10−1
IL-4	−0.17 (−0.37 − 0.04)	1.1×10−1	0.00 (−0.11 − 0.04)	9.4×10−1
IL-5	−0.07 (−0.46 − 0.31)	7.1×10−1	0.00 (−0.07 − 0.08)	9.6×10−1
IL-6	0.15 (−0.04 − 0.34)	1.2×10−1	−0.03 (−0.09 − 0.01)	1.8×10−1
IL-9	0.01 (−0.11 − 0.13)	8.6×10−1	0.00 (−0.03 − 0.03)	9.2×10−1
TNF	−0.03 (−0.12 − 0.05)	4.5×10−1	0.00 (−0.02 − 0.06)	7.6×10−1

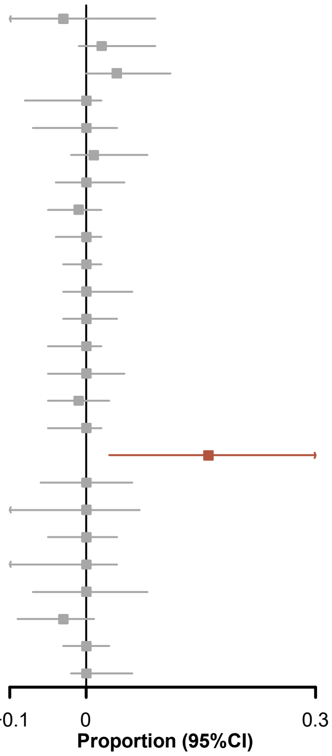

Mediation effects of cytokines in the association between large *TET2* CHIP and AP were presented as mediation proportion, 95% CI and P value. Point estimate boxes and error bars in red indicated significant effects, while grey ones meant non-significant. Effects of large *TET2* CHIP on each cytokine level and corresponding significances were also shown along with the forest plot.

Supplementary Figure S36: Forest plot of mediation analysis for mediation effect of cytokines between any overall CHIP and UAP in model 1

Cytokine	Effect of CHIP on cytokines: Beta (95% CI)	Effect of CHIP on cytokines: P value	Mediation effect of cytokines: Mediation proportion (95% CI)	Mediation effect of cytokines: P value
CSF-2	−0.02 (−0.04 − 0.00)	3.7×10−2	0.00 (0.00 − 0.02)	3.2×10−1
CSF-3	−0.01 (−0.03 − 0.02)	6.8×10−1	0.00 (0.00 − 0.00)	8.2×10−1
CXCL-1	0.12 (0.07 − 0.17)	4.0×10−6	0.00 (−0.01 − 0.02)	5.6×10−1
CXCL-8	0.02 (−0.02 − 0.06)	2.5×10−1	0.00 (−0.02 − 0.00)	2.0×10−1
EPO	−0.03 (−0.07 − 0.01)	2.0×10−1	0.00 (0.00 − 0.01)	2.6×10−1
IFNG	0.03 (−0.04 − 0.09)	4.1×10−1	0.00 (−0.01 − 0.00)	6.4×10−1
IL-10	0.07 (0.02 − 0.11)	2.3×10−3	0.00 (−0.02 − 0.01)	8.4×10−1
IL-11	−0.01 (−0.04 − 0.02)	5.2×10−1	0.00 (0.00 − 0.02)	4.0×10−1
IL-12	−0.01 (−0.05 − 0.04)	7.6×10−1	0.00 (−0.01 − 0.01)	9.2×10−1
IL-13	0.01 (−0.02 − 0.03)	5.9×10−1	0.00 (−0.01 − 0.00)	7.4×10−1
IL-17A	0.00 (−0.03 − 0.02)	7.0×10−1	0.00 (0.00 − 0.00)	8.4×10−1
IL-17C	0.01 (−0.02 − 0.05)	4.4×10−1	0.00 (−0.01 − 0.00)	4.2×10−1
IL-17D	0.01 (−0.01 − 0.02)	5.2×10−1	0.00 (−0.02 − 0.01)	5.8×10−1
IL-17F	0.04 (0.00 − 0.08)	4.4×10−2	0.00 (−0.01 − 0.01)	7.2×10−1
IL-18	0.09 (0.07 − 0.12)	5.2×10−13	−0.01 (−0.04 − 0.01)	3.4×10−1
IL-1α	0.04 (0.01 − 0.06)	1.4×10−2	0.00 (−0.01 − 0.01)	5.6×10−1
IL-1β	0.27 (0.25 − 0.30)	1.8×10−89	0.08 (0.02 − 0.16)	< 2x10-16
IL-2	0.01 (−0.01 − 0.03)	2.7×10−1	0.00 (0 − 0.01)	5.0×10−1
IL-22	0.03 (−0.01 − 0.07)	1.2×10−1	−0.01 (−0.02 − 0.00)	2.4×10−1
IL-3	0.02 (−0.01 − 0.05)	1.7×10−1	0.00 (−0.01 − 0.01)	1.0
IL-4	0.00 (−0.05 − 0.05)	10.0×10−1	0.00 (−0.01 − 0.01)	9.2×10−1
IL-5	0.01 (−0.06 − 0.09)	7.5×10−1	0.00 (−0.01 − 0.01)	6.6×10−1
IL-6	0.01 (−0.04 − 0.05)	7.8×10−1	0.00 (−0.01 − 0.01)	7.6×10−1
IL-9	0.00 (−0.03 − 0.03)	8.7×10−1	0.00 (−0.01 − 0.01)	9.2×10−1
TNF	0.02 (0.00 − 0.04)	1.3×10−1	−0.01 (−0.03 − 0.00)	1.2×10−1

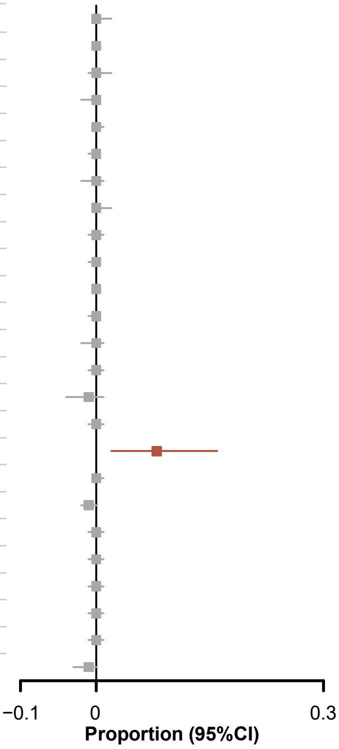

Mediation effects of cytokines in the association between any overall CHIP and UAP were presented as mediation proportion, 95% CI and P value. Point estimate boxes and error bars in red indicated significant effects, while grey ones meant non-significant. Effects of any overall CHIP on each cytokine level and corresponding significances were also shown along with the forest plot.

Supplementary Figure S37: Forest plot of mediation analysis for mediation effect of cytokines between large overall CHIP and UAP in model 1

	Effect of CHIP on cytokines		Mediation effect of cytokines	
Cytokine	Beta (95% CI)	P value	Mediation proportion (95% CI)	P value
CSF-2	−0.03 (−0.06 − 0.00)	6.0×10^{-2}	0.01 (−0.01 − 0.03)	2.4×10^{-1}
CSF-3	0.01 (−0.04 − 0.06)	7.4×10^{-1}	0.00 (−0.01 − 0.00)	9.6×10^{-1}
CXCL-1	0.12 (0.03 − 0.21)	1.2×10^{-2}	0.01 (0.00 − 0.03)	8.0×10^{-2}
CXCL-8	−0.02 (−0.09 − 0.04)	4.8×10^{-1}	0.01 (−0.01 − 0.03)	5.4×10^{-1}
EPO	−0.02 (−0.09 − 0.06)	6.4×10^{-1}	0.00 (0.00 − 0.01)	7.0×10^{-1}
IFNG	0.08 (−0.04 − 0.19)	1.9×10^{-1}	0.00 (−0.02 − 0.00)	3.6×10^{-1}
IL-10	0.13 (0.05 − 0.21)	8.0×10^{-4}	0.01 (−0.01 − 0.04)	4.0×10^{-1}
IL-11	−0.01 (−0.07 − 0.05)	7.5×10^{-1}	0.00 (−0.01 − 0.01)	9.2×10^{-1}
IL-12	−0.01 (−0.09 − 0.07)	7.6×10^{-1}	0.00 (−0.01 − 0.01)	8.4×10^{-1}
IL-13	0.03 (−0.02 − 0.08)	2.6×10^{-1}	0.00 (−0.01 − 0.01)	7.6×10^{-1}
IL-17A	0.00 (−0.04 − 0.05)	8.3×10^{-1}	0.00 (−0.01 − 0.01)	9.8×10^{-1}
IL-17C	0.06 (−0.02 − 0.13)	1.3×10^{-1}	−0.01 (−0.02 − 0.00)	2.2×10^{-1}
IL-17D	−0.01 (−0.04 − 0.03)	7.4×10^{-1}	0.00 (−0.01 − 0.01)	8.2×10^{-1}
IL-17F	0.09 (0.01 − 0.17)	2.1×10^{-2}	−0.01 (−0.03 − 0.01)	3.2×10^{-1}
IL-18	0.11 (0.07 − 0.16)	2.8×10^{-6}	0.01 (−0.02 − 0.03)	4.4×10^{-1}
IL-1α	0.07 (0.02 − 0.11)	5.7×10^{-3}	0.00 (−0.02 − 0.02)	8.6×10^{-1}
IL-1β	0.30 (0.25 − 0.36)	2.4×10^{-30}	0.14 (0.09 − 0.19)	<2×10-16
IL-2	0.01 (−0.02 − 0.04)	4.2×10^{-1}	0.00 (0.00 − 0.01)	8.2×10^{-1}
IL-22	0.04 (−0.03 − 0.11)	2.6×10^{-1}	0.00 (−0.02 − 0.00)	5.2×10^{-1}
IL-3	0.02 (−0.04 − 0.07)	5.4×10^{-1}	0.00 (−0.01 − 0.01)	8.2×10^{-1}
IL-4	0.00 (−0.09 − 0.09)	9.8×10^{-1}	0.00 (−0.01 − 0.01)	9.8×10^{-1}
IL-5	−0.11 (−0.24 − 0.02)	10.0×10^{-2}	0.00 (−0.01 − 0.01)	7.0×10^{-1}
IL-6	0.02 (−0.05 − 0.10)	5.4×10^{-1}	0.00 (−0.01 − 0.00)	6.0×10^{-1}
IL-9	0.00 (−0.06 − 0.05)	9.2×10^{-1}	0.00 (−0.01 − 0.01)	9.4×10^{-1}
TNF	0.01 (−0.02 − 0.05)	4.4×10^{-1}	−0.01 (−0.02 − 0.01)	4.2×10^{-1}

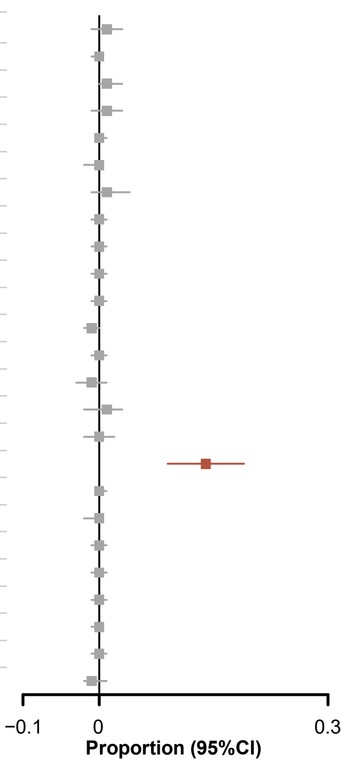

Mediation effects of cytokines in the association between large overall CHIP and UAP were presented as mediation proportion, 95% CI and P value. Point estimate boxes and error bars in red indicated significant effects, while grey ones meant non-significant. Effects of large overall CHIP on each cytokine level and corresponding significances were also shown along with the forest plot.

Supplementary Figure S38: Forest plot of mediation analysis for mediation effect of cytokines between any *DNMT3A* CHIP and UAP in model 1

	Effect of CHIP on cytokines		Mediation effect of cytokines	
Cytokine	Beta (95% CI)	P value	Mediation proportion (95% CI)	P value
CSF-2	−0.03 (−0.05 − 0.00)	9.4×10−2	0.00 (−0.19 − 0.27)	9.2×10−1
CSF-3	−0.04 (−0.08 − 0.01)	1.1×10−1	0.00 (−0.44 − 0.20)	8.2×10−1
CXCL-1	0.06 (−0.02 − 0.14)	1.1×10−1	0.01 (−0.42 − 0.53)	8.4×10−1
CXCL-8	0.03 (−0.03 − 0.08)	3.7×10−1	0.00 (−0.34 − 0.31)	9.4×10−1
EPO	−0.03 (−0.09 − 0.04)	4.1×10−1	−0.01 (−0.64 − 0.59)	7.4×10−1
IFNG	0.03 (−0.06 − 0.13)	5.0×10−1	0.00 (−0.08 − 0.32)	9.2×10−1
IL-10	0.06 (−0.01 − 0.12)	9.5×10−2	0.01 (−0.33 − 0.24)	8.0×10−1
IL-11	−0.02 (−0.08 − 0.03)	3.8×10−1	0.00 (−0.15 − 0.18)	9.2×10−1
IL-12	−0.03 (−0.10 − 0.03)	3.3×10−1	−0.01 (−1.54 − 0.66)	6.8×10−1
IL-13	−0.01 (−0.05 − 0.03)	7.9×10−1	0.00 (−0.16 − 0.17)	9.4×10−1
IL-17A	−0.03 (−0.06 − 0.01)	1.8×10−1	0.00 (−0.07 − 0.27)	6.2×10−1
IL-17C	−0.03 (−0.09 − 0.03)	3.0×10−1	0.00 (−0.22 − 0.14)	9.0×10−1
IL-17D	0.01 (−0.02 − 0.04)	6.4×10−1	0.01 (−0.39 − 0.43)	7.0×10−1
IL-17F	0.04 (−0.02 − 0.10)	2.3×10−1	0.00 (−0.10 − 0.53)	9.2×10−1
IL-18	0.04 (0.01 − 0.08)	2.7×10−2	0.03 (−0.89 − 0.59)	5.8×10−1
IL-1α	0.00 (−0.04 − 0.05)	8.7×10−1	0.00 (−0.13 − 0.20)	8.8×10−1
IL-1β	0.10 (0.06 − 0.15)	4.9×10−6	0.00 (−0.50 − 3.19)	9.8×10−1
IL-2	0.00 (−0.03 − 0.03)	8.9×10−1	0.00 (−0.10 − 0.17)	9.8×10−1
IL-22	−0.01 (−0.07 − 0.05)	8.4×10−1	0.00 (−0.13 − 0.28)	9.6×10−1
IL-3	0.06 (0.01 − 0.11)	3.1×10−2	0.00 (−0.20 − 0.23)	9.4×10−1
IL-4	0.03 (−0.05 − 0.10)	5.0×10−1	0.00 (−0.11 − 0.14)	9.4×10−1
IL-5	−0.01 (−0.13 − 0.11)	8.3×10−1	0.00 (−0.13 − 0.10)	9.4×10−1
IL-6	−0.05 (−0.11 − 0.02)	1.7×10−1	−0.02 (−4.68 − 0.92)	7.6×10−1
IL-9	0.01 (−0.03 − 0.06)	5.9×10−1	0.00 (−0.13 − 0.13)	6.6×10−1
TNF	−0.01 (−0.04 − 0.02)	5.1×10−1	−0.01 (−0.61 − 0.30)	8.2×10−1

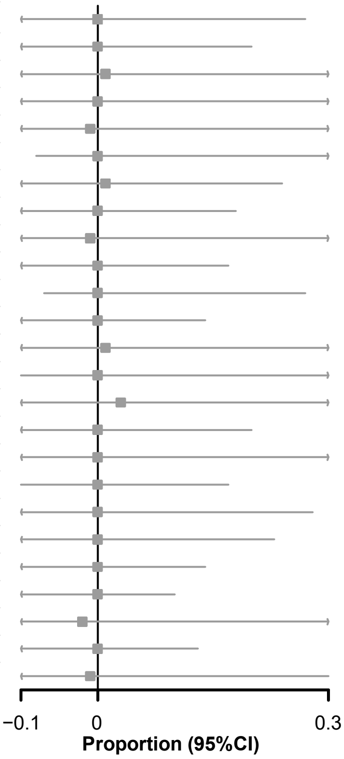

Mediation effects of cytokines in the association between any *DNMT3A* CHIP and UAP were presented as mediation proportion, 95% CI and P value. Point estimate boxes and error bars in red indicated significant effects, while grey ones meant non-significant. Effects of any *DNMT3A* CHIP on each cytokine level and corresponding significances were also shown along with the forest plot.

Supplementary Figure S39: Forest plot of mediation analysis for mediation effect of cytokines between any *TET2* CHIP and UAP in model 1

	Effect of CHIP on cytokines		Mediation effect of cytokines	
Cytokine	Beta (95% CI)	P value	Mediation proportion (95% CI)	P value
CSF-2	−0.06 (−0.10 − −0.01)	2.1×10−2	0.01 (−0.04 − 0.07)	6.0×10−1
CSF-3	0.03 (−0.04 − 0.10)	3.8×10−1	0.00 (−0.01 − 0.03)	6.4×10−1
CXCL-1	0.08 (−0.05 − 0.22)	2.2×10−1	0.02 (−0.02 − 0.04)	3.0×10−1
CXCL-8	0.00 (−0.09 − 0.09)	1.00	0.00 (−0.03 − 0.03)	8.8×10−1
EPO	0.00 (−0.09 − 0.09)	9.4×10−1	0.00 (−0.01 − 0.02)	8.8×10−1
IFNG	0.01 (−0.14 − 0.16)	9.2×10−1	0.00 (−0.01 − 0.01)	8.4×10−1
IL-10	0.08 (−0.03 − 0.19)	1.8×10−1	0.00 (−0.02 − 0.02)	9.8×10−1
IL-11	−0.04 (−0.12 − 0.03)	2.5×10−1	0.00 (−0.01 − 0.03)	5.0×10−1
IL-12	−0.02 (−0.13 − 0.09)	6.7×10−1	0.00 (−0.01 − 0.01)	9.8×10−1
IL-13	−0.02 (−0.09 − 0.04)	5.4×10−1	0.00 (−0.02 − 0.01)	6.4×10−1
IL-17A	−0.02 (−0.07 − 0.04)	6.2×10−1	0.00 (−0.01 − 0.02)	8.6×10−1
IL-17C	−0.02 (−0.11 − 0.07)	6.2×10−1	0.00 (−0.01 − 0.03)	5.2×10−1
IL-17D	−0.02 (−0.06 − 0.03)	5.0×10−1	0.00 (−0.01 − 0.04)	5.2×10−1
IL-17F	0.07 (−0.04 − 0.18)	2.0×10−1	0.00 (−0.01 − 0.02)	8.8×10−1
IL-18	0.10 (0.04 − 0.16)	2.1×10−3	0.00 (−0.02 − 0.04)	7.4×10−1
IL-1α	0.04 (−0.03 − 0.11)	3.0×10−1	0.00 (−0.01 − 0.02)	9.4×10−1
IL-1β	0.30 (0.23 − 0.37)	3.4×10−17	0.15 (0.07 − 0.22)	<2×10-16
IL-2	0.00 (−0.04 − 0.04)	9.6×10−1	0.00 (−0.01 − 0.01)	9.6×10−1
IL-22	0.03 (−0.05 − 0.12)	4.4×10−1	−0.01 (−0.03 − 0.02)	5.6×10−1
IL-3	0.01 (−0.06 − 0.08)	7.5×10−1	0.00 (−0.02 − 0.02)	8.8×10−1
IL-4	0.03 (−0.08 − 0.14)	5.7×10−1	0.00 (−0.02 − 0.01)	9.0×10−1
IL-5	0.05 (−0.14 − 0.24)	6.0×10−1	0.00 (−0.02 − 0.01)	8.0×10−1
IL-6	0.02 (−0.09 − 0.13)	7.0×10−1	0.00 (−0.01 − 0.01)	9.2×10−1
IL-9	−0.02 (−0.09 − 0.04)	4.7×10−1	0.00 (−0.01 − 0.01)	9.0×10−1
TNF	0.02 (−0.02 − 0.07)	3.4×10−1	−0.01 (−0.04 − 0.01)	5.6×10−1

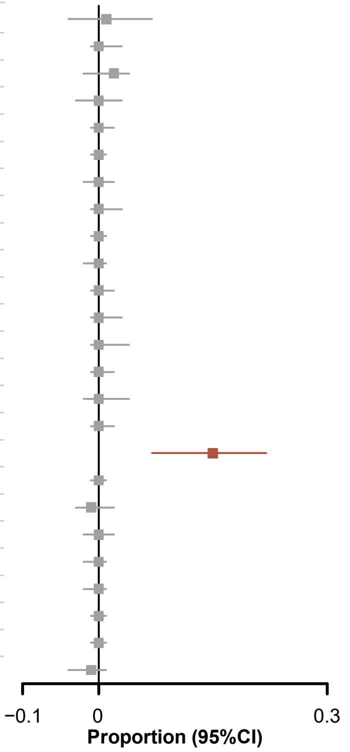

Mediation effects of cytokines in the association between any *TET2* CHIP and UAP were presented as mediation proportion, 95% CI and P value. Point estimate boxes and error bars in red indicated significant effects, while grey ones meant non-significant. Effects of any *TET2* CHIP on each cytokine level and corresponding significances were also shown along with the forest plot.

Supplementary Figure S40: Forest plot of mediation analysis for mediation effect of cytokines between large *TET2* CHIP and UAP in model 2

Cytokine	Effect of CHIP on cytokines: Beta (95% CI)	Effect of CHIP on cytokines: P value	Mediation effect of cytokines: Mediation proportion (95% CI)	Mediation effect of cytokines: P value
CSF-2	−0.21 (−0.31 − −0.11)	3.1×10−5	−0.01 (−0.19 − 0.22)	9.2×10−1
CSF-3	0.02 (−0.11 − 0.16)	7.3×10−1	0.01 (−0.05 − 0.09)	5.4×10−1
CXCL-1	0.41 (0.15 − 0.67)	2.0×10−3	0.06 (−0.02 − 0.21)	1.2×10−1
CXCL-8	0.03 (−0.14 − 0.19)	7.6×10−1	0.00 (−0.04 − 0.02)	7.2×10−1
EPO	0.03 (−0.16 − 0.21)	7.7×10−1	0.00 (−0.03 − 0.03)	9.4×10−1
IFNG	0.09 (−0.20 − 0.38)	5.5×10−1	0.01 (−0.02 − 0.09)	5.8×10−1
IL-10	0.07 (−0.14 − 0.28)	5.0×10−1	0.00 (−0.04 − 0.06)	7.8×10−1
IL-11	−0.09 (−0.27 − 0.09)	3.2×10−1	−0.01 (−0.07 − 0.02)	4.0×10−1
IL-13	0.06 (−0.10 − 0.21)	4.9×10−1	0.00 (−0.06 − 0.03)	7.0×10−1
IL-17A	0.04 (−0.09 − 0.16)	5.6×10−1	0.00 (−0.02 − 0.08)	6.8×10−1
IL-17C	−0.13 (−0.32 − 0.06)	2.0×10−1	0.00 (−0.05 − 0.04)	7.4×10−1
IL-17D	−0.01 (−0.09 − 0.07)	8.0×10−1	0.00 (−0.05 − 0.03)	9.8×10−1
IL-17F	0.17 (−0.07 − 0.41)	1.7×10−1	0.02 (−0.03 − 0.12)	4.0×10−1
IL-18	0.12 (−0.02 − 0.26)	1.0×10−1	−0.01 (−0.10 − 0.05)	7.0×10−1
IL-1α	0.01 (−0.13 − 0.14)	9.2×10−1	0.00 (−0.03 − 0.04)	9.4×10−1
IL-1β	0.39 (0.23 − 0.56)	6.3×10−6	0.16 (0.01 − 0.40)	2.0×10−2
IL-2	0.05 (−0.05 − 0.15)	3.3×10−1	0.00 (−0.05 − 0.05)	9.8×10−1
IL-22	0.09 (−0.11 − 0.28)	3.7×10−1	0.00 (−0.10 − 0.03)	8.0×10−1
IL-3	0.01 (−0.14 − 0.15)	9.3×10−1	0.00 (−0.07 − 0.03)	9.4×10−1
IL-4	−0.09 (−0.32 − 0.14)	4.4×10−1	0.00 (−0.07 − 0.03)	8.0×10−1
IL-5	−0.29 (−0.70 − 0.12)	1.7×10−1	0.03 (−0.02 − 0.12)	3.0×10−1
IL-6	0.00 (−0.15 − 0.16)	9.6×10−1	0.00 (−0.06 − 0.06)	8.8×10−1
IL-7	0.35 (0.18 − 0.52)	7.9×10−5	0.04 (−0.06 − 0.21)	3.6×10−1
IL-9	0.00 (−0.12 − 0.13)	9.4×10−1	0.00 (−0.11 − 0.08)	8.8×10−1
TNF	−0.07 (−0.15 − 0.02)	1.2×10−1	0.00 (−0.07 − 0.07)	8.0×10−1

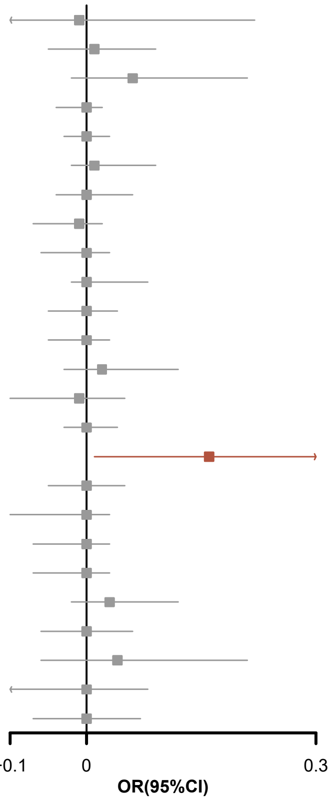

Mediation effects of cytokines in the association between large *TET2* CHIP and UAP were presented as mediation proportion, 95% CI and P value. Point estimate boxes and error bars in red indicated significant effects, while grey ones meant non-significant. Effects of any *TET2* CHIP on each cytokine level and corresponding significances were also shown along with the forest plot.

Supplementary Figure S41: Forest plot of mediation analysis for mediation effect of cytokines between large *TET2* CHIP and UAP in model 3

Cytokine	Effect of CHIP on cytokines: Beta (95% CI)	Effect of CHIP on cytokines: P value	Mediation effect of cytokines: Mediation proportion (95% CI)	Mediation effect of cytokines: P value
CSF-2	−0.22 (−0.31 − −0.12)	1.8×10^{-5}	0.02 (−0.20 − 0.30)	7.6×10^{-1}
CSF-3	0.03 (−0.11 − 0.17)	6.7×10^{-1}	0.00 (−0.05 − 0.11)	7.8×10^{-1}
CXCL-1	0.40 (0.14 − 0.66)	2.6×10^{-3}	0.07 (−0.01 − 0.19)	1.8×10^{-1}
CXCL-8	0.03 (−0.14 − 0.20)	7.2×10^{-1}	0.00 (−0.03 − 0.03)	1.0
EPO	0.03 (−0.16 − 0.21)	7.7×10^{-1}	0.00 (−0.03 − 0.04)	9.4×10^{-1}
IFNG	0.08 (−0.21 − 0.37)	5.8×10^{-1}	0.01 (−0.04 − 0.06)	5.4×10^{-1}
IL-10	0.09 (−0.10 − 0.29)	3.5×10^{-1}	0.01 (−0.06 − 0.09)	5.2×10^{-1}
IL-11	−0.08 (−0.26 − 0.10)	3.7×10^{-1}	−0.02 (−0.18 − 0.02)	4.2×10^{-1}
IL-13	0.08 (−0.08 − 0.23)	3.3×10^{-1}	0.00 (−0.06 − 0.04)	9.4×10^{-1}
IL-17A	0.02 (−0.10 − 0.15)	7.0×10^{-1}	0.00 (−0.05 − 0.05)	9.4×10^{-1}
IL-17C	−0.12 (−0.32 − 0.07)	2.1×10^{-1}	0.00 (−0.05 − 0.05)	8.6×10^{-1}
IL-17D	−0.01 (−0.09 − 0.07)	8.2×10^{-1}	0.00 (−0.03 − 0.03)	9.4×10^{-1}
IL-17F	0.17 (−0.07 − 0.41)	1.6×10^{-1}	0.02 (−0.04 − 0.12)	6.0×10^{-1}
IL-18	0.12 (−0.03 − 0.26)	1.1×10^{-1}	−0.01 (−0.14 − 0.08)	6.4×10^{-1}
IL-1α	0.00 (−0.13 − 0.14)	9.5×10^{-1}	0.00 (−0.05 − 0.04)	9.6×10^{-1}
IL-1β	0.39 (0.23 − 0.56)	6.3×10^{-6}	0.16 (0.00 − 0.43)	4.0×10^{-2}
IL-2	0.06 (−0.04 − 0.16)	2.7×10^{-1}	0.00 (−0.09 − 0.03)	9.4×10^{-1}
IL-22	0.10 (−0.09 − 0.30)	3.0×10^{-1}	0.00 (−0.06 − 0.05)	8.2×10^{-1}
IL-3	0.00 (−0.15 − 0.14)	9.6×10^{-1}	0.00 (−0.04 − 0.04)	8.6×10^{-1}
IL-4	−0.10 (−0.33 − 0.12)	3.7×10^{-1}	0.00 (−0.06 − 0.05)	8.8×10^{-1}
IL-5	−0.19 (−0.57 − 0.19)	3.2×10^{-1}	0.00 (−0.04 − 0.07)	7.8×10^{-1}
IL-6	0.00 (−0.15 − 0.16)	9.6×10^{-1}	0.00 (−0.07 − 0.05)	8.0×10^{-1}
IL-7	0.35 (0.17 − 0.52)	1.0×10^{-4}	0.04 (−0.11 − 0.19)	5.0×10^{-1}
IL-9	0.00 (−0.12 − 0.12)	9.9×10^{-1}	−0.01 (−0.18 − 0.07)	7.6×10^{-1}
TNF	−0.08 (−0.16 − 0.01)	8.8×10^{-2}	0.01 (−0.07 − 0.10)	6.2×10^{-1}

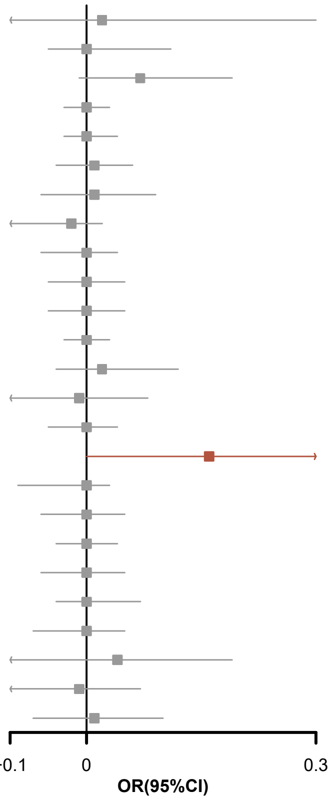

Mediation effects of cytokines in the association between large *TET2* CHIP and UAP were presented as mediation proportion, 95% CI and P value. Point estimate boxes and error bars in red indicated significant effects, while grey ones meant non-significant. Effects of any *TET2* CHIP on each cytokine level and corresponding significances were also shown along with the forest plot.

Supplementary Figure S42: Forest plot of mediation analysis for mediation effect of cytokines between large *TET2* CHIP and UAP in model 4

Cytokine	Effect of CHIP on cytokines: Beta (95% CI)	Effect of CHIP on cytokines: P value	Mediation effect of cytokines: Mediation proportion (95% CI)	Mediation effect of cytokines: P value
CSF-2	−0.22 (−0.31 − −0.12)	1.8×10−5	0.05 (−0.19 − 0.27)	6.6×10−1
CSF-3	0.03 (−0.11 − 0.17)	6.7×10−1	0.00 (−0.05 − 0.10)	8.4×10−1
CXCL-1	0.40 (0.14 − 0.66)	2.6×10−3	0.06 (−0.06 − 0.18)	2×10−1
CXCL-8	0.03 (−0.14 − 0.20)	7.2×10−1	0.00 (−0.03 − 0.03)	9.8×10−1
EPO	0.03 (−0.16 − 0.21)	7.7×10−1	0.00 (−0.06 − 0.02)	7.6×10−1
IFNG	0.08 (−0.21 − 0.37)	5.8×10−1	0.01 (−0.04 − 0.08)	6.4×10−1
IL-10	0.09 (−0.10 − 0.29)	3.5×10−1	0.02 (−0.02 − 0.08)	3.8×10−1
IL-11	−0.08 (−0.26 − 0.10)	3.7×10−1	−0.01 (−0.13 − 0.03)	4.8×10−1
IL-13	0.08 (−0.08 − 0.23)	3.3×10−1	0.00 (−0.07 − 0.06)	8.4×10−1
IL-17A	0.02 (−0.10 − 0.15)	7.0×10−1	0.00 (−0.04 − 0.03)	9.8×10−1
IL-17C	−0.12 (−0.32 − 0.07)	2.1×10−1	0.00 (−0.09 − 0.06)	7.6×10−1
IL-17D	−0.01 (−0.09 − 0.07)	8.2×10−1	0.00 (−0.05 − 0.03)	9.6×10−1
IL-17F	0.17 (−0.07 − 0.41)	1.6×10−1	0.01 (−0.02 − 0.10)	5.6×10−1
IL-18	0.12 (−0.03 − 0.26)	1.1×10−1	−0.02 (−0.09 − 0.07)	6.2×10−1
IL-1α	0.00 (−0.13 − 0.14)	9.5×10−1	0.00 (−0.05 − 0.04)	9.8×10−1
IL-1β	0.38 (0.21 − 0.54)	1.7×10−5	0.15 (0.02 − 0.49)	2×10−2
IL-2	0.06 (−0.04 − 0.16)	2.7×10−1	0.00 (−0.06 − 0.04)	9.6×10−1
IL-22	0.1 (−0.09 − 0.30)	3.0×10−1	0.00 (−0.13 − 0.06)	7.8×10−1
IL-3	0.00 (−0.15 − 0.14)	9.6×10−1	0.00 (−0.04 − 0.04)	8.8×10−1
IL-4	−0.10 (−0.33 − 0.12)	3.7×10−1	0.00 (−0.08 − 0.03)	8.6×10−1
IL-5	−0.19 (−0.57 − 0.19)	3.2×10−1	0.00 (−0.03 − 0.07)	8.2×10−1
IL-6	0.00 (−0.15 − 0.16)	9.6×10−1	0.00 (−0.06 − 0.04)	9.8×10−1
IL-7	0.35 (0.17 − 0.52)	1.0×10−4	0.05 (−0.08 − 0.16)	5×10−1
IL-9	0.00 (−0.12 − 0.12)	9.9×10−1	0.00 (−0.13 − 0.07)	9.8×10−1
TNF	−0.08 (−0.16 − 0.01)	8.8×10−2	0.01 (−0.05 − 0.09)	6.4×10−1

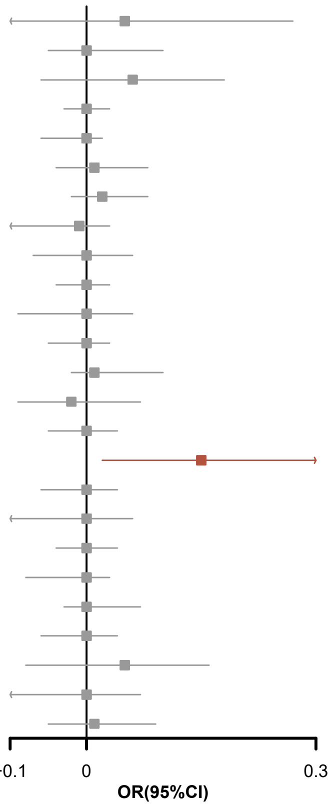

Mediation effects of cytokines in the association between large *TET2* CHIP and UAP were presented as mediation proportion, 95% CI and P value. Point estimate boxes and error bars in red indicated significant effects, while grey ones meant non-significant. Effects of any *TET2* CHIP on each cytokine level and corresponding significances were also shown along with the forest plot.

Supplementary Figure S43: Forest plot of mediation analysis for mediation effect of cytokines between large *TET2* CHIP and UAP in model 5

Cytokine	Effect of CHIP on cytokines: Beta (95% CI)	Effect of CHIP on cytokines: P value	Mediation effect of cytokines: Mediation proportion (95% CI)	Mediation effect of cytokines: P value
CSF-2	−0.22 (−0.31 − −0.12)	1.8×10^{-5}	0.03 (−0.22 − 0.26)	6.6×10^{-1}
CSF-3	0.03 (−0.11 − 0.17)	6.7×10^{-1}	0.00 (−0.05 − 0.08)	6.6×10^{-1}
CXCL-1	0.40 (0.14 − 0.66)	2.6×10^{-3}	0.05 (−0.03 − 0.27)	2.4×10^{-1}
CXCL-8	0.03 (−0.14 − 0.20)	7.2×10^{-1}	0.00 (−0.06 − 0.03)	9.0×10^{-1}
EPO	0.03 (−0.16 − 0.21)	7.7×10^{-1}	0.00 (−0.05 − 0.03)	9.6×10^{-1}
IFNG	0.08 (−0.21 − 0.37)	5.8×10^{-1}	0.01 (−0.04 − 0.10)	5.8×10^{-1}
IL-10	0.09 (−0.10 − 0.29)	3.5×10^{-1}	0.01 (−0.05 − 0.10)	6.4×10^{-1}
IL-11	−0.08 (−0.26 − 0.10)	3.7×10^{-1}	−0.01 (−0.24 − 0.03)	4.8×10^{-1}
IL-13	0.08 (−0.08 − 0.23)	3.3×10^{-1}	0.00 (−0.05 − 0.05)	9.8×10^{-1}
IL-17A	0.02 (−0.10 − 0.15)	7.0×10^{-1}	0.00 (−0.03 − 0.06)	8.8×10^{-1}
IL-17C	−0.12 (−0.32 − 0.07)	2.1×10^{-1}	0.00 (−0.07 − 0.04)	7.8×10^{-1}
IL-17D	−0.01 (−0.09 − 0.07)	8.2×10^{-1}	0.00 (−0.04 − 0.03)	9.4×10^{-1}
IL-17F	0.17 (−0.07 − 0.41)	1.6×10^{-1}	0.02 (−0.07 − 0.09)	4.4×10^{-1}
IL-18	0.12 (−0.03 − 0.26)	1.1×10^{-1}	−0.01 (−0.07 − 0.05)	7.6×10^{-1}
IL-1α	0.00 (−0.13 − 0.14)	9.5×10^{-1}	0.00 (−0.06 − 0.03)	9.4×10^{-1}
IL-1β	0.39 (0.22 − 0.56)	9.6×10^{-6}	0.15 (0.01 − 0.53)	4.0×10^{-2}
IL-2	0.06 (−0.04 − 0.16)	2.7×10^{-1}	0.00 (−0.08 − 0.05)	8.8×10^{-1}
IL-22	0.10 (−0.09 − 0.3)	3.0×10^{-1}	0.00 (−0.09 − 0.03)	8.2×10^{-1}
IL-3	0.00 (−0.15 − 0.14)	9.6×10^{-1}	0.00 (−0.05 − 0.05)	9.2×10^{-1}
IL-4	−0.10 (−0.33 − 0.12)	3.7×10^{-1}	0.00 (−0.08 − 0.06)	8.8×10^{-1}
IL-5	−0.19 (−0.57 − 0.19)	3.2×10^{-1}	0.00 (−0.04 − 0.06)	7.8×10^{-1}
IL-6	0.00 (−0.15 − 0.16)	9.6×10^{-1}	0.00 (−0.06 − 0.04)	9.0×10^{-1}
IL-7	0.35 (0.17 − 0.52)	1.0×10^{-4}	0.04 (−0.11 − 0.21)	4.8×10^{-1}
IL-9	0.00 (−0.12 − 0.12)	9.9×10^{-1}	0.00 (−0.14 − 0.09)	8.8×10^{-1}
TNF	−0.08 (−0.16 − 0.01)	8.8×10^{-2}	0.01 (−0.06 − 0.11)	6.0×10^{-1}

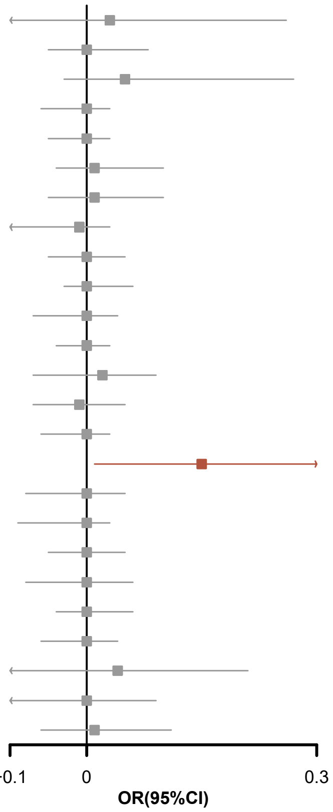

Mediation effects of cytokines in the association between large *TET2* CHIP and UAP were presented as mediation proportion, 95% CI and P value. Point estimate boxes and error bars in red indicated significant effects, while grey ones meant non-significant. Effects of any *TET2* CHIP on each cytokine level and corresponding significances were also shown along with the forest plot.

Supplementary Figure S44: Forest plot of mediation analysis for mediation effect of cytokines between large *TET2* CHIP and UAP in model 6

	Effect of CHIP on cytokines		Mediation effect of cytokines	
Cytokine	Beta (95% CI)	P value	Mediation proportion (95% CI)	P value
CSF-2	−0.21 (−0.31 − −0.11)	2.8×10−5	0.04 (−0.84 − 0.66)	8.2×10−1
CSF-3	0.01 (−0.12 − 0.15)	8.4×10−1	0.00 (−0.06 − 0.13)	7.6×10−1
CXCL-1	0.42 (0.16 − 0.68)	1.8×10−3	0.08 (−0.13 − 0.25)	4.2×10−1
CXCL-8	−0.01 (−0.21 − 0.19)	9.3×10−1	0.00 (−0.05 − 0.03)	8.8×10−1
EPO	0.05 (−0.14 − 0.24)	6.0×10−1	0.00 (−0.04 − 0.09)	8.6×10−1
IFNG	0.02 (−0.26 − 0.30)	8.8×10−1	0.00 (−0.05 − 0.11)	9.6×10−1
IL-10	0.08 (−0.12 − 0.28)	4.3×10−1	0.02 (−0.04 − 0.19)	5.6×10−1
IL-11	−0.09 (−0.27 − 0.09)	3.5×10−1	−0.02 (−0.23 − 0.07)	4.8×10−1
IL-13	0.08 (−0.08 − 0.23)	3.2×10−1	0.00 (−0.12 − 0.08)	9.2×10−1
IL-17A	0.02 (−0.10 − 0.15)	7.0×10−1	0.00 (−0.06 − 0.09)	8.8×10−1
IL-17C	−0.13 (−0.32 − 0.06)	1.9×10−1	−0.01 (−0.19 − 0.09)	6.8×10−1
IL-17D	0.00 (−0.08 − 0.08)	9.0×10−1	0.00 (−0.15 − 0.06)	9.8×10−1
IL-17F	0.19 (−0.05 − 0.43)	1.2×10−1	0.04 (−0.03 − 0.21)	3.6×10−1
IL-18	0.13 (−0.02 − 0.27)	8.4×10−2	−0.04 (−0.19 − 0.03)	3.8×10−1
IL-1α	−0.02 (−0.15 − 0.12)	7.8×10−1	0.00 (−0.10 − 0.08)	8.0×10−1
IL-1β	0.41 (0.25 − 0.57)	1.5×10−6	0.12 (0.03 − 0.30)	2.0×10−2
IL-2	0.06 (−0.04 − 0.16)	2.5×10−1	0.00 (−0.10 − 0.07)	8.6×10−1
IL-22	0.12 (−0.07 − 0.31)	2.0×10−1	0.00 (−0.21 − 0.21)	9.2×10−1
IL-3	0.01 (−0.13 − 0.16)	8.7×10−1	0.00 (−0.10 − 0.22)	9.4×10−1
IL-4	−0.12 (−0.35 − 0.10)	2.9×10−1	−0.01 (−0.15 − 0.05)	6.0×10−1
IL-5	−0.16 (−0.52 − 0.2)	3.9×10−1	0.00 (−0.08 − 0.11)	8.4×10−1
IL-6	0.02 (−0.14 − 0.17)	8.3×10−1	0.00 (−0.13 − 0.16)	8.4×10−1
IL-7	0.36 (0.19 − 0.54)	6.3×10−5	−0.01 (−0.37 − 0.37)	8.8×10−1
IL-9	−0.01 (−0.13 − 0.12)	9.3×10−1	0.00 (−0.36 − 0.18)	9.2×10−1
TNF	−0.06 (−0.15 − 0.02)	1.5×10−1	0.01 (−0.10 − 0.15)	6.6×10−1

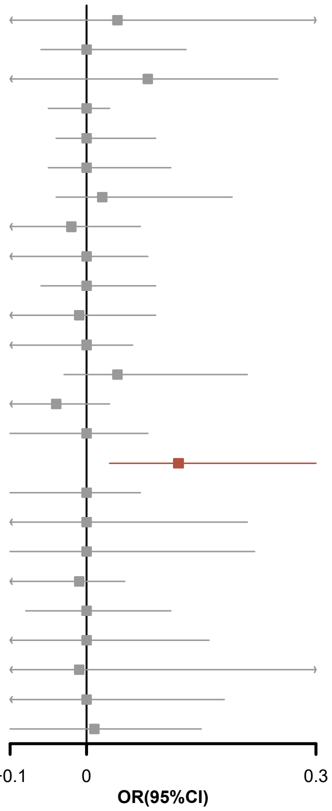

Mediation effects of cytokines in the association between large *TET2* CHIP and UAP were presented as mediation proportion, 95% CI and P value. Point estimate boxes and error bars in red indicated significant effects, while grey ones meant non-significant. Effects of any *TET2* CHIP on each cytokine level and corresponding significances were also shown along with the forest plot.

Supplementary Figure S45: Forest plot of mediation analysis for mediation effect of cytokines between large *TET2* CHIP and UAP in model 7

Cytokine	Effect of CHIP on cytokines: Beta (95% CI)	Effect of CHIP on cytokines: P value	Mediation effect of cytokines: Mediation proportion (95% CI)	Mediation effect of cytokines: P value
CSF-2	−0.21 (−0.31 − −0.12)	2.8×10−5	0.08 (−0.98 − 0.68)	6.4×10−1
CSF-3	0.03 (−0.11 − 0.16)	7.1×10−1	0.00 (−0.13 − 0.08)	8.8×10−1
CXCL-1	0.4 (0.13 − 0.66)	3.5×10−3	0.06 (−0.76 − 0.75)	5.6×10−1
CXCL-8	−0.03 (−0.23 − 0.17)	7.5×10−1	0.00 (−0.16 − 0.12)	9.8×10−1
EPO	0.04 (−0.15 − 0.22)	6.9×10−1	0.00 (−0.26 − 0.17)	9.8×10−1
IFNG	0.09 (−0.19 − 0.37)	5.3×10−1	0.01 (−0.08 − 0.28)	6.8×10−1
IL-10	0.09 (−0.11 − 0.29)	3.9×10−1	0.01 (−0.16 − 0.77)	8.0×10−1
IL-11	−0.11 (−0.29 − 0.07)	2.3×10−1	0.00 (−0.26 − 0.48)	7.2×10−1
IL-13	0.07 (−0.08 − 0.23)	3.5×10−1	−0.01 (−0.28 − 0.08)	8.4×10−1
IL-17A	0.01 (−0.11 − 0.14)	8.2×10−1	0.00 (−0.09 − 0.15)	7.8×10−1
IL-17C	−0.12 (−0.31 − 0.08)	2.3×10−1	0.00 (−0.21 − 0.17)	8.2×10−1
IL-17D	−0.01 (−0.09 − 0.07)	8.8×10−1	0.00 (−0.13 − 1.15)	9.8×10−1
IL-17F	0.16 (−0.07 − 0.40)	1.8×10−1	0.00 (−0.19 − 0.37)	8.4×10−1
IL-18	0.11 (−0.04 − 0.25)	1.4×10−1	−0.03 (−0.41 − 0.43)	3.6×10−1
IL-1α	−0.02 (−0.17 − 0.12)	7.3×10−1	0.00 (−0.21 − 0.09)	8.0×10−1
IL-1β	0.41 (0.25 − 0.57)	1.5×10−6	0.12 (0.03 − 0.25)	2.0×10−2
IL-2	0.04 (−0.06 − 0.14)	4.6×10−1	−0.01 (−0.32 − 0.08)	6.4×10−1
IL-22	0.12 (−0.07 − 0.31)	2.2×10−1	0.02 (−0.47 − 0.40)	6.8×10−1
IL-3	0.02 (−0.13 − 0.17)	8.1×10−1	0.00 (−0.33 − 0.17)	9.0×10−1
IL-4	−0.15 (−0.38 − 0.07)	1.9×10−1	0.00 (−0.17 − 0.12)	9.4×10−1
IL-5	−0.16 (−0.52 − 0.20)	4.0×10−1	0.00 (−0.12 − 0.20)	9.0×10−1
IL-6	0.02 (−0.14 − 0.17)	8.4×10−1	0.00 (−0.16 − 0.15)	8.8×10−1
IL-7	0.37 (0.19 − 0.54)	7.8×10−5	0.09 (−0.19 − 1.57)	4×10−1
IL-9	−0.01 (−0.13 − 0.12)	9.1×10−1	0.00 (−0.68 − 0.32)	9.2×10−1
TNF	−0.06 (−0.15 − 0.03)	1.7×10−1	0.00 (−0.27 − 0.13)	1.0

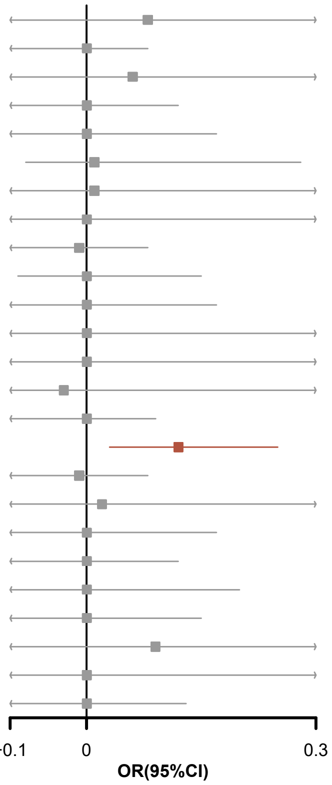

Mediation effects of cytokines in the association between large *TET2* CHIP and UAP were presented as mediation proportion, 95% CI and P value. Point estimate boxes and error bars in red indicated significant effects, while grey ones meant non-significant. Effects of any *TET2* CHIP on each cytokine level and corresponding significances were also shown along with the forest plot.

Supplementary Figure S46: Forest plot of mediation analysis for mediation effect of candidate metabolites

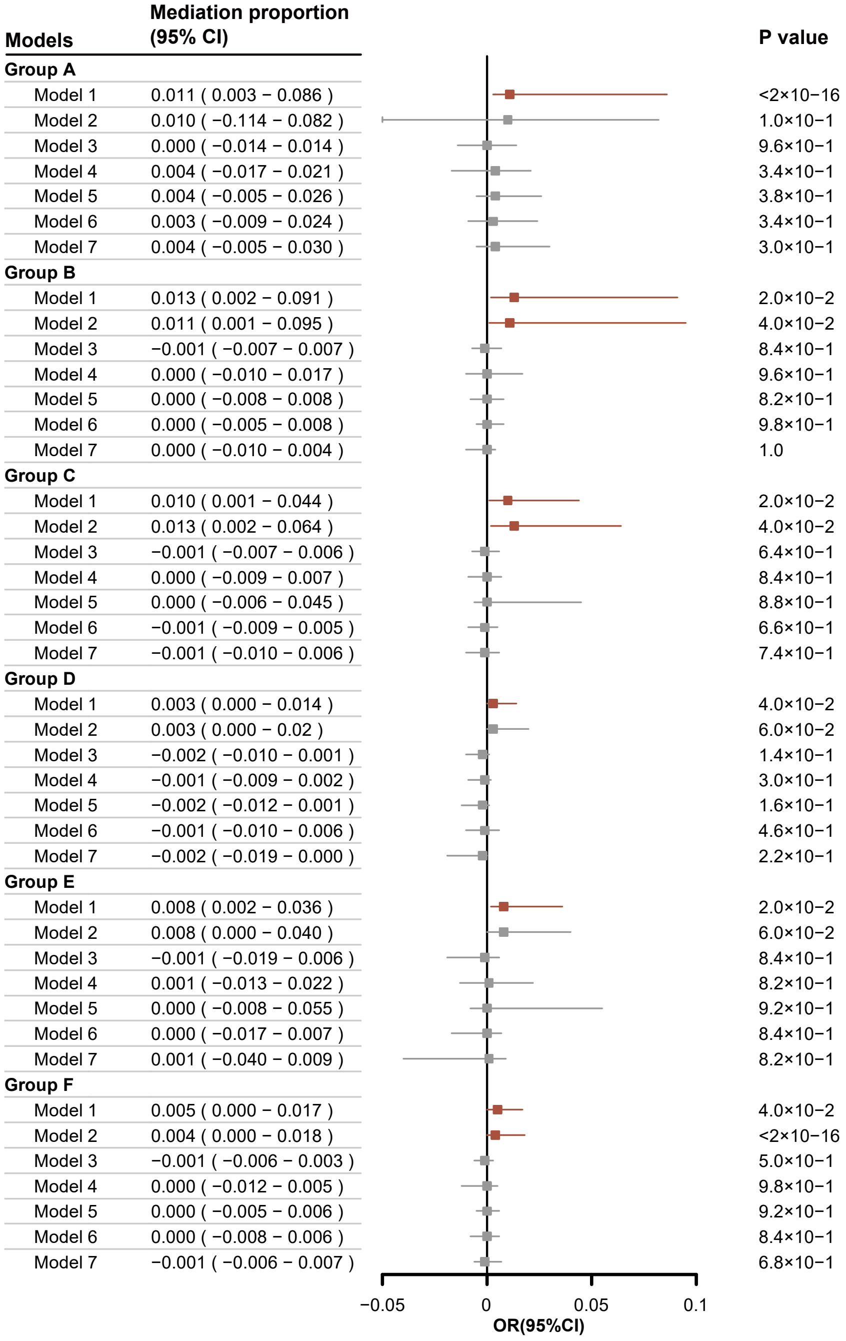

Group ID	Exposure	Mediator	Outcome
Group A	Large TET2 CHIP	Albumin	Unstable angina pectoris
Group B	Large overall CHIP	Cholesterol to Total Lipids in Very Large VLDL percentage	Unstable angina pectoris
Group C	Large overall CHIP	Cholesteryl Esters to Total Lipids in Very Large VLDL percentage	Unstable angina pectoris
Group D	Large TET2 CHIP	Phenylalanine	Unstable angina pectoris
Group E	Large overall CHIP	Tyrosine	Angina pectoris
Group F	Large TET2 CHIP	Tyrosine	Angina pectoris

Supplementary Tables

Supplementary Table S1: Phenotyping identification in UKB cohort

Phenotype	Source	Code
AP	ICD-9	413
	ICD-10	I20
UAP	ICD-10	I20.0
SAP	ICD-10	I20.8
Congenital heart disease	ICD-9*	746, 747
	ICD-10*	Q20, Q21, Q22, Q23
Atherosclerotic cardiovascular diseases	ICD-9*	410, 4109, 411, 4119, 412, 4129, 4140, 4148, 4149, 434, 4340, 4341, 4349, 436, 4400, 4402, 4438, 4439
	ICD-10*	I21, I22, I23, I24, I25, I63, I64, I70, I73.8, I73.9
Coronary artery diseases	ICD-9*	410, 4109, 411, 4119, 412, 4129, 4140, 4148, 4149
	ICD-10*	I21, I22, I23, I24, I25
Myocardial infarction	ICD-9*	410
	ICD-10*	I21
Haematological malignancies	ICD-9*	200, 201, 202, 204, 205, 208, 238
	ICD-10*	C81, C82, C83, C84, C85, C86, C88, C90, C91, C92, C93, C94, C95, C96, D45, D46, D473, D474

Abbreviations: AP, angina pectoris; UAP, unstable angina pectoris; SAP, stable angina pectoris.
* For congenital heart disease, haematological malignancies, atherosclerotic cardiovascular diseases and coronary artery diseases, ICD9 and ICD10 codes were adopted. In definitions of other diseases, main ICD9 and main ICD10 codes were used for higher specificity.

Supplementary Table S2: Olink0 proteins used in association and mediation analysis

Coding*	Abbreviation	Full name
698	CSF-2	Granulocyt×10-macrophage colony-stimulating factor
701	CSF-3	Granulocyte colony-stimulating factor
739	CXCL-1	Growth-regulated alpha protein
750	CXCL-8	Interleukin-8
952	EPO	Erythropoietin
1329	IFNG	Interferon gamma
1360	IL-10	Interleukin-10
1363	IL-11	Interleukin-11
1364	IL-12	Interleukin-12
1368	IL-13	Interleukin-13
1374	IL-17A	Interleukin-17A
1375	IL-17C	Interleukin-17C
1376	IL-17D	Interleukin-17D
1377	IL-17F	Interleukin-17F
1380	IL-18	Interleukin-18
1385	IL--1α	Interleukin-1 alpha
1386	IL--1β	Interleukin-1 beta
1393	IL-2	Interleukin-2
1398	IL-22	Interleukin-22
1405	IL-3	Interleukin-3
1414	IL-4	Interleukin-4
1416	IL-5	Interleukin-5
1418	IL-6	Interleukin-6
1423	IL-9	Interleukin-9
2712	TNF	Tumor necrosis factor

* The coding relates the integer Protein ID presented in the OLINK dataset to the UniProt meaning.

Supplementary Table S3: Baseline characteristics of the *DNMT3A* CHIP cohorts in association analysis of covariate models

	CHIP, No.(%)				
**Characteristics**	**No CHIP (n=419801)**	**Any CHIP (n=12596)**	**P value**	**Large CHIP (n=3279)**	**P value**
Age, mean (SD), y	56.3 (8.10)	59.5 (7.16)	<0.001	60.3 (6.88)	<0.001
Sex			<0.001		<0.001
Female	228475 (54.4)	7441 (59.1)		1935 (59.0)	
Male	191326 (45.6)	5155 (40.9)		1344 (41.0)	
Ancestry			0.640		0.036
White British	353145 (84.1)	10616 (84.3)		2803 (85.5)	
Other	66656 (15.9)	1980 (15.7)		476 (14.5)	
BMI, mean (SD)	27.4 (4.77)	27.3 (4.63)	0.023	27.2 (4.47)	0.004
Alcohol intake frequency			<0.001		0.009
Never	32903 (7.84)	1016 (8.07)		257 (7.84)	
Special occasions only	47869 (11.4)	1475 (11.7)		382 (11.6)	
One to three times a month	46951 (11.2)	1367 (10.9)		353 (10.8)	
Once or twice a week	108822 (25.9)	3119 (24.8)		820 (25.0)	
Three or four times a week	97848 (23.3)	2817 (22.4)		716 (21.8)	
Daily or almost daily	85408 (20.3)	2802 (22.2)		751 (22.9)	

	CHIP, No.(%)				
**Characteristics**	**No CHIP (n=419801)**	**Any CHIP (n=12596)**	**P value**	**Large CHIP (n=3279)**	**P value**
Ever smoked			<0.001		<0.001
Ever	250541 (59.7)	7943 (63.1)		2101 (64.1)	
Never	169260 (40.3)	4653 (36.9)		1178 (35.9)	
Townsend deprivation index, mean (SD)	-1.33 (3.07)	-1.41 (3.03)	0.002	-1.47 (3.04)	0.007
Diabetes	2974 (0.71)	88 (0.70)	0.940	26 (0.79)	0.638
Atherosclerotic heart disease	21590 (5.14)	720 (5.72)	0.004	188 (5.73)	0.138
Atrial fibrillation and flutter	13896 (3.31)	466 (3.70)	0.017	126 (3.84)	0.099
Heart failure	4480 (1.07)	171 (1.36)	0.002	53 (1.62)	0.003
Chronic renal failure	1314 (0.31)	41 (0.33)	0.868	11 (0.34)	0.942
Colchicine use			1.000		0.215
Yes	203 (0.05)	6 (0.05)		3 (0.09)	
No	419598 (99.95)	12590 (99.95)		3276 (99.91)	

Baseline characteristics were compared between participants of non-CHIP and different CHIP statuses using t-test. Chi-squared or exact Fisher test was used for categorical variables. Abbreviations: SD, standard deviation; BMI, body mass index.

Supplementary Table S4: Baseline characteristics of the *TET2* CHIP cohorts in association analysis of covariate models

	CHIP, No.(%)				
**Characteristics**	**No CHIP (n=419801)**	**Any CHIP (n=5150)**	**P value**	**Large CHIP (n=1091)**	**P value**
Age, mean (SD), y	56.3 (8.10)	59.0 (7.63)	<0.001	61.0 (6.73)	<0.001
Sex			<0.001		0.007
Female	228475 (54.4)	2664 (51.7)		549 (50.3)	
Male	191326 (45.6)	2486 (48.3)		542 (49.7)	
Ancestry			0.302		0.470
White British	353145 (84.1)	4360 (84.7)		927 (85.0)	
Other	66656 (15.9)	790 (15.3)		164 (15.0)	
BMI, mean (SD)	27.4 (4.77)	27.6 (4.76)	0.004	27.8 (4.62)	0.008
Alcohol intake frequency			<0.001		<0.001
Never	32903 (7.84)	451 (8.76)		87 (7.97)	
Special occasions only	47869 (11.4)	539 (10.5)		118 (10.8)	
One to three times a month	46951 (11.2)	506 (9.83)		92 (8.43)	
Once or twice a week	108822 (25.9)	1323 (25.7)		261 (23.9)	
Three or four times a week	97848 (23.3)	1180 (22.9)		256 (23.5)	
Daily or almost daily	85408 (20.3)	1151 (22.3)		277 (25.4)	

	CHIP, No.(%)				
**Characteristics**	**No CHIP (n=419801)**	**Any CHIP (n=5150)**	**P value**	**Large CHIP (n=1091)**	**P value**
Ever smoked			0.776		0.029
Ever	250541 (59.7)	3063 (59.5)		687 (63.0)	
Never	169260 (40.3)	2087 (40.5)		404 (37.0)	
Townsend deprivation index, mean (SD)	-1.33 (3.07)	-1.38 (3.07)	0.274	-1.42 (2.99)	0.297
Diabetes	2974 (0.71)	45 (0.87)	0.187	9 (0.82)	0.781
Atherosclerotic heart disease	21590 (5.14)	302 (5.86)	0.022	85 (7.79)	<0.001
Atrial fibrillation and flutter	13896 (3.31)	199 (3.86)	0.030	51 (4.67)	0.015
Heart failure	4480 (1.07)	73 (1.42)	0.018	18 (1.65)	0.085
Chronic renal failure	1314 (0.31)	20 (0.39)	0.404	5 (0.46)	0.403
Colchicine use			0.324		0.411
Yes	203 (0.05)	4 (0.08)		1 (0.09)	
No	419598 (99.95)	5146 (99.92)		1090 (99.91)	

Baseline characteristics were compared between participants of non-CHIP and different CHIP statuses using t-test. Chi-squared or exact Fisher test was used for categorical variables. Abbreviations: SD, standard deviation; BMI, body mass index.

Supplementary Table S5: Baseline characteristics of the *ASXL1* CHIP cohorts in association analysis of covariate models

	CHIP, No.(%)				
**Characteristics**	**No CHIP (n=419801)**	**Any CHIP (n=1089)**	**P value**	**Large CHIP (n=339)**	**P value**
Age, mean (SD), y	56.3 (8.10)	60.3 (6.96)	<0.001	62.3 (5.56)	<0.001
Sex			<0.001		<0.001
Female	228475 (54.4)	436 (40.0)		120 (35.4)	
Male	191326 (45.6)	653 (60.0)		219 (64.6)	
Ancestry			0.029		0.347
White British	353145 (84.1)	943 (86.6)		292 (86.1)	
Other	66656 (15.9)	146 (13.4)		47 (13.9)	
BMI, mean (SD)	27.4 (4.77)	27.8 (4.51)	0.004	27.7 (4.29)	0.148
Alcohol intake frequency			0.067		0.177
Never	32903 (7.84)	85 (7.81)		27 (7.96)	
Special occasions only	47869 (11.4)	117 (10.7)		38 (11.2)	
One to three times a month	46951 (11.2)	100 (9.18)		29 (8.55)	
Once or twice a week	108822 (25.9)	285 (26.2)		87 (25.7)	
Three or four times a week	97848 (23.3)	245 (22.5)		71 (20.9)	
Daily or almost daily	85408 (20.3)	257 (23.6)		87 (25.7)	

	CHIP, No.(%)				
**Characteristics**	**No CHIP (n=419801)**	**Any CHIP (n=1089)**	**P value**	**Large CHIP (n=339)**	**P value**
Ever smoked			<0.001		<0.001
Ever	250541 (59.7)	742 (68.1)		248 (73.2)	
Never	169260 (40.3)	347 (31.9)		91 (26.8)	
Townsend deprivation index, mean (SD)	-1.33 (3.07)	-1.32 (3.22)	0.942	-1.30 (3.22)	0.882
Diabetes	2974 (0.71)	11 (1.01)	0.315	6 (1.77)	0.035
Atherosclerotic heart disease	21590 (5.14)	92 (8.45)	<0.001	32 (9.44)	0.001
Atrial fibrillation and flutter	13896 (3.31)	51 (4.68)	0.015	22 (6.49)	0.002
Heart failure	4480 (1.07)	20 (1.84)	0.020	13 (3.83)	<0.001
Chronic renal failure	1314 (0.31)	7 (0.64)	0.091	2 (0.59)	0.287
Colchicine use			0.099		0.152
Yes	203 (0.05)	2 (0.18)		1 (0.29)	
No	419598 (99.95)	1087 (99.82)		338 (99.71)	

Baseline characteristics were compared between participants of non-CHIP and different CHIP statuses using t-test. Chi-squared or exact Fisher test was used for categorical variables. Abbreviations: SD, standard deviation; BMI, body mass index.

Supplementary Table S6: Association between CHIP and mortality of AP based on the model partially adjusted for covariates

CHIP gene	VAF threshold	Outcome	P value	OR	se
overall	0.02	AP	7.62E-01	8.03E-01	7.26E-01
DNMT3A	0.02	AP	8.24E-01	7.98E-01	1.01E+00
ASXL1	0.1	AP	9.84E-01	2.42E-05	5.30E+02
DNMT3A	0.1	AP	9.79E-01	4.38E-06	4.59E+02
ASXL1	0.02	AP	9.83E-01	8.24E-06	5.56E+02
TET2	0.02	AP	9.79E-01	3.74E-06	4.82E+02
TET2	0.1	AP	9.80E-01	9.82E-06	4.69E+02
overall	0.1	AP	9.96E-01	1.01E+00	1.01E+00

Abbreviations: VAF, variant allele fraction.

Supplementary Table S7: Baseline characteristics of the overall CHIP cohorts in association analysis of PSM models

	CHIP, No.(%)			CHIP, No.(%)		
**Characteristics**	**No CHIP (n=32484)**	**Any CHIP (n=32484)**	**P value**	**No CHIP (n=9899)**	**Large CHIP (n=9899)**	**P value**
Age, mean (SD), y	58.4 (7.72)	58.4 (7.72)	1.000	58.8 (7.73)	58.8 (7.73)	1.000
Sex			1.000			1.000
Female	18133 (55.8)	18133 (55.8)		5499 (55.6)	5499 (55.6)	
Male	14351 (44.2)	14351 (44.2)		4400 (44.4)	4400 (44.4)	
Ancestry			1.000			1.000
White British	27175 (83.7)	27175 (83.7)		8116 (82.0)	8116 (82.0)	
Other	5309 (16.3)	5309 (16.3)		1783 (18.0)	1783 (18.0)	
BMI, mean (SD)	27.4 (4.77)	27.4 (4.73)	0.770	27.5 (4.67)	27.4 (4.69)	0.731
Alcohol intake frequency			0.817			0.732
Never	2667 (8.21)	2661 (8.19)		829 (8.37)	843 (8.52)	
Special occasions only	3834 (11.8)	3839 (11.8)		1215 (12.3)	1194 (12.1)	
One to three times a month	3595 (11.1)	3527 (10.9)		1091 (11.0)	1068 (10.8)	
Once or twice a week	8222 (25.3)	8144 (25.1)		2472 (25.0)	2478 (25.0)	
Three or four times a week	7343 (22.6)	7363 (22.7)		2238 (22.6)	2182 (22.0)	
Daily or almost daily	6823 (21.0)	6950 (21.4)		2054 (20.7)	2134 (21.6)	

	CHIP, No.(%)			CHIP, No.(%)		
**Characteristics**	**No CHIP (n=32484)**	**Any CHIP (n=32484)**	**P value**	**No CHIP (n=9899)**	**Large CHIP (n=9899)**	**P value**
Ever smoked			0.148			0.175
Ever	19585 (60.3)	19766 (60.8)		6021 (60.8)	6115 (61.8)	
Never	12899 (39.7)	12718 (39.2)		3878 (39.2)	3784 (38.2)	
Townsend deprivation index, mean (SD)	-1.37 (3.05)	-1.36 (3.08)	0.631	-1.33 (3.06)	-1.31 (3.11)	0.627
Diabetes	244 (0.75)	248 (0.76)	0.892	87 (0.88)	78 (0.79)	0.532
Atherosclerotic heart disease	1860 (5.73)	1860 (5.73)	1.000	584 (5.90)	584 (5.90)	1.000
Atrial fibrillation and flutter	1252 (3.85)	1157 (3.56)	0.051	358 (3.62)	364 (3.68)	0.850
Heart failure	394 (1.21)	418 (1.29)	0.417	149 (1.51)	147 (1.48)	0.953
Chronic renal failure	113 (0.35)	103 (0.32)	0.540	36 (0.36)	35 (0.35)	1.000
Colchicine use			0.222			0.803
Yes	26 (0.08)	17 (0.05)		9 (0.09)	7 (0.07)	
No	14351 (99.92)	14351 (99.95)		4400 (99.91)	4400 (99.93)	

Baseline characteristics were compared between participants of non-CHIP and different CHIP statuses using t-test. Chi-squared or exact Fisher test was used for categorical variables. Abbreviations: SD, standard deviation; BMI, body mass index.

Supplementary Table S8: Baseline characteristics of the *DNMT3A* CHIP cohorts in association analysis of PSM models

	CHIP, No.(%)			CHIP, No.(%)		
**Characteristics**	**No CHIP (n=12596)**	**Any CHIP (n=12596)**	**P value**	**No CHIP (n=3279)**	**Large CHIP (n=3279)**	**P value**
Age, mean (SD), y	59.5 (7.16)	59.5 (7.16)	0.997	60.3 (6.88)	60.3 (6.88)	0.997
Sex			1.000			1.000
Female	7441 (59.1)	7441 (59.1)		1935 (59.0)	1935 (59.0)	
Male	5155 (40.9)	5155 (40.9)		1344 (41.0)	1344 (41.0)	
Ancestry			0.959			1.000
White British	10620 (84.3)	10616 (84.3)		2804 (85.5)	2803 (85.5)	
Other	1976 (15.7)	1980 (15.7)		475 (14.5)	476 (14.5)	
BMI, mean (SD)	27.3 (4.64)	27.3 (4.63)	0.833	27.3 (4.68)	27.2 (4.47)	0.241
Alcohol intake frequency			1.000			1.000
Never	1018 (8.08)	1016 (8.07)		258 (7.87)	257 (7.84)	
Special occasions only	1474 (11.7)	1475 (11.7)		382 (11.6)	382 (11.6)	
One to three times a month	1366 (10.8)	1367 (10.9)		353 (10.8)	353 (10.8)	
Once or twice a week	3120 (24.8)	3119 (24.8)		820 (25.0)	820 (25.0)	
Three or four times a week	2815 (22.3)	2817 (22.4)		715 (21.8)	716 (21.8)	
Daily or almost daily	2803 (22.3)	2802 (22.2)		751 (22.9)	751 (22.9)	

	CHIP, No.(%)			CHIP, No.(%)		
**Characteristics**	**No CHIP (n=12596)**	**Any CHIP (n=12596)**	**P value**	**No CHIP (n=3279)**	**Large CHIP (n=3279)**	**P value**
Ever smoked			1.000			1.000
Ever	7944 (63.1)	7943 (63.1)		2101 (64.1)	2101 (64.1)	
Never	4652 (36.9)	4653 (36.9)		1178 (35.9)	1178 (35.9)	
Townsend deprivation index, mean (SD)	-1.43 (3.00)	-1.41 (3.03)	0.626	-1.48 (3.01)	-1.47 (3.04)	0.879
Diabetes	84 (0.67)	88 (0.70)	0.818	25 (0.76)	26 (0.79)	1.000
Atherosclerotic heart disease	719 (5.71)	720 (5.72)	1.000	186 (5.67)	188 (5.73)	0.958
Atrial fibrillation and flutter	450 (3.57)	466 (3.70)	0.614	134 (4.09)	126 (3.84)	0.658
Heart failure	159 (1.26)	171 (1.36)	0.542	40 (1.22)	53 (1.62)	0.210
Chronic renal failure	44 (0.35)	41 (0.33)	0.828	12 (0.37)	11 (0.34)	1.000
Colchicine use			1.000			1.000
Yes	6 (0.05)	6 (0.05)		2 (0.06)	3 (0.09)	
No	12590 (99.95)	12590 (99.95)		3277 (99.94)	3276 (99.91)	

Baseline characteristics were compared between participants of non-CHIP and different CHIP statuses using t-test. Chi-squared or exact Fisher test was used for categorical variables. Abbreviations: SD, standard deviation; BMI, body mass index.

Supplementary Table S9: Baseline characteristics of the *TET2* CHIP cohorts in association analysis of PSM models

	CHIP, No.(%)			CHIP, No.(%)		
**Characteristics**	**No CHIP (n=5150)**	**Any CHIP (n=5150)**	**P value**	**No CHIP (n=1091)**	**Large CHIP (n=1091)**	**P value**
Age, mean (SD), y	59.0 (7.63)	59.0 (7.63)	1.000	61.0 (6.73)	61.0 (6.73)	1.000
Sex			1.000			1.000
Female	2664 (51.7)	2664 (51.7)		549 (50.3)	549 (50.3)	
Male	2486 (48.3)	2486 (48.3)		542 (49.7)	542 (49.7)	
Ancestry			1.000			1.000
White British	4360 (84.7)	4360 (84.7)		927 (85.0)	927 (85.0)	
Other	790 (15.3)	790 (15.3)		164 (15.0)	164 (15.0)	
BMI, mean (SD)	27.6 (4.71)	27.6 (4.76)	0.718	27.7 (4.83)	27.8 (4.62)	0.578
Alcohol intake frequency			0.483			0.545
Never	429 (8.33)	451 (8.76)		96 (8.80)	87 (7.97)	
Special occasions only	599 (11.6)	539 (10.5)		105 (9.62)	118 (10.8)	
One to three times a month	502 (9.75)	506 (9.83)		115 (10.5)	92 (8.43)	
Once or twice a week	1290 (25.0)	1323 (25.7)		258 (23.6)	261 (23.9)	
Three or four times a week	1199 (23.3)	1180 (22.9)		250 (22.9)	256 (23.5)	
Daily or almost daily	1131 (22.0)	1151 (22.3)		267 (24.5)	277 (25.4)	

	CHIP, No.(%)			CHIP, No.(%)		
**Characteristics**	**No CHIP (n=5150)**	**Any CHIP (n=5150)**	**P value**	**No CHIP (n=1091)**	**Large CHIP (n=1091)**	**P value**
Ever smoked			0.703			0.533
Ever	3083 (59.9)	3063 (59.5)		702 (64.3)	687 (63.0)	
Never	2067 (40.1)	2087 (40.5)		389 (35.7)	404 (37.0)	
Townsend deprivation index, mean (SD)	-1.38 (3.05)	-1.38 (3.07)	0.932	-1.41 (3.01)	-1.42 (2.99)	0.904
Diabetes	37 (0.72)	45 (0.87)	0.438	6 (0.55)	9 (0.82)	0.604
Atherosclerotic heart disease	302 (5.86)	302 (5.86)	1.000	85 (7.79)	85 (7.79)	1.000
Atrial fibrillation and flutter	202 (3.92)	199 (3.86)	0.919	53 (4.86)	51 (4.67)	0.920
Heart failure	72 (1.40)	73 (1.42)	1.000	22 (2.02)	18 (1.65)	0.632
Chronic renal failure	10 (0.19)	20 (0.39)	0.100	7 (0.64)	5 (0.46)	0.772
Colchicine use			0.546			1.000
Yes	7 (0.14)	4 (0.08)		1 (0.09)	1 (0.07)	
No	5143 (99.86)	5146 (99.92)		1090 (99.91)	1090 (99.91)	

Baseline characteristics were compared between participants of non-CHIP and different CHIP statuses using t-test. Chi-squared or exact Fisher test was used for categorical variables. Abbreviations: SD, standard deviation; BMI, body mass index.

Supplementary Table S10: Baseline characteristics of the *ASXL1* CHIP cohorts in association analysis of PSM models

	CHIP, No.(%)			CHIP, No.(%)		
**Characteristics**	**No CHIP (n=1089)**	**No CHIP (n=1089)**	**P value**	**No CHIP (n=339)**	**Large CHIP (n=339)**	**P value**
Age, mean (SD), y	60.3 (6.96)	60.3 (6.96)	1.000	62.3 (5.56)	62.3 (5.56)	1.000
Sex			1.000			1.000
Female	436 (40.0)	436 (40.0)		120 (35.4)	120 (35.4)	
Male	653 (60.0)	653 (60.0)		219 (64.6)	219 (64.6)	
Ancestry			1.000			1.000
White British	943 (86.6)	943 (86.6)		292 (86.1)	292 (86.1)	
Other	146 (13.4)	146 (13.4)		47 (13.9)	47 (13.9)	
BMI, mean (SD)	27.7 (4.59)	27.8 (4.51)	0.600	27.8 (4.80)	27.7 (4.29)	0.766
Alcohol intake frequency			1.000			1.000
Never	85 (7.81)	85 (7.81)		27 (7.96)	27 (7.96)	
Special occasions only	117 (10.7)	117 (10.7)		38 (11.2)	38 (11.2)	
One to three times a month	100 (9.18)	100 (9.18)		29 (8.55)	29 (8.55)	
Once or twice a week	285 (26.2)	285 (26.2)		87 (25.7)	87 (25.7)	
Three or four times a week	245 (22.5)	245 (22.5)		71 (20.9)	71 (20.9)	
Daily or almost daily	257 (23.6)	257 (23.6)		87 (25.7)	87 (25.7)	

	CHIP, No.(%)			CHIP, No.(%)		
**Characteristics**	**No CHIP (n=1089)**	**No CHIP (n=1089)**	**P value**	**No CHIP (n=339)**	**Large CHIP (n=339)**	**P value**
Ever smoked			1.000			1.000
Ever	742 (68.1)	742 (68.1)		248 (73.2)	248 (73.2)	
Never	347 (31.9)	347 (31.9)		91 (26.8)	91 (26.8)	
Townsend deprivation index, mean (SD)	-1.36 (3.03)	-1.32 (3.22)	0.766	-1.55 (2.87)	-1.30 (3.22)	0.300
Diabetes	11 (1.01)	11 (1.01)	1.000	3 (0.88)	6 (1.77)	0.505
Atherosclerotic heart disease	92 (8.45)	92 (8.45)	1.000	32 (9.44)	32 (9.44)	1.000
Atrial fibrillation and flutter	61 (5.60)	51 (4.68)	0.383	22 (6.49)	22 (6.49)	1.000
Heart failure	19 (1.74)	20 (1.84)	1.000	10 (2.95)	13 (3.83)	0.671
Chronic renal failure	5 (0.46)	7 (0.64)	0.772	1 (0.29)	2 (0.59)	1.000
Colchicine use			1.000			1.000
Yes	2 (0.18)	2 (0.18)		0 (0.00)	1 (0.29)	
No	1087 (99.82)	1087 (99.82)		339 (100.00)	338 (99.71)	

Baseline characteristics were compared between participants of non-CHIP and different CHIP statuses using t-test. Chi-squared or exact Fisher test was used for categorical variables. Abbreviations: SD, standard deviation; BMI, body mass index.

Supplementary Table S11: Stratified odds ratios of any overall CHIP associated with different types of AP

Subset	Status	Disease	OR (95%CI)	P value
Sex	Female	AP	0.93(0.83-1.04)	0.180
Sex	Female	UAP	0.79(0.66-0.95)	0.012
Sex	Female	SAP	1.01(0.69-1.47)	0.960
Sex	Male	AP	1.06(0.96-1.16)	0.250
Sex	Male	UAP	1.03(0.91-1.18)	0.630
Sex	Male	SAP	1.27(0.93-1.73)	0.130
Ancestry	White British	AP	0.99(0.92-1.07)	0.820
Ancestry	White British	UAP	0.91(0.81-1.03)	0.130
Ancestry	White British	SAP	1.23(0.95-1.59)	0.120
Ancestry	Other	AP	1.05(0.88-1.25)	0.590
Ancestry	Other	UAP	1.06(0.83-1.35)	0.630
Ancestry	Other	SAP	0.85(0.44-1.62)	0.610
BMI	High	AP	0.99(0.9-1.09)	0.860
BMI	High	UAP	0.92(0.8-1.06)	0.280
BMI	High	SAP	1.17(0.84-1.63)	0.350
BMI	Low	AP	1.01(0.91-1.12)	0.910
BMI	Low	UAP	0.95(0.81-1.11)	0.540
BMI	Low	SAP	1.13(0.8-1.6)	0.480

Subset	Status	Disease	OR (95%CI)	P value
Ever smoked	Ever	AP	1(0.91-1.09)	0.930
Ever smoked	Ever	UAP	0.98(0.86-1.11)	0.730
Ever smoked	Ever	SAP	1.01(0.74-1.38)	0.950
Ever smoked	Never	AP	1.01(0.89-1.14)	0.930
Ever smoked	Never	UAP	0.85(0.7-1.03)	0.100
Ever smoked	Never	SAP	1.42(0.98-2.06)	0.062
Alcohol intake frequency	High	AP	1.03(0.95-1.13)	0.470
Alcohol intake frequency	High	UAP	0.98(0.85-1.12)	0.730
Alcohol intake frequency	High	SAP	1.22(0.91-1.64)	0.180
Alcohol intake frequency	Low	AP	0.95(0.84-1.06)	0.360
Alcohol intake frequency	Low	UAP	0.88(0.74-1.05)	0.150
Alcohol intake frequency	Low	SAP	1.04(0.69-1.57)	0.860
Townsend deprivation index	High	AP	1.08(0.98-1.19)	0.100
Townsend deprivation index	High	UAP	0.96(0.83-1.1)	0.520
Townsend deprivation index	High	SAP	0.97(0.68-1.37)	0.860
Townsend deprivation index	Low	AP	0.9(0.81-1.01)	0.069
Townsend deprivation index	Low	UAP	0.91(0.78-1.07)	0.280
Townsend deprivation index	Low	SAP	1.39(1-1.94)	0.050
Diabetes	Yes	AP	1.14(0.95-1.37)	0.160
Diabetes	Yes	UAP	0.93(0.72-1.21)	0.600
Diabetes	Yes	SAP	1.4(0.76-2.57)	0.280

Subset	Status	Disease	OR (95%CI)	P value
Diabetes	No	AP	0.97(0.9-1.05)	0.510
Diabetes	No	UAP	0.93(0.83-1.05)	0.250
Diabetes	No	SAP	1.12(0.86-1.45)	0.400
Peripheral artery and capillary disease	Yes	AP	0.9(0.66-1.23)	0.500
Peripheral artery and capillary disease	Yes	UAP	0.87(0.57-1.34)	0.540
Peripheral artery and capillary disease	Yes	SAP	0.99(0.35-2.83)	0.980
Peripheral artery and capillary disease	No	AP	1(0.93-1.08)	0.910
Peripheral artery and capillary disease	No	UAP	0.94(0.85-1.05)	0.290
Peripheral artery and capillary disease	No	SAP	1.16(0.91-1.49)	0.230
Atrial fibrillation and flutter	Yes	AP	0.89(0.71-1.12)	0.320
Atrial fibrillation and flutter	Yes	UAP	0.88(0.65-1.21)	0.440
Atrial fibrillation and flutter	Yes	SAP	1.41(0.67-2.96)	0.370
Atrial fibrillation and flutter	No	AP	1.02(0.94-1.1)	0.670
Atrial fibrillation and flutter	No	UAP	0.95(0.85-1.06)	0.370
Atrial fibrillation and flutter	No	SAP	1.13(0.88-1.45)	0.340
Heart failure	Yes	AP	1.17(0.89-1.55)	0.250
Heart failure	Yes	UAP	1.04(0.73-1.48)	0.840
Heart failure	Yes	SAP	1.07(0.42-2.74)	0.890
Heart failure	No	AP	0.99(0.92-1.07)	0.810
Heart failure	No	UAP	0.93(0.83-1.04)	0.210
Heart failure	No	SAP	1.16(0.91-1.49)	0.230

Hypertensive disease	Yes	AP	0.65(0.37-1.12)	0.120
Hypertensive disease	Yes	UAP	0.56(0.26-1.21)	0.140
Hypertensive disease	Yes	SAP	0(0-Inf)	1.000
Hypertensive disease	No	AP	1.01(0.94-1.09)	0.790
Hypertensive disease	No	UAP	0.95(0.86-1.06)	0.370
Hypertensive disease	No	SAP	1.17(0.92-1.49)	0.190
Stroke	Yes	AP	1.12(0.83-1.52)	0.460
Stroke	Yes	UAP	1.11(0.74-1.66)	0.620
Stroke	Yes	SAP	1.78(0.74-4.28)	0.200
Stroke	No	AP	0.99(0.92-1.07)	0.880
Stroke	No	UAP	0.93(0.83-1.04)	0.180
Stroke	No	SAP	1.12(0.87-1.44)	0.370
Chronic renal failure	Yes	AP	0.84(0.36-1.96)	0.690
Chronic renal failure	Yes	UAP	1.13(0.38-3.41)	0.820
Chronic renal failure	Yes	SAP	0(0-Inf)	1.000
Chronic renal failure	No	AP	1(0.93-1.08)	0.950
Chronic renal failure	No	UAP	0.94(0.85-1.04)	0.250
Chronic renal failure	No	SAP	1.16(0.92-1.48)	0.210

Model adjusted for age at recruitment, sex, genetic ancestry, BMI, ever smoked, alcohol intake frequency, Townsend deprivation index, diabetes, atherosclerotic heart disease, hypertensive diseases, atrial fibrillation and flutter, stroke, heart failure, peripheral artery and capillary disease, chronic renal failure. Abbreviations: AP, angina pectoris; UAP, unstable angina pectoris; SAP, stable angina pectoris; BMI, body mass index.

Supplementary Table S12: Stratified odds ratios of large overall CHIP associated with different types of AP

Subset	Status	Disease	OR (95%CI)	P value
Sex	Female	AP	0.83(0.67-1.02)	0.0780
Sex	Female	UAP	0.71(0.5-0.99)	0.0440
Sex	Female	SAP	0.87(0.43-1.77)	0.7100
Sex	Male	AP	0.8(0.68-0.96)	0.0130
Sex	Male	UAP	0.75(0.58-0.96)	0.0240
Sex	Male	SAP	1.22(0.71-2.09)	0.4600
Ancestry	White British	AP	0.82(0.71-0.95)	0.0094
Ancestry	White British	UAP	0.7(0.56-0.88)	0.0020
Ancestry	White British	SAP	1.16(0.73-1.84)	0.5300
Ancestry	Other	AP	0.74(0.54-1.03)	0.0790
Ancestry	Other	UAP	0.84(0.54-1.32)	0.4600
Ancestry	Other	SAP	0.76(0.24-2.4)	0.6400
BMI	High	AP	0.83(0.69-0.99)	0.0390
BMI	High	UAP	0.82(0.64-1.07)	0.1400
BMI	High	SAP	0.76(0.38-1.54)	0.4500
BMI	Low	AP	0.79(0.64-0.96)	0.0160
BMI	Low	UAP	0.6(0.43-0.84)	0.0024
BMI	Low	SAP	1.38(0.81-2.37)	0.2400
Ever smoked	Ever	AP	0.81(0.69-0.95)	0.0110

Subset	Status	Disease	OR (95%CI)	P value
Ever smoked	Ever	UAP	0.72(0.57-0.92)	0.0085
Ever smoked	Ever	SAP	1.1(0.66-1.85)	0.7200
Ever smoked	Never	AP	0.8(0.63-1.01)	0.0640
Ever smoked	Never	UAP	0.73(0.51-1.06)	0.0950
Ever smoked	Never	SAP	1.01(0.48-2.16)	0.9700
Alcohol intake frequency	High	AP	0.77(0.65-0.92)	0.0031
Alcohol intake frequency	High	UAP	0.63(0.48-0.83)	0.0009
Alcohol intake frequency	High	SAP	1.13(0.67-1.89)	0.6500
Alcohol intake frequency	Low	AP	0.87(0.7-1.08)	0.1900
Alcohol intake frequency	Low	UAP	0.88(0.65-1.19)	0.4100
Alcohol intake frequency	Low	SAP	0.98(0.46-2.08)	0.9600
Townsend deprivation index	High	AP	0.84(0.7-1)	0.0510
Townsend deprivation index	High	UAP	0.76(0.58-0.98)	0.0370
Townsend deprivation index	High	SAP	0.95(0.52-1.73)	0.8600
Townsend deprivation index	Low	AP	0.78(0.63-0.95)	0.0150
Townsend deprivation index	Low	UAP	0.69(0.5-0.95)	0.0220
Townsend deprivation index	Low	SAP	1.25(0.68-2.29)	0.4700
Diabetes	Yes	AP	0.79(0.56-1.1)	0.1600
Diabetes	Yes	UAP	0.45(0.25-0.8)	0.0062
Diabetes	Yes	SAP	1.71(0.69-4.27)	0.2500
Diabetes	No	AP	0.81(0.7-0.94)	0.0046

Subset	Status	Disease	OR (95%CI)	P value
Diabetes	No	UAP	0.79(0.64-0.98)	0.0310
Diabetes	No	SAP	0.97(0.6-1.58)	0.9000
Peripheral artery and capillary disease	Yes	AP	0.68(0.39-1.17)	0.1600
Peripheral artery and capillary disease	Yes	UAP	0.67(0.31-1.42)	0.2900
Peripheral artery and capillary disease	Yes	SAP	1.98(0.59-6.69)	0.2700
Peripheral artery and capillary disease	No	AP	0.82(0.71-0.94)	0.0042
Peripheral artery and capillary disease	No	UAP	0.73(0.59-0.9)	0.0036
Peripheral artery and capillary disease	No	SAP	1(0.63-1.58)	1.0000
Atrial fibrillation and flutter	Yes	AP	0.9(0.61-1.32)	0.5800
Atrial fibrillation and flutter	Yes	UAP	0.86(0.51-1.47)	0.5900
Atrial fibrillation and flutter	Yes	SAP	1.06(0.25-4.4)	0.9400
Atrial fibrillation and flutter	No	AP	0.8(0.7-0.93)	0.0024
Atrial fibrillation and flutter	No	UAP	0.72(0.58-0.89)	0.0026
Atrial fibrillation and flutter	No	SAP	1.08(0.69-1.69)	0.7400
Heart failure	Yes	AP	0.91(0.57-1.46)	0.7000
Heart failure	Yes	UAP	0.88(0.48-1.6)	0.6700
Heart failure	Yes	SAP	0.56(0.08-4.18)	0.5700
Heart failure	No	AP	0.81(0.7-0.93)	0.0023
Heart failure	No	UAP	0.72(0.58-0.89)	0.0024
Heart failure	No	SAP	1.12(0.72-1.73)	0.6100
Hypertensive disease	Yes	AP	0.92(0.39-2.18)	0.8500

Subset	Status	Disease	OR (95%CI)	P value
Hypertensive disease	Yes	UAP	0.45(0.1-1.96)	0.2900
Hypertensive disease	Yes	SAP	0(0-Inf)	1.0000
Hypertensive disease	No	AP	0.81(0.71-0.93)	0.0022
Hypertensive disease	No	UAP	0.74(0.6-0.91)	0.0038
Hypertensive disease	No	SAP	1.09(0.71-1.68)	0.6800
Stroke	Yes	AP	0.87(0.5-1.5)	0.6100
Stroke	Yes	UAP	0.8(0.38-1.7)	0.5600
Stroke	Yes	SAP	1.86(0.43-7.96)	0.4000
Stroke	No	AP	0.81(0.7-0.93)	0.0023
Stroke	No	UAP	0.73(0.59-0.89)	0.0027
Stroke	No	SAP	1.04(0.66-1.62)	0.8700
Chronic renal failure	Yes	AP	0.36(0.05-2.83)	0.3300
Chronic renal failure	Yes	UAP	1.05(0.13-8.34)	0.9600
Chronic renal failure	Yes	SAP	0(0-Inf)	1.0000
Chronic renal failure	No	AP	0.81(0.71-0.93)	0.0024
Chronic renal failure	No	UAP	0.73(0.59-0.89)	0.0020
Chronic renal failure	No	SAP	1.08(0.71-1.66)	0.7100

Model adjusted for age at recruitment, sex, genetic ancestry, BMI, ever smoked, alcohol intake frequency, Townsend deprivation index, diabetes, atherosclerotic heart disease, hypertensive diseases, atrial fibrillation and flutter, stroke, heart failure, peripheral artery and capillary disease, chronic renal failure. Abbreviations: AP, angina pectoris; UAP, unstable angina pectoris; SAP, stable angina pectoris; BMI, body mass index.

Supplementary Table S13: Stratified odds ratios of any *DNMT3A* CHIP associated with different types of AP

Subset	Status	Disease	OR (95%CI)	P value
Sex	Female	AP	0.95(0.81-1.13)	0.580
Sex	Female	UAP	0.91(0.7-1.17)	0.450
Sex	Female	SAP	1.28(0.77-2.12)	0.340
Sex	Male	AP	1.08(0.93-1.24)	0.330
Sex	Male	UAP	1.03(0.84-1.27)	0.790
Sex	Male	SAP	1.14(0.68-1.91)	0.630
Ancestry	White British	AP	1.01(0.89-1.14)	0.910
Ancestry	White British	UAP	0.93(0.78-1.12)	0.450
Ancestry	White British	SAP	1.26(0.85-1.87)	0.260
Ancestry	Other	AP	1.11(0.86-1.44)	0.430
Ancestry	Other	UAP	1.21(0.84-1.72)	0.300
Ancestry	Other	SAP	1.05(0.43-2.6)	0.910
BMI	High	AP	1.12(0.97-1.3)	0.120
BMI	High	UAP	1.02(0.82-1.26)	0.880
BMI	High	SAP	1.19(0.7-1.99)	0.520
BMI	Low	AP	0.91(0.77-1.07)	0.240
BMI	Low	UAP	0.92(0.72-1.18)	0.510
BMI	Low	SAP	1.24(0.75-2.05)	0.410
Ever smoked	Ever	AP	1.03(0.9-1.18)	0.650

Subset	Status	Disease	OR (95%CI)	P value
Ever smoked	Ever	UAP	1.04(0.86-1.25)	0.690
Ever smoked	Ever	SAP	1.17(0.75-1.84)	0.490
Ever smoked	Never	AP	1.01(0.83-1.23)	0.940
Ever smoked	Never	UAP	0.84(0.62-1.15)	0.280
Ever smoked	Never	SAP	1.31(0.71-2.41)	0.380
Alcohol intake frequency	High	AP	1.03(0.89-1.18)	0.720
Alcohol intake frequency	High	UAP	0.96(0.78-1.18)	0.690
Alcohol intake frequency	High	SAP	1.11(0.69-1.79)	0.650
Alcohol intake frequency	Low	AP	1.02(0.85-1.22)	0.820
Alcohol intake frequency	Low	UAP	1.02(0.79-1.31)	0.900
Alcohol intake frequency	Low	SAP	1.4(0.8-2.46)	0.240
Townsend deprivation index	High	AP	1.09(0.94-1.26)	0.250
Townsend deprivation index	High	UAP	1.02(0.82-1.26)	0.880
Townsend deprivation index	High	SAP	0.92(0.53-1.6)	0.760
Townsend deprivation index	Low	AP	0.95(0.8-1.12)	0.530
Townsend deprivation index	Low	UAP	0.93(0.73-1.2)	0.600
Townsend deprivation index	Low	SAP	1.62(1-2.61)	0.050
Diabetes	Yes	AP	1.28(0.96-1.72)	0.093
Diabetes	Yes	UAP	1.08(0.72-1.62)	0.730
Diabetes	Yes	SAP	1.04(0.32-3.32)	0.950
Diabetes	No	AP	0.98(0.87-1.11)	0.780

Subset	Status	Disease	OR (95%CI)	P value
Diabetes	No	UAP	0.95(0.8-1.14)	0.600
Diabetes	No	SAP	1.24(0.85-1.82)	0.270
Peripheral artery and capillary disease	Yes	AP	1.05(0.65-1.68)	0.850
Peripheral artery and capillary disease	Yes	UAP	1.03(0.54-1.96)	0.920
Peripheral artery and capillary disease	Yes	SAP	2.03(0.6-6.9)	0.260
Peripheral artery and capillary disease	No	AP	1.02(0.91-1.14)	0.710
Peripheral artery and capillary disease	No	UAP	0.97(0.82-1.15)	0.760
Peripheral artery and capillary disease	No	SAP	1.17(0.8-1.71)	0.430
Atrial fibrillation and flutter	Yes	AP	0.94(0.66-1.32)	0.710
Atrial fibrillation and flutter	Yes	UAP	0.91(0.56-1.48)	0.710
Atrial fibrillation and flutter	Yes	SAP	2.16(0.85-5.48)	0.100
Atrial fibrillation and flutter	No	AP	1.04(0.93-1.17)	0.520
Atrial fibrillation and flutter	No	UAP	0.99(0.84-1.18)	0.950
Atrial fibrillation and flutter	No	SAP	1.12(0.75-1.66)	0.580
Heart failure	Yes	AP	1.03(0.67-1.58)	0.890
Heart failure	Yes	UAP	1.15(0.69-1.93)	0.580
Heart failure	Yes	SAP	0.94(0.22-4)	0.930
Heart failure	No	AP	1.02(0.91-1.15)	0.680
Heart failure	No	UAP	0.97(0.81-1.14)	0.690
Heart failure	No	SAP	1.24(0.85-1.8)	0.260
Hypertensive disease	Yes	AP	0.69(0.31-1.54)	0.370

Subset	Status	Disease	OR (95%CI)	P value
Hypertensive disease	Yes	UAP	0.84(0.31-2.24)	0.730
Hypertensive disease	Yes	SAP	0(0-Inf)	1.000
Hypertensive disease	No	AP	1.03(0.93-1.15)	0.560
Hypertensive disease	No	UAP	0.99(0.84-1.16)	0.860
Hypertensive disease	No	SAP	1.24(0.86-1.78)	0.250
Stroke	Yes	AP	1.03(0.65-1.63)	0.890
Stroke	Yes	UAP	0.87(0.46-1.65)	0.660
Stroke	Yes	SAP	1.96(0.58-6.63)	0.280
Stroke	No	AP	1.03(0.92-1.15)	0.630
Stroke	No	UAP	0.99(0.84-1.18)	0.950
Stroke	No	SAP	1.18(0.8-1.72)	0.400
Chronic renal failure	Yes	AP	1(0.28-3.63)	1.000
Chronic renal failure	Yes	UAP	1.71(0.34-8.52)	0.510
Chronic renal failure	Yes	SAP	0(0-Inf)	1.000
Chronic renal failure	No	AP	1.03(0.92-1.14)	0.660
Chronic renal failure	No	UAP	0.98(0.83-1.15)	0.790
Chronic renal failure	No	SAP	1.23(0.85-1.76)	0.270

Model adjusted for age at recruitment, sex, genetic ancestry, BMI, ever smoked, alcohol intake frequency, Townsend deprivation index, diabetes, atherosclerotic heart disease, hypertensive diseases, atrial fibrillation and flutter, stroke, heart failure, peripheral artery and capillary disease, chronic renal failure. Abbreviations: AP, angina pectoris; UAP, unstable angina pectoris; SAP, stable angina pectoris; BMI, body mass index.

Supplementary Table S14: Stratified odds ratios of large *DNMT3A* CHIP associated with different types of AP

Subset	Status	Disease	OR (95%CI)	P value
Sex	Female	AP	0.88(0.63-1.23)	0.4600
Sex	Female	UAP	0.98(0.6-1.6)	0.9300
Sex	Female	SAP	1.21(0.44-3.28)	0.7100
Sex	Male	AP	0.78(0.57-1.06)	0.1100
Sex	Male	UAP	0.88(0.58-1.33)	0.5400
Sex	Male	SAP	1.38(0.57-3.37)	0.4800
Ancestry	White British	AP	0.83(0.65-1.06)	0.1300
Ancestry	White British	UAP	0.9(0.63-1.27)	0.5400
Ancestry	White British	SAP	1.45(0.72-2.93)	0.3000
Ancestry	Other	AP	0.78(0.44-1.4)	0.4000
Ancestry	Other	UAP	0.98(0.46-2.09)	0.9700
Ancestry	Other	SAP	0.81(0.11-5.87)	0.8300
BMI	High	AP	0.93(0.68-1.26)	0.6200
BMI	High	UAP	1.12(0.75-1.68)	0.5700
BMI	High	SAP	0.61(0.15-2.45)	0.4800
BMI	Low	AP	0.71(0.51-1)	0.0480
BMI	Low	UAP	0.69(0.41-1.14)	0.1500
BMI	Low	SAP	1.96(0.92-4.19)	0.0820
Ever smoked	Ever	AP	0.85(0.65-1.11)	0.2300

Subset	Status	Disease	OR (95%CI)	P value
Ever smoked	Ever	UAP	0.86(0.59-1.26)	0.4300
Ever smoked	Ever	SAP	1.72(0.85-3.48)	0.1300
Ever smoked	Never	AP	0.75(0.48-1.16)	0.1900
Ever smoked	Never	UAP	1.06(0.61-1.86)	0.8400
Ever smoked	Never	SAP	0.48(0.07-3.46)	0.4700
Alcohol intake frequency	High	AP	0.69(0.51-0.93)	0.0160
Alcohol intake frequency	High	UAP	0.66(0.42-1.04)	0.0710
Alcohol intake frequency	High	SAP	1.33(0.59-3)	0.4900
Alcohol intake frequency	Low	AP	1.06(0.75-1.5)	0.7400
Alcohol intake frequency	Low	UAP	1.39(0.89-2.18)	0.1500
Alcohol intake frequency	Low	SAP	1.32(0.42-4.16)	0.6400
Townsend deprivation index	High	AP	0.81(0.59-1.1)	0.1800
Townsend deprivation index	High	UAP	0.98(0.65-1.48)	0.9400
Townsend deprivation index	High	SAP	0.79(0.25-2.48)	0.6900
Townsend deprivation index	Low	AP	0.83(0.59-1.16)	0.2800
Townsend deprivation index	Low	UAP	0.83(0.5-1.36)	0.4600
Townsend deprivation index	Low	SAP	2.01(0.89-4.55)	0.0940
Diabetes	Yes	AP	0.79(0.43-1.43)	0.4300
Diabetes	Yes	UAP	0.9(0.43-1.91)	0.7900
Diabetes	Yes	SAP	1.06(0.14-7.8)	0.9500
Diabetes	No	AP	0.83(0.65-1.06)	0.1300

Subset	Status	Disease	OR (95%CI)	P value
Diabetes	No	UAP	0.91(0.65-1.29)	0.6100
Diabetes	No	SAP	1.37(0.68-2.76)	0.3800
Peripheral artery and capillary disease	Yes	AP	1.23(0.55-2.77)	0.6200
Peripheral artery and capillary disease	Yes	UAP	0.95(0.31-2.92)	0.9300
Peripheral artery and capillary disease	Yes	SAP	5.67(1.53-21.01)	0.0093
Peripheral artery and capillary disease	No	AP	0.8(0.63-1.01)	0.0600
Peripheral artery and capillary disease	No	UAP	0.91(0.66-1.27)	0.5800
Peripheral artery and capillary disease	No	SAP	0.96(0.43-2.15)	0.9200
Atrial fibrillation and flutter	Yes	AP	0.93(0.49-1.77)	0.8200
Atrial fibrillation and flutter	Yes	UAP	1.31(0.6-2.83)	0.5000
Atrial fibrillation and flutter	Yes	SAP	2.83(0.66-12.21)	0.1600
Atrial fibrillation and flutter	No	AP	0.82(0.64-1.04)	0.1100
Atrial fibrillation and flutter	No	UAP	0.88(0.62-1.24)	0.4600
Atrial fibrillation and flutter	No	SAP	1.14(0.54-2.42)	0.7300
Heart failure	Yes	AP	0.66(0.28-1.53)	0.3300
Heart failure	Yes	UAP	1.15(0.46-2.83)	0.7700
Heart failure	Yes	SAP	0(0-Inf)	0.9900
Heart failure	No	AP	0.84(0.66-1.06)	0.1400
Heart failure	No	UAP	0.89(0.64-1.25)	0.5200
Heart failure	No	SAP	1.46(0.75-2.83)	0.2600
Hypertensive disease	Yes	AP	0.62(0.12-3.15)	0.5700

Subset	Status	Disease	OR (95%CI)	P value
Hypertensive disease	Yes	UAP	0.52(0.06-4.43)	0.5500
Hypertensive disease	Yes	SAP	0(0-Inf)	1.0000
Hypertensive disease	No	AP	0.83(0.66-1.04)	0.1100
Hypertensive disease	No	UAP	0.93(0.68-1.28)	0.6700
Hypertensive disease	No	SAP	1.35(0.7-2.62)	0.3700
Stroke	Yes	AP	1.05(0.47-2.34)	0.9100
Stroke	Yes	UAP	1.38(0.52-3.65)	0.5200
Stroke	Yes	SAP	5.64(1.24-25.6)	0.0250
Stroke	No	AP	0.81(0.64-1.03)	0.0860
Stroke	No	UAP	0.89(0.64-1.25)	0.5100
Stroke	No	SAP	1.11(0.52-2.35)	0.7900
Chronic renal failure	Yes	AP	0(0-Inf)	0.9800
Chronic renal failure	Yes	UAP	0(0-Inf)	0.9900
Chronic renal failure	Yes	SAP	0(0-Inf)	1.0000
Chronic renal failure	No	AP	0.82(0.66-1.03)	0.0960
Chronic renal failure	No	UAP	0.92(0.67-1.26)	0.6000
Chronic renal failure	No	SAP	1.33(0.69-2.59)	0.4000

Model adjusted for age at recruitment, sex, genetic ancestry, BMI, ever smoked, alcohol intake frequency, Townsend deprivation index, diabetes, atherosclerotic heart disease, hypertensive diseases, atrial fibrillation and flutter, stroke, heart failure, peripheral artery and capillary disease, chronic renal failure. Abbreviations: AP, angina pectoris; UAP, unstable angina pectoris; SAP, stable angina pectoris; BMI, body mass index.

Supplementary Table S15: Stratified odds ratios of any *TET2* CHIP associated with different types of AP

Subset	Status	Disease	OR (95%CI)	P value
Sex	Female	AP	0.85(0.64-1.13)	0.2700
Sex	Female	UAP	0.69(0.42-1.11)	0.1200
Sex	Female	SAP	1.58(0.74-3.36)	0.2400
Sex	Male	AP	0.95(0.76-1.18)	0.6400
Sex	Male	UAP	0.88(0.63-1.22)	0.4400
Sex	Male	SAP	1.53(0.79-2.99)	0.2100
Ancestry	White British	AP	0.9(0.74-1.09)	0.2700
Ancestry	White British	UAP	0.79(0.59-1.07)	0.1200
Ancestry	White British	SAP	1.64(0.96-2.81)	0.0680
Ancestry	Other	AP	0.98(0.63-1.53)	0.9300
Ancestry	Other	UAP	0.9(0.46-1.73)	0.7400
Ancestry	Other	SAP	1.13(0.28-4.64)	0.8700
BMI	High	AP	0.81(0.63-1.04)	0.1100
BMI	High	UAP	0.61(0.4-0.92)	0.0180
BMI	High	SAP	1.58(0.78-3.19)	0.2100
BMI	Low	AP	1(0.79-1.29)	0.9700
BMI	Low	UAP	1.04(0.73-1.48)	0.8400
BMI	Low	SAP	1.49(0.74-3.03)	0.2700
Ever smoked	Ever	AP	0.82(0.65-1.02)	0.0800

Subset	Status	Disease	OR (95%CI)	P value
Ever smoked	Ever	UAP	0.72(0.51-1.01)	0.0580
Ever smoked	Ever	SAP	1.03(0.48-2.18)	0.9400
Ever smoked	Never	AP	1.1(0.83-1.46)	0.5200
Ever smoked	Never	UAP	1.01(0.65-1.57)	0.9700
Ever smoked	Never	SAP	2.55(1.3-5.01)	0.0065
Alcohol intake frequency	High	AP	0.9(0.72-1.12)	0.3300
Alcohol intake frequency	High	UAP	0.84(0.6-1.17)	0.3000
Alcohol intake frequency	High	SAP	1.78(1-3.18)	0.0500
Alcohol intake frequency	Low	AP	0.94(0.7-1.26)	0.7000
Alcohol intake frequency	Low	UAP	0.76(0.48-1.21)	0.2400
Alcohol intake frequency	Low	SAP	1.17(0.43-3.17)	0.7500
Townsend deprivation index	High	AP	1.02(0.81-1.29)	0.8700
Townsend deprivation index	High	UAP	0.9(0.63-1.29)	0.5600
Townsend deprivation index	High	SAP	1.69(0.87-3.3)	0.1200
Townsend deprivation index	Low	AP	0.8(0.61-1.05)	0.1000
Townsend deprivation index	Low	UAP	0.72(0.47-1.08)	0.1100
Townsend deprivation index	Low	SAP	1.4(0.66-2.99)	0.3800
Diabetes	Yes	AP	0.89(0.56-1.41)	0.6200
Diabetes	Yes	UAP	0.6(0.29-1.23)	0.1600
Diabetes	Yes	SAP	1.37(0.33-5.64)	0.6700
Diabetes	No	AP	0.91(0.75-1.1)	0.3300

Subset	Status	Disease	OR (95%CI)	P value
Diabetes	No	UAP	0.85(0.63-1.13)	0.2700
Diabetes	No	SAP	1.56(0.92-2.67)	0.1000
Peripheral artery and capillary disease	Yes	AP	0.88(0.4-1.94)	0.7500
Peripheral artery and capillary disease	Yes	UAP	0.43(0.1-1.81)	0.2500
Peripheral artery and capillary disease	Yes	SAP	0(0-Inf)	0.9900
Peripheral artery and capillary disease	No	AP	0.91(0.76-1.09)	0.3100
Peripheral artery and capillary disease	No	UAP	0.84(0.63-1.1)	0.2000
Peripheral artery and capillary disease	No	SAP	1.64(1-2.71)	0.0520
Atrial fibrillation and flutter	Yes	AP	0.64(0.34-1.18)	0.1500
Atrial fibrillation and flutter	Yes	UAP	0.34(0.1-1.07)	0.0660
Atrial fibrillation and flutter	Yes	SAP	2.18(0.52-9.13)	0.2900
Atrial fibrillation and flutter	No	AP	0.94(0.78-1.13)	0.5000
Atrial fibrillation and flutter	No	UAP	0.87(0.66-1.15)	0.3400
Atrial fibrillation and flutter	No	SAP	1.48(0.87-2.52)	0.1500
Heart failure	Yes	AP	1.22(0.64-2.31)	0.5500
Heart failure	Yes	UAP	0.45(0.14-1.46)	0.1800
Heart failure	Yes	SAP	3.47(0.8-15.11)	0.0980
Heart failure	No	AP	0.89(0.74-1.07)	0.2100
Heart failure	No	UAP	0.84(0.64-1.11)	0.2200
Heart failure	No	SAP	1.45(0.85-2.47)	0.1700
Hypertensive disease	Yes	AP	1.21(0.39-3.79)	0.7400

Subset	Status	Disease	OR (95%CI)	P value
Hypertensive disease	Yes	UAP	0.52(0.07-4.14)	0.5400
Hypertensive disease	Yes	SAP	0(0-Inf)	1.0000
Hypertensive disease	No	AP	0.91(0.76-1.08)	0.2800
Hypertensive disease	No	UAP	0.82(0.63-1.08)	0.1600
Hypertensive disease	No	SAP	1.58(0.96-2.6)	0.0740
Stroke	Yes	AP	1.62(0.82-3.2)	0.1700
Stroke	Yes	UAP	1.67(0.69-4.05)	0.2600
Stroke	Yes	SAP	2.55(0.34-19.44)	0.3700
Stroke	No	AP	0.88(0.73-1.06)	0.1700
Stroke	No	UAP	0.76(0.57-1.01)	0.0620
Stroke	No	SAP	1.53(0.91-2.56)	0.1100
Chronic renal failure	Yes	AP	0(0-Inf)	0.9800
Chronic renal failure	Yes	UAP	0(0-Inf)	0.9900
Chronic renal failure	Yes	SAP	0(0-Inf)	1.0000
Chronic renal failure	No	AP	0.91(0.77-1.09)	0.3200
Chronic renal failure	No	UAP	0.81(0.62-1.07)	0.1300
Chronic renal failure	No	SAP	1.56(0.95-2.57)	0.0810

Model adjusted for age at recruitment, sex, genetic ancestry, BMI, ever smoked, alcohol intake frequency, Townsend deprivation index, diabetes, atherosclerotic heart disease, hypertensive diseases, atrial fibrillation and flutter, stroke, heart failure, peripheral artery and capillary disease, chronic renal failure. Abbreviations: AP, angina pectoris; UAP, unstable angina pectoris; SAP, stable angina pectoris; BMI, body mass index.

Supplementary Table S16: Stratified odds ratios of large *TET2* CHIP associated with different types of AP

Subset	Status	Disease	OR (95%CI)	P value
Sex	Female	AP	0.36(0.15-0.82)	0.0160
Sex	Female	UAP	0.3(0.07-1.22)	0.0930
Sex	Female	SAP	0.94(0.13-6.81)	0.9500
Sex	Male	AP	0.69(0.43-1.13)	0.1400
Sex	Male	UAP	0.56(0.26-1.2)	0.1400
Sex	Male	SAP	1.29(0.32-5.22)	0.7300
Ancestry	White British	AP	0.63(0.41-0.97)	0.0370
Ancestry	White British	UAP	0.54(0.28-1.07)	0.0760
Ancestry	White British	SAP	0.92(0.23-3.72)	0.9100
Ancestry	Other	AP	0.16(0.02-1.15)	0.0690
Ancestry	Other	UAP	0(0-1.67707488475029e+141)	0.9400
Ancestry	Other	SAP	2.49(0.33-18.71)	0.3800
BMI	High	AP	0.43(0.22-0.82)	0.0110
BMI	High	UAP	0.51(0.21-1.25)	0.1400
BMI	High	SAP	0.78(0.11-5.58)	0.8000
BMI	Low	AP	0.69(0.4-1.2)	0.1900
BMI	Low	UAP	0.41(0.15-1.13)	0.0850
BMI	Low	SAP	1.39(0.34-5.69)	0.6500
Ever smoked	Ever	AP	0.59(0.36-0.96)	0.0350

Subset	Status	Disease	OR (95%CI)	P value
Ever smoked	Ever	UAP	0.53(0.24-1.13)	0.1000
Ever smoked	Ever	SAP	1.12(0.28-4.55)	0.8700
Ever smoked	Never	AP	0.51(0.23-1.13)	0.0960
Ever smoked	Never	UAP	0.34(0.08-1.42)	0.1400
Ever smoked	Never	SAP	1.2(0.17-8.71)	0.8600
Alcohol intake frequency	High	AP	0.56(0.34-0.93)	0.0250
Alcohol intake frequency	High	UAP	0.3(0.11-0.81)	0.0180
Alcohol intake frequency	High	SAP	1.66(0.53-5.23)	0.3900
Alcohol intake frequency	Low	AP	0.57(0.27-1.21)	0.1400
Alcohol intake frequency	Low	UAP	0.88(0.35-2.21)	0.7800
Alcohol intake frequency	Low	SAP	0(0-7.64182037712414e+177)	0.9600
Townsend deprivation index	High	AP	0.61(0.35-1.07)	0.0860
Townsend deprivation index	High	UAP	0.39(0.14-1.08)	0.0700
Townsend deprivation index	High	SAP	2.3(0.73-7.29)	0.1600
Townsend deprivation index	Low	AP	0.52(0.28-0.98)	0.0430
Townsend deprivation index	Low	UAP	0.57(0.23-1.41)	0.2200
Townsend deprivation index	Low	SAP	0(0-1.34099664744629e+211)	0.9600
Diabetes	Yes	AP	0.62(0.22-1.78)	0.3800
Diabetes	Yes	UAP	0.32(0.04-2.36)	0.2600
Diabetes	Yes	SAP	6.5(1.5-28.16)	0.0120
Diabetes	No	AP	0.55(0.35-0.86)	0.0092

Subset	Status	Disease	OR (95%CI)	P value
Diabetes	No	UAP	0.49(0.24-1)	0.0510
Diabetes	No	SAP	0.43(0.06-3.09)	0.4000
Peripheral artery and capillary disease	Yes	AP	0(0-2.23921097482952e+241)	0.9600
Peripheral artery and capillary disease	Yes	UAP	0(0-2.43153112483654e+244)	0.9700
Peripheral artery and capillary disease	Yes	SAP	0(0-Inf)	0.9900
Peripheral artery and capillary disease	No	AP	0.6(0.4-0.92)	0.0180
Peripheral artery and capillary disease	No	UAP	0.5(0.26-0.99)	0.0450
Peripheral artery and capillary disease	No	SAP	1.23(0.39-3.86)	0.7200
Atrial fibrillation and flutter	Yes	AP	0.43(0.1-1.87)	0.2600
Atrial fibrillation and flutter	Yes	UAP	0(0-2.22624211754641e+156)	0.9500
Atrial fibrillation and flutter	Yes	SAP	0(0-Inf)	0.9800
Atrial fibrillation and flutter	No	AP	0.57(0.37-0.88)	0.0120
Atrial fibrillation and flutter	No	UAP	0.53(0.27-1.03)	0.0620
Atrial fibrillation and flutter	No	SAP	1.24(0.39-3.88)	0.7200
Heart failure	Yes	AP	0.61(0.13-2.86)	0.5300
Heart failure	Yes	UAP	0(0-9.13105713265745e+271)	0.9700
Heart failure	Yes	SAP	0(0-Inf)	1.0000
Heart failure	No	AP	0.56(0.36-0.87)	0.0094
Heart failure	No	UAP	0.52(0.27-1.03)	0.0590
Heart failure	No	SAP	1.24(0.4-3.89)	0.7100
Hypertensive disease	Yes	AP	1.07(0.12-9.81)	0.9600

Subset	Status	Disease	OR (95%CI)	P value
Hypertensive disease	Yes	UAP	0(0-Inf)	0.9800
Hypertensive disease	Yes	SAP	0(0-Inf)	1.0000
Hypertensive disease	No	AP	0.56(0.36-0.85)	0.0072
Hypertensive disease	No	UAP	0.49(0.25-0.96)	0.0380
Hypertensive disease	No	SAP	1.17(0.37-3.67)	0.7900
Stroke	Yes	AP	0.88(0.23-3.33)	0.8500
Stroke	Yes	UAP	0(0-2.22493363851361e+300)	0.9700
Stroke	Yes	SAP	0(0-Inf)	0.9800
Stroke	No	AP	0.55(0.35-0.85)	0.0073
Stroke	No	UAP	0.53(0.27-1.04)	0.0660
Stroke	No	SAP	1.27(0.4-3.97)	0.6800
Chronic renal failure	Yes	AP	0(0-Inf)	0.9800
Chronic renal failure	Yes	UAP	0(0-Inf)	0.9800
Chronic renal failure	Yes	SAP	0(0-Inf)	1.0000
Chronic renal failure	No	AP	0.57(0.38-0.87)	0.0087
Chronic renal failure	No	UAP	0.47(0.24-0.93)	0.0300
Chronic renal failure	No	SAP	1.17(0.37-3.66)	0.7900

Model adjusted for age at recruitment, sex, genetic ancestry, BMI, ever smoked, alcohol intake frequency, Townsend deprivation index, diabetes, atherosclerotic heart disease, hypertensive diseases, atrial fibrillation and flutter, stroke, heart failure, peripheral artery and capillary disease, chronic renal failure. Abbreviations: AP, angina pectoris; UAP, unstable angina pectoris; SAP, stable angina pectoris; BMI, body mass index.

Supplementary Table S17: Instrumental variables used in Mendelian randomization analysis for causal association of any CHIP on each AP type

CHIP gene	rsID	Effect allele	Other allele	Beta in exposure	SE in exposure	EAF in exposure	P value in exposure	Beta in outcome	SE in outcome	EAF in outcome	P value in outcome	F statistics
Overall	rs10069690	T	C	0.053	0.011	0.266	1.23×10-6	-0.022	0.029	0.258	0.440	23.215
Overall	rs13174919	C	G	-0.052	0.011	0.258	5.08×10-6	0.045	0.028	0.256	0.110	22.347
Overall	rs2736100	A	C	-0.105	0.010	0.495	8.03×10-27	0.078	0.025	0.497	0.002	110.250
Overall	rs2736108	T	C	0.069	0.011	0.305	5.89×10-11	-0.007	0.027	0.304	0.789	39.347
Overall	rs2853676	T	C	0.064	0.011	0.272	4.60×10-9	-0.009	0.028	0.270	0.753	33.851
Overall	rs2853677	G	A	0.105	0.010	0.425	3.10×10-26	-0.066	0.025	0.423	0.009	110.250
Overall	rs37004	T	C	-0.078	0.012	0.225	8.86×10-11	0.012	0.029	0.236	0.677	42.250
Overall	rs55892935	T	C	-0.121	0.027	0.041	6.25×10-6	0.093	0.061	0.044	0.125	20.084
Overall	rs6469211	C	T	-0.048	0.010	0.338	4.33×10-6	0.007	0.026	0.348	0.779	23.040
Overall	rs72845145	A	G	-0.084	0.018	0.086	4.89×10-6	-0.002	0.047	0.077	0.964	21.778
Overall	rs74953683	G	A	-0.156	0.035	0.023	8.95×10-6	-0.029	0.083	0.025	0.729	19.866
Overall	rs7631792	C	T	-0.045	0.010	0.476	5.48×10-6	0.008	0.025	0.470	0.762	20.250
Overall	rs76338240	C	T	-0.120	0.027	0.038	7.33×10-6	-0.073	0.065	0.041	0.263	19.753
Overall	rs962454	C	A	-0.046	0.010	0.441	2.89×10-6	-0.037	0.025	0.452	0.143	21.160
DNMT3A	rs10069690	T	C	0.101	0.017	0.266	3.44×10-9	-0.022	0.029	0.258	0.440	35.298
DNMT3A	rs10131341	C	A	0.084	0.019	0.198	9.75×10-6	0.019	0.032	0.185	0.562	19.546
DNMT3A	rs1040285	A	G	0.096	0.018	0.210	1.71×10-7	0.083	0.031	0.199	0.007	28.444
DNMT3A	rs10890839	A	C	0.081	0.016	0.414	1.83×10-7	-0.022	0.026	0.408	0.381	25.629
DNMT3A	rs1136410	G	A	-0.135	0.022	0.152	1.49×10-9	-0.071	0.035	0.153	0.045	37.655
DNMT3A	rs115494926	T	C	-0.224	0.043	0.039	2.29×10-7	0.046	0.062	0.040	0.455	27.137
DNMT3A	rs116597408	G	A	0.208	0.040	0.032	1.49×10-7	-0.092	0.073	0.033	0.207	27.040

CHIP gene	rsID	Effect allele	Other allele	Beta in exposure	SE in exposure	EAF in exposure	P value in exposure	Beta in outcome	SE in outcome	EAF in outcome	P value in outcome	F statistics
DNMT3A	rs117019271	C	T	0.212	0.044	0.025	1.67×10-6	0.020	0.076	0.027	0.792	23.215
DNMT3A	rs117044651	A	G	0.321	0.069	0.009	2.86×10-6	-0.337	0.150	0.009	0.025	21.643
DNMT3A	rs11708118	A	G	0.091	0.017	0.256	1.39×10-7	0.010	0.028	0.264	0.711	28.654
DNMT3A	rs13174919	C	G	-0.081	0.018	0.258	5.64×10-6	0.045	0.028	0.256	0.110	20.250
DNMT3A	rs2048485	C	T	0.121	0.021	0.145	1.46×10-8	0.025	0.037	0.140	0.491	33.200
DNMT3A	rs2270860	T	C	0.078	0.016	0.308	2.47×10-6	-0.010	0.027	0.293	0.705	23.766
DNMT3A	rs2522490	A	C	0.094	0.016	0.385	2.78×10-9	-0.011	0.026	0.370	0.663	34.516
DNMT3A	rs2615000	T	C	0.092	0.021	0.152	8.17×10-6	-0.012	0.035	0.153	0.720	19.193
DNMT3A	rs2647264	G	A	0.125	0.027	0.080	3.17×10-6	0.029	0.046	0.082	0.526	21.433
DNMT3A	rs27064	T	C	0.097	0.022	0.135	8.26×10-6	0.004	0.037	0.127	0.915	19.440
DNMT3A	rs2736100	A	C	-0.207	0.015	0.496	6.40×10-41	0.078	0.025	0.497	0.002	190.440
DNMT3A	rs2736108	T	C	0.130	0.016	0.305	1.41×10-15	-0.007	0.027	0.304	0.789	66.016
DNMT3A	rs2853676	T	C	0.110	0.017	0.272	6.33×10-11	-0.009	0.028	0.270	0.753	41.869
DNMT3A	rs2853677	G	A	0.189	0.015	0.424	1.10×10-34	-0.066	0.025	0.423	0.009	158.760
DNMT3A	rs37004	T	C	-0.126	0.019	0.226	3.49×10-11	0.012	0.029	0.236	0.677	43.978
DNMT3A	rs61318425	C	T	-0.107	0.017	0.295	4.94×10-10	-0.064	0.028	0.300	0.021	39.616
DNMT3A	rs61914383	C	T	0.136	0.026	0.086	1.39×10-7	-0.076	0.046	0.088	0.095	27.361
DNMT3A	rs61914392	G	A	0.158	0.028	0.070	1.82×10-8	-0.052	0.050	0.070	0.300	31.842
DNMT3A	rs6533163	A	G	-0.071	0.015	0.454	5.04×10-6	-0.042	0.025	0.458	0.095	22.404
DNMT3A	rs7631792	C	T	-0.093	0.015	0.477	1.75×10-9	0.008	0.025	0.470	0.762	38.440
DNMT3A	rs7762767	C	T	0.125	0.028	0.076	1.00×10-5	0.014	0.048	0.076	0.770	19.930
DNMT3A	rs9374080	C	T	-0.110	0.016	0.447	1.38×10-12	-0.040	0.025	0.460	0.108	47.266

CHIP gene	rsID	Effect allele	Other allele	Beta in exposure	SE in exposure	EAF in exposure	P value in exposure	Beta in outcome	SE in outcome	EAF in outcome	P value in outcome	F statistics
DNMT3A	rs939336	A	G	-0.070	0.016	0.440	5.99×10-6	-0.001	0.025	0.448	0.957	19.141
DNMT3A	rs9418459	A	C	-0.071	0.016	0.404	5.81×10-6	-0.033	0.025	0.412	0.198	19.691
TET2	rs117363469	A	G	0.323	0.072	0.021	7.49×10-6	-0.079	0.090	0.022	0.378	20.125
TET2	rs117791472	G	A	0.275	0.060	0.032	3.90×10-6	0.066	0.068	0.033	0.332	21.007
TET2	rs12759306	A	C	0.130	0.029	0.203	6.71×10-6	0.002	0.031	0.203	0.945	20.095
TET2	rs2853677	G	A	0.125	0.024	0.423	2.03×10-7	-0.066	0.025	0.423	0.009	27.127
TET2	rs7726159	A	C	0.161	0.025	0.327	8.75×10-11	-0.068	0.027	0.328	0.012	41.474
TET2	rs78750274	T	C	0.368	0.073	0.019	4.52×10-7	0.188	0.080	0.021	0.019	25.413
ASXL1	rs112143127	T	C	0.552	0.124	0.027	8.54×10-6	-0.055	0.078	0.028	0.481	19.817
ASXL1	rs112283783	C	A	0.481	0.108	0.040	7.83×10-6	-0.005	0.062	0.042	0.938	19.835
ASXL1	rs114073300	A	C	0.619	0.134	0.022	3.60×10-6	0.059	0.081	0.023	0.466	21.339
ASXL1	rs114530628	G	A	0.627	0.135	0.021	3.16×10-6	0.122	0.080	0.022	0.126	21.571
ASXL1	rs116181573	T	C	0.834	0.175	0.010	1.78×10-6	-0.056	0.125	0.011	0.655	22.712
ASXL1	rs117270864	G	A	0.833	0.172	0.011	1.25×10-6	0.115	0.114	0.012	0.311	23.455
ASXL1	rs56387343	A	G	0.567	0.128	0.024	9.27×10-6	0.102	0.075	0.026	0.173	19.622
ASXL1	rs61938994	C	T	0.508	0.108	0.038	2.45×10-6	-0.034	0.064	0.040	0.601	22.125
ASXL1	rs6948538	A	G	-0.259	0.056	0.393	3.13×10-6	0.011	0.026	0.393	0.659	21.391
ASXL1	rs72966733	G	A	0.571	0.118	0.030	1.23×10-6	0.013	0.071	0.031	0.851	23.416
ASXL1	rs78136542	A	G	0.500	0.112	0.035	8.56×10-6	-0.030	0.067	0.037	0.650	19.930
ASXL1	rs79607292	T	G	0.717	0.145	0.017	7.20×10-7	-0.063	0.096	0.018	0.510	24.451

Abbreviations: AP, angina pectoris; UAP, unstable angina pectoris; SAP, stable angina pectoris; EAF, effect allele frequency.

Supplementary Table S18: Instrumental variables used in Mendelian randomization analysis for causal association of large CHIP on each AP type

CHIP gene	rsID	Effect allele	Other allele	Beta in exposure	SE in exposure	EAF in exposure	P value in exposure	Beta in outcome	SE in outcome	EAF in outcome	P value in outcome	F statistics
Overall	rs116957998	A	G	0.212	0.045	0.033	3.00×10-6	-0.131	0.075	0.033	0.083	22.194
Overall	rs12103149	A	G	0.139	0.027	0.098	3.35×10-7	0.032	0.042	0.093	0.454	26.503
Overall	rs13090261	A	C	0.098	0.022	0.183	7.70×10-6	0.057	0.031	0.188	0.070	19.842
Overall	rs17461	A	C	0.131	0.027	0.100	1.60×10-6	0.025	0.041	0.098	0.554	23.540
Overall	rs2853676	T	C	0.087	0.019	0.272	5.48×10-6	-0.009	0.028	0.270	0.753	20.966
Overall	rs2853677	G	A	0.126	0.017	0.424	4.26×10-13	-0.066	0.025	0.423	0.009	54.934
Overall	rs2874282	A	G	-0.147	0.018	0.431	3.68×10-16	-0.027	0.026	0.441	0.285	66.694
Overall	rs347845	C	T	-0.089	0.020	0.280	7.30×10-6	-0.036	0.029	0.263	0.203	19.80
Overall	rs56192173	A	G	0.174	0.038	0.046	5.22×10-6	0.024	0.064	0.039	0.704	20.966
Overall	rs7726159	A	C	0.132	0.018	0.327	2.61×10-13	-0.068	0.027	0.328	0.012	53.777
Overall	rs80339021	G	C	0.526	0.116	0.039	6.18×10-6	0.131	0.161	0.039	0.417	20.561
Overall	rs840016	T	C	-0.081	0.018	0.395	6.73×10-6	-0.027	0.025	0.407	0.295	20.25
DNMT3A	rs1136410	G	A	-0.217	0.044	0.152	9.89×10-7	-0.071	0.035	0.153	0.045	24.322
DNMT3A	rs12823940	C	T	0.331	0.074	0.030	8.09×10-6	-0.006	0.072	0.032	0.938	20.007
DNMT3A	rs1799986	T	C	0.204	0.039	0.146	1.69×10-7	-0.046	0.036	0.148	0.197	27.360
DNMT3A	rs2048485	C	T	0.210	0.040	0.145	1.80×10-7	0.025	0.037	0.140	0.491	27.562
DNMT3A	rs2522490	A	C	0.139	0.030	0.384	4.57×10-6	-0.011	0.026	0.370	0.663	21.467
DNMT3A	rs2736100	A	C	-0.228	0.030	0.497	2.08×10-14	0.078	0.025	0.497	0.002	57.760
DNMT3A	rs2736108	T	C	0.160	0.031	0.305	3.33×10-7	-0.007	0.027	0.304	0.789	26.638
DNMT3A	rs2853677	G	A	0.222	0.030	0.423	6.58×10-14	-0.066	0.025	0.423	0.009	54.760
DNMT3A	rs71521808	A	C	0.363	0.081	0.024	6.51×10-6	0.120	0.075	0.025	0.110	20.083

CHIP gene	rsID	Effect allele	Other allele	Beta in exposure	SE in exposure	EAF in exposure	P value in exposure	Beta in outcome	SE in outcome	EAF in outcome	P value in outcome	F statistics
DNMT3A	rs719737	C	T	-0.213	0.047	0.133	6.22×10-6	-0.024	0.037	0.131	0.523	20.538
DNMT3A	rs77398655	G	T	-0.390	0.084	0.045	3.76×10-6	0.005	0.058	0.048	0.933	21.556
DNMT3A	rs77914690	G	C	0.760	0.166	0.046	4.87×10-6	0.217	0.144	0.046	0.131	20.960
DNMT3A	rs9374080	C	T	-0.172	0.030	0.448	1.15×10-8	-0.040	0.025	0.460	0.108	32.871
TET2	rs117217874	A	G	0.537	0.120	0.029	8.46×10-6	-0.131	0.078	0.030	0.092	20.025
TET2	rs12317348	G	A	0.312	0.069	0.134	6.15×10-6	-0.088	0.037	0.138	0.018	20.446
TET2	rs17380425	T	C	0.623	0.139	0.020	7.97×10-6	0.053	0.087	0.020	0.544	20.088
TET2	rs1808542	A	G	-0.267	0.059	0.329	6.25×10-6	-0.046	0.027	0.325	0.088	20.479
TET2	rs28367616	G	A	4.401	0.778	2.29×10-5	1.56×10-8	1.613	1.468	1.83×10-5	0.272	31.999
TET2	rs2853677	G	A	0.252	0.052	0.423	1.43×10-6	-0.066	0.025	0.423	0.009	23.485
TET2	rs74751128	T	G	0.711	0.142	0.019	5.46×10-7	-0.127	0.107	0.015	0.237	25.070
TET2	rs74883244	G	A	0.681	0.154	0.015	9.51×10-6	-0.081	0.103	0.016	0.430	19.554
TET2	rs74965890	A	T	0.675	0.149	0.017	5.74×10-6	0.026	0.097	0.016	0.793	20.522
TET2	rs75268734	A	G	0.561	0.121	0.028	3.65×10-6	0.067	0.071	0.029	0.344	21.495
TET2	rs75720165	A	G	0.753	0.169	0.012	8.53×10-6	-0.013	0.113	0.013	0.910	19.852
TET2	rs7726159	A	C	0.331	0.053	0.326	4.70×10-10	-0.068	0.027	0.328	0.012	39.003
TET2	rs7739188	G	A	0.576	0.117	0.030	8.81×10-7	-0.156	0.077	0.031	0.043	24.236
ASXL1	rs114661498	A	G	0.854	0.192	0.027	8.84×10-6	-0.078	0.080	0.027	0.329	19.783
ASXL1	rs117362476	A	G	0.967	0.215	0.020	7.15×10-6	-0.130	0.095	0.020	0.172	20.229
ASXL1	rs117477647	C	T	5.022	1.064	1.76×10-5	2.37×10-6	1.593	1.470	1.49×10-5	0.278	22.277

CHIP gene	rsID	Effect allele	Other allele	Beta in exposure	SE in exposure	EAF in exposure	P value in exposure	Beta in outcome	SE in outcome	EAF in outcome	P value in outcome	F statistics
ASXL1	rs117690000	T	A	1.934	0.422	0.016	4.71×10-6	-0.455	0.322	0.016	0.158	21.003
ASXL1	rs11791740	T	C	-0.494	0.108	0.336	5.23×10-6	-0.010	0.026	0.354	0.700	20.922
ASXL1	rs11854976	G	T	0.686	0.154	0.055	8.69×10-6	-0.010	0.055	0.055	0.860	19.842
ASXL1	rs12369853	A	G	0.798	0.170	0.038	2.65×10-6	-0.016	0.064	0.040	0.805	22.034
ASXL1	rs146742828	G	A	6.692	1.503	3.38×10-5	8.48×10-6	2.383	1.647	1.67×10-5	0.148	19.824
ASXL1	rs1997823	T	C	0.623	0.139	0.089	7.47×10-6	-0.030	0.046	0.095	0.520	20.088
ASXL1	rs35580035	T	C	0.737	0.166	0.042	8.74×10-6	0.032	0.059	0.045	0.593	19.711
ASXL1	rs4955158	T	A	1.624	0.357	0.028	5.53×10-6	-0.375	0.240	0.028	0.118	20.693
ASXL1	rs56139829	A	G	0.487	0.110	0.166	8.85×10-6	0.032	0.033	0.164	0.341	19.600
ASXL1	rs58317340	T	C	0.750	0.169	0.044	9.17×10-6	-0.052	0.063	0.043	0.414	19.694
ASXL1	rs72790980	G	T	1.343	0.230	0.011	4.96×10-9	0.137	0.107	0.012	0.200	34.095
ASXL1	rs73484681	G	C	1.298	0.250	0.011	2.02×10-7	-0.106	0.131	0.011	0.416	26.956
ASXL1	rs75017035	T	C	0.578	0.124	0.104	3.06×10-6	-0.041	0.042	0.101	0.329	21.727
ASXL1	rs77234116	G	T	1.163	0.219	0.015	1.03×10-7	-0.084	0.104	0.016	0.417	28.201

Abbreviations: AP, angina pectoris; UAP, unstable angina pectoris; SAP, stable angina pectoris; EAF, effect allele frequency.

Supplementary Table S19: Pleiotropy analysis for causal association of CHIP on each AP type

CHIP gene of exposure	VAF threshold of exposure	AP type	Egger intercept	SE	P value
Overall	0.02	AP	0.025	0.019	0.212
Overall	0.1	AP	-0.025	0.031	0.434
DNMT3A	0.02	AP	0.029	0.013	0.039
DNMT3A	0.1	AP	-0.010	0.028	0.733
TET2	0.02	AP	-0.054	0.037	0.217
TET2	0.1	AP	0.004	0.028	0.881
ASXL1	0.02	AP	0.001	0.031	0.980
ASXL1	0.1	AP	0.006	0.029	0.851
Overall	0.02	UAP	0.032	0.026	0.238
Overall	0.1	UAP	0.028	0.048	0.570
DNMT3A	0.02	UAP	0.063	0.020	0.003
DNMT3A	0.1	UAP	-0.030	0.052	0.575
TET2	0.02	UAP	-0.129	0.065	0.115
TET2	0.1	UAP	-0.037	0.051	0.483
ASXL1	0.02	UAP	-0.038	0.046	0.435
ASXL1	0.1	UAP	0.038	0.044	0.409
Overall	0.02	SAP	-0.024	0.069	0.731
Overall	0.1	SAP	0.061	0.081	0.474
DNMT3A	0.02	SAP	-0.012	0.047	0.804
DNMT3A	0.1	SAP	-0.052	0.083	0.544

CHIP gene of exposure	VAF threshold of exposure	AP type	Egger intercept	SE	P value
TET2	0.02	SAP	-0.022	0.120	0.862
TET2	0.1	SAP	-0.126	0.092	0.198
ASXL1	0.02	SAP	0.050	0.115	0.671
ASXL1	0.1	SAP	-0.162	0.098	0.118

Abbreviations: AP, angina pectoris; UAP, unstable angina pectoris; SAP, stable angina pectoris; VAF, variant allele fraction.

Supplementary Table S20: Heterogeneity analysis for causal association of CHIP on each AP type

CHIP gene of exposure	VAF threshold of exposure	AP type	MR method	Q	Q df	Q p value
Overall	0.02	AP	MR Egger	17.079	12	0.147
Overall	0.02	AP	Inverse variance weighted	19.549	13	0.107
Overall	0.1	AP	MR Egger	22.127	10	0.014
Overall	0.1	AP	Inverse variance weighted	23.595	11	0.015
DNMT3A	0.02	AP	MR Egger	41.118	29	0.067
DNMT3A	0.02	AP	Inverse variance weighted	47.739	30	0.021
DNMT3A	0.1	AP	MR Egger	17.559	11	0.092
DNMT3A	0.1	AP	Inverse variance weighted	17.755	12	0.123
TET2	0.02	AP	MR Egger	6.530	4	0.163
TET2	0.02	AP	Inverse variance weighted	10.025	5	0.075
TET2	0.1	AP	MR Egger	14.888	11	0.188
TET2	0.1	AP	Inverse variance weighted	14.919	12	0.246
ASXL1	0.02	AP	MR Egger	9.369	10	0.498
ASXL1	0.02	AP	Inverse variance weighted	9.369	11	0.588
ASXL1	0.1	AP	MR Egger	12.812	15	0.617
ASXL1	0.1	AP	Inverse variance weighted	12.848	16	0.684
Overall	0.02	UAP	MR Egger	14.043	12	0.298
Overall	0.02	UAP	Inverse variance weighted	15.851	13	0.257
Overall	0.1	UAP	MR Egger	24.107	10	0.007
Overall	0.1	UAP	Inverse variance weighted	24.938	11	0.009
DNMT3A	0.02	UAP	MR Egger	40.443	29	0.077
DNMT3A	0.02	UAP	Inverse variance weighted	54.908	30	0.004

CHIP gene of exposure	VAF threshold of exposure	AP type	MR method	Q	Q df	Q p value
DNMT3A	0.1	UAP	MR Egger	29.343	11	0.002
DNMT3A	0.1	UAP	Inverse variance weighted	30.234	12	0.003
TET2	0.02	UAP	MR Egger	9.124	4	0.058
TET2	0.02	UAP	Inverse variance weighted	18.294	5	0.003
TET2	0.1	UAP	MR Egger	23.505	11	0.015
TET2	0.1	UAP	Inverse variance weighted	24.629	12	0.017
ASXL1	0.02	UAP	MR Egger	6.758	10	0.748
ASXL1	0.02	UAP	Inverse variance weighted	7.418	11	0.764
ASXL1	0.1	UAP	MR Egger	15.356	15	0.426
ASXL1	0.1	UAP	Inverse variance weighted	16.094	16	0.446
Overall	0.02	SAP	MR Egger	16.634	12	0.164
Overall	0.02	SAP	Inverse variance weighted	16.806	13	0.208
Overall	0.1	SAP	MR Egger	6.841	10	0.740
Overall	0.1	SAP	Inverse variance weighted	7.393	11	0.766
DNMT3A	0.02	SAP	MR Egger	37.375	29	0.137
DNMT3A	0.02	SAP	Inverse variance weighted	37.456	30	0.164
DNMT3A	0.1	SAP	MR Egger	6.510	11	0.837
DNMT3A	0.1	SAP	Inverse variance weighted	6.902	12	0.864
TET2	0.02	SAP	MR Egger	5.294	4	0.258
TET2	0.02	SAP	Inverse variance weighted	5.340	5	0.376
TET2	0.1	SAP	MR Egger	16.184	11	0.134
TET2	0.1	SAP	Inverse variance weighted	18.950	12	0.090
ASXL1	0.02	SAP	MR Egger	6.470	10	0.774
ASXL1	0.02	SAP	Inverse variance weighted	6.662	11	0.826

CHIP gene of exposure	VAF threshold of exposure	AP type	MR method	Q	Q df	Q p value
ASXL1	0.1	SAP	MR Egger	16.314	15	0.362
ASXL1	0.1	SAP	Inverse variance weighted	19.309	16	0.253

Abbreviations: AP, angina pectoris; UAP, unstable angina pectoris; SAP, stable angina pectoris; MR, Mendelian randomization; VAF, variant allele fraction.

Supplementary Table S21: Instrumental variables associated with other phenotypes

rsID	Associated phenotypes
rs10069690	breast carcinoma, glioma, systolic blood pressure, ovarian serous carcinoma, sex hormon×10-binding globulin measurement, estrogen-receptor negative breast cancer, age at onset, glioblastoma multiforme, ovarian carcinoma, uterine fibroid, central nervous system cancer, glioma, diastolic blood pressure, central nervous system cancer, tripl×10-negative breast cancer, mean arterial pressure, breast cancer, central nervous system cancer, glioblastoma multiforme, total blood protein measurement, serum albumin measurement, prostate carcinoma, high grade ovarian serous adenocarcinoma, malignant epithelial tumor of ovary, pulse pressure measurement, pulmonary fibrosis, thyroid carcinoma, uterine leiomyoma, serum non-albumin protein measurement, breast cancer, ovarian carcinoma, blood protein measurement, breast carcinoma, uterine leiomyoma, chronic lymphocytic leukemia, systemic lupus erythaematosus
rs10890839	Cancer
rs1136410	Telomere length
rs11708118	Liver enzyme levels, Gamma glutamyl transferase levels
rs1799986	Height
rs2048485	Schizophrenia
rs2270860	Diastolic blood pressure, Systolic blood pressure, Pulse pressure, Mean arterial pressure, Hypertension
rs2522490	Blond vs. brown/black hair color
rs2615000	Systolic blood pressure
rs2647264	Immune response to anthrax vaccine
rs2736100	lung adenocarcinoma, central nervous system cancer, glioma, platelet count, aging, epigenetic status, lung carcinoma, telomere length, mean corpuscular haemoglobin, mean corpuscular volume, clonal haematopoiesis, erythrocyte count, Testicular Germ Cell Tumor, head and neck carcinoma, lung cancer, interstitial lung disease, platelet measurement, testicular carcinoma, aging, abdominal aortic aneurysm, monocyte percentage of leukocytes, idiopathic pulmonary fibrosis
rs2736108	Breast cancer or lung cancer
rs2853676	Glioma, Uterine fibroids, Systolic blood pressure, Diastolic blood pressure, Gall stone disease or coronary artery disease

rsID	Associated phenotypes
rs2853677	lung carcinoma, lung adenocarcinoma, mean corpuscular volume, clonal haematopoiesis, mosaic loss of chromosome Y measurement, platelet crit, platelet count, telomere length, erythrocyte count, benign prostatic hyperplasia, lower urinary tract symptom, eosinophil percentage of leukocytes, neutrophil percentage of leukocytes, lymphocyte percentage of leukocytes, mean platelet volume, mean reticulocyte volume, neutrophil count, ovarian serous adenocarcinoma, non-small cell lung carcinoma, keratinocyte carcinoma, renal carcinoma, cancer, skin neoplasm, myeloproliferative disorder, aging, leukocyte count, mean corpuscular haemoglobin, prostate specific antigen measurement, eosinophil count
rs2874282	DKK1/PDGFA protein level ratio
rs37004	Prostat×10-specific antigen levels, Eosinophil counts
rs4955158	Night sleep phenotypes
rs7726159	Haematocrit, Breast cancer, Differentiated thyroid cancer, Telomere length, White blood cell count, Red blood cell count
rs840016	Rheumatoid arthritis
rs9374080	EBAG9/FKBP5 protein level ratio, Mean corpuscular volume

* Associated phenotypes were from GWAS catalog project. Instrumental variables associated with no other phenotypes were not shown.

Supplementary Table S22: Steiger directional test for causal association of CHIP on each AP type

CHIP gene in exposure	VAF threshold in exposure	Outcome	SNP r2 in exposure	SNP r2 in outcome	Correct causal direction	Steiger P value
Overall	0.02	AP	1.19×10-3	5.29×10-5	TRUE	3.47×10-38
Overall	0.02	UAP	1.19×10-3	5.79×10-5	TRUE	2.71×10-37
Overall	0.02	SAP	1.19×10-3	4.09×10-5	TRUE	1.38×10-40
Overall	0.1	AP	8.12×10-4	5.51×10-5	TRUE	1.5×10-23
Overall	0.1	UAP	8.12×10-4	5.56×10-5	TRUE	1.79×10-23
Overall	0.1	SAP	8.12×10-4	1.70×10-5	TRUE	6.03×10-31
DNMT3A	0.02	AP	2.65×10-3	1.10×10-4	TRUE	2.61×10-84
DNMT3A	0.02	UAP	2.65×10-3	1.28×10-4	TRUE	6.1×10-81
DNMT3A	0.02	SAP	2.65×10-3	8.71×10-5	TRUE	6.12×10-89
DNMT3A	0.1	AP	8.35×10-4	4.03×10-5	TRUE	1.02×10-26
DNMT3A	0.1	UAP	8.35×10-4	6.84×10-5	TRUE	1.27×10-22
DNMT3A	0.1	SAP	8.35×10-4	1.91×10-5	TRUE	2.63×10-31
TET2	0.02	AP	3.47×10-4	2.33×10-5	TRUE	5.87×10-11
TET2	0.02	UAP	3.47×10-4	4.51×10-5	TRUE	1.59×10-8
TET2	0.02	SAP	3.47×10-4	1.75×10-5	TRUE	7.12×10-12
TET2	0.1	AP	6.78×10-4	3.92×10-5	TRUE	6.12×10-21
TET2	0.1	UAP	6.78×10-4	7.35×10-5	TRUE	1.11×10-16
TET2	0.1	SAP	6.78×10-4	4.53×10-5	TRUE	4.86×10-20
ASXL1	0.02	AP	5.79×10-4	2.09×10-5	TRUE	2.16×10-20
ASXL1	0.02	UAP	5.79×10-4	1.69×10-5	TRUE	2.75×10-21
ASXL1	0.02	SAP	5.79×10-4	1.51×10-5	TRUE	9.94×10-22
ASXL1	0.1	AP	8.37×10-4	2.86×10-5	TRUE	4.38×10-29

CHIP gene in exposure	VAF threshold in exposure	Outcome	SNP r2 in exposure	SNP r2 in outcome	Correct causal direction	Steiger P value
ASXL1	0.1	UAP	8.37×10-4	3.77×10-5	TRUE	2.87×10-27
ASXL1	0.1	SAP	8.37×10-4	4.34×10-5	TRUE	2.86×10-26

Abbreviations: AP, angina pectoris; UAP, unstable angina pectoris; SAP, stable angina pectoris; VAF, variant allele fraction.

Supplementary Table S23: Reverse Mendelian randomization analysis for causal association of AP on CHIP

CHIP gene in outcome	VAF threshold in outcome	MR method	Beta	SE	P value
Overall	0.02	MR Egger	0.007	0.069	0.923
Overall	0.02	Weighted median	0.000	0.036	0.997
Overall	0.02	Inverse variance weighted	0.011	0.026	0.674
Overall	0.02	Simple mode	-0.019	0.063	0.770
Overall	0.02	Weighted mode	-0.023	0.057	0.692
Overall	0.1	MR Egger	-0.057	0.116	0.632
Overall	0.1	Weighted median	-0.030	0.065	0.642
Overall	0.1	Inverse variance weighted	-0.014	0.045	0.764
Overall	0.1	Simple mode	-0.037	0.104	0.726
Overall	0.1	Weighted mode	-0.035	0.087	0.693
DNMT3A	0.02	MR Egger	-0.066	0.119	0.589
DNMT3A	0.02	Weighted median	-0.022	0.059	0.704
DNMT3A	0.02	Inverse variance weighted	-0.041	0.045	0.362
DNMT3A	0.02	Simple mode	-0.025	0.103	0.813
DNMT3A	0.02	Weighted mode	-0.053	0.092	0.573
DNMT3A	0.1	MR Egger	-0.101	0.222	0.654
DNMT3A	0.1	Weighted median	-0.131	0.114	0.250
DNMT3A	0.1	Inverse variance weighted	-0.076	0.084	0.365
DNMT3A	0.1	Simple mode	-0.168	0.200	0.412
DNMT3A	0.1	Weighted mode	-0.138	0.160	0.399
TET2	0.02	MR Egger	-0.049	0.160	0.764
TET2	0.02	Weighted median	-0.036	0.086	0.671

CHIP gene in outcome	VAF threshold in outcome	MR method	Beta	SE	P value
TET2	0.02	Inverse variance weighted	-0.015	0.063	0.814
TET2	0.02	Simple mode	-0.115	0.167	0.500
TET2	0.02	Weighted mode	0.183	0.162	0.271
TET2	0.1	MR Egger	-0.304	0.359	0.409
TET2	0.1	Weighted median	-0.043	0.196	0.828
TET2	0.1	Inverse variance weighted	-0.108	0.139	0.434
TET2	0.1	Simple mode	-0.171	0.410	0.682
TET2	0.1	Weighted mode	0.260	0.378	0.501
ASXL1	0.02	MR Egger	-0.036	0.344	0.919
ASXL1	0.02	Weighted median	0.142	0.191	0.458
ASXL1	0.02	Inverse variance weighted	0.066	0.136	0.629
ASXL1	0.02	Simple mode	0.312	0.327	0.352
ASXL1	0.02	Weighted mode	0.279	0.309	0.379
ASXL1	0.1	MR Egger	0.017	0.600	0.977
ASXL1	0.1	Weighted median	-0.130	0.342	0.704
ASXL1	0.1	Inverse variance weighted	0.025	0.240	0.918
ASXL1	0.1	Simple mode	-0.157	0.581	0.790
ASXL1	0.1	Weighted mode	-0.108	0.523	0.839

Abbreviations: VAF, variant allele fraction; MR, Mendelian randomization.

Supplementary Table S24: Reverse Mendelian randomization analysis for causal association of UAP on CHIP

CHIP gene in outcome	VAF threshold in outcome	MR method	b	se	pval
Overall	0.02	MR Egger	-0.047	0.049	0.344
Overall	0.02	Weighted median	0.014	0.024	0.567
Overall	0.02	Inverse variance weighted	-0.001	0.017	0.955
Overall	0.02	Simple mode	0.021	0.048	0.659
Overall	0.02	Weighted mode	0.025	0.046	0.601
Overall	0.1	MR Egger	-0.119	0.091	0.207
Overall	0.1	Weighted median	-0.006	0.043	0.895
Overall	0.1	Inverse variance weighted	0.037	0.033	0.271
Overall	0.1	Simple mode	-0.035	0.075	0.648
Overall	0.1	Weighted mode	-0.035	0.069	0.620
DNMT3A	0.02	MR Egger	-0.133	0.078	0.105
DNMT3A	0.02	Weighted median	-0.036	0.037	0.330
DNMT3A	0.02	Inverse variance weighted	-0.012	0.028	0.662
DNMT3A	0.02	Simple mode	-0.083	0.077	0.295
DNMT3A	0.02	Weighted mode	-0.078	0.073	0.298
DNMT3A	0.1	MR Egger	-0.235	0.149	0.129
DNMT3A	0.1	Weighted median	0.022	0.077	0.778
DNMT3A	0.1	Inverse variance weighted	0.075	0.052	0.147
DNMT3A	0.1	Simple mode	-0.092	0.151	0.546
DNMT3A	0.1	Weighted mode	-0.077	0.142	0.591
TET2	0.02	MR Egger	-0.095	0.122	0.444
TET2	0.02	Weighted median	-0.055	0.056	0.328

CHIP gene in outcome	VAF threshold in outcome	MR method	b	se	pval
TET2	0.02	Inverse variance weighted	-0.030	0.042	0.468
TET2	0.02	Simple mode	-0.090	0.124	0.475
TET2	0.02	Weighted mode	-0.110	0.108	0.323
TET2	0.1	MR Egger	-0.156	0.274	0.574
TET2	0.1	Weighted median	0.052	0.131	0.691
TET2	0.1	Inverse variance weighted	0.033	0.091	0.714
TET2	0.1	Simple mode	0.120	0.261	0.652
TET2	0.1	Weighted mode	0.130	0.247	0.603
ASXL1	0.02	MR Egger	0.053	0.317	0.870
ASXL1	0.02	Weighted median	0.117	0.130	0.367
ASXL1	0.02	Inverse variance weighted	0.096	0.106	0.367
ASXL1	0.02	Simple mode	0.262	0.227	0.262
ASXL1	0.02	Weighted mode	0.228	0.219	0.310
ASXL1	0.1	MR Egger	-0.129	0.584	0.828
ASXL1	0.1	Weighted median	0.042	0.233	0.855
ASXL1	0.1	Inverse variance weighted	0.107	0.198	0.590
ASXL1	0.1	Simple mode	-0.067	0.433	0.878
ASXL1	0.1	Weighted mode	-0.136	0.386	0.729

Abbreviations: VAF, variant allele fraction; MR, Mendelian randomization.

Supplementary Table S25: Reverse Mendelian randomization analysis for causal association of SAP on CHIP

CHIP gene in outcome	VAF threshold in outcome	MR method	b	se	pval
Overall	0.02	MR Egger	-0.047	0.043	0.297
Overall	0.02	Weighted median	-0.011	0.018	0.529
Overall	0.02	Inverse variance weighted	-0.015	0.016	0.333
Overall	0.02	Simple mode	-0.010	0.027	0.721
Overall	0.02	Weighted mode	-0.010	0.023	0.681
Overall	0.1	MR Egger	0.024	0.058	0.690
Overall	0.1	Weighted median	-0.003	0.029	0.909
Overall	0.1	Inverse variance weighted	-0.005	0.021	0.818
Overall	0.1	Simple mode	-0.001	0.048	0.979
Overall	0.1	Weighted mode	0.002	0.047	0.960
DNMT3A	0.02	MR Egger	-0.050	0.053	0.361
DNMT3A	0.02	Weighted median	-0.013	0.027	0.621
DNMT3A	0.02	Inverse variance weighted	-0.019	0.019	0.327
DNMT3A	0.02	Simple mode	-0.029	0.049	0.572
DNMT3A	0.02	Weighted mode	-0.017	0.045	0.715
DNMT3A	0.1	MR Egger	0.051	0.108	0.646
DNMT3A	0.1	Weighted median	0.072	0.051	0.158
DNMT3A	0.1	Inverse variance weighted	0.051	0.039	0.188
DNMT3A	0.1	Simple mode	0.006	0.095	0.953
DNMT3A	0.1	Weighted mode	0.083	0.086	0.350
TET2	0.02	MR Egger	-0.090	0.100	0.389
TET2	0.02	Weighted median	-0.065	0.046	0.161

CHIP gene in outcome	VAF threshold in outcome	MR method	b	se	pval
TET2	0.02	Inverse variance weighted	-0.015	0.037	0.676
TET2	0.02	Simple mode	-0.069	0.084	0.428
TET2	0.02	Weighted mode	-0.072	0.081	0.394
TET2	0.1	MR Egger	0.037	0.217	0.867
TET2	0.1	Weighted median	-0.093	0.100	0.353
TET2	0.1	Inverse variance weighted	-0.009	0.078	0.907
TET2	0.1	Simple mode	-0.169	0.156	0.299
TET2	0.1	Weighted mode	-0.147	0.148	0.340
ASXL1	0.02	MR Egger	0.223	0.160	0.191
ASXL1	0.02	Weighted median	0.073	0.088	0.403
ASXL1	0.02	Inverse variance weighted	0.031	0.064	0.624
ASXL1	0.02	Simple mode	-0.093	0.144	0.532
ASXL1	0.02	Weighted mode	0.122	0.148	0.424
ASXL1	0.1	MR Egger	0.254	0.303	0.420
ASXL1	0.1	Weighted median	-0.036	0.157	0.820
ASXL1	0.1	Inverse variance weighted	0.011	0.115	0.925
ASXL1	0.1	Simple mode	-0.160	0.284	0.584
ASXL1	0.1	Weighted mode	-0.057	0.274	0.839

Abbreviations: VAF, variant allele fraction; MR, Mendelian randomization.

Supplementary Table S26: Instrumental variables used in MR analysis for causal association of each AP type on CHIP

AP type of exposure	rsID	Effect allele	Other allele	Beta in exposure	SE in exposure	EAF in exposure	P value in exposure	Beta in outcome	SE in outcome	EAF in outcome	P value in outcome
AP	rs11016483	A	G	0.129	0.028	0.088	5.54×10-6	0.111	0.090	0.085	0.217
AP	rs1007042	T	G	-0.078	0.017	0.450	3.92×10-6	0.024	0.053	0.464	0.654
AP	rs11611287	C	G	0.092	0.021	0.195	9.02×10-6	0.030	0.066	0.195	0.644
AP	rs117609511	T	C	0.259	0.055	0.019	2.42×10-6	0.038	0.190	0.019	0.843
AP	rs117733303	G	A	0.415	0.052	0.019	1.83×10-15	0.059	0.189	0.019	0.756
AP	rs12212886	T	C	0.183	0.040	0.040	4.68×10-6	-0.059	0.140	0.038	0.676
AP	rs1333048	C	A	0.141	0.017	0.498	8.06×10-17	-0.015	0.053	0.497	0.782
AP	rs2083637	G	A	-0.100	0.019	0.264	2.97×10-7	-0.029	0.060	0.263	0.627
AP	rs34980328	G	A	0.135	0.030	0.077	6.68×10-6	-0.050	0.102	0.076	0.621
AP	rs35868609	C	T	-0.330	0.075	0.018	9.41×10-6	0.112	0.189	0.018	0.555
AP	rs3731239	G	A	-0.081	0.018	0.367	4.33×10-6	-0.028	0.055	0.352	0.614
AP	rs429358	C	T	0.120	0.025	0.157	1.10×10-6	0.044	0.081	0.154	0.586
AP	rs660240	T	C	-0.134	0.021	0.216	3.42×10-10	0.074	0.063	0.219	0.239
AP	rs685336	G	A	0.075	0.017	0.483	8.06×10-6	0.025	0.053	0.491	0.634
AP	rs74617384	T	A	0.234	0.029	0.080	2.46×10-16	0.126	0.095	0.073	0.185
AP	rs75363344	A	G	0.197	0.044	0.037	7.19×10-6	-0.209	0.169	0.035	0.216
AP	rs77174882	A	G	0.116	0.026	0.108	8.00×10-6	0.046	0.083	0.110	0.577

AP type of exposure	rsID	Effect allele	Other allele	Beta in exposure	SE in exposure	EAF in exposure	P value in exposure	Beta in outcome	SE in outcome	EAF in outcome	P value in outcome
AP	rs997602	G	A	0.113	0.025	0.113	8.60×10-6	0.083	0.081	0.109	0.306
AP	rs9982601	T	C	0.121	0.024	0.130	4.69×10-7	-0.042	0.080	0.130	0.604
UAP	rs10455872	G	A	0.298	0.041	0.081	2.36×10-13	0.102	0.095	0.075	0.282
UAP	rs10757278	G	A	0.158	0.025	0.482	2.38×10-10	-0.039	0.053	0.478	0.460
UAP	rs11629588	T	C	-0.250	0.049	0.090	3.30×10-7	-0.028	0.095	0.085	0.771
UAP	rs116335507	A	G	-0.310	0.067	0.048	3.96×10-6	-0.136	0.133	0.045	0.306
UAP	rs117733303	G	A	0.472	0.075	0.019	3.09×10-10	0.059	0.189	0.019	0.756
UAP	rs17026734	C	T	-0.442	0.100	0.024	9.77×10-6	-0.016	0.168	0.029	0.924
UAP	rs2268539	C	T	0.234	0.049	0.056	1.81×10-6	-0.388	0.133	0.063	0.004
UAP	rs35727218	C	A	0.161	0.036	0.124	6.50×10-6	-0.030	0.079	0.134	0.707
UAP	rs429358	C	T	0.176	0.036	0.157	1.05×10-6	0.044	0.081	0.154	0.586
UAP	rs4294600	A	G	0.173	0.037	0.112	3.70×10-6	0.011	0.083	0.111	0.897
UAP	rs4675502	G	A	0.119	0.025	0.422	2.22×10-6	0.029	0.053	0.426	0.585
UAP	rs4691148	G	A	0.121	0.025	0.456	1.45×10-6	-0.024	0.053	0.443	0.649
UAP	rs599839	G	A	-0.153	0.031	0.228	7.40×10-7	0.063	0.062	0.240	0.305
UAP	rs67187482	A	T	0.379	0.085	0.016	7.70×10-6	-0.200	0.227	0.021	0.378
UAP	rs6919211	G	C	0.162	0.033	0.181	1.28×10-6	0.200	0.070	0.182	0.004
UAP	rs6935921	C	T	-0.135	0.028	0.299	1.69×10-6	-0.070	0.059	0.309	0.230
UAP	rs7119196	C	T	0.409	0.086	0.015	1.90×10-6	0.136	0.199	0.018	0.494
UAP	rs72710037	T	C	-0.159	0.034	0.182	3.08×10-6	0.036	0.067	0.185	0.592

AP type of exposure	rsID	Effect allele	Other allele	Beta in exposure	SE in exposure	EAF in exposure	P value in exposure	Beta in outcome	SE in outcome	EAF in outcome	P value in outcome
UAP	rs752502	T	C	-0.230	0.052	0.075	9.67×10-6	0.097	0.095	0.079	0.307
UAP	rs79570361	G	A	0.194	0.044	0.076	8.70×10-6	0.079	0.095	0.079	0.407
UAP	rs9457998	G	A	0.165	0.032	0.163	2.45×10-7	0.104	0.069	0.157	0.135
UAP	rs997602	G	A	0.173	0.037	0.113	2.97×10-6	0.083	0.081	0.109	0.306
SAP	rs10006362	T	C	0.313	0.067	0.252	3.39×10-6	-0.028	0.061	0.257	0.648
SAP	rs1039829	G	A	0.455	0.098	0.076	3.21×10-6	0.039	0.095	0.094	0.681
SAP	rs112261276	A	G	0.904	0.195	0.011	3.73×10-6	0.086	0.252	0.010	0.734
SAP	rs11554178	C	T	0.656	0.145	0.026	6.44×10-6	0.112	0.155	0.027	0.472
SAP	rs13280637	A	T	1.510	0.331	0.019	5.06×10-6	-0.126	0.510	0.020	0.805
SAP	rs34145779	A	C	0.514	0.115	0.049	8.21×10-6	-0.035	0.125	0.046	0.780
SAP	rs4694424	G	A	0.285	0.063	0.419	6.45×10-6	0.030	0.054	0.426	0.575
SAP	rs4871969	T	A	1.599	0.344	0.017	3.41×10-6	0.482	0.421	0.017	0.252
SAP	rs62410637	C	T	0.789	0.170	0.016	3.49×10-6	0.158	0.188	0.019	0.400
SAP	rs6810446	C	A	0.379	0.084	0.119	6.26×10-6	-0.054	0.082	0.124	0.510
SAP	rs720395	T	C	0.867	0.192	0.012	6.33×10-6	-0.103	0.252	0.014	0.682
SAP	rs75392500	A	G	0.620	0.136	0.032	5.06×10-6	0.176	0.140	0.031	0.209
SAP	rs9906785	C	T	0.319	0.067	0.295	1.65×10-6	-0.073	0.061	0.296	0.229

Abbreviations: AP, angina pectoris; UAP, unstable angina pectoris; SAP, stable angina pectoris; EAF, effect allele frequency.

Supplementary Table S27: Pleiotropy analysis for causal association of each AP type on CHIP

AP type in exposure	CHIP gene in outcome	VAF threshold in exposure	Egger intercept	se	P value
AP	Overall	0.02	0.001	0.009	0.947
AP	Overall	0.1	0.006	0.015	0.692
AP	*DNMT3A*	0.02	0.003	0.015	0.828
AP	*DNMT3A*	0.1	0.003	0.028	0.905
AP	*TET2*	0.02	0.005	0.020	0.820
AP	*TET2*	0.1	0.027	0.045	0.563
AP	*ASXL1*	0.02	0.014	0.044	0.752
AP	*ASXL1*	0.1	0.001	0.077	0.990
UAP	Overall	0.02	0.009	0.009	0.323
UAP	Overall	0.1	0.031	0.017	0.084
UAP	*DNMT3A*	0.02	0.024	0.015	0.116
UAP	*DNMT3A*	0.1	0.061	0.028	0.038
UAP	*TET2*	0.02	0.013	0.023	0.577
UAP	*TET2*	0.1	0.037	0.050	0.470
UAP	*ASXL1*	0.02	0.008	0.059	0.887
UAP	*ASXL1*	0.1	0.047	0.109	0.672
SAP	Overall	0.02	0.014	0.018	0.443
SAP	Overall	0.1	-0.013	0.024	0.604
SAP	*DNMT3A*	0.02	0.014	0.022	0.534
SAP	*DNMT3A*	0.1	5.49×10-6	0.045	1.000
SAP	*TET2*	0.02	0.033	0.041	0.440
SAP	*TET2*	0.1	-0.021	0.090	0.822

AP type in exposure	CHIP gene in outcome	VAF threshold in exposure	Egger intercept	se	P value
SAP	*ASXL1*	0.02	-0.089	0.068	0.218
SAP	*ASXL1*	0.1	-0.110	0.127	0.405

Abbreviations: AP, angina pectoris; UAP, unstable angina pectoris; SAP, stable angina pectoris; VAF, variant allele fraction.

Supplementary Table S28: Heterogeneity analysis for causal association of each AP type on CHIP

AP type in exposure	CHIP gene in outcome	VAF threshold in outcome	MR method	Q	Q df	Q pval
AP	Overall	0.02	MR Egger	19.057	17	0.325
AP	Overall	0.02	Inverse variance weighted	19.062	18	0.388
AP	Overall	0.1	MR Egger	14.801	17	0.61
AP	Overall	0.1	Inverse variance weighted	14.964	18	0.664
AP	*DNMT3A*	0.02	MR Egger	23.019	17	0.149
AP	*DNMT3A*	0.02	Inverse variance weighted	23.085	18	0.187
AP	*DNMT3A*	0.1	MR Egger	21.112	17	0.221
AP	*DNMT3A*	0.1	Inverse variance weighted	21.131	18	0.273
AP	*TET2*	0.02	MR Egger	12.926	17	0.741
AP	*TET2*	0.02	Inverse variance weighted	12.979	18	0.793
AP	*TET2*	0.1	MR Egger	13.651	17	0.692
AP	*TET2*	0.1	Inverse variance weighted	14	18	0.729
AP	*ASXL1*	0.02	MR Egger	9.896	17	0.908
AP	*ASXL1*	0.02	Inverse variance weighted	9.999	18	0.932
AP	*ASXL1*	0.1	MR Egger	14.308	17	0.645
AP	*ASXL1*	0.1	Inverse variance weighted	14.308	18	0.709
UAP	Overall	0.02	MR Egger	19.606	20	0.483
UAP	Overall	0.02	Inverse variance weighted	20.632	21	0.482
UAP	Overall	0.1	MR Egger	22.847	20	0.296
UAP	Overall	0.1	Inverse variance weighted	26.631	21	0.183
UAP	*DNMT3A*	0.02	MR Egger	21.058	20	0.394

AP type in exposure	CHIP gene in outcome	VAF threshold in outcome	MR method	Q	Q df	Q pval
UAP	*DNMT3A*	0.02	Inverse variance weighted	23.892	21	0.298
UAP	*DNMT3A*	0.1	MR Egger	16.711	20	0.672
UAP	*DNMT3A*	0.1	Inverse variance weighted	21.654	21	0.42
UAP	*TET2*	0.02	MR Egger	16.907	20	0.659
UAP	*TET2*	0.02	Inverse variance weighted	17.228	21	0.697
UAP	*TET2*	0.1	MR Egger	20.004	20	0.458
UAP	*TET2*	0.1	Inverse variance weighted	20.546	21	0.487
UAP	*ASXL1*	0.02	MR Egger	28.466	20	0.099
UAP	*ASXL1*	0.02	Inverse variance weighted	28.495	21	0.127
UAP	*ASXL1*	0.1	MR Egger	32.329	20	0.04
UAP	*ASXL1*	0.1	Inverse variance weighted	32.627	21	0.051
SAP	Overall	0.02	MR Egger	18.876	11	0.063
SAP	Overall	0.02	Inverse variance weighted	19.963	12	0.068
SAP	Overall	0.1	MR Egger	6.449	11	0.842
SAP	Overall	0.1	Inverse variance weighted	6.735	12	0.875
SAP	*DNMT3A*	0.02	MR Egger	11.234	11	0.424
SAP	*DNMT3A*	0.02	Inverse variance weighted	11.655	12	0.474
SAP	*DNMT3A*	0.1	MR Egger	13.518	11	0.261
SAP	*DNMT3A*	0.1	Inverse variance weighted	13.518	12	0.333
SAP	*TET2*	0.02	MR Egger	16.814	11	0.114
SAP	*TET2*	0.02	Inverse variance weighted	17.796	12	0.122
SAP	*TET2*	0.1	MR Egger	16.875	11	0.112
SAP	*TET2*	0.1	Inverse variance weighted	16.956	12	0.151

AP type in exposure	CHIP gene in outcome	VAF threshold in outcome	MR method	Q	Q df	Q pval
SAP	*ASXL1*	0.02	MR Egger	5.16	11	0.923
SAP	*ASXL1*	0.02	Inverse variance weighted	6.868	12	0.866
SAP	*ASXL1*	0.1	MR Egger	8.458	11	0.672
SAP	*ASXL1*	0.1	Inverse variance weighted	9.208	12	0.685

Abbreviations: AP, angina pectoris; UAP, unstable angina pectoris; SAP, stable angina pectoris; MR, Mendelian randomization; VAF, variant allele fraction.

Supplementary Table S29: Association analysis between CHIP and cytokines using covariate models

CHIP gene	VAF threshold	Cytokine	Beta	SE	P value
overall	0.02	CSF-2	-0.020	0.007	3.86E-03
overall	0.1	CSF-2	-0.019	0.013	1.31E-01
DNMT3A	0.02	CSF-2	-0.026	0.011	1.77E-02
DNMT3A	0.1	CSF-2	-0.038	0.021	6.90E-02
TET2	0.02	CSF-2	-0.059	0.017	6.26E-04
TET2	0.1	CSF-2	-0.138	0.035	9.82E-05[a]
ASXL1	0.02	CSF-2	0.051	0.036	1.61E-01
ASXL1	0.1	CSF-2	0.120	0.073	1.03E-01
overall	0.02	CSF-3	-0.017	0.010	7.41E-02
overall	0.1	CSF-3	-0.006	0.017	7.10E-01
DNMT3A	0.02	CSF-3	-0.035	0.015	1.83E-02
DNMT3A	0.1	CSF-3	-0.035	0.029	2.25E-01
TET2	0.02	CSF-3	-0.011	0.023	6.51E-01
TET2	0.1	CSF-3	0.038	0.050	4.45E-01
ASXL1	0.02	CSF-3	0.004	0.049	9.42E-01
ASXL1	0.1	CSF-3	-0.223	0.096	2.05E-02
overall	0.02	CXCL-1	-0.019	0.019	3.04E-01
overall	0.1	CXCL-1	-0.004	0.034	9.15E-01
DNMT3A	0.02	CXCL-1	-0.001	0.029	9.67E-01
DNMT3A	0.1	CXCL-1	-0.018	0.056	7.52E-01
TET2	0.02	CXCL-1	-0.025	0.046	5.82E-01
TET2	0.1	CXCL-1	0.079	0.097	4.12E-01

CHIP gene	VAF threshold	Cytokine	Beta	SE	P value
ASXL1	0.02	CXCL-1	0.160	0.097	9.94E-02
ASXL1	0.1	CXCL-1	0.183	0.188	3.30E-01
overall	0.02	CXCL-8	-0.024	0.014	8.05E-02
overall	0.1	CXCL-8	-0.048	0.024	4.60E-02
DNMT3A	0.02	CXCL-8	-0.001	0.021	9.77E-01
DNMT3A	0.1	CXCL-8	-0.024	0.040	5.44E-01
TET2	0.02	CXCL-8	-0.024	0.033	4.66E-01
TET2	0.1	CXCL-8	-0.019	0.069	7.81E-01
ASXL1	0.02	CXCL-8	-0.071	0.070	3.07E-01
ASXL1	0.1	CXCL-8	0.019	0.137	8.87E-01
overall	0.02	EPO	-0.016	0.015	2.62E-01
overall	0.1	EPO	-0.016	0.026	5.47E-01
DNMT3A	0.02	EPO	-0.018	0.023	4.34E-01
DNMT3A	0.1	EPO	-0.062	0.044	1.58E-01
TET2	0.02	EPO	-0.006	0.035	8.73E-01
TET2	0.1	EPO	0.077	0.075	3.05E-01
ASXL1	0.02	EPO	-0.004	0.075	9.59E-01
ASXL1	0.1	EPO	0.066	0.146	6.50E-01
overall	0.02	IFNG	-0.008	0.023	7.34E-01
overall	0.1	IFNG	0.051	0.041	2.09E-01
DNMT3A	0.02	IFNG	-0.002	0.035	9.47E-01
DNMT3A	0.1	IFNG	0.040	0.067	5.50E-01
TET2	0.02	IFNG	-0.046	0.055	4.01E-01
TET2	0.1	IFNG	0.188	0.117	1.09E-01

CHIP gene	VAF threshold	Cytokine	Beta	SE	P value
ASXL1	0.02	IFNG	-0.158	0.116	1.73E-01
ASXL1	0.1	IFNG	0.144	0.227	5.27E-01
overall	0.02	IL-10	0.037	0.016	2.04E-02
overall	0.1	IL-10	0.051	0.029	7.59E-02
DNMT3A	0.02	IL-10	0.047	0.025	6.21E-02
DNMT3A	0.1	IL-10	0.107	0.048	2.51E-02
TET2	0.02	IL-10	0.026	0.039	5.10E-01
TET2	0.1	IL-10	-0.020	0.083	8.08E-01
ASXL1	0.02	IL-10	0.079	0.082	3.35E-01
ASXL1	0.1	IL-10	-0.037	0.161	8.18E-01
overall	0.02	IL-11	-0.027	0.013	3.27E-02
overall	0.1	IL-11	-0.020	0.023	3.76E-01
DNMT3A	0.02	IL-11	-0.017	0.019	3.87E-01
DNMT3A	0.1	IL-11	-0.012	0.037	7.58E-01
TET2	0.02	IL-11	-0.068	0.030	2.51E-02
TET2	0.1	IL-11	-0.053	0.065	4.13E-01
ASXL1	0.02	IL-11	-0.073	0.064	2.54E-01
ASXL1	0.1	IL-11	-0.253	0.126	4.43E-02
overall	0.02	IL-12	-0.033	0.015	3.04E-02
overall	0.1	IL-12	-0.028	0.027	2.99E-01
DNMT3A	0.02	IL-12	-0.043	0.024	7.02E-02
DNMT3A	0.1	IL-12	-0.040	0.045	3.77E-01
TET2	0.02	IL-12	-0.035	0.037	3.38E-01
TET2	0.1	IL-12	0.039	0.078	6.17E-01

CHIP gene	VAF threshold	Cytokine	Beta	SE	P value
ASXL1	0.02	IL-12	0.035	0.077	6.50E-01
ASXL1	0.1	IL-12	0.167	0.151	2.69E-01
overall	0.02	IL-13	0.001	0.010	9.49E-01
overall	0.1	IL-13	0.017	0.018	3.35E-01
DNMT3A	0.02	IL-13	0.003	0.015	8.56E-01
DNMT3A	0.1	IL-13	0.005	0.029	8.62E-01
TET2	0.02	IL-13	-0.025	0.024	2.86E-01
TET2	0.1	IL-13	-0.002	0.050	9.63E-01
ASXL1	0.02	IL-13	-0.023	0.050	6.44E-01
ASXL1	0.1	IL-13	-0.060	0.098	5.36E-01
overall	0.02	IL-17A	-0.003	0.009	7.41E-01
overall	0.1	IL-17A	0.006	0.015	6.84E-01
DNMT3A	0.02	IL-17A	-0.012	0.013	3.72E-01
DNMT3A	0.1	IL-17A	-0.004	0.025	8.62E-01
TET2	0.02	IL-17A	-0.007	0.021	7.42E-01
TET2	0.1	IL-17A	0.032	0.044	4.60E-01
ASXL1	0.02	IL-17A	0.016	0.043	7.16E-01
ASXL1	0.1	IL-17A	0.128	0.085	1.34E-01
overall	0.02	IL-17C	-0.012	0.014	3.68E-01
overall	0.1	IL-17C	0.026	0.025	2.89E-01
DNMT3A	0.02	IL-17C	-0.022	0.021	3.00E-01
DNMT3A	0.1	IL-17C	0.007	0.041	8.68E-01
TET2	0.02	IL-17C	-0.066	0.033	4.82E-02
TET2	0.1	IL-17C	-0.132	0.071	6.49E-02

CHIP gene	VAF threshold	Cytokine	Beta	SE	P value
ASXL1	0.02	IL-17C	0.030	0.070	6.72E-01
ASXL1	0.1	IL-17C	0.179	0.138	1.96E-01
overall	0.02	IL-17D	0.000	0.007	9.94E-01
overall	0.1	IL-17D	0.011	0.012	3.53E-01
DNMT3A	0.02	IL-17D	0.012	0.010	2.31E-01
DNMT3A	0.1	IL-17D	0.046	0.020	2.16E-02
TET2	0.02	IL-17D	-0.007	0.016	6.45E-01
TET2	0.1	IL-17D	-0.008	0.034	8.17E-01
ASXL1	0.02	IL-17D	-0.032	0.034	3.48E-01
ASXL1	0.1	IL-17D	-0.008	0.067	9.05E-01
overall	0.02	IL-17F	0.021	0.015	1.67E-01
overall	0.1	IL-17F	0.061	0.027	2.56E-02
DNMT3A	0.02	IL-17F	0.016	0.024	4.95E-01
DNMT3A	0.1	IL-17F	0.090	0.045	4.68E-02
TET2	0.02	IL-17F	0.040	0.037	2.83E-01
TET2	0.1	IL-17F	0.120	0.079	1.27E-01
ASXL1	0.02	IL-17F	-0.114	0.078	1.42E-01
ASXL1	0.1	IL-17F	-0.129	0.152	3.96E-01
overall	0.02	IL-18	0.005	0.009	5.51E-01
overall	0.1	IL-18	0.030	0.017	6.72E-02
DNMT3A	0.02	IL-18	0.002	0.014	8.98E-01
DNMT3A	0.1	IL-18	0.052	0.027	5.66E-02
TET2	0.02	IL-18	0.009	0.022	6.99E-01
TET2	0.1	IL-18	0.091	0.047	5.27E-02

CHIP gene	VAF threshold	Cytokine	Beta	SE	P value
ASXL1	0.02	IL-18	0.055	0.047	2.48E-01
ASXL1	0.1	IL-18	0.171	0.091	6.11E-02
overall	0.02	IL--1α	-0.006	0.011	6.05E-01
overall	0.1	IL--1α	-0.011	0.020	5.70E-01
DNMT3A	0.02	IL--1α	-0.027	0.017	1.23E-01
DNMT3A	0.1	IL--1α	-0.071	0.033	3.38E-02
TET2	0.02	IL--1α	0.006	0.027	8.21E-01
TET2	0.1	IL--1α	-0.043	0.058	4.63E-01
ASXL1	0.02	IL--1α	-0.038	0.057	5.07E-01
ASXL1	0.1	IL--1α	0.082	0.113	4.66E-01
overall	0.02	IL--1β	0.027	0.011	1.83E-02
overall	0.1	IL--1β	0.057	0.020	4.86E-03
DNMT3A	0.02	IL--1β	0.015	0.018	4.01E-01
DNMT3A	0.1	IL--1β	0.026	0.034	4.44E-01
TET2	0.02	IL--1β	0.052	0.027	6.02E-02
TET2	0.1	IL--1β	0.166	0.058	4.46E-03
ASXL1	0.02	IL--1β	0.077	0.058	1.83E-01
ASXL1	0.1	IL--1β	0.156	0.113	1.69E-01
overall	0.02	IL-2	-0.005	0.006	4.31E-01
overall	0.1	IL-2	0.005	0.011	6.33E-01
DNMT3A	0.02	IL-2	-0.001	0.010	9.35E-01
DNMT3A	0.1	IL-2	-0.005	0.018	7.67E-01
TET2	0.02	IL-2	-0.016	0.015	3.01E-01
TET2	0.1	IL-2	0.009	0.032	7.69E-01

CHIP gene	VAF threshold	Cytokine	Beta	SE	P value
ASXL1	0.02	IL-2	-0.010	0.032	7.48E-01
ASXL1	0.1	IL-2	-0.057	0.062	3.56E-01
overall	0.02	IL-22	0.020	0.013	1.38E-01
overall	0.1	IL-22	0.060	0.024	1.11E-02
DNMT3A	0.02	IL-22	0.017	0.021	4.14E-01
DNMT3A	0.1	IL-22	0.048	0.039	2.23E-01
TET2	0.02	IL-22	0.017	0.032	6.06E-01
TET2	0.1	IL-22	0.042	0.067	5.28E-01
ASXL1	0.02	IL-22	0.067	0.069	3.32E-01
ASXL1	0.1	IL-22	0.022	0.137	8.73E-01
overall	0.02	IL-3	0.022	0.012	5.98E-02
overall	0.1	IL-3	0.017	0.021	4.12E-01
DNMT3A	0.02	IL-3	0.029	0.018	1.09E-01
DNMT3A	0.1	IL-3	0.022	0.035	5.37E-01
TET2	0.02	IL-3	0.004	0.029	8.78E-01
TET2	0.1	IL-3	0.051	0.059	3.87E-01
ASXL1	0.02	IL-3	-0.035	0.063	5.76E-01
ASXL1	0.1	IL-3	0.130	0.123	2.92E-01
overall	0.02	IL-4	-0.008	0.018	6.65E-01
overall	0.1	IL-4	-0.011	0.032	7.40E-01
DNMT3A	0.02	IL-4	0.016	0.028	5.64E-01
DNMT3A	0.1	IL-4	0.025	0.053	6.33E-01
TET2	0.02	IL-4	0.017	0.043	7.03E-01
TET2	0.1	IL-4	-0.036	0.093	6.99E-01

CHIP gene	VAF threshold	Cytokine	Beta	SE	P value
ASXL1	0.02	IL-4	-0.073	0.092	4.26E-01
ASXL1	0.1	IL-4	-0.201	0.180	2.65E-01
overall	0.02	IL-5	-0.013	0.028	6.36E-01
overall	0.1	IL-5	-0.107	0.050	3.35E-02
DNMT3A	0.02	IL-5	-0.007	0.044	8.66E-01
DNMT3A	0.1	IL-5	-0.026	0.083	7.57E-01
TET2	0.02	IL-5	-0.007	0.068	9.20E-01
TET2	0.1	IL-5	-0.053	0.145	7.17E-01
ASXL1	0.02	IL-5	-0.029	0.143	8.40E-01
ASXL1	0.1	IL-5	-0.232	0.282	4.09E-01
overall	0.02	IL-6	-0.023	0.014	1.14E-01
overall	0.1	IL-6	0.005	0.026	8.48E-01
DNMT3A	0.02	IL-6	-0.044	0.022	4.84E-02
DNMT3A	0.1	IL-6	-0.033	0.043	4.39E-01
TET2	0.02	IL-6	-0.008	0.035	8.25E-01
TET2	0.1	IL-6	0.053	0.074	4.74E-01
ASXL1	0.02	IL-6	-0.018	0.073	8.04E-01
ASXL1	0.1	IL-6	0.074	0.144	6.05E-01
overall	0.02	IL-9	0.002	0.011	8.50E-01
overall	0.1	IL-9	0.004	0.019	8.50E-01
DNMT3A	0.02	IL-9	0.008	0.017	6.35E-01
DNMT3A	0.1	IL-9	-0.006	0.032	8.42E-01
TET2	0.02	IL-9	-0.041	0.026	1.19E-01
TET2	0.1	IL-9	-0.051	0.055	3.50E-01

CHIP gene	VAF threshold	Cytokine	Beta	SE	P value
ASXL1	0.02	IL-9	0.012	0.057	8.27E-01
ASXL1	0.1	IL-9	0.053	0.112	6.35E-01
overall	0.02	TNF	-0.003	0.007	7.21E-01
overall	0.1	TNF	0.012	0.013	3.75E-01
DNMT3A	0.02	TNF	-0.003	0.011	7.87E-01
DNMT3A	0.1	TNF	0.007	0.022	7.62E-01
TET2	0.02	TNF	-0.014	0.018	4.35E-01
TET2	0.1	TNF	-0.023	0.038	5.40E-01
ASXL1	0.02	TNF	-0.023	0.039	5.50E-01
ASXL1	0.1	TNF	-0.008	0.074	9.09E-01

Abbreviations: VAF, variant allele fraction

[a] Indicates statistical significance in multiple comparisons.

Supplementary Table S30: Association analysis between CHIP and cytokines using PSM models

CHIP gene	VAF threshold	Cytokine	Beta	SE	P value
overall	0.02	CSF-2	-0.020	0.010	3.71E-02
overall	0.1	CSF-2	-0.031	0.017	5.99E-02
DNMT3A	0.02	CSF-2	-0.025	0.015	9.41E-02
DNMT3A	0.1	CSF-2	-0.064	0.028	2.55E-02
TET2	0.02	CSF-2	-0.055	0.024	2.11E-02
TET2	0.1	CSF-2	-0.140	0.048	4.20E-03
ASXL1	0.02	CSF-2	0.033	0.049	5.08E-01
ASXL1	0.1	CSF-2	0.054	0.102	6.02E-01
overall	0.02	CSF-3	-0.006	0.014	6.79E-01
overall	0.1	CSF-3	0.009	0.026	7.40E-01
DNMT3A	0.02	CSF-3	-0.035	0.022	1.07E-01
DNMT3A	0.1	CSF-3	-0.035	0.043	4.09E-01
TET2	0.02	CSF-3	0.030	0.034	3.84E-01
TET2	0.1	CSF-3	0.076	0.071	2.88E-01
ASXL1	0.02	CSF-3	0.051	0.080	5.27E-01
ASXL1	0.1	CSF-3	-0.183	0.143	2.04E-01
overall	0.02	CXCL-1	0.119	0.026	3.98E-06[a]
overall	0.1	CXCL-1	0.118	0.047	1.25E-02
DNMT3A	0.02	CXCL-1	0.064	0.041	1.13E-01
DNMT3A	0.1	CXCL-1	-0.026	0.076	7.33E-01
TET2	0.02	CXCL-1	0.083	0.068	2.24E-01
TET2	0.1	CXCL-1	0.247	0.130	5.86E-02

CHIP gene	VAF threshold	Cytokine	Beta	SE	P value
ASXL1	0.02	CXCL-1	0.198	0.139	1.56E-01
ASXL1	0.1	CXCL-1	0.327	0.242	1.82E-01
overall	0.02	CXCL-8	0.022	0.019	2.47E-01
overall	0.1	CXCL-8	-0.024	0.034	4.83E-01
DNMT3A	0.02	CXCL-8	0.027	0.030	3.65E-01
DNMT3A	0.1	CXCL-8	-0.008	0.057	8.89E-01
TET2	0.02	CXCL-8	0.000	0.047	9.96E-01
TET2	0.1	CXCL-8	0.023	0.085	7.90E-01
ASXL1	0.02	CXCL-8	-0.041	0.091	6.52E-01
ASXL1	0.1	CXCL-8	-0.161	0.183	3.85E-01
overall	0.02	EPO	-0.026	0.020	1.97E-01
overall	0.1	EPO	-0.017	0.037	6.38E-01
DNMT3A	0.02	EPO	-0.027	0.032	4.09E-01
DNMT3A	0.1	EPO	-0.019	0.057	7.36E-01
TET2	0.02	EPO	0.003	0.046	9.43E-01
TET2	0.1	EPO	0.117	0.093	2.09E-01
ASXL1	0.02	EPO	0.001	0.106	9.90E-01
ASXL1	0.1	EPO	0.173	0.205	4.00E-01
overall	0.02	IFNG	0.026	0.031	4.07E-01
overall	0.1	IFNG	0.077	0.059	1.92E-01
DNMT3A	0.02	IFNG	0.033	0.050	5.02E-01
DNMT3A	0.1	IFNG	0.063	0.094	5.01E-01
TET2	0.02	IFNG	0.008	0.077	9.18E-01
TET2	0.1	IFNG	0.242	0.152	1.14E-01

CHIP gene	VAF threshold	Cytokine	Beta	SE	P value
ASXL1	0.02	IFNG	-0.181	0.193	3.50E-01
ASXL1	0.1	IFNG	-0.298	0.261	2.59E-01
overall	0.02	IL-10	0.068	0.022	2.27E-03
overall	0.1	IL-10	0.132	0.039	8.00E-04
DNMT3A	0.02	IL-10	0.057	0.034	9.53E-02
DNMT3A	0.1	IL-10	0.174	0.070	1.29E-02
TET2	0.02	IL-10	0.076	0.056	1.79E-01
TET2	0.1	IL-10	0.082	0.122	5.01E-01
ASXL1	0.02	IL-10	0.245	0.105	1.98E-02
ASXL1	0.1	IL-10	0.088	0.200	6.64E-01
overall	0.02	IL-11	-0.011	0.017	5.16E-01
overall	0.1	IL-11	-0.010	0.031	7.51E-01
DNMT3A	0.02	IL-11	-0.023	0.027	3.79E-01
DNMT3A	0.1	IL-11	0.048	0.052	3.57E-01
TET2	0.02	IL-11	-0.044	0.039	2.51E-01
TET2	0.1	IL-11	-0.061	0.093	5.14E-01
ASXL1	0.02	IL-11	-0.008	0.078	9.15E-01
ASXL1	0.1	IL-11	-0.166	0.107	1.27E-01
overall	0.02	IL-12	-0.007	0.022	7.63E-01
overall	0.1	IL-12	-0.012	0.040	7.63E-01
DNMT3A	0.02	IL-12	-0.033	0.034	3.35E-01
DNMT3A	0.1	IL-12	0.014	0.065	8.33E-01
TET2	0.02	IL-12	-0.024	0.056	6.73E-01
TET2	0.1	IL-12	-0.021	0.119	8.59E-01

CHIP gene	VAF threshold	Cytokine	Beta	SE	P value
ASXL1	0.02	IL-12	0.044	0.126	7.24E-01
ASXL1	0.1	IL-12	0.202	0.239	4.01E-01
overall	0.02	IL-13	0.007	0.013	5.95E-01
overall	0.1	IL-13	0.028	0.025	2.62E-01
DNMT3A	0.02	IL-13	-0.005	0.021	7.93E-01
DNMT3A	0.1	IL-13	-0.011	0.042	7.97E-01
TET2	0.02	IL-13	-0.020	0.033	5.42E-01
TET2	0.1	IL-13	0.029	0.079	7.18E-01
ASXL1	0.02	IL-13	0.018	0.052	7.29E-01
ASXL1	0.1	IL-13	0.054	0.091	5.59E-01
overall	0.02	IL-17A	-0.005	0.012	7.04E-01
overall	0.1	IL-17A	0.005	0.022	8.26E-01
DNMT3A	0.02	IL-17A	-0.026	0.019	1.77E-01
DNMT3A	0.1	IL-17A	0.001	0.036	9.84E-01
TET2	0.02	IL-17A	-0.015	0.030	6.21E-01
TET2	0.1	IL-17A	0.040	0.074	5.92E-01
ASXL1	0.02	IL-17A	-0.037	0.077	6.29E-01
ASXL1	0.1	IL-17A	0.113	0.142	4.31E-01
overall	0.02	IL-17C	0.015	0.019	4.37E-01
overall	0.1	IL-17C	0.055	0.036	1.28E-01
DNMT3A	0.02	IL-17C	-0.032	0.031	3.01E-01
DNMT3A	0.1	IL-17C	0.062	0.059	2.92E-01
TET2	0.02	IL-17C	-0.023	0.047	6.24E-01
TET2	0.1	IL-17C	-0.087	0.092	3.48E-01

CHIP gene	VAF threshold	Cytokine	Beta	SE	P value
ASXL1	0.02	IL-17C	-0.007	0.110	9.53E-01
ASXL1	0.1	IL-17C	0.000	0.210	9.98E-01
overall	0.02	IL-17D	0.006	0.009	5.22E-01
overall	0.1	IL-17D	-0.006	0.018	7.43E-01
DNMT3A	0.02	IL-17D	0.007	0.015	6.39E-01
DNMT3A	0.1	IL-17D	0.025	0.034	4.50E-01
TET2	0.02	IL-17D	-0.016	0.024	4.98E-01
TET2	0.1	IL-17D	-0.025	0.042	5.52E-01
ASXL1	0.02	IL-17D	0.020	0.047	6.72E-01
ASXL1	0.1	IL-17D	0.053	0.103	6.07E-01
overall	0.02	IL-17F	0.042	0.021	4.37E-02
overall	0.1	IL-17F	0.093	0.040	2.06E-02
DNMT3A	0.02	IL-17F	0.038	0.032	2.29E-01
DNMT3A	0.1	IL-17F	0.105	0.066	1.11E-01
TET2	0.02	IL-17F	0.072	0.056	2.02E-01
TET2	0.1	IL-17F	0.142	0.128	2.68E-01
ASXL1	0.02	IL-17F	-0.054	0.106	6.08E-01
ASXL1	0.1	IL-17F	0.075	0.163	6.49E-01
overall	0.02	IL-18	0.092	0.013	5.19E-13[a]
overall	0.1	IL-18	0.115	0.024	2.80E-06[a]
DNMT3A	0.02	IL-18	0.045	0.020	2.69E-02
DNMT3A	0.1	IL-18	0.087	0.040	2.79E-02
TET2	0.02	IL-18	0.099	0.032	2.07E-03
TET2	0.1	IL-18	0.162	0.074	2.96E-02

CHIP gene	VAF threshold	Cytokine	Beta	SE	P value
ASXL1	0.02	IL-18	0.174	0.069	1.28E-02
ASXL1	0.1	IL-18	0.268	0.134	5.06E-02
overall	0.02	IL--1α	0.035	0.014	1.39E-02
overall	0.1	IL--1α	0.066	0.024	5.71E-03
DNMT3A	0.02	IL--1α	0.004	0.022	8.73E-01
DNMT3A	0.1	IL--1α	-0.069	0.043	1.14E-01
TET2	0.02	IL--1α	0.037	0.036	2.99E-01
TET2	0.1	IL--1α	0.054	0.064	3.99E-01
ASXL1	0.02	IL--1α	0.038	0.062	5.39E-01
ASXL1	0.1	IL--1α	0.166	0.159	3.00E-01
overall	0.02	IL--1β	0.274	0.013	1.78E-89[a]
overall	0.1	IL--1β	0.305	0.026	2.42E-30[a]
DNMT3A	0.02	IL--1β	0.105	0.023	4.92E-06[a]
DNMT3A	0.1	IL--1β	0.135	0.045	2.75E-03
TET2	0.02	IL--1β	0.298	0.035	3.44E-17[a]
TET2	0.1	IL--1β	0.440	0.081	1.63E-07[a]
ASXL1	0.02	IL--1β	0.227	0.069	1.07E-03
ASXL1	0.1	IL--1β	0.299	0.193	1.28E-01
overall	0.02	IL-2	0.009	0.008	2.73E-01
overall	0.1	IL-2	0.013	0.016	4.19E-01
DNMT3A	0.02	IL-2	-0.002	0.014	8.92E-01
DNMT3A	0.1	IL-2	-0.009	0.026	7.39E-01
TET2	0.02	IL-2	0.001	0.022	9.61E-01
TET2	0.1	IL-2	0.012	0.050	8.08E-01

CHIP gene	VAF threshold	Cytokine	Beta	SE	P value
ASXL1	0.02	IL-2	-0.033	0.046	4.74E-01
ASXL1	0.1	IL-2	-0.120	0.093	2.04E-01
overall	0.02	IL-22	0.029	0.019	1.18E-01
overall	0.1	IL-22	0.040	0.035	2.57E-01
DNMT3A	0.02	IL-22	-0.006	0.030	8.42E-01
DNMT3A	0.1	IL-22	0.037	0.057	5.24E-01
TET2	0.02	IL-22	0.034	0.045	4.45E-01
TET2	0.1	IL-22	0.013	0.097	8.97E-01
ASXL1	0.02	IL-22	0.064	0.096	5.05E-01
ASXL1	0.1	IL-22	0.061	0.170	7.22E-01
overall	0.02	IL-3	0.022	0.016	1.71E-01
overall	0.1	IL-3	0.018	0.029	5.36E-01
DNMT3A	0.02	IL-3	0.056	0.026	3.09E-02
DNMT3A	0.1	IL-3	0.107	0.055	4.99E-02
TET2	0.02	IL-3	0.012	0.037	7.51E-01
TET2	0.1	IL-3	0.040	0.075	5.90E-01
ASXL1	0.02	IL-3	0.013	0.091	8.83E-01
ASXL1	0.1	IL-3	-0.021	0.153	8.92E-01
overall	0.02	IL-4	0.000	0.024	9.98E-01
overall	0.1	IL-4	-0.001	0.045	9.79E-01
DNMT3A	0.02	IL-4	0.025	0.037	4.96E-01
DNMT3A	0.1	IL-4	0.010	0.061	8.75E-01
TET2	0.02	IL-4	0.032	0.056	5.68E-01
TET2	0.1	IL-4	-0.168	0.104	1.10E-01

CHIP gene	VAF threshold	Cytokine	Beta	SE	P value
ASXL1	0.02	IL-4	-0.151	0.132	2.55E-01
ASXL1	0.1	IL-4	-0.130	0.301	6.67E-01
overall	0.02	IL-5	0.012	0.038	7.53E-01
overall	0.1	IL-5	-0.110	0.067	9.96E-02
DNMT3A	0.02	IL-5	-0.013	0.060	8.29E-01
DNMT3A	0.1	IL-5	0.008	0.113	9.41E-01
TET2	0.02	IL-5	0.050	0.095	6.03E-01
TET2	0.1	IL-5	-0.074	0.196	7.08E-01
ASXL1	0.02	IL-5	0.085	0.182	6.42E-01
ASXL1	0.1	IL-5	0.114	0.319	7.22E-01
overall	0.02	IL-6	0.006	0.021	7.83E-01
overall	0.1	IL-6	0.023	0.038	5.43E-01
DNMT3A	0.02	IL-6	-0.045	0.033	1.67E-01
DNMT3A	0.1	IL-6	0.003	0.062	9.56E-01
TET2	0.02	IL-6	0.022	0.057	7.03E-01
TET2	0.1	IL-6	0.148	0.095	1.20E-01
ASXL1	0.02	IL-6	0.007	0.106	9.51E-01
ASXL1	0.1	IL-6	0.237	0.210	2.63E-01
overall	0.02	IL-9	-0.003	0.015	8.68E-01
overall	0.1	IL-9	-0.003	0.028	9.23E-01
DNMT3A	0.02	IL-9	0.013	0.024	5.91E-01
DNMT3A	0.1	IL-9	-0.048	0.049	3.26E-01
TET2	0.02	IL-9	-0.024	0.034	4.74E-01
TET2	0.1	IL-9	0.011	0.060	8.57E-01

CHIP gene	VAF threshold	Cytokine	Beta	SE	P value
ASXL1	0.02	IL-9	0.022	0.089	8.06E-01
ASXL1	0.1	IL-9	0.071	0.118	5.48E-01
overall	0.02	TNF	0.016	0.010	1.34E-01
overall	0.1	TNF	0.015	0.019	4.43E-01
DNMT3A	0.02	TNF	-0.011	0.016	5.12E-01
DNMT3A	0.1	TNF	0.007	0.027	7.99E-01
TET2	0.02	TNF	0.022	0.023	3.36E-01
TET2	0.1	TNF	-0.034	0.044	4.48E-01
ASXL1	0.02	TNF	-0.007	0.048	8.83E-01
ASXL1	0.1	TNF	0.005	0.080	9.48E-01

Abbreviations: VAF, variant allele fraction

[a] Indicates statistical significance in multiple comparisons.

Supplementary Table S31: Metabolites significantly associated with CHIP status

CHIP gene	Threshold	Metabolite	P value	Beta	SE
ASXL1	0.1	Free.Cholesterol.in.Small.LDL...Instance.0	0.00027	-0.25248	0.069306
ASXL1	0.1	Free.Cholesterol.in.Medium.LDL...Instance.0	0.000284	-0.25119	0.069204
overall	0.02	Tyrosine...Instance.0	0.000299	-0.02718	0.007516
overall	0.1	Linoleic.Acid...Instance.0	0.000431	-0.04642	0.013186
ASXL1	0.1	Phospholipids.to.Total.Lipids.in.Small.HDL.percentage...Instance.0	0.000485	0.237406	0.068048
ASXL1	0.1	Free.Cholesterol.in.LDL...Instance.0	0.000557	-0.23534	0.068174
ASXL1	0.1	Cholesterol.to.Total.Lipids.in.Very.Large.HDL.percentage...Instance.0	0.000572	-0.23299	0.067639
overall	0.1	Tyrosine...Instance.0	0.000602	-0.04537	0.013225
ASXL1	0.1	Lactate...Instance.0	0.000622	0.242011	0.070722
overall	0.1	Omega.6.Fatty.Acids...Instance.0	0.000686	-0.04447	0.013098
ASXL1	0.1	Clinical.LDL.Cholesterol...Instance.0	0.000772	-0.23057	0.068565
ASXL1	0.1	Cholesterol.to.Total.Lipids.in.IDL.percentage...Instance.0	0.000803	-0.21858	0.065212
ASXL1	0.02	Docosahexaenoic.Acid...Instance.0	0.000821	-0.12432	0.037157
ASXL1	0.1	Phospholipids.to.Total.Lipids.in.IDL.percentage...Instance.0	0.00087	0.231368	0.069488
DNMT3A	0.02	Spectrometer.corrected.alanine...Instance.0	0.000888	-0.03906	0.011753
ASXL1	0.1	Free.Cholesterol.in.Large.LDL...Instance.0	0.000997	-0.22174	0.067365
TET2	0.1	Albumin...Instance.0	0.001016	0.128904	0.039227
ASXL1	0.1	Phospholipids.in.Large.LDL...Instance.0	0.001061	-0.22387	0.06838
ASXL1	0.1	Phospholipids.in.LDL...Instance.0	0.001071	-0.22584	0.06904
ASXL1	0.1	Phospholipids.in.Medium.LDL...Instance.0	0.001358	-0.22257	0.069478
ASXL1	0.1	Free.Cholesterol.in.IDL...Instance.0	0.001479	-0.20997	0.066054
ASXL1	0.02	Phospholipids.to.Total.Lipids.in.IDL.percentage...Instance.0	0.001509	0.123629	0.038963
DNMT3A	0.02	Tyrosine...Instance.0	0.001518	-0.03753	0.011835

ASXL1	0.1	Cholesterol.in.Small.LDL...Instance.0	0.001548	-0.21962	0.069376
ASXL1	0.1	LDL.Cholesterol...Instance.0	0.001624	-0.21716	0.068904
TET2	0.1	Tyrosine...Instance.0	0.00164	-0.1238	0.039316
ASXL1	0.1	Cholesterol.in.Medium.LDL...Instance.0	0.001693	-0.21778	0.069367
ASXL1	0.1	Cholesterol.in.Large.LDL...Instance.0	0.001991	-0.21104	0.068262
DNMT3A	0.1	Albumin...Instance.0	0.002017	0.070513	0.022837
ASXL1	0.1	Total.Lipids.in.LDL...Instance.0	0.002116	-0.21218	0.069036
ASXL1	0.1	Total.Lipids.in.Medium.LDL...Instance.0	0.002251	-0.21198	0.069388
ASXL1	0.1	Total.Lipids.in.Large.LDL...Instance.0	0.002388	-0.20782	0.068425
ASXL1	0.1	Cholesterol.to.Total.Lipids.in.Small.LDL.percentage...Instance.0	0.002551	-0.20712	0.068645
ASXL1	0.1	Phospholipids.in.Small.LDL...Instance.0	0.002705	-0.2087	0.069581
ASXL1	0.1	Cholesteryl.Esters.in.LDL...Instance.0	0.002716	-0.2072	0.069106
ASXL1	0.1	Total.Lipids.in.Small.LDL...Instance.0	0.002786	-0.20763	0.069431
TET2	0.1	Linoleic.Acid...Instance.0	0.002796	-0.11712	0.039178
ASXL1	0.1	Cholesteryl.Esters.in.Large.LDL...Instance.0	0.002797	-0.20496	0.068566
ASXL1	0.1	Cholesterol.in.IDL...Instance.0	0.002801	-0.19661	0.06578
TET2	0.1	Omega.6.Fatty.Acids...Instance.0	0.002847	-0.11614	0.038922
DNMT3A	0.02	Alanine...Instance.0	0.002888	-0.03539	0.011877
overall	0.02	Linoleic.Acid...Instance.0	0.003207	-0.0221	0.0075
ASXL1	0.1	Cholesteryl.Esters.to.Total.Lipids.in.Very.Large.HDL.percentage...Instance.0	0.003235	-0.20504	0.069635
ASXL1	0.1	Triglycerides.to.Total.Lipids.in.Medium.LDL.percentage...Instance.0	0.003316	0.198678	0.06765
overall	0.1	Polyunsaturated.Fatty.Acids...Instance.0	0.003537	-0.03801	0.01303
ASXL1	0.02	Free.Cholesterol.in.Small.LDL...Instance.0	0.003553	-0.11331	0.038866
ASXL1	0.1	Concentration.of.Medium.LDL.Particles...Instance.0	0.00359	-0.20191	0.069333
ASXL1	0.1	Total.Cholesterol.Minus.HDL.C...Instance.0	0.003599	-0.19987	0.068653
ASXL1	0.1	Phospholipids.to.Total.Lipids.in.Very.Small.VLDL.percentage...Instance.0	0.003644	0.191978	0.06603
overall	0.1	Average.Diameter.for.VLDL.Particles...Instance.0	0.003783	-0.0349	0.012053
ASXL1	0.1	Cholesteryl.Esters.in.IDL...Instance.0	0.003815	-0.19026	0.065765

ASXL1	0.1	Cholesteryl.Esters.in.Medium.LDL...Instance.0	0.004002	-0.19936	0.06927
ASXL1	0.1	Cholesteryl.Esters.in.Medium.VLDL...Instance.0	0.004093	-0.19551	0.0681
ASXL1	0.1	Cholesterol.to.Total.Lipids.in.Small.HDL.percentage...Instance.0	0.004129	-0.19757	0.068885
ASXL1	0.02	Free.Cholesterol.in.Medium.LDL...Instance.0	0.004248	-0.11096	0.038809
ASXL1	0.1	Cholesteryl.Esters.in.Small.LDL...Instance.0	0.00432	-0.19777	0.069302
ASXL1	0.1	Free.Cholesterol.to.Total.Lipids.in.IDL.percentage...Instance.0	0.004354	-0.19637	0.06887
ASXL1	0.1	Concentration.of.LDL.Particles...Instance.0	0.004468	-0.19691	0.069261
overall	0.1	Total.Lipids.in.Lipoprotein.Particles...Instance.0	0.004566	-0.03696	0.013031
overall	0.02	Spectrometer.corrected.alanine...Instance.0	0.004832	-0.02103	0.007464
overall	0.02	Alanine...Instance.0	0.004874	-0.02125	0.007547
ASXL1	0.1	Total.Esterified.Cholesterol...Instance.0	0.004904	-0.18505	0.065775
ASXL1	0.02	Cholesterol.in.Small.HDL...Instance.0	0.005036	-0.10795	0.038489
overall	0.02	Total.Esterified.Cholesterol...Instance.0	0.005079	-0.02003	0.007147
ASXL1	0.02	Omega.3.Fatty.Acids...Instance.0	0.005152	-0.10666	0.038127
overall	0.02	Omega.6.Fatty.Acids...Instance.0	0.005187	-0.02082	0.00745
overall	0.02	Total.Cholesterol...Instance.0	0.005335	-0.02004	0.007193
TET2	0.1	Concentration.of.Small.HDL.Particles...Instance.0	0.005395	-0.10879	0.039097
ASXL1	0.02	Cholesteryl.Esters.in.Small.HDL...Instance.0	0.005481	-0.10761	0.038745
ASXL1	0.1	Total.Cholesterol...Instance.0	0.005589	-0.18343	0.066196
overall	0.1	Phosphatidylcholines...Instance.0	0.005628	-0.0339	0.012243
ASXL1	0.1	Triglycerides.to.Total.Lipids.in.IDL.percentage...Instance.0	0.005842	0.177158	0.064268
overall	0.1	Total.Phospholipids.in.Lipoprotein.Particles...Instance.0	0.005859	-0.03439	0.01248
ASXL1	0.1	Concentration.of.Large.LDL.Particles...Instance.0	0.006075	-0.18979	0.069173
ASXL1	0.02	Concentration.of.Small.HDL.Particles...Instance.0	0.006075	-0.1059	0.038596
ASXL1	0.02	Phospholipids.in.Medium.LDL...Instance.0	0.006134	-0.10678	0.038963
ASXL1	0.1	Cholesterol.in.Medium.VLDL...Instance.0	0.006263	-0.18825	0.068861
ASXL1	0.1	Triglycerides.to.Total.Lipids.in.Large.LDL.percentage...Instance.0	0.006316	0.180253	0.066004
ASXL1	0.1	Apolipoprotein.B...Instance.0	0.00632	-0.18871	0.069105

overall	0.1	Albumin...Instance.0	0.006882	0.035649	0.013191
TET2	0.1	Cholesterol.in.Small.HDL...Instance.0	0.006893	-0.10535	0.038989
ASXL1	0.1	Cholesteryl.Esters.to.Total.Lipids.in.IDL.percentage...Instance.0	0.006903	-0.17721	0.065595
overall	0.02	Total.Free.Cholesterol...Instance.0	0.006922	-0.01974	0.007308
ASXL1	0.1	Total.Lipids.in.IDL...Instance.0	0.006929	-0.17868	0.06617
ASXL1	0.02	Phospholipids.in.Small.LDL...Instance.0	0.007036	-0.10516	0.039019
ASXL1	0.1	Phospholipids.to.Total.Lipids.in.Very.Large.HDL.percentage...Instance.0	0.007154	0.17411	0.064734
overall	0.02	Cholesteryl.Esters.in.Large.LDL...Instance.0	0.007188	-0.02002	0.007449
TET2	0.1	Total.Lipids.in.Small.HDL...Instance.0	0.00737	-0.10386	0.038759
overall	0.02	Cholesterol.in.Large.LDL...Instance.0	0.007374	-0.01987	0.007416
overall	0.1	Total.Lipids.in.Medium.VLDL...Instance.0	0.007391	-0.03526	0.013161
ASXL1	0.1	Glucose...Instance.0	0.007528	-0.16881	0.063165
overall	0.02	Cholesteryl.Esters.in.LDL...Instance.0	0.007579	-0.02005	0.007508
TET2	0.1	Cholesteryl.Esters.to.Total.Lipids.in.Large.LDL.percentage...Instance.0	0.007598	-0.1064	0.039857
TET2	0.1	Total.Concentration.of.Lipoprotein.Particles...Instance.0	0.007673	-0.09598	0.035998
overall	0.02	LDL.Cholesterol...Instance.0	0.007778	-0.01992	0.007486
overall	0.02	Total.Lipids.in.Large.LDL...Instance.0	0.008011	-0.01971	0.007434
DNMT3A	0.1	Lactate...Instance.0	0.008156	0.061754	0.023343
overall	0.02	Total.Lipids.in.Lipoprotein.Particles...Instance.0	0.008281	-0.01957	0.007413
ASXL1	0.1	Concentration.of.IDL.Particles...Instance.0	0.008457	-0.17919	0.06805
overall	0.02	Total.Lipids.in.LDL...Instance.0	0.00849	-0.01974	0.007501
ASXL1	0.02	Lactate...Instance.0	0.008522	0.104155	0.039592
ASXL1	0.02	Omega.3.Fatty.Acids.to.Total.Fatty.Acids.percentage...Instance.0	0.008536	-0.10027	0.038124
TET2	0.1	Cholesteryl.Esters.in.Small.HDL...Instance.0	0.008589	-0.10315	0.03925
ASXL1	0.1	Concentration.of.Small.LDL.Particles...Instance.0	0.008594	-0.18222	0.069343
ASXL1	0.02	Free.Cholesterol.in.LDL...Instance.0	0.008713	-0.10028	0.038231
ASXL1	0.1	Cholesteryl.Esters.to.Total.Lipids.in.Small.HDL.percentage...Instance.0	0.008869	-0.18204	0.069557
overall	0.1	Spectrometer.corrected.alanine...Instance.0	0.008923	-0.03433	0.013129

ASXL1	0.1	Total.Free.Cholesterol...Instance.0	0.008981	-0.17568	0.067239
overall	0.02	Total.Phospholipids.in.Lipoprotein.Particles...Instance.0	0.009061	-0.01852	0.007098
overall	0.1	Triglycerides.in.Medium.VLDL...Instance.0	0.009231	-0.03353	0.012877
ASXL1	0.02	Phospholipids.in.LDL...Instance.0	0.009646	-0.10021	0.038717
TET2	0.1	Phospholipids.in.Small.HDL...Instance.0	0.009663	-0.09966	0.038515
overall	0.02	Free.Cholesterol.in.Large.LDL...Instance.0	0.009766	-0.01891	0.007318
overall	0.1	Cholesteryl.Esters.to.Total.Lipids.in.Large.LDL.percentage...Instance.0	0.009777	-0.03455	0.013374
TET2	0.1	Valine...Instance.0	0.009825	-0.0981	0.037993
TET2	0.1	Concentration.of.HDL.Particles...Instance.0	0.009861	-0.0925	0.035842
overall	0.1	Total.Fatty.Acids...Instance.0	0.010137	-0.0339	0.013185
ASXL1	0.02	Docosahexaenoic.Acid.to.Total.Fatty.Acids.percentage...Instance.0	0.01019	-0.09602	0.037372
overall	0.02	Cholesterol.in.Medium.LDL...Instance.0	0.010337	-0.01933	0.007536
ASXL1	0.02	Total.Lipids.in.Medium.LDL...Instance.0	0.010467	-0.09962	0.038913
overall	0.1	Phosphoglycerides...Instance.0	0.010509	-0.0318	0.012428
overall	0.1	Total.Cholines...Instance.0	0.010542	-0.03143	0.012291
ASXL1	0.02	LDL.Cholesterol...Instance.0	0.01055	-0.09882	0.038641
ASXL1	0.02	Cholesterol.in.Medium.LDL...Instance.0	0.010729	-0.09925	0.038901
overall	0.02	Cholesteryl.Esters.in.Medium.LDL...Instance.0	0.010783	-0.01919	0.007526
TET2	0.1	Docosahexaenoic.Acid.to.Total.Fatty.Acids.percentage...Instance.0	0.010796	0.096535	0.037868
overall	0.1	Total.Free.Cholesterol...Instance.0	0.010938	-0.03269	0.012847
overall	0.02	Concentration.of.Medium.LDL.Particles...Instance.0	0.010974	-0.01916	0.007533
ASXL1	0.02	Cholesteryl.Esters.in.Large.LDL...Instance.0	0.01106	-0.09769	0.03845
overall	0.02	Free.Cholesterol.in.LDL...Instance.0	0.011095	-0.01881	0.007405
ASXL1	0.1	Remnant.Cholesterol..Non.HDL..Non.LDL..Cholesterol....Instance.0	0.011148	-0.17389	0.068513
overall	0.1	Alanine...Instance.0	0.011231	-0.03364	0.013268
overall	0.02	Total.Lipids.in.Medium.LDL...Instance.0	0.011299	-0.0191	0.007539
ASXL1	0.1	Triglycerides.to.Total.Lipids.in.Small.LDL.percentage...Instance.0	0.01133	0.169542	0.06695
ASXL1	0.02	Cholesterol.in.Large.LDL...Instance.0	0.011402	-0.09685	0.038279

ASXL1	0.02	Total.Lipids.in.LDL...Instance.0	0.011595	-0.09773	0.038715
overall	0.02	Total.Cholesterol.Minus.HDL.C...Instance.0	0.011628	-0.01883	0.007461
overall	0.1	Total.Cholesterol...Instance.0	0.011656	-0.0319	0.012647
TET2	0.1	Free.Cholesterol.in.Small.HDL...Instance.0	0.0117	-0.0965	0.038278
overall	0.02	Phospholipids.in.LDL...Instance.0	0.011921	-0.01886	0.007501
ASXL1	0.02	Omega.6.Fatty.Acids.to.Omega.3.Fatty.Acids.ratio...Instance.0	0.011929	0.096401	0.038342
overall	0.1	Triglycerides.in.Large.VLDL...Instance.0	0.012217	-0.03185	0.012709
overall	0.1	Concentration.of.Medium.VLDL.Particles...Instance.0	0.012228	-0.03309	0.013205
ASXL1	0.02	Cholesteryl.Esters.in.LDL...Instance.0	0.012477	-0.09682	0.038754
overall	0.1	Total.Esterified.Cholesterol...Instance.0	0.012578	-0.03136	0.012566
ASXL1	0.1	Free.Cholesterol.in.Small.VLDL...Instance.0	0.012682	-0.17276	0.06931
overall	0.02	Phospholipids.in.Large.LDL...Instance.0	0.01293	-0.01847	0.007429
DNMT3A	0.1	Linoleic.Acid.to.Total.Fatty.Acids.percentage...Instance.0	0.013085	-0.05269	0.021234
overall	0.02	Total.Lipids.in.Small.LDL...Instance.0	0.013101	-0.01872	0.007544
ASXL1	0.02	Total.Lipids.in.Large.LDL...Instance.0	0.013335	-0.09496	0.038371
overall	0.1	Cholesteryl.Esters.in.LDL...Instance.0	0.013362	-0.03266	0.013201
overall	0.02	Clinical.LDL.Cholesterol...Instance.0	0.013388	-0.01842	0.007449
ASXL1	0.02	Free.Cholesterol.in.Small.HDL...Instance.0	0.013505	-0.09335	0.03779
overall	0.02	Cholesterol.in.Small.LDL...Instance.0	0.013524	-0.01861	0.007537
overall	0.02	Phospholipids.in.Small.LDL...Instance.0	0.013546	-0.01867	0.007561
overall	0.1	Phospholipids.in.Medium.VLDL...Instance.0	0.014035	-0.03248	0.013221
overall	0.1	Cholesteryl.Esters.in.Large.LDL...Instance.0	0.014213	-0.03211	0.013097
overall	0.02	Phospholipids.in.Medium.LDL...Instance.0	0.014324	-0.01849	0.007549
ASXL1	0.02	Clinical.LDL.Cholesterol...Instance.0	0.014601	-0.0939	0.038451
ASXL1	0.02	Total.Lipids.in.Small.LDL...Instance.0	0.014685	-0.09501	0.038937
ASXL1	0.1	Cholesterol.to.Total.Lipids.in.Medium.LDL.percentage...Instance.0	0.014807	-0.16616	0.06818
overall	0.1	Cholesteryl.Esters.in.Medium.LDL...Instance.0	0.01495	-0.0322	0.013232
ASXL1	0.02	Free.Cholesterol.in.Large.LDL...Instance.0	0.014975	-0.09191	0.037776

overall	0.02	Phosphatidylcholines...Instance.0	0.015027	-0.01693	0.006961
ASXL1	0.1	Phospholipids.to.Total.Lipids.in.Small.VLDL.percentage...Instance.0	0.015153	-0.15833	0.065191
TET2	0.1	Polyunsaturated.Fatty.Acids...Instance.0	0.01528	-0.09393	0.038723
overall	0.02	Free.Cholesterol.in.Medium.LDL...Instance.0	0.015318	-0.01823	0.007516
overall	0.02	Sphingomyelins...Instance.0	0.015442	-0.01682	0.006945
ASXL1	0.02	Cholesterol.in.Small.LDL...Instance.0	0.015487	-0.09419	0.038907
overall	0.1	Free.Cholesterol.in.Medium.VLDL...Instance.0	0.015679	-0.03198	0.013237
overall	0.02	Cholesteryl.Esters.in.Small.LDL...Instance.0	0.015681	-0.0182	0.00753
overall	0.02	Concentration.of.LDL.Particles...Instance.0	0.015765	-0.01817	0.007527
overall	0.02	Total.Cholines...Instance.0	0.015814	-0.01687	0.006989
ASXL1	0.02	Phospholipids.in.Large.LDL...Instance.0	0.015999	-0.09238	0.038347
TET2	0.1	Phosphatidylcholines...Instance.0	0.016137	-0.08753	0.036384
overall	0.02	Cholesterol.in.Medium.VLDL...Instance.0	0.016348	-0.01796	0.007482
overall	0.02	Phospholipids.in.IDL...Instance.0	0.016447	-0.01728	0.007204
ASXL1	0.02	Phospholipids.to.Total.Lipids.in.Small.HDL.percentage...Instance.0	0.016743	0.09129	0.03816
overall	0.1	Docosahexaenoic.Acid.to.Total.Fatty.Acids.percentage...Instance.0	0.016872	0.03046	0.012748
overall	0.02	Concentration.of.Small.LDL.Particles...Instance.0	0.017034	-0.01798	0.007535
ASXL1	0.1	Free.Cholesterol.to.Total.Lipids.in.Small.LDL.percentage...Instance.0	0.017191	-0.16108	0.067605
ASXL1	0.1	Free.Cholesterol.in.Medium.VLDL...Instance.0	0.017199	-0.16504	0.069272
overall	0.1	Total.Concentration.of.Lipoprotein.Particles...Instance.0	0.017532	-0.02875	0.012105
ASXL1	0.02	Cholesteryl.Esters.in.Medium.LDL...Instance.0	0.017676	-0.09216	0.038847
overall	0.1	Acetone...Instance.0	0.017831	0.031894	0.013462
overall	0.1	Free.Cholesterol.in.Small.HDL...Instance.0	0.017905	-0.03047	0.012871
overall	0.02	Free.Cholesterol.in.Medium.VLDL...Instance.0	0.017958	-0.01782	0.007529
overall	0.1	LDL.Cholesterol...Instance.0	0.018034	-0.03113	0.013162
ASXL1	0.1	Apolipoprotein.B.to.Apolipoprotein.A1.ratio...Instance.0	0.018088	-0.15912	0.067316
overall	0.1	Cholesterol.in.Medium.LDL...Instance.0	0.018261	-0.03127	0.013249
ASXL1	0.1	Free.Cholesterol.to.Total.Lipids.in.Very.Small.VLDL.percentage...Instance.0	0.018548	-0.16213	0.068859

overall	0.02	Apolipoprotein.B...Instance.0	0.018566	-0.01768	0.007511
overall	0.02	Polyunsaturated.Fatty.Acids...Instance.0	0.018778	-0.01742	0.007413
overall	0.02	Free.Cholesterol.in.Small.LDL...Instance.0	0.019156	-0.01763	0.007527
overall	0.02	Valine...Instance.0	0.019294	-0.01699	0.00726
ASXL1	0.02	Total.Esterified.Cholesterol...Instance.0	0.01941	-0.08622	0.036885
overall	0.1	Total.Lipids.in.Large.VLDL...Instance.0	0.019447	-0.0297	0.012708
overall	0.1	Cholesterol.in.Large.LDL...Instance.0	0.01951	-0.03045	0.013039
overall	0.02	Free.Cholesterol.in.Small.VLDL...Instance.0	0.019534	-0.01759	0.007533
TET2	0.1	Total.Concentration.of.Branched.Chain.Amino.Acids..Leucine...Isoleucine...Valine....Instance.0	0.019874	-0.08887	0.038163
overall	0.02	Total.Lipids.in.IDL...Instance.0	0.020051	-0.01672	0.007191
ASXL1	0.1	Free.Cholesterol.to.Total.Lipids.in.Small.VLDL.percentage...Instance.0	0.020112	-0.15037	0.064697
overall	0.1	Total.Lipids.in.Small.LDL...Instance.0	0.020685	-0.03069	0.013264
overall	0.1	Total.Lipids.in.Medium.LDL...Instance.0	0.021036	-0.03058	0.013254
overall	0.02	Cholesteryl.Esters.in.Medium.VLDL...Instance.0	0.021092	-0.01706	0.007398
overall	0.1	Total.Lipids.in.LDL...Instance.0	0.021236	-0.03038	0.013187
overall	0.02	Free.Cholesterol.in.Small.HDL...Instance.0	0.021421	-0.01684	0.007321
TET2	0.1	Average.Diameter.for.VLDL.Particles...Instance.0	0.021513	-0.08237	0.035831
ASXL1	0.1	Triglycerides.to.Total.Lipids.in.Small.VLDL.percentage...Instance.0	0.02153	0.152683	0.066425
DNMT3A	0.1	Alanine...Instance.0	0.02153	-0.05281	0.022977
overall	0.1	Cholesteryl.Esters.in.Small.LDL...Instance.0	0.022001	-0.03032	0.013239
ASXL1	0.1	Phospholipids.in.IDL...Instance.0	0.022228	-0.15157	0.066289
TET2	0.1	Spectrometer.corrected.alanine...Instance.0	0.022443	-0.08915	0.039053
overall	0.02	Remnant.Cholesterol..Non.HDL..Non.LDL..Cholesterol....Instance.0	0.022693	-0.01697	0.007447
TET2	0.02	Albumin...Instance.0	0.022762	0.041719	0.018318
TET2	0.1	Phosphoglycerides...Instance.0	0.022785	-0.0841	0.036932
overall	0.1	Total.Cholesterol.Minus.HDL.C...Instance.0	0.022837	-0.02986	0.013117
overall	0.02	Total.Concentration.of.Lipoprotein.Particles...Instance.0	0.022855	-0.01566	0.006883
ASXL1	0.1	Degree.of.Unsaturation...Instance.0	0.023033	-0.14854	0.065353

overall	0.1	Free.Cholesterol.to.Total.Lipids.in.Chylomicrons.and.Extremely.Large.VLDL.percentage...Instance.0	0.023173	0.03088	0.0136
overall	0.02	Concentration.of.Medium.VLDL.Particles...Instance.0	0.023186	-0.01705	0.007512
overall	0.02	Concentration.of.Large.LDL.Particles...Instance.0	0.023361	-0.01704	0.007517
overall	0.1	Phospholipids.in.Small.LDL...Instance.0	0.023503	-0.03011	0.013292
overall	0.1	Cholesterol.in.Small.LDL...Instance.0	0.023663	-0.02998	0.013253
overall	0.02	Acetone...Instance.0	0.023924	0.017315	0.007667
overall	0.1	Total.Lipids.in.Large.LDL...Instance.0	0.024061	-0.02949	0.013071
TET2	0.1	Total.Phospholipids.in.Lipoprotein.Particles...Instance.0	0.024098	-0.08368	0.037101
overall	0.1	Cholesteryl.Esters.to.Total.Lipids.in.Very.Large.VLDL.percentage...Instance.0	0.024245	0.028677	0.012727
overall	0.1	Lactate...Instance.0	0.024726	0.030293	0.013489
overall	0.02	Phospholipids.in.Medium.VLDL...Instance.0	0.024826	-0.01688	0.007521
overall	0.1	Concentration.of.LDL.Particles...Instance.0	0.024925	-0.02968	0.013233
TET2	0.1	Total.Cholines...Instance.0	0.024926	-0.08191	0.036525
overall	0.1	Concentration.of.Small.HDL.Particles...Instance.0	0.025181	-0.02942	0.013143
overall	0.02	Phosphoglycerides...Instance.0	0.025432	-0.01579	0.007067
overall	0.1	Total.Lipids.in.VLDL...Instance.0	0.025717	-0.02883	0.012926
overall	0.02	Cholesterol.in.IDL...Instance.0	0.02598	-0.01592	0.007148
overall	0.1	Saturated.Fatty.Acids...Instance.0	0.026115	-0.02918	0.013119
overall	0.1	Concentration.of.Medium.LDL.Particles...Instance.0	0.026207	-0.02944	0.013244
ASXL1	0.1	Cholesteryl.Esters.in.Small.HDL...Instance.0	0.026255	-0.15357	0.0691
overall	0.02	Cholesteryl.Esters.in.IDL...Instance.0	0.026329	-0.01587	0.007146
ASXL1	0.1	Cholesterol.in.Small.VLDL...Instance.0	0.026478	-0.15348	0.06916
overall	0.1	Cholesterol.to.Total.Lipids.in.Very.Large.VLDL.percentage...Instance.0	0.026692	0.028071	0.012667
TET2	0.1	Total.Fatty.Acids...Instance.0	0.026879	-0.08673	0.039186
ASXL1	0.02	Total.Cholesterol...Instance.0	0.026932	-0.08213	0.037122
overall	0.1	Phospholipids.in.Medium.LDL...Instance.0	0.027263	-0.0293	0.013271
ASXL1	0.02	Cholesteryl.Esters.to.Total.Lipids.in.IDL.percentage...Instance.0	0.027347	-0.08116	0.036783
ASXL1	0.02	Phospholipids.to.Total.Lipids.in.Very.Small.VLDL.percentage...Instance.0	0.02761	0.081554	0.037023

TET2	0.1	Total.Lipids.in.Lipoprotein.Particles...Instance.0	0.027636	-0.08533	0.038742
overall	0.1	Phospholipids.in.Small.VLDL...Instance.0	0.027662	-0.02911	0.013218
TET2	0.1	Alanine...Instance.0	0.027728	-0.08685	0.039458
DNMT3A	0.02	Phospholipids.to.Total.Lipids.in.Very.Large.VLDL.percentage...Instance.0	0.027743	-0.02644	0.012014
TET2	0.02	Tyrosine...Instance.0	0.028118	-0.04031	0.018358
overall	0.02	Phospholipids.in.Small.VLDL...Instance.0	0.028762	-0.01644	0.00752
DNMT3A	0.02	Phospholipids.to.Total.Lipids.in.Medium.LDL.percentage...Instance.0	0.028901	0.02621	0.011996
overall	0.1	Free.Cholesterol.in.VLDL...Instance.0	0.028945	-0.0286	0.013095
ASXL1	0.1	Cholesterol.to.Total.Lipids.in.Small.VLDL.percentage...Instance.0	0.028974	-0.14648	0.067075
overall	0.02	Free.Cholesterol.in.IDL...Instance.0	0.02913	-0.01566	0.007179
overall	0.1	Concentration.of.Small.LDL.Particles...Instance.0	0.029224	-0.02889	0.013249
overall	0.02	Total.Lipids.in.Medium.VLDL...Instance.0	0.029266	-0.01632	0.007487
DNMT3A	0.02	Linoleic.Acid...Instance.0	0.029747	-0.02565	0.0118
overall	0.1	Concentration.of.Large.LDL.Particles...Instance.0	0.029755	-0.02872	0.013216
overall	0.1	Total.Lipids.in.Small.HDL...Instance.0	0.029951	-0.02829	0.013031
overall	0.1	Phospholipids.in.LDL...Instance.0	0.029959	-0.02863	0.013188
overall	0.1	Cholesterol.in.Small.HDL...Instance.0	0.030361	-0.02838	0.013107
overall	0.1	Triglycerides.in.VLDL...Instance.0	0.030572	-0.02749	0.012711
DNMT3A	0.1	Linoleic.Acid...Instance.0	0.030597	-0.04935	0.022822
ASXL1	0.1	Sphingomyelins...Instance.0	0.031328	-0.13755	0.06389
overall	0.1	Monounsaturated.Fatty.Acids...Instance.0	0.031654	-0.028	0.013029
ASXL1	0.02	Cholesteryl.Esters.in.IDL...Instance.0	0.032199	-0.07899	0.036878
DNMT3A	0.02	Cholesteryl.Esters.to.Total.Lipids.in.Very.Large.VLDL.percentage...Instance.0	0.032397	0.024396	0.011403
overall	0.1	Concentration.of.Large.VLDL.Particles...Instance.0	0.032517	-0.02712	0.012687
ASXL1	0.02	Cholesteryl.Esters.in.Small.LDL...Instance.0	0.032934	-0.0829	0.038866
overall	0.1	Cholesteryl.Esters.to.Total.Lipids.in.Medium.LDL.percentage...Instance.0	0.033084	-0.0279	0.013091
overall	0.1	Cholesterol.in.Large.VLDL...Instance.0	0.033806	-0.02725	0.012837
overall	0.1	Free.Cholesterol.in.Small.VLDL...Instance.0	0.03404	-0.02807	0.013244

overall	0.1	Concentration.of.HDL.Particles...Instance.0	0.034148	-0.02553	0.012053
overall	0.1	Apolipoprotein.B...Instance.0	0.034207	-0.02796	0.013204
ASXL1	0.02	Degree.of.Unsaturation...Instance.0	0.034315	-0.07756	0.036647
ASXL1	0.1	Concentration.of.Medium.VLDL.Particles...Instance.0	0.034405	-0.14618	0.069106
ASXL1	0.1	Cholesterol.to.Total.Lipids.in.Large.LDL.percentage...Instance.0	0.034536	-0.13905	0.065785
overall	0.1	Phospholipids.in.VLDL...Instance.0	0.034562	-0.02753	0.013025
overall	0.1	Total.Lipids.in.Small.VLDL...Instance.0	0.03472	-0.02768	0.013107
ASXL1	0.1	Free.Cholesterol.to.Total.Lipids.in.Medium.VLDL.percentage...Instance.0	0.034815	-0.13626	0.06456
overall	0.1	Cholesteryl.Esters.in.Large.VLDL...Instance.0	0.034817	-0.02745	0.013008
TET2	0.1	Apolipoprotein.A1...Instance.0	0.034965	-0.07326	0.034739
ASXL1	0.1	Phospholipids.in.Medium.VLDL...Instance.0	0.035134	-0.14576	0.069187
ASXL1	0.02	Total.Lipids.in.Small.HDL...Instance.0	0.035827	-0.08031	0.038264
overall	0.1	Remnant.Cholesterol..Non.HDL..Non.LDL..Cholesterol....Instance.0	0.036397	-0.0274	0.013093
ASXL1	0.02	Total.Concentration.of.Lipoprotein.Particles...Instance.0	0.036406	-0.07435	0.035533
overall	0.1	Cholesterol.in.Medium.VLDL...Instance.0	0.03697	-0.02745	0.013157
ASXL1	0.1	Cholesterol.to.Total.Lipids.in.Very.Small.VLDL.percentage...Instance.0	0.037012	-0.13119	0.062902
overall	0.1	Clinical.LDL.Cholesterol...Instance.0	0.03711	-0.0273	0.013097
ASXL1	0.1	Cholesterol.in.Small.HDL...Instance.0	0.037333	-0.14292	0.068642
overall	0.1	Free.Cholesterol.in.Large.VLDL...Instance.0	0.037376	-0.02635	0.01266
ASXL1	0.1	Monounsaturated.Fatty.Acids.to.Total.Fatty.Acids.percentage...Instance.0	0.03758	0.131164	0.063077
overall	0.1	Phospholipids.in.Small.HDL...Instance.0	0.037852	-0.02689	0.01295
overall	0.1	Concentration.of.VLDL.Particles...Instance.0	0.038345	-0.02726	0.013162
ASXL1	0.02	Total.Cholesterol.Minus.HDL.C...Instance.0	0.038442	-0.0797	0.038501
ASXL1	0.02	Cholesterol.in.IDL...Instance.0	0.038559	-0.07632	0.036887
ASXL1	0.1	Phospholipids.in.Small.VLDL...Instance.0	0.038595	-0.14307	0.069168
overall	0.1	Phospholipids.in.Large.LDL...Instance.0	0.038823	-0.02699	0.013062
ASXL1	0.1	Tyrosine...Instance.0	0.038934	-0.14292	0.069215
ASXL1	0.1	Polyunsaturated.Fatty.Acids...Instance.0	0.038999	-0.14072	0.06817

overall	0.1	Glycoprotein.Acetyls...Instance.0	0.039009	-0.02593	0.012565
overall	0.02	Phenylalanine...Instance.0	0.039106	-0.01583	0.007671
TET2	0.1	Concentration.of.Medium.HDL.Particles...Instance.0	0.039502	-0.07208	0.035009
ASXL1	0.1	Concentration.of.Small.HDL.Particles...Instance.0	0.040018	-0.14135	0.068834
overall	0.02	Cholesterol.in.Small.VLDL...Instance.0	0.040068	-0.01544	0.007518
ASXL1	0.02	Glucose...Instance.0	0.040557	-0.07254	0.035417
ASXL1	0.1	Cholesteryl.Esters.in.Small.VLDL...Instance.0	0.041575	-0.14058	0.068988
DNMT3A	0.1	Saturated.Fatty.Acids.to.Total.Fatty.Acids.percentage...Instance.0	0.041587	0.046081	0.022615
TET2	0.1	Cholesteryl.Esters.in.Large.LDL...Instance.0	0.041653	-0.07933	0.038945
overall	0.1	Triglycerides.in.Small.VLDL...Instance.0	0.041812	-0.02623	0.012888
DNMT3A	0.02	Glucose...Instance.0	0.042445	-0.02194	0.01081
DNMT3A	0.02	Polyunsaturated.Fatty.Acids.to.Monounsaturated.Fatty.Acids.ratio...Instance.0	0.04273	0.021995	0.010854
overall	0.02	Cholesteryl.Esters.in.VLDL...Instance.0	0.043252	-0.01518	0.00751
ASXL1	0.1	Free.Cholesterol.to.Total.Lipids.in.Very.Large.HDL.percentage...Instance.0	0.043457	-0.12345	0.061137
TET2	0.1	Total.Lipids.in.Medium.HDL...Instance.0	0.043553	-0.07158	0.035466
ASXL1	0.1	Phospholipids.to.Total.Lipids.in.Medium.VLDL.percentage...Instance.0	0.043918	-0.13195	0.065487
ASXL1	0.1	Free.Cholesterol.to.Total.Lipids.in.Large.LDL.percentage...Instance.0	0.04392	-0.12858	0.063814
ASXL1	0.02	Cholesterol.to.Total.Lipids.in.Small.HDL.percentage...Instance.0	0.044453	-0.07763	0.038628
overall	0.1	VLDL.Cholesterol...Instance.0	0.044569	-0.02648	0.013184
TET2	0.02	Linoleic.Acid.to.Total.Fatty.Acids.percentage...Instance.0	0.04458	-0.0342	0.017027
TET2	0.1	Total.Esterified.Cholesterol...Instance.0	0.044585	-0.07504	0.037362
overall	0.1	Phospholipids.in.Large.VLDL...Instance.0	0.045186	-0.0253	0.012633
ASXL1	0.1	Omega.6.Fatty.Acids...Instance.0	0.045362	-0.13713	0.068521
overall	0.1	Free.Cholesterol.in.Medium.LDL...Instance.0	0.045958	-0.02638	0.013217
overall	0.1	Triglycerides.in.Very.Large.VLDL...Instance.0	0.045984	-0.02506	0.01256
DNMT3A	0.02	Omega.6.Fatty.Acids...Instance.0	0.046099	-0.02338	0.011721
ASXL1	0.02	Polyunsaturated.Fatty.Acids...Instance.0	0.046162	-0.07623	0.038231
overall	0.02	Cholesterol.in.Very.Small.VLDL...Instance.0	0.046236	-0.01454	0.007295

overall	0.1	Total.Triglycerides...Instance.0	0.046628	-0.02547	0.012803
overall	0.1	Cholesteryl.Esters.in.Small.HDL...Instance.0	0.046637	-0.02625	0.013194
ASXL1	0.1	Cholesteryl.Esters.in.VLDL...Instance.0	0.046839	-0.13733	0.069089
DNMT3A	0.02	Total.Lipids.in.Lipoprotein.Particles...Instance.0	0.046991	-0.02317	0.011664
overall	0.1	Total.Lipids.in.Very.Large.VLDL...Instance.0	0.04728	-0.025	0.012603
overall	0.1	Free.Cholesterol.in.LDL...Instance.0	0.047511	-0.0258	0.013021
overall	0.02	Cholesteryl.Esters.in.Very.Small.VLDL...Instance.0	0.047512	-0.01431	0.007223
TET2	0.1	Cholesteryl.Esters.in.LDL...Instance.0	0.047941	-0.07764	0.039254
TET2	0.1	Phospholipids.in.Medium.HDL...Instance.0	0.047992	-0.07109	0.03595
overall	0.02	Free.Cholesterol.to.Total.Lipids.in.Small.HDL.percentage...Instance.0	0.04844	-0.01408	0.007136
overall	0.02	Concentration.of.HDL.Particles...Instance.0	0.048475	-0.01352	0.006853
DNMT3A	0.02	Total.Phospholipids.in.Lipoprotein.Particles...Instance.0	0.048482	-0.02204	0.01117
overall	0.02	VLDL.Cholesterol...Instance.0	0.048645	-0.01479	0.007499
overall	0.02	Concentration.of.IDL.Particles...Instance.0	0.048765	-0.01457	0.007395
overall	0.1	Concentration.of.Small.VLDL.Particles...Instance.0	0.048855	-0.02576	0.013078
ASXL1	0.02	Sphingomyelins...Instance.0	0.049275	-0.07044	0.035826

Supplementary Table S32: Metabolites significantly associated with AP and its subtypes

Disease	Metabolite	P value	Beta	SE
AP	Cholesterol.in.IDL...Instance.0	1.07E-128	-0.00809	0.000335
AP	Cholesteryl.Esters.in.IDL...Instance.0	3.25E-128	-0.00808	0.000335
AP	Free.Cholesterol.in.IDL...Instance.0	2.47E-123	-0.00789	0.000334
AP	Total.Lipids.in.IDL...Instance.0	2.42E-119	-0.00774	0.000333
AP	Cholesterol.to.Total.Lipids.in.Very.Small.VLDL.percentage...Instance.0	1.57E-115	-0.00802	0.000351
AP	Cholesteryl.Esters.to.Total.Lipids.in.Very.Small.VLDL.percentage...Instance.0	1.01E-114	-0.00807	0.000354
AP	Phospholipids.in.IDL...Instance.0	4.61E-114	-0.00755	0.000333
AP	Free.Cholesterol.in.Large.LDL...Instance.0	2.19E-113	-0.00741	0.000327
AP	Total.Cholesterol...Instance.0	1.28E-112	-0.00752	0.000333
AP	Cholesteryl.Esters.in.Medium.VLDL...Instance.0	1.87E-112	-0.0073	0.000324
AP	Total.Esterified.Cholesterol...Instance.0	3.65E-112	-0.00755	0.000335
AP	Triglycerides.to.Total.Lipids.in.Very.Small.VLDL.percentage...Instance.0	8.45E-112	0.007812	0.000348
AP	Concentration.of.IDL.Particles...Instance.0	2.09E-110	-0.00724	0.000324
AP	Total.Free.Cholesterol...Instance.0	1.90E-109	-0.00729	0.000328
AP	Free.Cholesterol.in.LDL...Instance.0	4.25E-106	-0.00708	0.000324
AP	Clinical.LDL.Cholesterol...Instance.0	4.78E-106	-0.00704	0.000322
AP	Triglycerides.to.Total.Lipids.in.IDL.percentage...Instance.0	8.23E-106	0.007507	0.000343
AP	Phospholipids.to.Total.Lipids.in.Medium.VLDL.percentage...Instance.0	1.29E-104	-0.00732	0.000337
AP	Phospholipids.in.Large.LDL...Instance.0	8.33E-104	-0.00698	0.000323
AP	Remnant.Cholesterol..Non.HDL..Non.LDL..Cholesterol....Instance.0	1.37E-101	-0.00689	0.000322
AP	Total.Cholesterol.Minus.HDL.C...Instance.0	3.00E-101	-0.00686	0.000321
AP	Cholesteryl.Esters.in.Very.Small.VLDL...Instance.0	8.68E-101	-0.00707	0.000332
AP	Cholesterol.in.Medium.VLDL...Instance.0	5.94E-100	-0.0068	0.00032
AP	Cholesterol.in.Large.LDL...Instance.0	1.06E-99	-0.00685	0.000323
AP	Cholesterol.to.Total.Lipids.in.IDL.percentage...Instance.0	1.23E-99	-0.00717	0.000338

AP	Total.Lipids.in.Large.LDL...Instance.0	3.82E-96	-0.00671	0.000322
AP	Free.Cholesterol.to.Total.Lipids.in.Medium.VLDL.percentage...Instance.0	1.47E-94	-0.00706	0.000342
AP	LDL.Cholesterol...Instance.0	1.39E-93	-0.00657	0.00032
AP	Cholesteryl.Esters.in.Large.LDL...Instance.0	3.94E-93	-0.00659	0.000322
AP	Phospholipids.in.LDL...Instance.0	1.83E-92	-0.00652	0.000319
AP	Free.Cholesterol.to.Total.Lipids.in.Small.VLDL.percentage...Instance.0	8.96E-92	-0.00693	0.000341
AP	Free.Cholesterol.in.Medium.LDL...Instance.0	3.50E-90	-0.00642	0.000319
AP	Cholesterol.in.Very.Small.VLDL...Instance.0	5.84E-90	-0.00661	0.000329
AP	Triglycerides.to.Total.Lipids.in.Medium.VLDL.percentage...Instance.0	9.83E-90	0.006969	0.000347
AP	Concentration.of.Large.LDL.Particles...Instance.0	3.52E-89	-0.00639	0.000319
AP	Phospholipids.to.Total.Lipids.in.Small.VLDL.percentage...Instance.0	4.77E-89	-0.00678	0.000339
AP	Apolipoprotein.B...Instance.0	5.94E-89	-0.00638	0.000319
UAP	Cholesteryl.Esters.in.IDL...Instance.0	1.22E-88	-0.00458	0.000229
UAP	Cholesterol.in.IDL...Instance.0	1.44E-88	-0.00458	0.000229
AP	Total.Lipids.in.LDL...Instance.0	1.58E-88	-0.00638	0.00032
AP	Cholesteryl.Esters.to.Total.Lipids.in.IDL.percentage...Instance.0	3.46E-88	-0.0067	0.000336
AP	Free.Cholesterol.in.Small.LDL...Instance.0	4.45E-88	-0.00634	0.000318
AP	Triglycerides.to.Total.Lipids.in.Large.LDL.percentage...Instance.0	1.51E-87	0.006649	0.000335
AP	Cholesteryl.Esters.in.LDL...Instance.0	9.12E-87	-0.0063	0.000319
AP	Concentration.of.LDL.Particles...Instance.0	3.25E-86	-0.00627	0.000318
UAP	Total.Lipids.in.IDL...Instance.0	9.36E-85	-0.00445	0.000228
AP	Linoleic.Acid...Instance.0	1.50E-84	-0.00623	0.00032
AP	Cholesterol.to.Total.Lipids.in.Large.LDL.percentage...Instance.0	4.79E-84	-0.00655	0.000337
AP	Cholesterol.in.Small.LDL...Instance.0	6.79E-84	-0.00617	0.000318
UAP	Free.Cholesterol.in.IDL...Instance.0	8.61E-84	-0.00443	0.000228
UAP	Total.Cholesterol...Instance.0	1.84E-83	-0.00441	0.000228
AP	Triglycerides.to.Total.Lipids.in.Small.VLDL.percentage...Instance.0	2.45E-83	0.006429	0.000332
UAP	Total.Esterified.Cholesterol...Instance.0	1.08E-82	-0.00442	0.000229

UAP	Phospholipids.in.IDL...Instance.0	2.30E-82	-0.00438	0.000228
AP	Sphingomyelins...Instance.0	2.49E-82	-0.00664	0.000345
UAP	Total.Free.Cholesterol...Instance.0	3.63E-82	-0.00431	0.000224
AP	Cholesterol.to.Total.Lipids.in.Medium.VLDL.percentage...Instance.0	6.24E-82	-0.00669	0.000349
UAP	Free.Cholesterol.in.Large.LDL...Instance.0	7.08E-81	-0.00427	0.000224
AP	Cholesterol.to.Total.Lipids.in.Small.LDL.percentage...Instance.0	3.22E-80	-0.00611	0.000322
AP	Cholesterol.to.Total.Lipids.in.Medium.LDL.percentage...Instance.0	1.38E-78	-0.0061	0.000325
UAP	Cholesteryl.Esters.in.Medium.VLDL...Instance.0	1.59E-78	-0.00416	0.000222
UAP	Clinical.LDL.Cholesterol...Instance.0	2.73E-78	-0.00412	0.00022
UAP	Concentration.of.IDL.Particles...Instance.0	3.97E-78	-0.00415	0.000222
UAP	Phospholipids.in.Large.LDL...Instance.0	1.55E-77	-0.00411	0.000221
AP	Total.Lipids.in.Small.LDL...Instance.0	3.58E-77	-0.00591	0.000318
UAP	Total.Cholesterol.Minus.HDL.C...Instance.0	4.31E-77	-0.00408	0.00022
AP	Cholesterol.to.Total.Lipids.in.Small.VLDL.percentage...Instance.0	7.00E-77	-0.00611	0.000329
UAP	Free.Cholesterol.in.LDL...Instance.0	1.80E-76	-0.0041	0.000221
UAP	Remnant.Cholesterol..Non.HDL..Non.LDL..Cholesterol....Instance.0	3.22E-76	-0.00407	0.00022
AP	Phospholipids.in.Small.LDL...Instance.0	8.84E-76	-0.00584	0.000317
AP	Cholesteryl.Esters.in.Small.LDL...Instance.0	8.88E-76	-0.00587	0.000318
AP	Cholesteryl.Esters.to.Total.Lipids.in.Medium.VLDL.percentage...Instance.0	2.26E-75	-0.00644	0.00035
AP	Cholesterol.in.Medium.LDL...Instance.0	2.66E-75	-0.00584	0.000318
UAP	Cholesterol.in.Large.LDL...Instance.0	3.90E-75	-0.00406	0.000221
AP	Concentration.of.Small.LDL.Particles...Instance.0	5.40E-75	-0.00583	0.000318
AP	Free.Cholesterol.in.Medium.VLDL...Instance.0	7.49E-74	-0.00579	0.000318
UAP	Total.Lipids.in.Large.LDL...Instance.0	1.83E-73	-0.004	0.00022
UAP	Cholesterol.in.Medium.VLDL...Instance.0	3.54E-73	-0.00397	0.000219
AP	Total.Lipids.in.Lipoprotein.Particles...Instance.0	1.57E-72	-0.00583	0.000323
UAP	LDL.Cholesterol...Instance.0	2.63E-72	-0.00394	0.000219
UAP	Cholesteryl.Esters.in.Large.LDL...Instance.0	1.01E-71	-0.00394	0.00022

UAP	Cholesteryl.Esters.in.Very.Small.VLDL...Instance.0	2.39E-71	-0.00405	0.000227
UAP	Phospholipids.in.LDL...Instance.0	8.66E-71	-0.00389	0.000219
AP	Omega.6.Fatty.Acids...Instance.0	1.17E-70	-0.00572	0.000322
AP	Cholesteryl.Esters.in.VLDL...Instance.0	1.31E-70	-0.00567	0.000319
AP	Phospholipids.in.Medium.LDL...Instance.0	1.98E-70	-0.00564	0.000318
AP	Total.Phospholipids.in.Lipoprotein.Particles...Instance.0	3.29E-70	-0.00598	0.000338
AP	Concentration.of.Medium.LDL.Particles...Instance.0	4.54E-70	-0.00563	0.000318
AP	Total.Lipids.in.Medium.LDL...Instance.0	5.44E-70	-0.00562	0.000318
AP	Triglycerides.to.Total.Lipids.in.Medium.LDL.percentage...Instance.0	6.52E-70	0.005787	0.000327
AP	Linoleic.Acid.to.Total.Fatty.Acids.percentage...Instance.0	1.48E-69	-0.00606	0.000344
UAP	Total.Lipids.in.LDL...Instance.0	2.23E-69	-0.00385	0.000219
UAP	Linoleic.Acid...Instance.0	3.90E-69	-0.00384	0.000219
AP	Free.Cholesterol.to.Total.Lipids.in.Very.Small.VLDL.percentage...Instance.0	5.75E-69	-0.00563	0.000321
AP	Free.Cholesterol.in.Small.VLDL...Instance.0	6.00E-69	-0.00559	0.000318
UAP	Cholesteryl.Esters.in.LDL...Instance.0	6.32E-69	-0.00383	0.000218
AP	Free.Cholesterol.to.Total.Lipids.in.Large.LDL.percentage...Instance.0	1.50E-68	-0.00605	0.000346
UAP	Apolipoprotein.B...Instance.0	2.89E-68	-0.00381	0.000218
AP	Free.Cholesterol.to.Total.Lipids.in.Large.HDL.percentage...Instance.0	6.74E-68	-0.006	0.000345
UAP	Phospholipids.to.Total.Lipids.in.Medium.VLDL.percentage...Instance.0	2.03E-67	-0.004	0.00023
UAP	Free.Cholesterol.in.Medium.LDL...Instance.0	3.74E-67	-0.00378	0.000218
UAP	Concentration.of.Large.LDL.Particles...Instance.0	3.93E-67	-0.00378	0.000218
AP	Cholesteryl.Esters.in.Medium.LDL...Instance.0	1.44E-66	-0.00549	0.000319
UAP	Concentration.of.LDL.Particles...Instance.0	1.52E-66	-0.00375	0.000218
UAP	Cholesterol.to.Total.Lipids.in.Large.LDL.percentage...Instance.0	2.18E-66	-0.00397	0.00023
UAP	Cholesterol.in.Small.LDL...Instance.0	5.60E-66	-0.00373	0.000217
UAP	Cholesterol.in.Very.Small.VLDL...Instance.0	3.08E-65	-0.00383	0.000225
UAP	Cholesterol.to.Total.Lipids.in.Very.Small.VLDL.percentage...Instance.0	8.49E-64	-0.00405	0.00024
UAP	Triglycerides.to.Total.Lipids.in.IDL.percentage...Instance.0	1.39E-63	0.003956	0.000235

UAP	Cholesteryl.Esters.to.Total.Lipids.in.Very.Small.VLDL.percentage...Instance.0	1.80E-63	-0.00408	0.000243
UAP	Free.Cholesterol.in.Small.LDL...Instance.0	1.36E-62	-0.00364	0.000218
UAP	Cholesteryl.Esters.in.Small.LDL...Instance.0	4.64E-62	-0.00362	0.000218
UAP	Cholesterol.in.Medium.LDL...Instance.0	8.54E-62	-0.00361	0.000218
AP	Free.Cholesterol.in.Very.Small.VLDL...Instance.0	1.05E-61	-0.00535	0.000323
UAP	Total.Lipids.in.Small.LDL...Instance.0	2.48E-61	-0.00359	0.000217
UAP	Triglycerides.to.Total.Lipids.in.Large.LDL.percentage...Instance.0	3.67E-61	0.003783	0.000229
AP	Phospholipids.to.Total.Lipids.in.Large.HDL.percentage...Instance.0	3.93E-61	0.005791	0.000351
AP	Phospholipids.in.Medium.VLDL...Instance.0	1.05E-60	-0.00524	0.000319
UAP	Triglycerides.to.Total.Lipids.in.Very.Small.VLDL.percentage...Instance.0	1.13E-60	0.003908	0.000238
UAP	Cholesterol.to.Total.Lipids.in.Medium.LDL.percentage...Instance.0	1.16E-60	-0.00365	0.000222
UAP	Total.Lipids.in.Lipoprotein.Particles...Instance.0	4.34E-60	-0.00362	0.000221
UAP	Cholesterol.to.Total.Lipids.in.Small.LDL.percentage...Instance.0	7.04E-60	-0.00359	0.00022
UAP	Cholesterol.to.Total.Lipids.in.IDL.percentage...Instance.0	2.89E-59	-0.00376	0.000231
UAP	Omega.6.Fatty.Acids...Instance.0	3.26E-59	-0.00357	0.00022
UAP	Concentration.of.Small.LDL.Particles...Instance.0	6.93E-59	-0.00352	0.000218
UAP	Total.Lipids.in.Medium.LDL...Instance.0	2.99E-58	-0.0035	0.000217
UAP	Free.Cholesterol.in.Medium.VLDL...Instance.0	3.72E-58	-0.0035	0.000218
AP	Polyunsaturated.Fatty.Acids...Instance.0	5.32E-58	-0.0052	0.000324
UAP	Phospholipids.in.Small.LDL...Instance.0	1.63E-57	-0.00347	0.000217
UAP	Sphingomyelins...Instance.0	1.95E-57	-0.00377	0.000236
AP	Free.Cholesterol.to.Total.Lipids.in.Medium.HDL.percentage...Instance.0	2.07E-57	-0.00604	0.000378
UAP	Phospholipids.in.Medium.LDL...Instance.0	2.62E-57	-0.00347	0.000217
UAP	Concentration.of.Medium.LDL.Particles...Instance.0	2.98E-57	-0.00347	0.000218
UAP	Total.Phospholipids.in.Lipoprotein.Particles...Instance.0	4.72E-57	-0.00368	0.000231
UAP	Cholesteryl.Esters.in.Medium.LDL...Instance.0	7.03E-57	-0.00346	0.000218
UAP	Free.Cholesterol.to.Total.Lipids.in.Medium.VLDL.percentage...Instance.0	7.83E-57	-0.00372	0.000234
AP	Total.Cholines...Instance.0	1.39E-56	-0.00544	0.000343

AP	VLDL.Cholesterol...Instance.0	1.50E-56	-0.00507	0.00032
UAP	Cholesteryl.Esters.in.VLDL...Instance.0	5.29E-56	-0.00344	0.000218
AP	Concentration.of.Medium.VLDL.Particles...Instance.0	5.49E-56	-0.00503	0.000319
UAP	Cholesteryl.Esters.to.Total.Lipids.in.IDL.percentage...Instance.0	3.06E-55	-0.0036	0.00023
AP	Concentration.of.Very.Small.VLDL.Particles...Instance.0	4.39E-55	-0.00502	0.000321
UAP	Free.Cholesterol.in.Small.VLDL...Instance.0	8.58E-55	-0.00339	0.000218
UAP	Free.Cholesterol.to.Total.Lipids.in.Small.VLDL.percentage...Instance.0	4.31E-53	-0.00358	0.000233
AP	Cholesteryl.Esters.to.Total.Lipids.in.Small.VLDL.percentage...Instance.0	7.65E-53	-0.00491	0.000321
UAP	Triglycerides.to.Total.Lipids.in.Medium.VLDL.percentage...Instance.0	7.93E-53	0.00363	0.000237
AP	Cholesterol.in.Small.VLDL...Instance.0	1.53E-52	-0.00487	0.000319
UAP	Phospholipids.to.Total.Lipids.in.Small.VLDL.percentage...Instance.0	4.78E-52	-0.00352	0.000232
UAP	Phospholipids.in.Medium.VLDL...Instance.0	3.47E-50	-0.00325	0.000218
AP	Total.Lipids.in.Very.Small.VLDL...Instance.0	1.02E-49	-0.00477	0.000322
UAP	Triglycerides.to.Total.Lipids.in.Small.VLDL.percentage...Instance.0	2.60E-49	0.003356	0.000227
UAP	Free.Cholesterol.in.Very.Small.VLDL...Instance.0	5.59E-48	-0.00321	0.000221
UAP	Polyunsaturated.Fatty.Acids...Instance.0	7.99E-48	-0.00321	0.000221
AP	Phospholipids.in.Small.VLDL...Instance.0	1.99E-47	-0.00461	0.000319
UAP	Triglycerides.to.Total.Lipids.in.Medium.LDL.percentage...Instance.0	2.99E-47	0.003233	0.000224
UAP	VLDL.Cholesterol...Instance.0	4.14E-47	-0.00315	0.000219
UAP	Cholesterol.to.Total.Lipids.in.Medium.VLDL.percentage...Instance.0	7.14E-47	-0.00343	0.000238
UAP	Free.Cholesterol.to.Total.Lipids.in.Large.HDL.percentage...Instance.0	1.22E-46	-0.00338	0.000236
UAP	Concentration.of.Medium.VLDL.Particles...Instance.0	1.42E-46	-0.00313	0.000218
UAP	Total.Cholines...Instance.0	1.62E-46	-0.00336	0.000235
AP	Phosphatidylcholines...Instance.0	2.72E-46	-0.00492	0.000345
UAP	Cholesterol.to.Total.Lipids.in.Small.VLDL.percentage...Instance.0	8.79E-46	-0.0032	0.000225
UAP	Linoleic.Acid.to.Total.Fatty.Acids.percentage...Instance.0	6.37E-45	-0.00331	0.000235
AP	Cholesterol.to.Total.Lipids.in.Large.HDL.percentage...Instance.0	2.04E-44	-0.00495	0.000354
UAP	Concentration.of.Very.Small.VLDL.Particles...Instance.0	2.46E-44	-0.00307	0.00022

AP	Apolipoprotein.B.to.Apolipoprotein.A1.ratio...Instance.0	5.00E-44	-0.00456	0.000328
UAP	Cholesterol.in.Small.VLDL...Instance.0	7.98E-44	-0.00303	0.000218
AP	Cholesteryl.Esters.in.Small.VLDL...Instance.0	3.83E-43	-0.0044	0.00032
UAP	Cholesteryl.Esters.to.Total.Lipids.in.Medium.VLDL.percentage...Instance.0	2.66E-42	-0.00327	0.00024
AP	Phosphoglycerides...Instance.0	3.80E-42	-0.00462	0.000339
UAP	Phospholipids.in.Small.VLDL...Instance.0	6.64E-41	-0.00292	0.000218
UAP	Total.Lipids.in.Very.Small.VLDL...Instance.0	8.82E-41	-0.00294	0.00022
AP	Total.Lipids.in.Medium.VLDL...Instance.0	1.52E-40	-0.00427	0.00032
UAP	Free.Cholesterol.to.Total.Lipids.in.Large.LDL.percentage...Instance.0	4.43E-40	-0.00314	0.000237
AP	Phospholipids.in.Very.Small.VLDL...Instance.0	1.50E-39	-0.00422	0.00032
UAP	Phospholipids.to.Total.Lipids.in.Large.HDL.percentage...Instance.0	2.49E-39	0.00315	0.00024
UAP	Phosphatidylcholines...Instance.0	3.78E-39	-0.00308	0.000236
AP	Phospholipids.to.Total.Lipids.in.Very.Small.VLDL.percentage...Instance.0	3.14E-38	0.004324	0.000334
UAP	Free.Cholesterol.to.Total.Lipids.in.Very.Small.VLDL.percentage...Instance.0	7.94E-38	-0.00282	0.000219
UAP	Free.Cholesterol.to.Total.Lipids.in.Medium.HDL.percentage...Instance.0	1.01E-37	-0.00332	0.000259
AP	Free.Cholesterol.in.VLDL...Instance.0	2.31E-37	-0.00411	0.000322
AP	Triglycerides.to.Total.Lipids.in.Small.LDL.percentage...Instance.0	2.32E-37	0.004219	0.00033
UAP	Cholesteryl.Esters.in.Small.VLDL...Instance.0	3.04E-37	-0.00279	0.000219
UAP	Phosphoglycerides...Instance.0	2.30E-36	-0.00292	0.000232
UAP	Total.Lipids.in.Medium.VLDL...Instance.0	3.09E-36	-0.00275	0.000219
AP	Concentration.of.VLDL.Particles...Instance.0	8.08E-35	-0.00394	0.00032
AP	Triglycerides.to.Total.Lipids.in.Very.Large.VLDL.percentage...Instance.0	9.61E-35	0.004073	0.000331
AP	Cholesteryl.Esters.to.Total.Lipids.in.Large.VLDL.percentage...Instance.0	5.54E-34	-0.00406	0.000334
UAP	Free.Cholesterol.in.VLDL...Instance.0	5.73E-34	-0.00267	0.00022
AP	Total.Concentration.of.Lipoprotein.Particles...Instance.0	5.77E-34	-0.00423	0.000348
AP	Free.Cholesterol.to.Total.Lipids.in.IDL.percentage...Instance.0	7.33E-34	-0.00389	0.000321
UAP	Phospholipids.in.Very.Small.VLDL...Instance.0	2.25E-33	-0.00264	0.000219
UAP	Apolipoprotein.B.to.Apolipoprotein.A1.ratio...Instance.0	5.23E-33	-0.00268	0.000224

UAP	Cholesteryl.Esters.to.Total.Lipids.in.Small.VLDL.percentage...Instance.0	1.72E-32	-0.0026	0.000219
UAP	Concentration.of.VLDL.Particles...Instance.0	8.08E-32	-0.00257	0.000219
AP	Cholesteryl.Esters.to.Total.Lipids.in.Large.HDL.percentage...Instance.0	4.14E-31	-0.00405	0.000349
AP	Free.Cholesterol.in.Very.Large.HDL...Instance.0	4.20E-31	-0.00406	0.00035
AP	Concentration.of.Very.Large.HDL.Particles...Instance.0	3.27E-30	-0.00416	0.000364
AP	Free.Cholesterol.to.Total.Lipids.in.Small.HDL.percentage...Instance.0	4.51E-30	-0.00383	0.000336
AP	Cholesterol.in.Very.Large.HDL...Instance.0	6.20E-30	-0.00417	0.000367
AP	Cholesteryl.Esters.in.Very.Large.VLDL...Instance.0	8.71E-30	-0.00371	0.000328
UAP	Total.Concentration.of.Lipoprotein.Particles...Instance.0	4.75E-29	-0.00267	0.000238
AP	Cholesteryl.Esters.in.Very.Large.HDL...Instance.0	7.48E-29	-0.00413	0.00037
UAP	Cholesterol.to.Total.Lipids.in.Large.HDL.percentage...Instance.0	2.84E-28	-0.00267	0.000242
UAP	Cholesteryl.Esters.in.Very.Large.VLDL...Instance.0	2.92E-28	-0.00247	0.000224
AP	Free.Cholesterol.to.Total.Lipids.in.Very.Large.VLDL.percentage...Instance.0	3.58E-28	-0.00363	0.00033
UAP	Cholesteryl.Esters.to.Total.Lipids.in.Large.LDL.percentage...Instance.0	7.12E-28	-0.00236	0.000216
AP	Cholesteryl.Esters.in.Large.VLDL...Instance.0	8.60E-28	-0.00354	0.000324
AP	Cholesterol.to.Total.Lipids.in.Large.VLDL.percentage...Instance.0	3.79E-27	-0.00348	0.000322
UAP	Cholesteryl.Esters.to.Total.Lipids.in.Small.LDL.percentage...Instance.0	6.20E-27	-0.00234	0.000218
AP	Cholesteryl.Esters.to.Total.Lipids.in.Small.LDL.percentage...Instance.0	7.30E-27	-0.00342	0.000319
UAP	Cholesteryl.Esters.in.Large.VLDL...Instance.0	5.36E-26	-0.00234	0.000222
AP	Phospholipids.in.VLDL...Instance.0	2.26E-25	-0.00337	0.000324
UAP	Phospholipids.in.VLDL...Instance.0	3.24E-25	-0.0023	0.000221
AP	Free.Cholesterol.in.HDL...Instance.0	5.26E-25	-0.00387	0.000374
UAP	Total.Fatty.Acids...Instance.0	1.41E-24	-0.00224	0.000219
UAP	Phospholipids.to.Total.Lipids.in.Very.Small.VLDL.percentage...Instance.0	1.78E-24	0.002336	0.000229
AP	Phospholipids.to.Total.Lipids.in.Medium.HDL.percentage...Instance.0	6.43E-24	0.003558	0.000353
AP	Cholesteryl.Esters.to.Total.Lipids.in.Large.LDL.percentage...Instance.0	6.68E-24	-0.00318	0.000316
AP	Total.Fatty.Acids...Instance.0	7.15E-24	-0.00322	0.00032
AP	Phospholipids.to.Total.Lipids.in.IDL.percentage...Instance.0	7.82E-23	0.003127	0.000318

AP	Total.Lipids.in.Very.Large.HDL...Instance.0	1.20E-22	-0.00358	0.000365
UAP	Total.Lipids.in.Small.VLDL...Instance.0	2.73E-22	-0.00214	0.00022
AP	Total.Lipids.in.Small.VLDL...Instance.0	5.68E-22	-0.0031	0.000322
UAP	Triglycerides.to.Total.Lipids.in.Small.LDL.percentage...Instance.0	1.19E-21	0.002159	0.000226
AP	Concentration.of.HDL.Particles...Instance.0	2.38E-20	-0.00324	0.00035
AP	Phospholipids.to.Total.Lipids.in.Very.Large.VLDL.percentage...Instance.0	2.50E-20	-0.00293	0.000318
AP	Free.Cholesterol.to.Total.Lipids.in.Medium.LDL.percentage...Instance.0	3.24E-20	-0.00312	0.000339
AP	HDL.Cholesterol...Instance.0	5.16E-20	-0.00345	0.000376
AP	Cholesterol.to.Total.Lipids.in.Very.Large.VLDL.percentage...Instance.0	8.36E-20	-0.00306	0.000336
AP	Average.Diameter.for.LDL.Particles...Instance.0	1.84E-19	-0.00297	0.000329
UAP	Free.Cholesterol.in.Small.HDL...Instance.0	2.04E-19	-0.00202	0.000224
UAP	Cholesteryl.Esters.to.Total.Lipids.in.Large.HDL.percentage...Instance.0	2.35E-19	-0.00215	0.000239
UAP	Cholesterol.in.Very.Large.VLDL...Instance.0	3.79E-19	-0.00202	0.000226
UAP	Phospholipids.to.Total.Lipids.in.Small.LDL.percentage...Instance.0	6.74E-19	0.001935	0.000218
UAP	Saturated.Fatty.Acids...Instance.0	9.86E-19	-0.00194	0.00022
UAP	Cholesteryl.Esters.to.Total.Lipids.in.Large.VLDL.percentage...Instance.0	1.67E-18	-0.00201	0.000229
AP	Free.Cholesterol.in.Large.HDL...Instance.0	2.13E-18	-0.0033	0.000378
UAP	Triglycerides.to.Total.Lipids.in.Very.Large.VLDL.percentage...Instance.0	2.58E-18	0.001977	0.000226
AP	Cholesteryl.Esters.in.HDL...Instance.0	3.15E-18	-0.00327	0.000375
UAP	Concentration.of.HDL.Particles...Instance.0	4.10E-18	-0.00208	0.000239
AP	Cholesterol.in.Very.Large.VLDL...Instance.0	4.23E-18	-0.00286	0.00033
AP	Phospholipids.to.Total.Lipids.in.Small.LDL.percentage...Instance.0	5.05E-18	0.002757	0.000319
UAP	Cholesterol.in.Large.VLDL...Instance.0	5.62E-18	-0.00194	0.000225
UAP	Free.Cholesterol.to.Total.Lipids.in.Small.HDL.percentage...Instance.0	5.80E-18	-0.00198	0.00023
UAP	Cholesteryl.Esters.to.Total.Lipids.in.Medium.LDL.percentage...Instance.0	5.80E-18	-0.0019	0.00022
UAP	Concentration.of.Small.VLDL.Particles...Instance.0	6.48E-18	-0.0019	0.00022
AP	Free.Cholesterol.to.Total.Lipids.in.Very.Large.HDL.percentage...Instance.0	8.29E-18	0.00311	0.000362
AP	Concentration.of.Large.HDL.Particles...Instance.0	2.11E-17	-0.00322	0.00038

UAP	Total.Lipids.in.VLDL...Instance.0	2.15E-17	-0.00189	0.000223
UAP	Free.Cholesterol.to.Total.Lipids.in.IDL.percentage...Instance.0	2.70E-17	-0.00186	0.000219
AP	Free.Cholesterol.in.Small.HDL...Instance.0	4.11E-17	-0.00276	0.000328
AP	Saturated.Fatty.Acids...Instance.0	4.22E-17	-0.0027	0.000321
AP	Cholesterol.in.Large.HDL...Instance.0	4.65E-17	-0.00319	0.000379
AP	Concentration.of.Small.VLDL.Particles...Instance.0	5.03E-17	-0.0027	0.000322
AP	Cholesterol.in.Large.VLDL...Instance.0	6.18E-17	-0.00275	0.000328
AP	Phospholipids.in.Very.Large.HDL...Instance.0	9.04E-17	-0.00303	0.000365
AP	Cholesteryl.Esters.in.Large.HDL...Instance.0	1.45E-16	-0.00314	0.000379
UAP	Free.Cholesterol.in.HDL...Instance.0	1.45E-16	-0.00212	0.000256
UAP	Concentration.of.Small.HDL.Particles...Instance.0	2.17E-16	-0.0018	0.000219
UAP	Phospholipids.to.Total.Lipids.in.Very.Large.VLDL.percentage...Instance.0	2.33E-16	-0.00178	0.000217
UAP	Cholesterol.to.Total.Lipids.in.Large.VLDL.percentage...Instance.0	7.21E-16	-0.00178	0.00022
AP	Total.Lipids.in.VLDL...Instance.0	9.52E-16	-0.00262	0.000326
AP	Cholesteryl.Esters.to.Total.Lipids.in.Very.Large.VLDL.percentage...Instance.0	2.58E-15	-0.00265	0.000335
AP	Creatinine...Instance.0	2.70E-15	0.002874	0.000364
AP	Free.Cholesterol.to.Total.Lipids.in.Small.LDL.percentage...Instance.0	4.10E-15	-0.00257	0.000327
UAP	Concentration.of.Very.Large.HDL.Particles...Instance.0	5.00E-15	-0.00195	0.000249
AP	Triglycerides.to.Total.Lipids.in.Large.HDL.percentage...Instance.0	7.07E-15	0.002639	0.000339
AP	Triglycerides.to.Total.Lipids.in.Large.VLDL.percentage...Instance.0	7.94E-15	0.002439	0.000314
UAP	Cholesterol.in.Small.HDL...Instance.0	8.81E-15	-0.00171	0.00022
UAP	Free.Cholesterol.to.Total.Lipids.in.Very.Large.VLDL.percentage...Instance.0	9.25E-15	-0.00175	0.000225
AP	Total.Lipids.in.HDL...Instance.0	1.55E-14	-0.00284	0.000369
UAP	Phospholipids.to.Total.Lipids.in.Medium.HDL.percentage...Instance.0	2.68E-14	0.001837	0.000241
UAP	Free.Cholesterol.to.Total.Lipids.in.Very.Large.HDL.percentage...Instance.0	3.04E-14	0.001878	0.000247
AP	Monounsaturated.Fatty.Acids.to.Total.Fatty.Acids.percentage...Instance.0	4.31E-14	0.002641	0.00035
AP	Cholesteryl.Esters.to.Total.Lipids.in.Medium.LDL.percentage...Instance.0	4.93E-14	-0.00243	0.000322
AP	Cholesterol.to.Total.Lipids.in.Small.HDL.percentage...Instance.0	5.30E-14	-0.00241	0.000321

AP	Apolipoprotein.A1...Instance.0	6.50E-14	-0.00271	0.000361
UAP	Free.Cholesterol.in.Very.Large.HDL...Instance.0	8.76E-14	-0.00179	0.000239
AP	Total.Lipids.in.Large.HDL...Instance.0	1.13E-13	-0.00281	0.000379
AP	Concentration.of.Small.HDL.Particles...Instance.0	1.39E-13	-0.00237	0.000321
UAP	Cholesterol.in.Very.Large.HDL...Instance.0	1.56E-13	-0.00185	0.000251
UAP	Phospholipids.to.Total.Lipids.in.IDL.percentage...Instance.0	2.92E-13	0.001586	0.000217
UAP	Cholesteryl.Esters.in.Very.Large.HDL...Instance.0	3.85E-13	-0.00184	0.000253
UAP	HDL.Cholesterol...Instance.0	1.15E-12	-0.00183	0.000257
AP	Free.Cholesterol.in.Medium.HDL...Instance.0	1.33E-12	-0.00258	0.000364
AP	Cholesterol.in.Small.HDL...Instance.0	1.60E-12	-0.00227	0.000322
AP	Cholesterol.to.Total.Lipids.in.Medium.HDL.percentage...Instance.0	2.73E-12	-0.00242	0.000346
UAP	Cholesteryl.Esters.in.Small.HDL...Instance.0	3.71E-12	-0.00152	0.000219
UAP	Cholesteryl.Esters.to.Total.Lipids.in.Very.Large.HDL.percentage...Instance.0	6.88E-12	-0.00149	0.000217
UAP	Triglycerides.to.Total.Lipids.in.Large.VLDL.percentage...Instance.0	7.21E-12	0.001471	0.000215
AP	Triglycerides.to.Total.Lipids.in.Very.Large.HDL.percentage...Instance.0	9.30E-12	0.002273	0.000333
UAP	Apolipoprotein.A1...Instance.0	9.72E-12	-0.00168	0.000247
UAP	Cholesteryl.Esters.in.HDL...Instance.0	2.25E-11	-0.00172	0.000257
UAP	Average.Diameter.for.LDL.Particles...Instance.0	3.09E-11	-0.00149	0.000225
UAP	Free.Cholesterol.in.Large.VLDL...Instance.0	4.22E-11	-0.0015	0.000228
AP	Cholesteryl.Esters.to.Total.Lipids.in.Very.Large.HDL.percentage...Instance.0	4.67E-11	-0.00209	0.000317
AP	Omega.6.Fatty.Acids.to.Total.Fatty.Acids.percentage...Instance.0	4.84E-11	-0.00223	0.000339
UAP	Free.Cholesterol.in.Very.Large.VLDL...Instance.0	8.29E-11	-0.00147	0.000227
UAP	Total.Lipids.in.HDL...Instance.0	9.55E-11	-0.00164	0.000253
AP	Phospholipids.in.Large.HDL...Instance.0	2.49E-10	-0.00239	0.000377
AP	Phospholipids.in.HDL...Instance.0	3.10E-10	-0.00229	0.000364
UAP	Free.Cholesterol.in.Medium.HDL...Instance.0	3.13E-10	-0.00157	0.000249
AP	Cholesteryl.Esters.in.Small.HDL...Instance.0	3.79E-10	-0.002	0.00032
UAP	Phospholipids.to.Total.Lipids.in.Medium.LDL.percentage...Instance.0	4.13E-10	0.001345	0.000215

AP	Average.Diameter.for.HDL.Particles...Instance.0	4.21E-10	-0.00235	0.000376
UAP	Total.Lipids.in.Small.HDL...Instance.0	5.90E-10	-0.00137	0.000221
UAP	Triglycerides.in.Medium.VLDL...Instance.0	6.56E-10	-0.00138	0.000224
UAP	Total.Lipids.in.Very.Large.HDL...Instance.0	6.70E-10	-0.00154	0.00025
UAP	Triglycerides.to.Total.Lipids.in.Large.HDL.percentage...Instance.0	1.55E-09	0.0014	0.000232
AP	Triglycerides.to.Total.Lipids.in.Small.HDL.percentage...Instance.0	2.46E-09	0.00205	0.000344
UAP	Total.Lipids.in.Large.VLDL...Instance.0	2.52E-09	-0.00135	0.000227
UAP	Free.Cholesterol.to.Total.Lipids.in.Medium.LDL.percentage...Instance.0	3.52E-09	-0.00137	0.000232
UAP	Cholesterol.to.Total.Lipids.in.Very.Large.VLDL.percentage...Instance.0	4.31E-09	-0.00135	0.00023
AP	Polyunsaturated.Fatty.Acids.to.Monounsaturated.Fatty.Acids.ratio...Instance.0	4.75E-09	-0.00204	0.000348
UAP	Free.Cholesterol.in.Large.HDL...Instance.0	7.08E-09	-0.00149	0.000258
UAP	Monounsaturated.Fatty.Acids...Instance.0	9.74E-09	-0.00127	0.000221
UAP	Concentration.of.Large.HDL.Particles...Instance.0	1.26E-08	-0.00148	0.00026
AP	Free.Cholesterol.in.Large.VLDL...Instance.0	1.27E-08	-0.00189	0.000333
UAP	Concentration.of.Large.VLDL.Particles...Instance.0	1.71E-08	-0.00128	0.000227
AP	Free.Cholesterol.in.Very.Large.VLDL...Instance.0	1.76E-08	-0.00187	0.000332
AP	Phospholipids.to.Total.Lipids.in.Small.HDL.percentage...Instance.0	2.88E-08	0.001802	0.000325
UAP	Phospholipids.in.HDL...Instance.0	3.03E-08	-0.00138	0.000249
UAP	Phospholipids.in.Very.Large.VLDL...Instance.0	3.04E-08	-0.00126	0.000228
AP	Concentration.of.Medium.HDL.Particles...Instance.0	3.96E-08	-0.00197	0.000358
AP	Docosahexaenoic.Acid...Instance.0	4.37E-08	-0.00182	0.000332
UAP	Phospholipids.in.Large.VLDL...Instance.0	4.65E-08	-0.00125	0.000228
AP	Polyunsaturated.Fatty.Acids.to.Total.Fatty.Acids.percentage...Instance.0	6.67E-08	-0.00184	0.000342
UAP	Cholesterol.in.Large.HDL...Instance.0	7.55E-08	-0.0014	0.000259
UAP	Phospholipids.in.Small.HDL...Instance.0	8.44E-08	-0.00119	0.000223
AP	Cholesterol.in.Medium.HDL...Instance.0	1.13E-07	-0.00192	0.000361
UAP	Cholesteryl.Esters.in.Chylomicrons.and.Extremely.Large.VLDL...Instance.0	1.21E-07	-0.0012	0.000227
UAP	Cholesteryl.Esters.in.Large.HDL...Instance.0	1.62E-07	-0.00136	0.00026

UAP	Concentration.of.Medium.HDL.Particles...Instance.0	2.15E-07	-0.00127	0.000245
UAP	Free.Cholesterol.to.Total.Lipids.in.Small.LDL.percentage...Instance.0	2.58E-07	-0.00115	0.000223
AP	Triglycerides.in.Medium.VLDL...Instance.0	2.60E-07	-0.00169	0.000327
AP	Phospholipids.to.Total.Lipids.in.Chylomicrons.and.Extremely.Large.VLDL.percentage...Instance.0	2.82E-07	0.001665	0.000324
UAP	Cholesterol.to.Total.Lipids.in.Small.HDL.percentage...Instance.0	4.04E-07	-0.00111	0.000219
SAP	Free.Cholesterol.to.Total.Lipids.in.Large.HDL.percentage...Instance.0	5.41E-07	-0.00048	9.67E-05
AP	Phospholipids.to.Total.Lipids.in.Medium.LDL.percentage...Instance.0	7.93E-07	0.001554	0.000315
UAP	Cholesteryl.Esters.to.Total.Lipids.in.Very.Large.VLDL.percentage...Instance.0	8.12E-07	-0.00113	0.000229
UAP	Phospholipids.in.Very.Large.HDL...Instance.0	8.65E-07	-0.00123	0.000249
AP	Total.Lipids.in.Large.VLDL...Instance.0	8.70E-07	-0.00163	0.000332
UAP	Triglycerides.in.Very.Large.HDL...Instance.0	1.14E-06	-0.00105	0.000215
UAP	Total.Lipids.in.Large.HDL...Instance.0	1.27E-06	-0.00125	0.000259
AP	Cholesteryl.Esters.in.Medium.HDL...Instance.0	1.42E-06	-0.00174	0.00036
UAP	Triglycerides.in.Large.LDL...Instance.0	1.97E-06	-0.00104	0.000219
AP	Phospholipids.to.Total.Lipids.in.Very.Large.HDL.percentage...Instance.0	2.26E-06	-0.00161	0.000341
UAP	Cholesterol.in.Medium.HDL...Instance.0	2.60E-06	-0.00116	0.000247
AP	Free.Cholesterol.to.Total.Lipids.in.Large.VLDL.percentage...Instance.0	3.93E-06	-0.00146	0.000317
UAP	Total.Lipids.in.Very.Large.VLDL...Instance.0	4.09E-06	-0.00105	0.000229
UAP	Triglycerides.in.Large.VLDL...Instance.0	4.17E-06	-0.00104	0.000227
AP	Total.Lipids.in.Small.HDL...Instance.0	4.32E-06	-0.00149	0.000324
AP	Triglycerides.to.Phosphoglycerides.ratio...Instance.0	6.17E-06	0.001602	0.000354
AP	Concentration.of.Large.VLDL.Particles...Instance.0	6.39E-06	-0.0015	0.000332
UAP	Cholesterol.to.Total.Lipids.in.Medium.HDL.percentage...Instance.0	6.75E-06	-0.00107	0.000237
UAP	Triglycerides.in.LDL...Instance.0	6.77E-06	-0.00099	0.00022
SAP	Cholesterol.to.Total.Lipids.in.Very.Small.VLDL.percentage...Instance.0	7.18E-06	-0.00044	9.84E-05
AP	Degree.of.Unsaturation...Instance.0	7.63E-06	-0.00151	0.000337
UAP	Phospholipids.to.Total.Lipids.in.Small.HDL.percentage...Instance.0	8.26E-06	0.00099	0.000222
UAP	Creatinine...Instance.0	9.12E-06	0.001105	0.000249

SAP	Cholesteryl.Esters.to.Total.Lipids.in.Very.Small.VLDL.percentage...Instance.0	9.31E-06	-0.00044	9.95E-05
UAP	Triglycerides.to.Total.Lipids.in.Very.Large.HDL.percentage...Instance.0	9.61E-06	0.001009	0.000228
SAP	Triglycerides.to.Total.Lipids.in.Very.Small.VLDL.percentage...Instance.0	9.92E-06	0.000431	9.75E-05
AP	Cholesteryl.Esters.to.Total.Lipids.in.Small.HDL.percentage...Instance.0	1.01E-05	-0.0014	0.000318
UAP	Cholesterol.in.Chylomicrons.and.Extremely.Large.VLDL...Instance.0	1.02E-05	-0.001	0.000227
AP	Phospholipids.in.Very.Large.VLDL...Instance.0	1.06E-05	-0.00147	0.000334
AP	Cholesteryl.Esters.in.Chylomicrons.and.Extremely.Large.VLDL...Instance.0	1.12E-05	-0.00146	0.000332
UAP	Concentration.of.Very.Large.VLDL.Particles...Instance.0	1.14E-05	-0.00101	0.000229
SAP	Free.Cholesterol.in.IDL...Instance.0	1.20E-05	-0.00041	9.37E-05
AP	Triglycerides.to.Total.Lipids.in.Medium.HDL.percentage...Instance.0	1.31E-05	0.00147	0.000337
AP	Triglycerides.in.Very.Large.HDL...Instance.0	1.37E-05	-0.00137	0.000315
AP	Monounsaturated.Fatty.Acids...Instance.0	1.75E-05	-0.00139	0.000324
UAP	Total.Lipids.in.Medium.HDL...Instance.0	1.91E-05	-0.00103	0.000242
UAP	Cholesteryl.Esters.in.Medium.HDL...Instance.0	1.95E-05	-0.00105	0.000246
AP	Phospholipids.in.Large.VLDL...Instance.0	2.93E-05	-0.00139	0.000334
UAP	Free.Cholesterol.to.Total.Lipids.in.Large.VLDL.percentage...Instance.0	3.08E-05	-0.0009	0.000217
AP	Total.Lipids.in.Medium.HDL...Instance.0	3.37E-05	-0.00147	0.000354
SAP	Cholesterol.in.IDL...Instance.0	3.38E-05	-0.00039	9.41E-05
UAP	Phospholipids.in.Large.HDL...Instance.0	4.03E-05	-0.00106	0.000258
AP	Omega.6.Fatty.Acids.to.Omega.3.Fatty.Acids.ratio...Instance.0	4.15E-05	-0.00133	0.000324
AP	Glutamine...Instance.0	4.29E-05	0.001298	0.000317
UAP	Triglycerides.in.IDL...Instance.0	4.51E-05	-0.00089	0.000219
UAP	Phospholipids.to.Total.Lipids.in.Chylomicrons.and.Extremely.Large.VLDL.percentage...Instance.0	5.40E-05	0.000894	0.000221
SAP	Docosahexaenoic.Acid...Instance.0	5.53E-05	-0.00038	9.33E-05
SAP	Cholesteryl.Esters.in.IDL...Instance.0	5.70E-05	-0.00038	9.41E-05
UAP	Monounsaturated.Fatty.Acids.to.Total.Fatty.Acids.percentage...Instance.0	5.76E-05	0.000962	0.000239
SAP	Cholesterol.to.Total.Lipids.in.Large.HDL.percentage...Instance.0	5.85E-05	-0.0004	9.93E-05
AP	X3.Hydroxybutyrate...Instance.0	5.85E-05	-0.00126	0.000315

UAP	Triglycerides.in.Medium.LDL...Instance.0	5.96E-05	-0.00089	0.000221
AP	Cholesteryl.Esters.to.Total.Lipids.in.Medium.HDL.percentage...Instance.0	6.44E-05	-0.00134	0.000335
UAP	Omega.6.Fatty.Acids.to.Total.Fatty.Acids.percentage...Instance.0	7.58E-05	-0.00092	0.000232
SAP	Free.Cholesterol.to.Total.Lipids.in.Medium.VLDL.percentage...Instance.0	7.66E-05	-0.00038	9.59E-05
SAP	Free.Cholesterol.to.Total.Lipids.in.Medium.HDL.percentage...Instance.0	9.40E-05	-0.00041	0.000106
SAP	Total.Lipids.in.IDL...Instance.0	0.000105737	-0.00036	9.35E-05
SAP	Triglycerides.to.Total.Lipids.in.Medium.VLDL.percentage...Instance.0	0.000109919	0.000376	9.73E-05
SAP	Sphingomyelins...Instance.0	0.000110195	-0.00037	9.69E-05
SAP	Phospholipids.to.Total.Lipids.in.Medium.VLDL.percentage...Instance.0	0.000130228	-0.00036	9.45E-05
UAP	Total.Triglycerides...Instance.0	0.00013249	-0.00086	0.000225
UAP	Triglycerides.in.VLDL...Instance.0	0.000133846	-0.00087	0.000227
UAP	Triglycerides.in.Small.LDL...Instance.0	0.000146028	-0.00085	0.000223
SAP	Cholesterol.to.Total.Lipids.in.Medium.VLDL.percentage...Instance.0	0.00014984	-0.00037	9.78E-05
UAP	Average.Diameter.for.HDL.Particles...Instance.0	0.000150719	-0.00097	0.000257
UAP	Omega.6.Fatty.Acids.to.Omega.3.Fatty.Acids.ratio...Instance.0	0.000158639	-0.00084	0.000221
SAP	Cholesterol.in.Very.Large.HDL...Instance.0	0.000176935	-0.00039	0.000103
SAP	Phospholipids.in.IDL...Instance.0	0.000188768	-0.00035	9.33E-05
SAP	Cholesteryl.Esters.in.Very.Large.HDL...Instance.0	0.000192314	-0.00039	0.000104
SAP	Cholesteryl.Esters.in.Very.Small.VLDL...Instance.0	0.000201624	-0.00035	9.31E-05
SAP	Phospholipids.to.Total.Lipids.in.Large.HDL.percentage...Instance.0	0.000201797	0.000366	9.84E-05
SAP	Free.Cholesterol.to.Total.Lipids.in.Large.LDL.percentage...Instance.0	0.000203287	-0.00036	9.70E-05
SAP	Free.Cholesterol.to.Total.Lipids.in.Very.Small.VLDL.percentage...Instance.0	0.000215921	-0.00033	9.00E-05
SAP	Cholesteryl.Esters.to.Total.Lipids.in.Medium.VLDL.percentage...Instance.0	0.000236325	-0.00036	9.83E-05
UAP	Phospholipids.in.Medium.HDL...Instance.0	0.000243608	-0.00088	0.000239
SAP	Triglycerides.to.Total.Lipids.in.IDL.percentage...Instance.0	0.000264167	0.000352	9.64E-05
SAP	Free.Cholesterol.in.Very.Large.HDL...Instance.0	0.00027078	-0.00036	9.81E-05
AP	Phospholipids.in.Small.HDL...Instance.0	0.000275342	-0.00118	0.000326
SAP	Total.Esterified.Cholesterol...Instance.0	0.000316096	-0.00034	9.41E-05

SAP	Triglycerides.to.Total.Lipids.in.Very.Large.VLDL.percentage...Instance.0	0.000346376	0.000333	9.32E-05
SAP	Concentration.of.Very.Large.HDL.Particles...Instance.0	0.000367362	-0.00036	0.000102
SAP	Free.Cholesterol.to.Total.Lipids.in.IDL.percentage...Instance.0	0.000380759	-0.00032	8.99E-05
SAP	Concentration.of.IDL.Particles...Instance.0	0.000389422	-0.00032	9.09E-05
SAP	Total.Cholesterol...Instance.0	0.000427895	-0.00033	9.35E-05
SAP	Total.Lipids.in.Very.Large.HDL...Instance.0	0.000443654	-0.00036	0.000102
SAP	Free.Cholesterol.in.Large.LDL...Instance.0	0.00044899	-0.00032	9.19E-05
SAP	Free.Cholesterol.to.Total.Lipids.in.Very.Large.VLDL.percentage...Instance.0	0.000468018	-0.00032	9.27E-05
UAP	Docosahexaenoic.Acid...Instance.0	0.000498934	-0.00079	0.000227
SAP	Free.Cholesterol.to.Total.Lipids.in.Small.VLDL.percentage...Instance.0	0.000545419	-0.00033	9.57E-05
SAP	Free.Cholesterol.in.Large.HDL...Instance.0	0.000564075	-0.00037	0.000106
SAP	Cholesterol.in.Very.Small.VLDL...Instance.0	0.000630356	-0.00032	9.22E-05
UAP	Albumin...Instance.0	0.000718595	-0.00074	0.000219
SAP	Cholesterol.in.Large.HDL...Instance.0	0.000737333	-0.00036	0.000106
SAP	Concentration.of.Large.HDL.Particles...Instance.0	0.000773041	-0.00036	0.000107
SAP	Cholesterol.to.Total.Lipids.in.IDL.percentage...Instance.0	0.000819803	-0.00032	9.49E-05
SAP	Phospholipids.in.Very.Large.HDL...Instance.0	0.000824009	-0.00034	0.000102
SAP	Cholesteryl.Esters.in.Large.HDL...Instance.0	0.000833746	-0.00036	0.000106
SAP	Cholesteryl.Esters.to.Total.Lipids.in.Large.HDL.percentage...Instance.0	0.000859134	-0.00033	9.79E-05
AP	Triglycerides.in.Large.VLDL...Instance.0	0.000932731	-0.0011	0.000332
UAP	Triglycerides.to.Total.Lipids.in.Small.HDL.percentage...Instance.0	0.000956285	0.000776	0.000235
UAP	Free.Cholesterol.in.Chylomicrons.and.Extremely.Large.VLDL...Instance.0	0.001048119	-0.00074	0.000227
SAP	Free.Cholesterol.in.HDL...Instance.0	0.001049497	-0.00034	0.000105
UAP	Glycine...Instance.0	0.001073351	-0.00075	0.00023
SAP	Phospholipids.to.Total.Lipids.in.Small.VLDL.percentage...Instance.0	0.001104161	-0.00031	9.50E-05
SAP	Total.Free.Cholesterol...Instance.0	0.001121088	-0.0003	9.20E-05
SAP	Triglycerides.to.Total.Lipids.in.Small.VLDL.percentage...Instance.0	0.001134516	0.000303	9.32E-05
SAP	Total.Lipids.in.Large.HDL...Instance.0	0.001205746	-0.00034	0.000106

AP	Omega.3.Fatty.Acids...Instance.0	0.001227492	-0.00105	0.000324
UAP	Omega.3.Fatty.Acids.to.Total.Fatty.Acids.percentage...Instance.0	0.001265745	0.000714	0.000221
SAP	Free.Cholesterol.in.LDL...Instance.0	0.001307385	-0.00029	9.08E-05
AP	Cholesterol.in.Chylomicrons.and.Extremely.Large.VLDL...Instance.0	0.001347844	-0.00106	0.000332
SAP	Docosahexaenoic.Acid.to.Total.Fatty.Acids.percentage...Instance.0	0.001363121	-0.0003	9.28E-05
AP	Phospholipids.in.Medium.HDL...Instance.0	0.001526745	-0.00111	0.000349
SAP	Cholesterol.to.Total.Lipids.in.Small.VLDL.percentage...Instance.0	0.001534148	-0.00029	9.23E-05
SAP	Triglycerides.to.Total.Lipids.in.Large.HDL.percentage...Instance.0	0.001604709	0.0003	9.51E-05
AP	Total.Lipids.in.Very.Large.VLDL...Instance.0	0.001705197	-0.00105	0.000335
SAP	Cholesteryl.Esters.in.Medium.VLDL...Instance.0	0.00171131	-0.00029	9.09E-05
SAP	Cholesteryl.Esters.to.Total.Lipids.in.Large.VLDL.percentage...Instance.0	0.001736143	-0.00029	9.36E-05
SAP	HDL.Cholesterol...Instance.0	0.001917023	-0.00033	0.000105
SAP	Phospholipids.in.Large.HDL...Instance.0	0.001950701	-0.00033	0.000106
SAP	Average.Diameter.for.HDL.Particles...Instance.0	0.002028698	-0.00033	0.000105
AP	Triglycerides.in.Large.LDL...Instance.0	0.002037042	-0.00099	0.000321
SAP	Cholesteryl.Esters.in.HDL...Instance.0	0.002594606	-0.00032	0.000105
UAP	Docosahexaenoic.Acid.to.Total.Fatty.Acids.percentage...Instance.0	0.002668822	0.000679	0.000226
AP	Citrate...Instance.0	0.002780758	-0.00096	0.000321
SAP	Clinical.LDL.Cholesterol...Instance.0	0.002834772	-0.00027	9.03E-05
UAP	X3.Hydroxybutyrate...Instance.0	0.002854603	-0.00064	0.000215
SAP	Free.Cholesterol.to.Total.Lipids.in.Very.Large.HDL.percentage...Instance.0	0.002975336	0.000301	0.000101
SAP	Cholesterol.to.Total.Lipids.in.Large.LDL.percentage...Instance.0	0.003090942	-0.00028	9.45E-05
SAP	Phospholipids.in.Large.LDL...Instance.0	0.003180346	-0.00027	9.05E-05
SAP	Cholesterol.in.Large.LDL...Instance.0	0.00322631	-0.00027	9.07E-05
SAP	Total.Cholines...Instance.0	0.003761605	-0.00028	9.63E-05
AP	Concentration.of.Very.Large.VLDL.Particles...Instance.0	0.003969496	-0.00096	0.000335
SAP	Total.Phospholipids.in.Lipoprotein.Particles...Instance.0	0.004081641	-0.00027	9.47E-05
SAP	Phosphatidylcholines...Instance.0	0.004263942	-0.00028	9.67E-05

SAP	Remnant.Cholesterol..Non.HDL..Non.LDL..Cholesterol....Instance.0	0.004376967	-0.00026	9.03E-05
SAP	Free.Cholesterol.to.Total.Lipids.in.Small.HDL.percentage...Instance.0	0.004617935	-0.00027	9.42E-05
SAP	Triglycerides.to.Total.Lipids.in.Large.LDL.percentage...Instance.0	0.004619238	0.000266	9.40E-05
SAP	Total.Lipids.in.Large.LDL...Instance.0	0.004799592	-0.00026	9.04E-05
SAP	Cholesterol.to.Total.Lipids.in.Large.VLDL.percentage...Instance.0	0.005065421	-0.00025	9.02E-05
SAP	Total.Cholesterol.Minus.HDL.C...Instance.0	0.005417667	-0.00025	9.01E-05
UAP	Phospholipids.to.Total.Lipids.in.Large.LDL.percentage...Instance.0	0.005540576	0.000598	0.000215
AP	Leucine...Instance.0	0.00558839	0.000911	0.000329
SAP	Free.Cholesterol.in.Small.LDL...Instance.0	0.00595011	-0.00025	8.93E-05
SAP	Phospholipids.to.Total.Lipids.in.Very.Large.HDL.percentage...Instance.0	0.005962198	-0.00026	9.57E-05
UAP	Glucose...Instance.0	0.005969069	0.000654	0.000238
AP	Triglycerides.in.LDL...Instance.0	0.006233925	-0.00088	0.000322
UAP	Cholesteryl.Esters.to.Total.Lipids.in.Small.HDL.percentage...Instance.0	0.006620235	-0.00059	0.000217
SAP	Cholesteryl.Esters.in.Large.LDL...Instance.0	0.006675929	-0.00024	9.03E-05
AP	Lactate...Instance.0	0.006955696	-0.00085	0.000313
SAP	Phospholipids.to.Total.Lipids.in.Very.Large.VLDL.percentage...Instance.0	0.007159915	-0.00024	8.93E-05
AP	Triglycerides.in.Small.HDL...Instance.0	0.00727022	0.000896	0.000334
UAP	Citrate...Instance.0	0.007280863	-0.00059	0.00022
SAP	Total.Lipids.in.HDL...Instance.0	0.00752563	-0.00028	0.000104
SAP	Triglycerides.to.Total.Lipids.in.Very.Large.HDL.percentage...Instance.0	0.007612734	0.000249	9.35E-05
SAP	Monounsaturated.Fatty.Acids.to.Total.Fatty.Acids.percentage...Instance.0	0.007624407	0.000262	9.81E-05
UAP	Triglycerides.in.Very.Large.VLDL...Instance.0	0.007797838	-0.00061	0.00023
UAP	Phospholipids.to.Total.Lipids.in.Very.Large.HDL.percentage...Instance.0	0.007815521	-0.00062	0.000233
SAP	Cholesterol.to.Total.Lipids.in.Very.Large.VLDL.percentage...Instance.0	0.008038164	-0.00025	9.46E-05
SAP	Cholesterol.in.Medium.VLDL...Instance.0	0.008355345	-0.00024	8.99E-05
AP	Glycine...Instance.0	0.008568756	-0.00088	0.000336
SAP	Cholesteryl.Esters.to.Total.Lipids.in.Small.VLDL.percentage...Instance.0	0.008779239	-0.00024	8.99E-05
UAP	Triglycerides.in.Large.HDL...Instance.0	0.008852429	-0.00058	0.00022

SAP	LDL.Cholesterol...Instance.0	0.009002035	-0.00023	8.98E-05
UAP	Total.Lipids.in.Chylomicrons.and.Extremely.Large.VLDL...Instance.0	0.009299896	-0.00059	0.000227
SAP	Free.Cholesterol.in.Very.Small.VLDL...Instance.0	0.009325523	-0.00024	9.05E-05
SAP	Cholesterol.to.Total.Lipids.in.Medium.LDL.percentage...Instance.0	0.009333387	-0.00024	9.11E-05
SAP	Free.Cholesterol.in.Medium.LDL...Instance.0	0.009388484	-0.00023	8.95E-05
SAP	Phosphoglycerides...Instance.0	0.010064032	-0.00025	9.53E-05
UAP	Triglycerides.in.Small.VLDL...Instance.0	0.010508558	-0.00057	0.000224
UAP	Polyunsaturated.Fatty.Acids.to.Monounsaturated.Fatty.Acids.ratio...Instance.0	0.010807995	-0.00061	0.000238
SAP	Phospholipids.to.Total.Lipids.in.Very.Small.VLDL.percentage...Instance.0	0.011722153	0.000236	9.38E-05
SAP	Phospholipids.in.LDL...Instance.0	0.012300354	-0.00022	8.96E-05
UAP	Concentration.of.Chylomicrons.and.Extremely.Large.VLDL.Particles...Instance.0	0.012310942	-0.00057	0.000227
AP	Cholesteryl.Esters.to.Total.Lipids.in.Chylomicrons.and.Extremely.Large.VLDL.percentage...Instance.0	0.012359465	-0.0008	0.000321
SAP	Phospholipids.to.Total.Lipids.in.Medium.HDL.percentage...Instance.0	0.012631319	0.000247	9.89E-05
UAP	Omega.3.Fatty.Acids...Instance.0	0.013318992	-0.00055	0.000221
SAP	Free.Cholesterol.in.Medium.HDL...Instance.0	0.014386791	-0.00025	0.000102
SAP	Total.Lipids.in.LDL...Instance.0	0.014494889	-0.00022	8.96E-05
SAP	Degree.of.Unsaturation...Instance.0	0.014609506	-0.00023	9.47E-05
UAP	Phospholipids.in.Chylomicrons.and.Extremely.Large.VLDL...Instance.0	0.014649083	-0.00056	0.000228
UAP	Polyunsaturated.Fatty.Acids.to.Total.Fatty.Acids.percentage...Instance.0	0.014863214	-0.00057	0.000233
SAP	Omega.3.Fatty.Acids...Instance.0	0.014903173	-0.00022	9.09E-05
SAP	Total.Concentration.of.Lipoprotein.Particles...Instance.0	0.015431044	-0.00024	9.77E-05
SAP	Phospholipids.in.HDL...Instance.0	0.016486144	-0.00024	0.000102
AP	Triglycerides.in.IDL...Instance.0	0.016635351	-0.00077	0.00032
SAP	Concentration.of.Large.LDL.Particles...Instance.0	0.018213682	-0.00021	8.94E-05
SAP	Cholesteryl.Esters.to.Total.Lipids.in.IDL.percentage...Instance.0	0.018373124	-0.00022	9.44E-05
SAP	Apolipoprotein.B...Instance.0	0.018377618	-0.00021	8.95E-05
SAP	Cholesteryl.Esters.in.LDL...Instance.0	0.01853862	-0.00021	8.95E-05
SAP	Triglycerides.to.Total.Lipids.in.Small.LDL.percentage...Instance.0	0.018777331	0.000218	9.26E-05

UAP	Cholesteryl.Esters.to.Total.Lipids.in.Chylomicrons.and.Extremely.Large.VLDL.percentage...Instance.0	0.019369441	-0.00051	0.000219
SAP	Apolipoprotein.A1...Instance.0	0.019929432	-0.00024	0.000101
SAP	Concentration.of.Very.Small.VLDL.Particles...Instance.0	0.020181937	-0.00021	9.01E-05
AP	Isoleucine...Instance.0	0.020660123	0.000744	0.000321
AP	Tyrosine...Instance.0	0.020912138	0.000738	0.000319
SAP	Total.Lipids.in.Lipoprotein.Particles...Instance.0	0.021217824	-0.00021	9.07E-05
AP	Acetoacetate...Instance.0	0.024212175	-0.00071	0.000313
SAP	Triglycerides.to.Phosphoglycerides.ratio...Instance.0	0.02440253	0.000224	9.94E-05
AP	Triglycerides.in.VLDL...Instance.0	0.024472419	-0.00075	0.000332
AP	Omega.3.Fatty.Acids.to.Total.Fatty.Acids.percentage...Instance.0	0.025245527	0.000725	0.000324
SAP	Cholesteryl.Esters.to.Total.Lipids.in.Very.Large.VLDL.percentage...Instance.0	0.025990208	-0.00021	9.41E-05
SAP	Cholesterol.to.Total.Lipids.in.Medium.HDL.percentage...Instance.0	0.02801946	-0.00021	9.71E-05
SAP	Concentration.of.LDL.Particles...Instance.0	0.028973727	-0.0002	8.93E-05
SAP	Average.Diameter.for.LDL.Particles...Instance.0	0.030345673	-0.0002	9.22E-05
SAP	Polyunsaturated.Fatty.Acids...Instance.0	0.030369915	-0.0002	9.08E-05
SAP	Glutamine...Instance.0	0.030582346	0.000192	8.90E-05
SAP	Omega.3.Fatty.Acids.to.Total.Fatty.Acids.percentage...Instance.0	0.030598776	-0.0002	9.09E-05
SAP	Triglycerides.to.Total.Lipids.in.Small.HDL.percentage...Instance.0	0.03162077	0.000207	9.64E-05
SAP	Cholesterol.in.Medium.HDL...Instance.0	0.031660589	-0.00022	0.000101
AP	Phenylalanine...Instance.0	0.032025025	0.000671	0.000313
SAP	Concentration.of.HDL.Particles...Instance.0	0.03354315	-0.00021	9.81E-05
AP	Total.Triglycerides...Instance.0	0.033966885	-0.0007	0.000329
SAP	Phospholipids.in.Small.LDL...Instance.0	0.033968241	-0.00019	8.89E-05
SAP	Total.Lipids.in.Very.Small.VLDL...Instance.0	0.034270327	-0.00019	9.02E-05
AP	Triglycerides.in.Medium.LDL...Instance.0	0.035621282	-0.00068	0.000323
SAP	Free.Cholesterol.to.Total.Lipids.in.Medium.LDL.percentage...Instance.0	0.035724166	-0.0002	9.51E-05
UAP	Phospholipids.to.Total.Lipids.in.Large.VLDL.percentage...Instance.0	0.037505129	-0.00047	0.000226
UAP	Triglycerides.to.Phosphoglycerides.ratio...Instance.0	0.037561733	0.000504	0.000242

SAP	Alanine...Instance.0	0.038339492	0.000185	8.91E-05
SAP	Cholesterol.to.Total.Lipids.in.Small.LDL.percentage...Instance.0	0.038424951	-0.00019	9.03E-05
UAP	Triglycerides.to.Total.Lipids.in.Medium.HDL.percentage...Instance.0	0.038571203	0.000477	0.000231
UAP	Acetoacetate...Instance.0	0.039150349	-0.00044	0.000214
SAP	Cholesteryl.Esters.in.Medium.HDL...Instance.0	0.03953584	-0.00021	0.000101
SAP	Triglycerides.to.Total.Lipids.in.Medium.LDL.percentage...Instance.0	0.039671242	0.000189	9.18E-05
SAP	Polyunsaturated.Fatty.Acids.to.Monounsaturated.Fatty.Acids.ratio...Instance.0	0.039808265	-0.0002	9.76E-05
SAP	Cholesterol.in.Small.LDL...Instance.0	0.039934873	-0.00018	8.92E-05
AP	Glucose...Instance.0	0.040729138	0.000712	0.000348
AP	Triglycerides.in.Small.LDL...Instance.0	0.041305203	-0.00066	0.000325
UAP	Free.Cholesterol.to.Total.Lipids.in.Chylomicrons.and.Extremely.Large.VLDL.percentage...Instance.0	0.042450497	0.000451	0.000222
SAP	Triglycerides.in.Small.HDL...Instance.0	0.043660437	0.000189	9.35E-05
UAP	Triglycerides.in.Chylomicrons.and.Extremely.Large.VLDL...Instance.0	0.043806323	-0.00046	0.000227
SAP	Concentration.of.Medium.HDL.Particles...Instance.0	0.043993811	-0.0002	0.0001
UAP	Triglycerides.in.Very.Small.VLDL...Instance.0	0.044067207	-0.00044	0.00022
UAP	Cholesteryl.Esters.to.Total.Lipids.in.Medium.HDL.percentage...Instance.0	0.048448147	-0.00045	0.000229
